# Synthesis of thiazolidin-4-ones and thiazinan-4-ones from 1-(2-aminoethyl)pyrrolidine as acetylcholinesterase inhibitors

**DOI:** 10.1080/14756366.2019.1680659

**Published:** 2019-10-23

**Authors:** Adriana M. das Neves, Gabriele A. Berwaldt, Cinara T. Avila, Taís B. Goulart, Bruna C. Moreira, Taís P. Ferreira, Mayara S. P. Soares, Nathalia S. Pedra, Luiza Spohr, Anita A. A. dE Souza, Roselia M. Spanevello, Wilson Cunico

**Affiliations:** a Laboratório de Química Aplicada a Bioativos, Centro Ciências Químicas, Farmacêuticas e de Alimentos, Universidade Federal de Pelotas, Capão do Leão, Brazil; b Laboratório de Neuroquímica, Inflamação e Câncer, Centro de Ciências Químicas Farmacêuticas e de Alimentos, Universidade Federal de Pelotas, Capão do Leão, Brazil

**Keywords:** Thiazolidin-4-ones, thiazinan-4-ones, pyrrolidine, acetylcholinesterase inhibitors, astrocytes

## Abstract

The present study describes the synthesis of a novel series of thiazolidin-4-one and thiazinan-4-one using 1-(2-aminoethyl)pyrrolidine as amine precursor. All compounds were synthesised by one-pot three component cyclocondensation reaction from the amine, a substituted benzaldehyde and a mercaptocarboxylic acid. The compounds were obtained in moderate to good yields and were identified and characterised by ^1^H, ^13 ^C, 2 D NMR and GC/MS techniques. The compounds also were screened for their *in vitro* acetylcholinesterase (AChE) activity in hippocampus and cerebral cortex on Wistar rats. The six most potent compounds have been investigated for their cytotoxicity by cell viability assay of astrocyte primary culture, an important cell of central nervous system. We highlighted two compounds (**6a** and **6k**) that had the lowest IC_50_ in hippocampus (5.20 and 4.46 µM) and cerebral cortex (7.40 and 6.83 µM). These preliminary and important results could be considered a starting point for the development of new AChE inhibitory agents.

## Introduction

Currently, the main cause of dementia in the elderly is Alzheimer’s disease (AD), which can impair cognitive and memory aspects. AD is responsible for about 60–80% of all dementia types in the elderly and is considered to be a progressive and irreversible neurodegenerative disease^1^. It is estimated that in 2015 more than 46.8 million people worldwide had dementia and this number is expected to double every 20 years reaching almost 131.5 million in 2050^2^. The etiopathogenesis of AD still remains unknown although but several involved factors have been identified and found consistent with its onset. Among them is the impairment of cholinergic system, as indicated by the presence of altered cholinergic markers in AD patients. This results in a pronounced acetylcholine (ACh) deficiency which translates into a generalised withdrawal of cholinergic tone^3^.

ACh is a cholinergic neurotransmitter, which may exert its effects via muscarinic and nicotinic receptors found throughout the central nervous system. However, once in the synaptic cleft, the ACh can still be hydrolysed in a reaction catalysed by the enzyme acetylcholinesterase (AChE), giving rise to acetate and choline, thus interrupting the signalling mediated by ACh. The progressive loss of cholinergic neurons contribute significantly to the memory and attention deficits found in AD^4^.

Although the exact mechanisms involved in the pathogenesis of AD are not yet known, the findings on the subject in the last decades encourage the search for new pharmacological therapies more effective and with fewer side effects. Thus, research suggests that specific dysfunctions in cholinergic transmission may be responsible for the decline in memory observed in older people^5^. AChE competitive inhibitors are widely used drugs to treat AD patients. AChE inhibitors can produce promising results increasing ACh levels in the synaptic cleft and partially ameliorate cognitive symptoms, enhancing the quality of life and diminish caregiver burden for patients with AD^4^.

There are currently four drugs approved treatments by US Food and Drug Administration that act as an AChE inhibitor and that are used to treat the cognitive manifestations of AD: rivastigmine (Exelon), galantamine (Razadyne, Reminyl), tacrine (Cognex) and donepezil (Aricept)^3^^,^^6^. Tacrine was the first approved potent and clinically effective AChE inhibitor in 1993 and was withdrawn from the pharmaceutical market in 1998 due to hepatotoxicity^7^. In general, galantamine, rivastigmine and donezepil are most effective when treatment is initiated in the early stages of disease. Rivastigmine is able to block butyrylcholinesterase, while galantamine modulates nicotinic acetylcholine receptors. On the other hand, donepezil prevents the synthesis of Aβ through the inhibition A*β* self-aggregation and *β*-secretase, as well as being able to interact with the sigma-1 receptors, which have anti-amnesic function^6^^,^^8^.

Although the inhibition of AChE is a widely used therapeutic approach for AD, this strategy only softens the symptoms of this disease and is, therefore, a palliative treatment. Moreover, AChE inhibitors have extensive side effects and limited beneficial effects^9^. Thus, several studies have focussed on the search for more effective therapies and with fewer adverse effects for AD. In this sense, medicinal chemistry has an important role to design, synthesise and develop new molecules with biological activity through the use of different strategies of detection for possible drugs^8^.

Heterocyclic compounds, with nitrogen and sulphur atoms and five-member and six-member rings, are of great interest in the field of synthetic organic chemistry and medicinal chemistry^10^^,^^11^. Thiazolidinone belongs to this class and is a versatile scaffold to the development of new bioactive compounds. Numerous studies describe a wide range of pharmacological properties of thiazolidinones: anti-HIV^12^, antitumor^13^^,^^14^, anti-inflammatory^15^, antimicrobial^16^, antihyperglycemic^17^, and acetyl/butyrylcholinesterase inhibition^5^. Thiazinanones (six-membered heterocycle) also show important biological activity such as anticancer^18^, antimalarial^19^, antihypertensive^20^, antibacterial^21^, and antihyperglycemic^17^.

**Figure 1.**
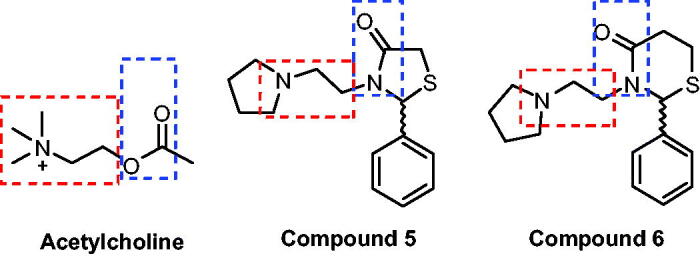
Similarity at chemical structure of acetylcholine, thiazolidin-4-ones (**5**) and thiazinan-4-ones (**6**).

Both thiazolidin-4-ones and thiazinan-4-ones have similar syntheses strategy. The main synthetic route involves three components (an aldehyde or ketone, a primary amine, and carboxylic acid). The reactions proceed by initial formation of an imine followed by intramolecular cyclisation and elimination of water. Moreover, these methods can be applied in two-steps, one step multicomponent (all reactants together at the beginning of reaction) or one-step one-pot reactions, under catalysis or not^22^. We have been studying these classes of heterocycles in a few years^23–26^ and in continuation of our research programme, this work aims to synthesise novel thiazolidin-4-ones and thiazinan-4-ones from a specific amine, the 1–(2-aminoethyl)pyrrolidine, affording compounds structurally similar to AChE that could act as AChE inhibitors (Figure 1). The same strategy was used in previous work with good results^27^. Another goal is the AChE inhibitory activity and cytotoxicity of synthesised compounds, contributing to the search for new compounds that can be used for the AD treatment.

## Experimental

### Chemistry

Reagents and solvents were used as obtained from commercial suppliers without further purification. The melting points were determined on a Fisatom brand machine with three capillary tubes, model 430, 230 V, 60 Hz, 50 W. Thermometer was set up to 360 °C. The characterisation of the molecules was performed using Gas Chromatograph coupled Mass Spectra (GC/MS) and ^1^H, ^13 ^C and 2 D nuclear magnetic resonance spectroscopy (NMR). The mass spectra were obtained on a Shimadzu GCMS-QP2010SE with a split-splitless injector and equipped with a RDX-SMS capillary column (30 m × 0.25 mm × 0.25 μm); helium was used as the carrier gas (56 kPa). ^1^H and ^13 ^C NMR spectra were recorded on a Bruker Ac-200F (^1^H de 200 MHz e ^13 ^C de 50 MHz), on a Bruker Avance 600 spectrometer (^1^H at 600 MHz and ^13 ^C at 150 MHz), in CDCl_3_ containing tetramethylsilane (TMS) as an internal standard. 2 D-NMR (COSY, HSQC and HMBC) spectra were obtained using the Bruker Ac-200F (250 MHz) spectrophotometer in chloroform (CDCl_3_) containing TMS as an internal standard. Spectra processing was performed using the FID file using the Magnetic Resonance Companion NMR Manager (MestReC), MestReNOVA 6.0.2-5475 and Advanced Chemistry Development (ACD 2 D NMR Manager). Mass spectra were obtained for all compounds on an LTQ Orbitrap Discovery mass spectrometer (Thermo Fisher Scientific). This hybrid system meets the LTQ XL linear ion trap mass spectrometer and an Orbitrap mass analyser. The experiments were performed via direct infusion of sample (flow: 15 μL/min) in the positive-ion mode using electrospray ionisation. Elemental composition calculations for comparison were executed using the specific tool included in the Qual Browser module of Xcalibur (Thermo Fisher Scientific, release 2.0.7) software.

#### General procedure for the synthesis of thiazolidin-4-ones 5a-u and thiazinan-4-ones 6a-s and 6v-w

The reaction was carried out of 1-(2-aminoethyl)pyrrolidine **1** (1 mmol) and arenealdehyde **2a-x** (1 mmol), under stirring, reflux of toluene (70 ml) and a Dean-Stark system for 2 h. Thereafter, a mercaptocarboxylic acid (3 mmol) was added in the reaction. The mercaptoacetic acid **4a** was used to obtain thiazolidin-4-ones **5** and the mercaptopropionic acid **4 b** was used to obtain thiazinan-4-ones **6**. The reaction mixture was maintaining in reflux of toluene for more 5 h and then it was neutralised with saturated NaHCO_3_ solution (30 ml) to separate into two phases: the organic phase and the aqueous phase. The aqueous phase was washed with ethyl acetate (2 × 10 ml) and the organic phases were combined. The organic phase was dried using anhydrous magnesium sulphate, filtered and the solvent was removed under reduced pressure to afford the products. Some thiazolidin-4-ones (**5n**, **5t**, **5 u**) and thiazinan-4-ones (**6c**, **6e**, **6g**, **6h**, **6j**, **6k**, **6 l**, **6n**, **6p**, **6q**, **6r**, **6x**, **6w**) required purification by washing hot hexane or hot hexane/ethyl acetate (8:2).
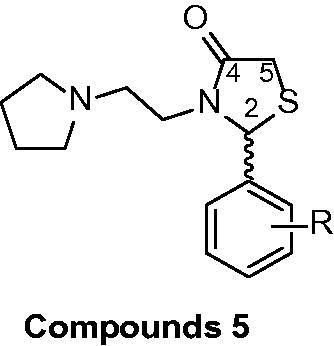


##### 2-phenyl-3-(2-(pyrrolidin-1-yl)ethyl)thiazolidin-4-one (5a)

Yield: 84%, oil, ^1^H NMR (400 MHz, CDCl_3_) δ (ppm, *J*_H-H_= Hz): 7.38–7.34 (m, 3H, Ar), 7.30 (dd, 2H, Ar, ^3 ^*J=* 10.2, ^4 ^*J=* 4.2), 5.86 (d, 1H, H2, ^4 ^*J* = 1.2), 3.86-3.78 (m, 2H, H5a, H6a), 3.73 (d, 1H, H5b, ^2 ^*J =* 15.4), 2.87–2.80 (m, 1H, H6b), 2.73–2.66 (m, 1H, H7a), 2.48–2.42 (m, 5H, H7b, H8), 1.75 (m, 4H, H9). ^13 ^C NMR (100 MHz, CDCl_3_) δ (ppm): 171.4 (C4), 139.7, 129.2, 129.1, 127.1, 64.1(C2), 54.2, 53.2, 41.7, 33.1 (C5), 23.6. MS (70 eV): *m/z* (%) = 276 (M^+^,1%), 135 (1%), 121 (2%), 99 (5%), 84 (100%).

##### 2-(2-fluorophenyl)-3-(2-(pyrrolidin-1-yl)ethyl)thiazolidin-4-one (5 b)

Yield: 98%, oil, ^1^H NMR (200 MHz, CDCl_3_) δ (ppm, *J*_H-H_= Hz): 7.03–7.38 (m, 4H, Ar), 6.16 (d, 1H, H2, ^4 ^*J* = 1.6), 3.68 (d, 1H, H5a, ^2 ^*J =* 15.4), 3.77–3.94 (m, 2H, H5b, H6a), 2.70–2.87 (m, 2H, H6b, H7a), 2.47–2.60 (m, 5H, H7b, H8), 1.74–1.80 (m, 4H, H9). ^13 ^C NMR (50 MHz, CDCl_3_) δ (ppm): 171.1 (C4), 160.9 (d, ^1^*J_C-F_*= 248.7), 130.5 (d, ^3^*J_C-F_*= 8.5), 128.0 (d, ^4^*J_C-F_*= 2.8), 127.0 (d, ^2^*J_C-F_*= 11.1), 124.7 (d, ^4^*J_C-F_*= 3.5), 116.1 (d, ^2^*J_C-F_*= 21.5), 52.9, 57.4 (d, C2, ^3^*J_C-F_*= 4.1), 53.9, 41.5, 32.6 (C5), 23.4. MS (70 eV): *m/z* (%) = 294 (M^+^,1%), 97 (10%), 84 (100%), 70 (3%), 42 (10%).

##### 2-(3-fluorophenyl)-3-(2-(pyrrolidin-1-yl)ethyl)thiazolidin-4-one (5c)

Yield: 64%, oil, ^1^H NMR (200 MHz, CDCl_3_) δ (ppm, *J*_H-H_= Hz): 7.37 (dt, 1H, Ar, ^3 ^*J* = 8.6, ^4 ^*J* = 1.7), 7.09 (dt, 3H, Ar, ^4 ^*J* = 2.1, ^3 ^*J* = 7.9), 5.90 (s, 1H, H2), 3.73 (d, 1H, H5a, ^2 ^*J=* 15.5), 3.79–3.89 (m, 2H, H5b, H6a), 2.76 (dt, 1H, H6b, ^3 ^*J* = 6.7, ^2 ^*J=* 13.5), 2.66 (dt, 1H, H7a, ^3 ^*J* = 6.4, ^2 ^*J=* 12.8), 2.46–2.51 (m, 5H, H7b, H8), 1.76–1.78 (m, 4H, H9). ^13 ^C NMR (50 MHz, CDCl_3_) δ (ppm): 171.3 (C4), 163.1 (d, ^1^*J_C-F_*= 247.9), 142.6 (d, ^3^*J_C-F_*= 6.8), 130.7 (d, ^3^*J_C-F_*= 8.1), 122.6 (d, ^4^*J_C-F_*= 2.7), 116.1 (d, ^2^*J_C-F_*= 21.5), 113.9 (d, ^2^*J_C-F_*= 21.4), 63.3 (d, C2, ^4^*J_C-F_*= 1.5), 54.1, 53.3, 41.6, 32.8 (C5), 23.5. MS (70 eV): *m/z* (%) = 294 (M^+^,1%), 97 (7%), 84 (100%), 69 (3%), 42 (10%).

##### 2-(4-fluorophenyl)-3-(2-(pyrrolidin-1-yl)ethyl)thiazolidin-4-one (5d)

Yield: 74%, 56–58 °C, ^1^H NMR (200 MHz, CDCl_3_) δ (ppm, *J*_H-H_= Hz): 7.02–7.12 (m, 2H, Ar), 7.26–7.34 (m, 2H, Ar), 5.86 (s, 1H, H2), 3.71 (d, 1H, H5a, ^2 ^*J=* 15.5), 3.75–3.88 (m, 2H, H5b, H6a), 2.65–2.88 (m, 2H, H6b, H7a), 2.46–2.60 (m, 5H, H7b, H8), 1.74–1.80 (m, 4H, H9). ^13 ^C NMR (50 MHz, CDCl_3_) δ (ppm): 171.3 (C4), 162.9 (d, ^1^*J_C-F_*= 247.7),135.3 (d, ^4^*J_C-F_*= 3.2), 129.1 (d, ^3^*J_C-F_*= 8.5), 116.0 (d, ^2^*J_C-F_*= 21.8), 63.2 (C2), 54.0, 52.9, 41.2, 32.9 (C5), 23.4. MS (70 eV): *m/z* (%) = 294 (M^+^,1%), 109 (8%), 97 (6%), 84 (100%), 42 (17%).

##### 2-(2-chlorophenyl)-3-(2-(pyrrolidin-1-yl)ethyl)thiazolidin-4-one (5e)

Yield: 69%, oil, ^1^H NMR (200 MHz, CDCl_3_) δ (ppm, *J*_H-H_= Hz): 7.42 (dd, 1H, Ar, ^3 ^*J* = 7.3, ^4 ^*J* = 2.1), 7.31 (dt, 2H, Ar, ^3 ^*J* = 8.5, ^4 ^*J* = 1.8), 6.37 (s, 1H, H2), 3.68 (dd, 1H, H5a, ^4 ^*J* = 1.3, ^2 ^*J=* 15.6), 3.72 (d, 1H, H5b, ^2 ^*J=* 15.5), 3.99–4.00 (m, 1H, H6a), 2.78–2.92 (m, 2H, H6b, H7a), 2.56–2.61 (m, 5H, H7b, H8), 1.68–1.72 (m, 4H, H9). ^13 ^C NMR (50 MHz, CDCl_3_) δ (ppm): 172.0 (C4), 137.2, 132.8, 130.4, 129.7, 127.6, 60.1 (C2), 54.1, 53.2, 41.6, 32.3 (C5), 23,5. MS (70 eV): *m/z* (%) = 310 (M^+^, 1%), 97 (10%), 84 (100%), 55 (5%), 42 (9%).

##### 2-(3-chlorophenyl)-3-(2-(pyrrolidin-1-yl)ethyl)thiazolidin-4-one (5f)

Yield: 97%, oil, ^1^H NMR (200 MHz, CDCl_3_) δ (ppm, *J*_H-H_= Hz): 7.19–7.21 (m, 1H, Ar), 7.28–7.32 (m, 3H, Ar), 5.87 (s, 1H, H2), 3.72 (d, 1H, H5a, ^2 ^*J=* 16.1), 3.76–3.94 (m, 2H, H5b, H6a), 2.76–2.95 (m, 2H, H6b, H7a), 2.65–2.73 (m, 5H, H7b, H8), 1.80–1.84 (m, 4H, H9). ^13 ^C NMR (50 MHz, CDCl_3_) δ (ppm): 171.4 (C4), 141.8, 134.9, 130.3, 129.3, 127.1, 125.2, 63.0 (C2), 53.9, 52.6, 40.9, 32.7 (C5), 23.4. MS (70 eV): *m/z* (%) = 310 (M^+^, 1%), 97 (10%), 84 (100%), 70 (2%), 42 (9%).

##### 2-(4-chlorophenyl)-3-(2-(pyrrolidin-1-yl)ethyl)thiazolidin-4-one (5 g)

Yield: 69%, 83-86 °C, ^1^H NMR (200 MHz, CDCl_3_) δ (ppm, *J*_H-H_= Hz): 7.35 (d, 2H, Ar, ^3 ^*J* = 8.5), 7.25 (d, 2H, Ar, ^3 ^*J* = 8.5), 5.86 (s, 1H, H2), 3.88–3.73 (m, 3H, H5a, H5b, H6a), 2.86–2.63 (m, 2H, H6b, H7a), 2.47–2.41 (m, 5H, H7b, H8), 1.79–1.70 (m, 4H, H9). ^13 ^C NMR (50 MHz, CDCl_3_) δ (ppm): 171.1 (C4), 138.3, 134.8, 129.2, 128.4, 63.3 (C2), 54.1, 53.2, 41.5, 32.8 (C5), 23.5. MS (70 eV): *m/z* (%) = 310 (M^+^, 1%), 97 (6%), 84 (100%), 69 (2%), 55 (5%), 42 (9%).

##### 2-(2-nitrophenyl)-3-(2-(pyrrolidin-1-yl)ethyl)thiazolidin-4-one (5 h)

Yield: 89%, 71–73 °C, ^1^H NMR (200 MHz, CDCl_3_) δ (ppm, *J*_H-H_= Hz): 8.09 (dd, 1H, Ar, ^3 ^*J* = 8.1, ^4 ^*J* = 1.3), 7.69 (td, 1H, Ar, ^3 ^*J* = 7.5, ^4 ^*J* = 1.2), 7.51 (td, Ar, 1H, ^3 ^*J* = 7.5, ^4 ^*J* = 1.4), 7.30 (dd, Ar, 1H, ^3 ^*J* = 7.8, ^4 ^*J* = 1.6), 6.54 (d, 1H, H2, ^4 ^*J* = 1.4), 3.77 (dd, 1H, H5a, ^2 ^*J=* 15.6, ^4 ^*J* = 1.5), 3.62 (d, 1H, H5b, ^2 ^*J=* 15.6), 3.93–4.02 (m, 1H, H6a), 2.77–2.93 (m, 2H, H6b, H7a), 2.39–2.57 (m, 5H, H7b, H8), 1.67–1.73 (m, 4H, H9). ^13 ^C NMR (50 MHz, CDCl_3_) δ (ppm): 172.3 (C4), 147.1, 136.8, 134.3, 129.0, 125.9, 125.6, 58.8 (C2), 53.9, 53.4, 41.6, 31.6 (C5), 23.3. MS (70 eV): *m/z* (%) = 291 (M-30, 2%), 97 (4%), 84 (100%), 55 (5%), 42 (11%).

##### 2-(3-nitrophenyl)-3-(2-(pyrrolidin-1-yl)ethyl)thiazolidin-4-one (5i)

Yield: 73%, 98–101 °C, ^1^H NMR (200 MHz, CDCl_3_) δ (ppm, *J*_H-H_= Hz): 8.24–8.17 (m, 2H, Ar), 7.68–7.55 (m, 2H, Ar), 6.03 (s,1H, H2), 3.94–3.81 (m, 2H, H5a, H6a) 3.74 (d, 1H, H5b, ^2 ^*J* = 15.5), 2.84–2.67 (m, 2H, H6b, H7a), 2.50–2.38 (m, 5H, H7b, H8), 1.70–1.80 (m, 4H, H9). ^13 ^C NMR (50 MHz, CDCl_3_) δ (ppm): 171.1 (C4), 148.5, 142.5, 132.8, 130.1, 123.9, 122.1, 62.9 (C2), 54.1, 53.6, 41.7, 32.7 (C5), 23.5. MS (70 eV): *m/z* (%) = 321 (M^+^, 1%), 97 (4%), 84 (100%), 55 (5%), 42 (10%).

##### 2-(4-nitrophenyl)-3-(2-(pyrrolidin-1-yl)ethyl)thiazolidin-4-one (5j)

Yield: 81%, oil, ^1^H NMR (200 MHz, CDCl_3_) δ (ppm, *J*_H-H_= Hz): 8.24 (d, 2H, Ar ^3 ^*J* = 8.7), 7.47 (d, 2H, Ar, ^3 ^*J* = 8.7), 6.03 (s,1H, H2), 3.73 (d, 1H, H5a, ^2 ^*J* = 15.5), 3.78–3.96 (m, 2H, H5b, H6a), 2.70–2.87 (m, 2H, H6b, H7a), 2.47–2.60 (m, 5H, H7b, H8), 1.74–1.80 (m, 4H, H9). ^13 ^C NMR (50 MHz, CDCl_3_) δ (ppm): 171.3 (C4), 148.1, 147.3, 127.7, 124.3, 62.7 (C2), 54.0, 53.3, 41.6, 32.6 (C5), 23.4. MS (70 eV): *m/z* (%) = 207 (M^+^-114,1%), 97 (4%), 84 (100%), 55 (6%), 42 (16%).

##### 3-(2-(pyrrolidin-1-yl)ethyl)-2-p-tolylthiazolidin-4-one (5k)

Yield: 78%, 57-60 °C, ^1^H NMR (200 MHz, CDCl_3_) δ (ppm, *J*_H-H_= Hz): 7.18 (s, 4H, Ar), 5.83 (s, 1H, H2), 3.79 (dd, 1H, H5a, ^4 ^*J* = 1.9, ^2 ^*J=* 14.4), 3.69 (d, 1H, H5b, ^2 ^*J=* 15.4), 3.80–3.87 (m, 1H, H6a), 2.61–2.88 (m, 2H, H6b, H7a), 2.40–2.48 (m, 5H, H7b, H8), 1.70–1.77 (m, 4H, H9). ^13 ^C NMR (50 MHz, CDCl_3_) δ (ppm): 171.2 (C4), 139.0, 136.6, 129.7, 126.9, 63.8 (C2), 54.1, 53.2, 41.5, 32.9 (C5), 23.5, 21.2 (CH_3_). MS (70 eV): *m/z* (%) = 290 (M^+^, 1%), 97 (11%), 84 (100%), 70 (5%), 55 (4%), 42 (9%).

##### 2-(2-methoxyphenyl)-3-(2-(pyrrolidin-1-yl)ethyl)thiazolidin-4-one (5 l)

Yield: 71%, oil, ^1^H NMR (200 MHz, CDCl_3_) δ (ppm, *J*_H-H_= Hz): 7.26–7.34 (m, 1H, Ar), 7.12 (dd, 1H, Ar, ^3 ^*J* = 7.5, ^4 ^*J* = 1.7), 6.89–6.99 (m, 2H, Ar), 6.16 (d, 1H, H2, ^4 ^*J* = 1.5), 3.74 (dd, 1H, H5a, ^4 ^*J* = 1.6, ^2 ^*J=* 15.3), 3.61 (d, 1H, H5b, ^2 ^*J=* 15.3), 3.81–3.96 (m, 4H, H6a, OCH_3_), 3.86 (s, 3H, OCH_3_), 2.67–2.92 (m, 2H, H6b, H7a), 2.45–2.57 (m, 5H, H7b, H8), 1.71–1.89 (m, 4H, H9). ^13 ^C NMR (50 MHz, CDCl_3_) δ (ppm): 172.0 (C4), 156.9, 129.8, 127.7, 126.7, 120.0, 111.0, 58.3 (C2), 55.5 (OCH_3_), 54.0, 52.9, 41.7, 32.6 (C5), 23.4. MS (70 eV): *m/z* (%) = 306 (M^+^, 2%), 97 (18%), 84 (100%), 70 (5%), 42 (9%).

##### 2-(3-methoxyphenyl)-3-(2-(pyrrolidin-1-yl)ethyl)thiazolidin-4-one (5 m)

Yield: 94%, oil, ^1^H NMR (200 MHz, CDCl_3_) δ (ppm, *J*_H-H_= Hz): 7.27 (t, 2H, Ar, ^3 ^*J* = 7.6), 6.84-6.89 (m, 2H, Ar), 5.84 (s, 1H, H2), 3.74–3.92 (m, 6H, H5a, H5b, H6a, OCH_3_), 3.79 (s, 3H, OCH_3_), 2.77–2.99 (m, 2H, H6b, H7a), 2.64–2.74 (m, 5H, H7b, H8), 1.78–1.85 (m, 4H, H9). ^13 ^C NMR (50 MHz, CDCl_3_) δ (ppm): 171.6 (C4), 160.1, 141.0, 130.0, 119.2, 114.6, 112.5, 63.7 (C2), 55.3 (OCH_3_), 53.9, 52.5, 40.9, 32.8 (C5), 23.3. MS (70 eV): *m/z* (%) = 306 (M^+^, 2%), 97 (12%), 84 (100%), 70 (4%), 55 (5%), 42 (8%).

##### 2-(4-methoxyphenyl)-3-(2-(pyrrolidin-1-yl)ethyl)thiazolidin-4-one (5n)

Yield: 83%, 60-63 °C, ^1^H NMR (200 MHz, CDCl_3_) δ (ppm, *J*_H-H_= Hz): 7.28–7.22 (m, 2H, Ar), 6.91–6.87 (m, 2H, Ar), 5.81 (s, 1H, H2), 3.91–3.81 (m, 4H, H6a, OCH_3_), 3.74–3.78 (m, 2H, H5a, H5b), 2.83 (dt, 1H, H6b, ^3 ^*J* = 6.7, ^2 ^*J=* 13.4), 2.67 (dt, 1H, H7a, ^3 ^*J* = 6.6, ^2 ^*J=* 13.2), 2.64–2.74 (m, 5H, H7b, H8), 1.78–1.85 (m, 4H, H9). ^13 ^C NMR (50 MHz, CDCl_3_) δ (ppm): 171.2 (C4), 160.1, 131.2, 128.6, 114.3, 63.7 (C2), 55.3 (OCH_3_), 54.0, 53.0, 41.4, 33.0 (C5), 23.4. MS (70 eV): *m/z* (%) = 306 (M^+^, 1%), 97 (11%), 84 (100%), 70 (6%), 42 (8%).

##### 2-(3-hydroxyphenyl)-3-(2-(pyrrolidin-1-yl)ethyl)thiazolidin-4-one (5o)

Yield: 85%, oil, ^1^H NMR (200 MHz, CDCl_3_) δ (ppm, *J*_H-H_= Hz): 7.17 (t, 1H, Ar ^3 ^*J* = 7.6), 6.68–6.75 (m, 3H, Ar), 5.68 (s, 1H, H2), 5.57 (s, 1H, OH), 3.88–3.72 (m, 2H, H6a, H5a), 3.64 (d, 1H, H5b, ^2 ^*J=* 15.4), 2.63–2.96 (m, 2H, H6b, H7a), 2.45–2.61 (m, 5H, H7b, H8), 1.71–1.80 (m, 4H, H9). ^13 ^C NMR (50 MHz, CDCl_3_) δ (ppm): 171.8 (C4), 157.7, 140.7, 130.2, 118.1, 110.5, 113.7, 63.9 (C2), 54.0, 52.5, 41.5, 32.9 (C5), 23.3. MS (70 eV): *m/z* (%) = 292 (M^+^, 1%), 97 (9%), 84 (100%), 55 (5%), 42 (10%).

##### 2-(2,3-dimethoxyphenyl)-3-(2-(pyrrolidin-1-yl)ethyl)thiazolidin-4-one (5p)

Yield: 70%, oil, ^1^H NMR (200 MHz, CDCl_3_) δ (ppm, *J*_H-H_= Hz): 7.06 (t, 1H, Ar, ^3 ^*J* = 7.9), 6.75 (d, 1H, Ar, ^3 ^*J* = 7.7), 6.90 (d, 1H, Ar, ^3 ^*J* = 8.1), 6.18 (s, 1H, H2), 3.87 (s, 3H, OCH_3_), 3.89 (s, 3H, OCH_3_), 3.78 (dd, 1H, H5a, ^2 ^*J=* 16.1, ^4 ^*J* = 1.0), 3.65 (d, 1H, H5b, ^2 ^*J=* 15.3), 3.91–3.99 (m, 1H, H6a), 2.58–2.90 (m, 2H, H6b, H7a), 2.50–2.58 (m, 5H, H7b, H8), 1.72–1.78 (m, 4H, H9). ^13 ^C NMR (50 MHz, CDCl_3_) δ (ppm): 171.7 (C4), 152.8, 146.9, 133.4, 124.3, 118.3, 112.7, 61.1 (C2), 57.8 (OCH_3_), 55.8 (OCH_3_), 54.0, 52.9, 41.7, 32.6 (C5), 23.4, MS (70 eV): *m/z* (%) = 336 (M^+^, 2%), 97 (18%), 84 (100%), 70 (6%), 55 (4%), 42 (8%).

##### 2-(2,4-dichlorophenyl)-3-(2-(pyrrolidin-1-yl)ethyl)thiazolidin-4-one (5q)

Yield: 94%, 82-85 °C, ^1^H NMR (200 MHz, CDCl_3_) δ (ppm, *J*_H-H_= Hz): 7.35 (s, 1H, Ar), 7.22 (d, 1H, Ar, ^3 ^*J* = 8.5), 7.06 (d, 1H, Ar, ^3 ^*J* = 8.4), 6.24 (s, 1H, H2), 3.62 (s, 1H, H5a), 3.58 (d, 1H, H5b, ^2 ^*J=* 15.6), 3.81–3.92 (m, 1H, H6a), 2.70–2.79 (m, 2H, H6b, H7a), 2.49–2.59 (m, 5H, H7b, H8), 1.69–1.75 (m, 4H, H9). ^13 ^C NMR (50 MHz, CDCl_3_) δ (ppm): 171.9 (C4), 135.0, 134.9, 133.5, 130.3, 127.9, 128.8, 59.7 (C2), 53.8, 52.9, 41.4, 32.1 (C5), 23.4. MS (70 eV): *m/z* (%) = 344 (M^+^-1, 1%), 97 (7%), 84 (100%),55 (5%), 42 (13%).

##### 2-(2,6-dichlorophenyl)-3-(2-(pyrrolidin-1-yl)ethyl)thiazolidin-4-one (5r)

Yield: 84%, 106–109 °C, ^1^H NMR (200 MHz, CDCl_3_) δ (ppm, *J*_H-H_= Hz): 7.14–7.35 (m, 3H, Ar), 6.80 (d, 1H, H2, ^4 ^*J* = 1.9), 3.74 (d, 1H, H5a, ^2 ^*J=* 15.0), 3.80–3.95 (m, 2H, H6a, H5b), 2.59–2.75 (m, 2H, H6b, H7a), 2.42–2.49 (m, 5H, H7b, H8), 1.70–1.76 (m, 4H, H9). ^13 ^C NMR (50 MHz, CDCl_3_) δ (ppm): 171.4 (C4), 135.5, 135.1, 132.6, 130.9, 130.0, 128.8, 58.9 (C2), 54.0, 53.0, 41.7, 34.1 (C5), 23.5.

##### 2-(2-chloro-6-fluorophenyl)-3-(2-(pyrrolidin-1-yl)ethyl)thiazolidin-4-one (5 s)

Yield: 83%, 65-68 °C, ^1^H NMR (600 MHz, CDCl_3_) δ (ppm, *J*_H-H_= Hz): 7.30–7.19 (m, 2H, Ar), 7.07–6.99 (m, 1H, Ar), 6.48 (s, 1H, H2), 3.90–3.81 (m, 1H, H6a, H5a), 3.80–3.67 (m, 1H, H5b), 2.89–2.84 (m, 1H, H6b), 2.80–2.74 (m, 1H, H7a), 2.61–2.54 (m, 5H, H7b, H8), 1.79–1.77 (m, 4H, H9). ^13 ^C NMR (150 MHz, CDCl_3_) δ (ppm): 171.5 (C4), 161.9 (d, ^1^*J_C-F_*= 253.9),134.1 (d, ^4^*J_C-F_*= 5.5), 130.5 (d, ^3^*J_C-F_*= 10.4.5), 127.8 (d, ^4^*J_C-F_*= 3.3), 125.8 (d, ^4^*J_C-F_*= 3.3), 115,8 (d, ^2^*J_C-F_*= 22.4), 57.5 (C2), 54.1, 53.1, 41.7, 33.2 (C5), 23.5. MS (70 eV): *m/z* (%) = 328 (M^+^, 2%), 97 (15%), 84 (100%), 55 (5%), 42 (13%).

##### 2-(2-hydroxyphenyl)-3-(2-(pyrrolidin-1-yl)ethyl)thiazolidin-4-one (5t)

Yield: 71%, oil, ^1^H NMR (600 MHz, CDCl_3_) δ (ppm, *J*_H-H_= Hz): 7.24 (dd, 1H, Ar, ^3 ^*J* = 7.7, ^4 ^*J* = 1.4), 7.17 (td, 1H, Ar, ^3 ^*J* = 8.0, ^4 ^*J* = 1.6), 6.89 (t, 1H, Ar, ^3 ^*J* = 7.2), 6.83 (d, 1H, Ar, ^3 ^*J* = 8.1), 6.16 (s, 1H, H2), 3.83 (dt, 1H, H6a, ^2 ^*J* = 14.4, ^3 ^*J* = 6.1, ^3 ^*J* = 5.7), 3,80 (dd, 1H, H5a, ^2 ^*J* = 15.6, ^4 ^*J* = 1.6), 3.64 (d, 1H, H5b, *J* = 15.4), 2.96 (dt, 1H, H6b, ^2 ^*J* = 14.3, ^3 ^*J* = 7,2, ^3 ^*J* = 6.3), 2.80 (dt, 1H, H7a, ^2 ^*J* = 12.8, ^3 ^*J* = 6.7, ^3 ^*J* = 6.0), 2.68–2.65 (m, 5H, H7b, H8), 1.85–1.83 (m, H9). ^13 ^C NMR (150 MHz, CDCl_3_) δ (ppm): 171.9 (C4), 155.7, 130.4, 128.4, 126.0, 120.7, 118.1, 59,1 (C2), 54.5, 54.0, 41.4, 33.3 (C5), 23.3. MS (70 eV): *m/z* (%) = 292 (M^+^, 3%), 97 (14%), 84 (100%), 55 (6%), 42 (11%).

##### 2-(2,4-dimethoxyphenyl)-3-(2-(pyrrolidin-1-yl)ethyl)thiazolidin-4-one (5 u)

Yield: 80%, oil, ^1^H NMR (200 MHz, CDCl_3_) δ (ppm, *J*_H-H_= Hz): 7.08 (d,1H, Ar, ^3 ^*J* = 8.1), 6.49–6.45 (m, 2H, Ar), 6.10 (s, 1H, H2), 3.87–3.82 (m, 4H, H6a, OCH_3_), 3.81(s, 3H, OCH_3_), 3.73 (dd, 1H, H5a, ^2 ^*J* = 15.3, ^4 ^*J* = 1.8), 3.63 (d, 1H, H5b, ^2 ^*J* = 15.3), 2.91 (dt, 1H, H6b, ^2 ^*J* = 13.8, ^3 ^*J* = 6.8), 2.79–2.71 (m, 1H, H7a), 2.61–2.52 (m, 5H, H7b, H8), 1.78 (t, 4H, H9, ^3 ^*J* = 6.0). ^13 ^C NMR (150 MHz, CDCl_3_) δ (ppm): 172.0 (C4), 161.4, 158.2, 128.3, 119.8, 104.7, 99.0, 58.6 (C2), 55.7, 55.5, 54.1, 52.9, 41.5, 32.9 (C5), 23.5. MS (70 eV): *m/z* (%) = 336 (M^+^, 4%), 97 (19%), 84 (100%), 70 (6%), 55 (4%), 42 (10%).
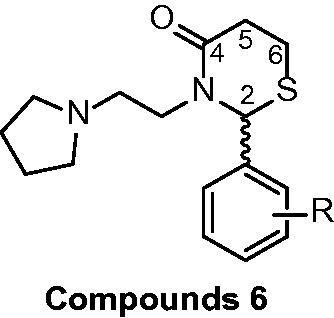


##### 2-phenyl-3-(2-(pyrrolidin-1-yl)ethyl)-1,3-thiazinan-4-one (6a)

Yield: 92%, oil, ^1^H NMR (600 MHz, CDCl_3_) δ (ppm, *J*_H-H_= Hz): 7,36 (t, 2H, Ar, ^3 ^*J* = 7.5), 7.31–7.29 (m, 1H, Ar), 7.23 (d, 2H, Ar, ^3 ^*J* = 7.5), 5.80 (s, 1H, H2), 4.22–4.17 (m, 1H, H7a), 2.90–2.81 (m, 4H, H5, H6), 2.80–2.77 (m, 1H, H7b), 2.68–2.62 (m, 2H, H8), 2.60–2.58 (m, 4H, H9), 1.80–1.75 (m, 4H, H10). ^13 ^C NMR (150 MHz, CDCl_3_) δ (ppm): 169.4 (C4), 139.3, 128.5, 128.0, 126.4, 62.4 (C2), 54.0, 53.4, 46.4, 34.4 (C5), 23.4, 21.6 (C6). MS (70 eV): *m/z* (%) = 290 (M^+^, 1%), 193 (1%), 121 (3%), 97 (32%), 84 (100%), 70 (5%) 55 (6%), 42 (11%).

##### 2-(2-fluorophenyl)-3-(2-(pyrrolidin-1-yl)ethyl)-1,3-thiazinan-4-one (6 b)

Yield: 82%, oil, ^1^H NMR (600 MHz, CDCl_3_) δ (ppm, *J*_H-H_= Hz): 7.34–7.30 (m, 1H, Ar), 7.14 (t, 1H, Ar, ^3 ^*J* = 7.5), 7.10 (t, 1H, Ar, ^3 ^*J* = 9.3), 7.06 (td, 1H, Ar, ^3 ^*J* = 7.6, ^4 ^*J* = 1.2), 6.07 (s, 1H, H2), 4.12–4.07 (m, 1H, H7a), 2.88–2.81 (m, 4H, H5, H6), 2.80–2.76 (m, 1H, H7b), 2.67–2.59 (m, 2H, H8), 2.54 (m, 4H, H9), 1.78–1.74 (m, 4H, H10). ^13 ^C NMR (150 MHz, CDCl_3_) δ (ppm): 169.3 (C4), 159.5 (d, ^1^*J*_C-F_= 248.7), 129.7 (d, ^3^*J*_C-F_= 8.3), 126.8 (d, ^2^*J*_C-F_= 11.9), 126.6 (d, ^4^*J*_C-F_= 2.6), 123.6 (d, ^4^*J*_C-F_= 3.5), 116.1 (d, ^2^*J*_C-F_= 21.9), 55.9 (d, C2, ^4^*J*_C-F_= 4.1), 54.0, 53.5, 46.2, 34.4 (C5), 23.4, 21.4 (C6). MS (70 eV): *m/z* (%) = 308 (M^+^, 1%), 304 (1%), 139 (2%), 124 (1%), 109 (5%), 97 (25%), 84 (100%), 70 (3%) 56 (6%), 42 (11%).

##### 2-(3-fluorophenyl)-3-(2-(pyrrolidin-1-yl)ethyl)-1,3-thiazinan-4-one (6c)

Yield: 81%, oil, ^1^H NMR (600 MHz, CDCl_3_) δ (ppm, *J*_H-H_= Hz): 7.33 (td, 1H, Ar, ^3 ^*J* = 5.8, ^4 ^*J* = 2.1), 7.03–6.96 (m, 3H, Ar), 5.83 (s, 1H, H2), 4.23–4.17 (m, 1H, H7a), 2.91–2.84 (m, 2H, H5a, H6a), 2.82–2.77 (m, 3H, H5b, H6b, H7b), 2.68–2.63 (m, 2H, H8), 2.61-2.58 (m, 4H, H9), 1.82–1.75 (m, 4H, H10). ^13 ^C NMR (150 MHz, CDCl_3_) δ (ppm): 169.2 (C4), 162.7 (d, ^1^*J*_C-F_= 247.5), 142.3 (d, ^3^*J*_C-F_= 6.4), 130.0 (d, ^3^*J*_C-F_= 8.2), 122.0 (d, ^4^*J*_C-F_= 2.7), 115.0 (d, ^2^*J*_C-F_= 21.1), 113.5 (d, ^2^*J*_C-F_= 22.7), 61.9 (d, C2, ^4^*J*_C-F_= 1.7), 54.0, 53.5, 46.4, 34.3 (C5), 23.4, 21.4 (C6). MS (70 eV): *m/z* (%) = 308 (M^+^, 1%), 304 (1%), 139 (3%), 124 (2%), 109 (7%), 97 (35%), 84 (100%), 70 (4%) 56 (7%), 42 (11%).

##### 2-(4-fluorophenyl)-3-(2-(pyrrolidin-1-yl)ethyl)-1,3-thiazinan-4-one (6d)

Yield: 78%, oil, ^1^H NMR (600 MHz, CDCl_3_) δ (ppm, *J*_H-H_= Hz): 7.23–7.21 (m, 2H, Ar), 7.07–7.04 (m, 2H, Ar), 5.80 (s, 1H, H2), 4.21–4.15 (m, 1H, H7a), 2.87–2.80 (m, 4H, H5, H6), 2.79–2.74 (m, 1H, H7b), 2.67–2.61 (m, 2H, H8), 2.56 (sl, 4H, H9), 1.79–1.74 (m, 4H, H10). ^13 ^C NMR (150 MHz, CDCl_3_) δ (ppm): 169.2 (C4), 162.2 (d, ^1^*J*_C-F_= 247.6), 135.2 (d, ^4^*J*_C-F_= 2.8), 128.1 (d, ^3^*J*_C-F_= 8.2), 115.4 (d, ^2^*J*_C-F_= 21.6), 61.9 (C2), 54.1, 53.5, 46.4, 34.4 (C5), 23.4, 21.6 (C6). MS (70 eV): *m/z* (%) = 308 (M^+^, 1%), 304 (1%), 139 (3%), 122 (2%), 109 (7%), 97 (24%), 84 (100%), 70 (3%) 55 (6%), 42 (11%).

##### 2-(2-chlorophenyl)-3-(2-(pyrrolidin-1-yl)ethyl)-1,3-thiazinan-4-one (6e)

Yield: 79%, oil, ^1^H NMR (600 MHz, CDCl_3_) δ (ppm, *J*_H-H_= Hz): 7.45–7.43 (m, 1H, Ar), 7.30–7.28 (m, 2H, Ar), 7.09–7.06 (m, 1H, Ar), 6.18 (d, 1H, H2, ^4 ^*J* = 0.9), 4.19 (ddd, 1H, H7a, ^3 ^*J* = 4.8, ^3 ^*J* = 6.9, ^2 ^*J* = 13.5), 2.90–2.83 (m, 3H, H5, H6a), 2.81–2.76 (m, 1H, H7b), 2.68–2.62 (dt, 1H, H6b, ^3 ^*J* = 5.7, ^2 ^*J* = 11.2), 2.61–2.57 (m, 1H, H8a), 2.56–2.52 (m, 1H, H8b), 2.51–2.46 (m, 4H, H9), 1.75 (sl, 4H, H10). ^13 ^C NMR (150 MHz, CDCl_3_) δ (ppm): 169.4 (C4), 136.4, 132.8, 130.8, 129.3, 126.6, 126.5, 59.3 (C2), 54.2, 54.1, 46.2, 34.6 (C5), 23.6, 21.1 (C6). MS (70 eV): *m/z* (%) = 324 (M^+^, 1%); 320 (1%); 152 (2%); 139 (2%); 125 (5%); 97 (36%); 84 (100%); 70 (4%); 55 (7%); 42 (11%).

##### 2-(3-chlorophenyl)-3-(2-(pyrrolidin-1-yl)ethyl)-1,3-thiazinan-4-one (6f)

Yield: 87%, 69–71 °C, ^1^H NMR (600 MHz, CDCl_3_) δ (ppm, *J*_H-H_= Hz): 7.31–7.27 (m, 2H, Ar), 7.24 (s, 1H, Ar), 7.13 (d, 1H, Ar, ^3 ^*J* = 7.0), 5.80 (s, 1H, H2), 4.22–4.16 (m, 1H, H7a), 2.88–2.80 (m, 4H, H5, H6), 2.79–2.76 (m, 1H, H7b), 2.66–2.61 (m, 2H, H8), 2.58–2.55 (m, 4H, H9), 1.79–1.74 (m, 4H, H10). ^13 ^C NMR (150 MHz, CDCl_3_) δ (ppm): 169.4 (C4), 141.8, 134.6, 129.7, 128.1, 126.5, 124.5, 61.9 (C2), 54.1, 53.6, 46.5, 34.3 (C5), 23.4, 21.6 (C6). MS (70 eV): *m/z* (%) = 324 (M^+^, 1%), 152 (1%), 139 (1%), 125 (3%), 97 (25%), 84 (100%), 70 (3%) 56 (3%), 42 (10%).

##### 2-(4-chlorophenyl)-3-(2-(pyrrolidin-1-yl)ethyl)-1,3-thiazinan-4-one (6 g)

Yield: 59%, oil, ^1^H NMR (600 MHz, CDCl_3_) δ (ppm, *J*_H-H_= Hz): 7.34 (d, 2H, Ar, ^3 ^*J* = 8.4), 7.18 (d, 2H, Ar, ^3 ^*J* = 8.4), 5.81 (s, 1H, H2), 4.21–4.16 (m, 1H, H17a), 2.89–2.80 (m, 4H, H5, H6), 2.78–2.73 (m, 1H, H7b), 2.66–2.62 (m, 2H, H8), 2.59–2.57 (m, 4H, H9), 1.80–1.75 (m, 4H, H10). ^13 ^C NMR (150 MHz, CDCl_3_) δ (ppm): 169.2 (C4), 138.1, 133.8, 128.7, 127.7, 61.9 (C2), 54.1, 53.5, 46.4, 34.4 (C5), 23.4, 21.6 (C6). MS (70 eV): *m/z* (%) = 324 (M^+^, 1%), 320 (1%), 152 (1%), 139 (1%), 125 (4%), 97 (23%), 84 (100%), 70 (3%) 56 (3%), 42 (10%).

##### 2-(2-nitrophenyl)-3-(2-(pyrrolidin-1-yl)ethyl)-1,3-thiazinan-4-one (6 h)

Yield: 81%, oil, ^1^H NMR (600 MHz, CDCl_3_) δ (ppm, *J*_H-H_= Hz): 8.09 (d, 1H, Ar ^3 ^*J* = 8.1), 7.61 (t, 1H, Ar, ^3 ^*J* = 7.5), 7.48 (t, 1H, Ar, ^3 ^*J* = 7.7), 7.23 (d, 1H, Ar, ^3 ^*J* = 7.8), 6.81 (s, 1H, H2), 4.28–4.22 (m, 1H, H7a), 3.03–2.98 (m, 1H, H6a), 2.88 (dd, 1H, H5a, ^2 ^*J* = 13.7, ^3 ^*J* = 7.0), 2.86–2.81 (m, 2H, H15b, H16b), 2.76–2.71 (m, 1H, H7b), 2.68–2.65 (m, 4H, H9), 2.64–2.60 (m, 1H, H8a), 2.61–2.57 (m, 1H, H8b), 1.80 (sl, 4H, H10). ^13 ^C NMR (150 MHz, CDCl_3_) δ (ppm): 169.7, 147.3, 135.5, 132.8, 129.0, 127.2, 126.8, 58.3 (C2), 54.1, 53.5, 46.4, 34.4 (C5), 23.6, 21.7 (C6). MS (70 eV): *m/z* (%) = 305 (M^+^-20, 1%), 234 (1%), 152 (1%), 130 (1%), 119 (1%), 97 (14%), 84 (100%), 70 (2%) 56 (3%), 42 (10%).

##### 2-(3-nitrophenyl)-3-(2-(pyrrolidin-1-yl)ethyl)-1,3-thiazinan-4-one (6i)

Yield: 94%, 56-58 °C, ^1^H NMR (600 MHz, CDCl_3_) δ (ppm, *J*_H-H_= Hz): 8.19–8.13 (m, 1H, Ar), 7.60 (d, 1H, Ar, ^3 ^*J* = 7.7), 7.56 (t, 1H, Ar, ^3 ^*J* = 7.8), 5.95 (s, 1H, H2), 4.21–4.16 (m, 1H, H7a), 2.92–2.79 (m, 4H, H5, H6), 2.78–2.72 (m, 1H, H7b), 2.72–2.67 (m, 1H, H8a), 2.62–2.57 (m, 1H, H8b), 2.50 (dd, 4H, H9, ^3 ^*J* = 9.5, ^3 ^*J* = 7.0), 1.73 (t, 4H, H10, ^3 ^*J* = 6.3). ^13 ^C NMR (150 MHz, CDCl_3_) δ (ppm): 169.1, 148.7, 142.8, 132.2, 129.6, 123.1, 121.7, 62.0 (C2), 54.3, 54.1, 46.8, 34.5 (C5), 23.7, 22.0 (C6). MS (70 eV): *m/z* (%) = 335 (M^+^, 1%), 265 (1%), 163 (1%), 130 (1%), 117 (1%), 97 (15%), 84 (100%), 70 (2%) 56 (4%), 42 (10%).

##### 2-(4-nitrophenyl)-3-(2-(pyrrolidin-1-yl)ethyl)-1,3-thiazinan-4-one (6j)

Yield: 89%, 88–90 °C, ^1^H NMR (600 MHz, CDCl_3_) δ (ppm, *J*_H-H_= Hz): 8.23 (d, 2H, Ar, ^3 ^*J* = 8.7), 7.45 (d, 2H, Ar, ^3 ^*J* = 8.7), 6.03 (s, 1H, H2), 4.24–4.20 (m, 1H, H17a), 2.96–2.83 (m, 5H, H5, H6, H7b), 2.76–2.66 (m, 2H, H8), 2.62 (m, 4H, H9), 1.82–1.79 (m, 4H, H10). ^13 ^C NMR (150 MHz, CDCl_3_) δ (ppm): 169.0 (C4), 147.4, 147.1, 127.2, 123.7, 61.7 (C2), 54.1, 53.6, 46.3, 34.3 (C5), 23.4, 21.5 (C6). MS (70 eV): *m/z* (%) = 335 (M^+^, 1%), 163 (1%), 133 (1%), 117 (1%), 97 (13%), 84 (100%), 70 (2%) 56 (3%), 42 (10%).

##### 3-(2-(pyrrolidin-1-yl)ethyl)-2-(p-tolyl)-1,3-thiazinan-4-one (6k)

Yield: 83%, oil, ^1^H NMR (600 MHz, CDCl_3_) δ (ppm, *J*_H-H_= Hz): 7.17 (d, 2H, Ar, ^3 ^*J* = 7.9), 7.11 (d, 2H, Ar, ^3 ^*J* = 8.1), 5.81 (s, 1H, H2), 4.19–4.14 (m, 1H, H7a), 3.00–2.95 (m, 2H, H5a, H6a), 2.83–2.74 (m, 7H, H5b, H6b, H7b, CH_3_, H8a), 2.65–2.62 (m, 1H, H8b), 2.35–2.33 (m, 4H, H9), 1.85–1.83 (m, 4H, H10). ^13 ^C NMR (150 MHz, CDCl_3_) δ (ppm): 169.7 (C4), 138.0, 136.1, 129.2, 126.3, 62.5 (C2), 54.0, 53.1, 46.0, 34.5 (C5), 23.4, 21.6 (C6), 21,0. MS (70 eV): *m/z* (%) = 305 (M^+^,1%), 271 (1%), 174 (1%), 146 (1%), 135 (2%), 119 (1%), 105 (7%), 97 (35%), 84 (100%), 70 (6%), 55 (6%), 42 (10%).

##### 2-(2-methoxyphenyl)-3-(2-(pyrrolidin-1-yl)ethyl)-1,3-thiazinan-4-one (6 l)

Yield: 85%, oil, ^1^H NMR (600 MHz, CDCl_3_) δ (ppm, *J*_H-H_= Hz): 7.28 (t, 1H, Ar, ^3 ^*J* = 7.9, ^4 ^*J* = 3.3) , 6.84 (dd, 1H, Ar, ^3 ^*J* = 8.2, ^4 ^*J* = 2.3), 6.80 (d, 1H, Ar, ^3 ^*J* = 7.6), 6.77 (m, 1H, Ar), 5.77 (s, 1H, H2), 4.21–4.17 (m, 1H, H7a), 3,80 (s.3H, OCH_3_), 2.89–2.80 (m, 5H, H5, H6, H7b), 2.69–2.62 (m, 2H, H8), 2.61–2.59 (m, 4H, H9), 1.79–1.76 (m, 4H, H10). ^13 ^C NMR (150 MHz, CDCl_3_) δ (ppm): 169.3 (C4), 159.7, 141.0, 129.5, 118.7, 113.2, 112.4, 62.4 (C2), 55.2, 54.1, 53.4, 46.4, 34.4 (C5), 23.4, 21.7 (C6). MS (70 eV): *m/z* (%) = 320 (M^+^, 1%), 223 (1%), 148 (2%), 135 (2%), 121 (3%), 97 (35%), 84 (100%), 70 (5%) 56 (3%), 42 (10%).

##### 2-(3-methoxyphenyl)-3-(2-(pyrrolidin-1-yl)ethyl)-1,3-thiazinan-4-one (6 m)

Yield: 63%, oil, ^1^H NMR (600 MHz, CDCl_3_) δ (ppm, *J*_H-H_= Hz): 7.30–7.25 (m, 1H, Ar), 6.84 (d, 1H, Ar, ^3 ^*J* = 7.8), 6.80 (d, 1H, Ar, ^3 ^*J* = 7.1), 6.77 (s,1H, Ar), 5.77 (s, 1H, H2), 4.19 (dd, 1H, H7a, ^2 ^*J* = 12.4, ^3 ^*J* = 6.6), 3.80 (s, 3H, OCH_3_), 2.97–2.80 (m, 5H, H5, H6, H7b), 2.75–2.61 (m, 6H, H8, H9), 1.81 (sl, 4H, H10). ^13 ^C NMR (150 MHz, CDCl_3_) δ (ppm): 169.6 (C4), 159.9, 141.2, 129.7, 118.9, 113.4, 112.6, 62.5 (C2), 55.4, 54.0, 53.2, 46.3, 34.6 (C5), 23.5, 21.9 (C6). MS (70 eV): *m/z* (%) = 320 (M^+^, 1%), 151 (1%), 136 (4%), 121 (2%), 97 (33%), 84 (100%), 70 (4%) 56 (5%), 42 (11%).

##### 2-(4-methoxyphenyl)-3-(2-(pyrrolidin-1-yl)ethyl)-1,3-thiazinan-4-one (6n)

Yield: 80%, oil, ^1^H NMR (600 MHz, CDCl_3_) δ (ppm, *J*_H-H_= Hz): 7.29 (dd, 1H, Ar, ^2 ^*J* = 11.1, ^3 ^*J* = 4.3), 6.98 (d, 1H, Ar, ^3 ^*J* = 7.2), 6.94 (d, 1H, Ar, ^3 ^*J* = 7.5), 6.92 (d, 1H, Ar, ^3 ^*J* = 8.1), 6.03 (s, 1H, H2), 4.12 (ddd, 1H, H7a, ^2 ^*J* = 13.4, ^3 ^*J* = 7.9, ^3 ^*J* = 5.3), 3.88 (s, 3H, OCH_3_), 2.87–2.80 (m, 4H, H5, H6), 2.78–2.73 (m, 1H, H7b), 2.63–2.58 (m, 2H, H8), 2.55 (sl, 4H, H9), 1.75 (t, 4H, H10, ^3 ^*J* = 5.9). ^13 ^C NMR (150 MHz, CDCl_3_) δ (ppm): 169.7 (C4), 156.2, 129.3, 127.4, 126.1, 120.0, 111.3, 56.7 (C2), 55.6, 54.2, 53.5, 46.6, 34.8 (C5), 23.6, 21.7 (C6). MS (70 eV): m/z (%) = 320 (M^+^, 2%), 222 (2%), 190 (1%), 148 (2%), 136 (1%), 121 (6%), 97 (54%), 84 (100%), 70 (4%) 56 (4%), 42 (12%).

##### 2-(3-hydroxyphenyl)-3-(2-(pyrrolidin-1-yl)ethyl)-1,3-thiazinan-4-one (6o)

Yield: 87%, oil, ^1^H NMR (600 MHz, CDCl_3_) δ (ppm, *J*_H-H_= Hz): 7.14 (t, 1H, Ar, ^3 ^*J* = 7.9), 6.65 (t, 1H, Ar, ^3 ^*J* = 6.3), 6.53 (s, 1H, Ar), 5.56 (s, 1H, H2), 4.23 (dd, 1H, H7a, ^2 ^*J* = 15.5, ^3 ^*J* = 6.6), 2.88–2.81 (m, 2H, H5a, H6a), 2.81–2.76 (m, 2H, H5b, H6b), 2.75–2.68 (m, 2H, H7b, H8a), 2.63 (sl, 4H, H9), 2.60–2.54 (m, 1H, H8b), 1.80 (sl, 4H, H10). ^13 ^C NMR (150 MHz, CDCl_3_) δ (ppm): 169.9 (C4), 157.6, 140.7, 129.9, 117.8, 115.5, 113.7, 62.4 (C5), 54.2, 53.3, 47.0, 34.4 (C5), 23.5, 21.7 (C6). MS (70 eV): *m/z* (%) = 306 (M^+^,1%), 134 (2%), 120 (1%), 107 (3%), 97 (32%), 84 (100%), 70 (4%), 56 (5%), 42 (12%).

##### 2-(2,3-dimethoxyphenyl)-3-(2-(pyrrolidin-1-yl)ethyl)-1,3-thiazinan-4-one (6p)

Yield: 87%, oil, ^1^H NMR (600 MHz, CDCl_3_) δ (ppm, *J*_H-H_= Hz): 7.02 (t, 1H, Ar, ^3 ^*J* = 8.0), 6.91 (d, 1H, Ar, ^3 ^*J* = 8.1), 6.60 (d, 1H, Ar, ^3 ^*J* = 7.7), 6.04 (s, 1H, H2), 4.14–4.10 (m, 1H, H7a), 3.95 (s, 3H, OCH_3_), 3.88 (s, 3H, OCH_3_), 2.94–2.74 (m, 5H, H5, H6, H7b), 2.70–2.63 (m, 2H, H8), 2.61 (m, 4H, H9), 1.80–1.76 (m, 4H, H10). ^13 ^C NMR (150 MHz, CDCl_3_) δ (ppm): 169.5 (C4), 152.9, 145.8, 132.8, 123.1, 118.0, 112.5, 62.7 (C2), 56.7, 55.8, 54.0, 53.2, 46.2, 34.5 (C5), 23.4, 21.4 (C6). MS (70 eV): *m/z* (%) = 350 (M^+^, 2%), 252 (2%), 178 (1%), 166 (1%), 151 (5%), 136 (1%), 121 (2%), 97 (57%), 84 (100%), 70 (4%) 56 (4%), 42 (12%).

##### 2-(2,4-dichlorophenyl)-3-(2-(pyrrolidin-1-yl)ethyl)-1,3-thiazinan-4-one (6q)

Yield: 78%, oil, ^1^H NMR (600 MHz, CDCl_3_) δ (ppm, *J*_H-H_= Hz): 7.48 (s, 1H, Ar), 7.27 (d, 1H, Ar, ^3 ^*J* = 9.8), 6.95 (d, 1H, Ar, ^3 ^*J* = 8.3), 6.04 (s, 1H, H2), 4.17–4.11 (m, 1H, H7a), 3.18–3.08 (m, 1H, H6a), 2.90 (d, 1H, H5a, ^3 ^*J* = 7.3), 2.88–2.84 (m, 4H, H9), 2.82 (dd, 2H, H5b, H6b, ^2 ^*J* = 13.7, ^3 ^*J* = 7.5), 2.77–2.70 (m, 1H, H7b), 2.65–2.50 (m, 2H, H8), 1.87 (sl, 4H, H10). ^13 ^C NMR (150 MHz, CDCl_3_) δ (ppm): 176.9, 169.9, 134.8, 133.7, 130.9, 127.2, 126.8, 59.5 (C2), 53.7, 52.7, 45.7, 35.9, 34.6 (C5), 23.5, 21.2 (C6). MS (70 eV): *m/z* (%) = 358 (M^+^-1, 1%), 189 (1%), 173 (1%), 159 (2%), 123 (1%), 97 (27%), 84 (100%), 82 (1%), 69 (3%) 56 (4%), 42 (12%).

##### 2-(2,6-dichlorophenyl)-3-(2-(pyrrolidin-1-yl)ethyl)-1,3-thiazinan-4-one (6r)

Yield: 81%, oil, ^1^H NMR (600 MHz, CDCl_3_) δ (ppm, *J*_H-H_= Hz): 7.34 (sl, 2H, Ar), 7.20 (t, 1H, Ar, ^3 ^*J* = 8.0), 6.58 (s, 1H, H2), 4.24–4.17 (m, 1H, H7a), 3.10–3.01 (m, 1H, H7b), 2.93–2.88 (m, 3H, H5, H6a), 2.86–2.81 (m, 1H, H6b), 2.65 (dt, 1H H8a, ^2 ^*J* = 13.8, ^3 ^*J* = 6.9), 2.53–2.44 (m, 5H, H8b, H9), 1.75–1.72 (m, 4H, H10). ^13 ^C NMR (150 MHz, CDCl_3_) δ (ppm): 169.6, 133.2, 129.7, 59.6 (C2), 54.3, 53.6, 44.6, 35.5 (C5), 24.0, 23.6 (C6). MS (70 eV): *m/z* (%) = 358 (M^+^-1, 1%); 186 (1%); 174 (1%); 159 (2%); 123 (1%); 97 (46%); 84 (100%); 69 (4%) 55 (12%); 42 (7%).

##### 2-(2-chloro-6-fluorophenyl)-3-(2-(pyrrolidin-1-yl)ethyl)-1,3-thiazinan-4-one (6 s)

Yield: 79%, oil, ^1^H NMR (600 MHz, CDCl_3_) δ (ppm, *J*_H-H_= Hz): 7.23 (dd, 2H, Ar, ^3 ^*J* = 9.8, ^3 ^*J* = 5.4), 7.07–6.99 (m, 1H, Ar), 6.26 (s, 1H, H2), 4.14–4.09 (m, 1H, H7a), 3.10–3.01 (m, 1H, H6a), 2.89–2.86 (m, 3H, H5, H6b), 2.85–2.81 (m, 1H, H7b), 2.79–2.74 (m, 1H, H8a), 2.53 (dd, 1H, H8b, ^2 ^*J* = 12.9, ^3 ^*J* = 7.6), 2.51-2.46 (m, 4H, H9), 1.73 (sl, 4H, H10). ^13 ^C NMR (150 MHz, CDCl_3_) δ (ppm): 169.1 (C4), 161,1 (d, ^1^*J*_C-F_= 250.3), 134.1, 129.7 (d, ^3^*J*_C-F_= 10.2), 125.4 (d, ^3^*J*_C-F_= 11.9). 115.7 (d, ^2^*J*_C-F_= 24.3), 58.1 (C2), 54.2, 54.1, 45.8, 34.8 (C5), 23.6, 23.3 (C6). MS (70 eV): *m/z* (%) = 342 (M^+^, 1%), 170 (2%), 157 (1%), 139 (1%), 120 (1%), 97 (46%), 84 (100%), 70 (3%) 56 (3%), 42 (14%).

##### 2-(2,5-dimethoxyphenyl)-3-(2-(pyrrolidin-1-yl)ethyl)-1,3-thiazinan-4-one (6x)

Yield: 46%, oil, ^1^H NMR (600 MHz, CDCl_3_) δ (ppm, *J*_H-H_= Hz): 6.85 (d, 1H, Ar, ^3 ^*J* = 8.8), 6.80 (dd, 1H, Ar, ^3 ^*J* = 8.8, ^4 ^*J* = 2.7), 6.58 (d, 1H, Ar, ^4 ^*J* = 2.4), 6.00 (s, 1H, H2), 4.14 (ddd, H7a, ^2 ^*J* = 13.4, ^3 ^*J* = 8.0, ^3 ^*J* = 5.3), 3.84 (s, 3H, OCH_3_), 3.75 (s, 3H, OCH_3_), 2.93–2.88 (m, 1H, H6a), 2.84 (dd, 2H, H5a, H6b, ^2 ^*J* = 10.3, ^3 ^*J* = 5.6), 2.83–2.79 (m, 1H, H5b), 2.73 (dd, 1H, H7b, ^2 ^*J* = 13.7, ^3 ^*J* = 7.3), 2.62–2.55 (m, 2H, H8), 2.52 (d, 4H, H9, ^3 ^*J* = 5.1), 1.74 (sl, 4H, H10). ^13 ^C NMR (150 MHz, CDCl_3_) δ (ppm): 169.4, 153.2, 150.3, 128.5, 113.3, 112.7, 112.0, 56.5 (C2), 56.0, 55.8, 54.2, 53.5, 46.6, 34.7 (C5), 23.6, 21.6 (C6). MS (70 eV): *m/z* (%) = 350 (M^+^, 3%), 253 (4%), 178 (1%), 166 (2%), 151 (8%), 137 (1%), 121 (4%), 97 (48%), 84 (100%), 70 (4%) 56 (5%), 42 (14%).

##### 2-(3,4-dimethoxyphenyl)-3-(2-(pyrrolidin-1-yl)ethyl)-1,3-thiazinan-4-one (6w)

Yield: 31%, oil, ^1^H NMR (600 MHz, CDCl_3_) δ (ppm, *J*_H-H_= Hz): 6.82 (d,1H, Ar, ^3 ^*J* = 8.2), 6.79 (s, 1H, Ar), 6.73 (d, 1H, Ar, ^3 ^*J* = 7.3), 5.73 (s, 1H, H2), 4.22–4.15 (m, 1H, H7a), 3.88 (d, 6H, OCH_3_, ^4 ^*J* = 2.8), 2.90–2.76 (m, 5H, H5, H6, H7b), 2.71–2.60 (m, 2H, H8), 2.53 (sl, 4H, H9), 1.75 (sl, 4H, H10). ^13 ^C NMR (150 MHz, CDCl_3_) δ (ppm): 169.4, 149.4, 149.0, 131.8, 118.8, 110.6, 110.0, 62.6 (C2), 56.0, 54.3, 53.8, 46.6, 34.6 (C5), 23.6, 22.1 (C6). MS (70 eV): *m/z* (%) = 350 (M^+^, 2%), 252 (1%), 181 (1%), 166 (4%), 151 (6%), 136 (1%), 120 (1%), 97 (33%), 84 (100%), 70 (7%) 56 (4%), 42 (11%).

## Biological activity

### Effects in vitro of thiazolidin-4-ones and thiazinan-4-ones in the brain AChE activity

*In vitro* was evaluated the potential inhibition of thiazolidin-4-ones and thiazinan-4-ones against AChE activity enzyme in hippocampus and cerebral cortex of Wistar rats (60 days, 250–300 g), since it is well described that these two structures in the brain play an important role in memory. All procedures involving the animals were approved by the Committee of Ethics in Animal Experimentation of UFPel (CEEA 9219). The animals were submitted to euthanasia and the brain was removed. Subsequently, the hippocampus and cerebral cortex were separated and homogenised in buffer Tris HCl 10 mM (pH 7.4). The homogenates were centrifuged at 1800 rpm for 10 min and the supernatant was used for *in vitro* analyses of the potential inhibitory effect of thiazolidin-4-one and thiazinan-4-ones on AChE activity.

The compounds were solubilised in methanol and prepared in different concentrations (0.1, 0.5, 1, 5, 10, 25, 50, 100 and 250 μM). The AChE activity was determined on a microplate according to the method of Elmann *et al.*^28^ which demonstrates as a principle the hydrolysis of the substrate acetylcholine which is converted into two products, acetate and thiocoline. The thiocholine, in turn, reacts with DTNB to form a chromophore, 5-thio-2-nitrobenzoic, which is quantified spectrophotometrically at 412 nm. The enzymatic activity was expressed in μmols of AcSCh/h/mg protein. Proteins were determined by the method of Bradford^29^ using the Coomassie Blue reagent. Bovine albumin was used as standard. It was emphasised that besides the water control, the action of methanol on AChE activity was also determined, in order to confirm that the solvent does not interfere with the enzymatic activity. Based on the results, it can be shown that methanol did not alter AChE activity compared to the water control group (*p* > .05).

### Cytotoxicity evaluation the thiazolidin-4-ones and thiazinan-4-ones in cortical astrocytes culture

In the evaluation of the cytotoxicity of the thiazolidin-4-ones and thiazinan-4-ones in astrocytes, Wistar rats (1–2 days) were used to obtain cortical astrocytes, which were cultured in 96-well plates (3x10^4^ per well) and maintained with Dulbecco’s modified Eagle’s medium (DMEM) supplemented with 10% Bovine Foetal Serum (FBS)^30^. Cells were maintained under standard conditions (5% CO_2_, 37 °C and humidified atmosphere) for 15 days, receiving periodic exchange of culture medium until treatments were received. After time and maturation, the astrocytes were treated with the compounds at the concentration of 100 μM. Cells were exposed to this concentration for 48 h and 72 h. After treatment, cell viability was analysed by the test of 3-(4,5-dimethylthiazol-2yl)-2,5-diphenyltetrazolium bromide (MTT). This method consists of measuring the number of cells with the metabolically active mitochondria, based on the reduction of the tetrazolium salt to the formazan crystals. The absorbance was determined on a microplate reader at 492 nm. The absorbance is linearly proportional to the number of cells with active mitochondria, indicating how many viable cells remained at the end of the treatment.

### Statistical analysis

Results were analysed by using one-way analysis of variance (ANOVA) followed by Tukey test for multiple comparisons in the software Graphpad Prism 5. The statistical difference was considered significant when (*p* < .05) and all results were expressed as mean ± standard error.

## Results

### Chemistry

**Scheme 1.**
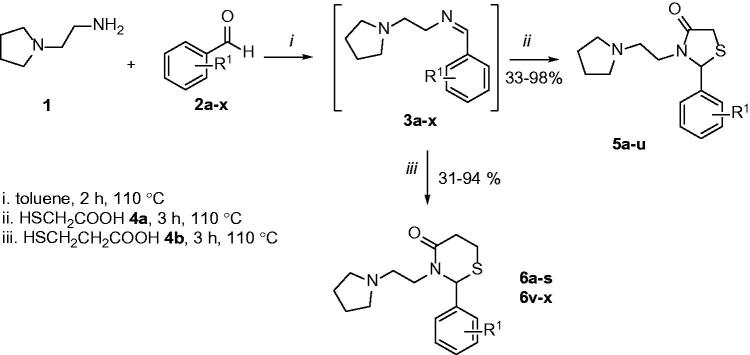
Synthetic pathway to the obtention of thiazolidinones and thiazinanones.

In continuation of our research programme, the synthetic route used to synthesise thiazolidin-4-ones (**5**) and thiazinan-4-ones (**6**) is illustrated in Scheme 1. Mercaptoacetic acid (**4a**) was used to obtain thiazolidin-4-ones (**5**) and mercaptopropionic acid (**4b**) was used to obtain thiazinan-4-ones **6**. It was used arenealdehydes (**2**) with substituents in *ortho*, *meta* and *para* positions, with electron-donating groups (CH_3_, OH, OCH_3_) and electron-withdrawing groups (F, Cl, NO_2_). Accordingly, the 1-(2-aminoethyl)pyrrolidine (**1**) reacts with different benzaldehydes (**2a-x**) and an appropriate carboxylic acid (**4a-b**) in toluene reflux for 5 h to give thiazolidin-4-ones (**5a-u**) and thiazinan-4-ones (**6a-s**,**v-x**).

During the process, the reactions were monitored by thin-layer chromatography (TLC) and some products required a purification step by washing with hot hexane or hot hexane/ethyl acetate (8:2). The products were obtained with good to moderate yields and the melting point analysis was performed for the solid compounds. All compounds were characterised and confirmed by GC/MS and ^1^H, ^13 ^C NMR analyses. Compound **5i** has also been analysed in 2 D NMR (COSY, HSQC, HMBC). Compounds **5d, 5 g, 5j, 5k** and **5o** were also identified by HRMS.

In the ^1^H NMR spectra, the signals that confirm the formation of thiazolidin-4-one are the H2 and the diastereopic H5 hydrogens. For thiazinan-4-one the signals are the H2 and diastereopic H5 and H6 hydrogens. For thiazolidin-4-one (**5**), hydrogen H5a appears as a double doublet and H5b as a doublet signal in the range of 3 to 4 ppm. For thiazinan-4-one (**6**), H5a, H5b, H6a and H6b appear as multiplets due to overlapping of signals in the range of 2 to 3 ppm with spectral signal of H6 more deshielded than H5. Another characteristic signal present in both heterocycles is the H2 belonging to the asymmetric carbon C2 that appears in the range of 5 to 6 ppm as singlet or doublet. In the ^13 ^C NMR spectra, the characteristic signals of thiazolidin-4-one and thiazinan-4-one are C4, C2 and C5 (plus C6 for thiazinan-4-one). The carbonyl C4 is the most deshielded signal in the spectrum at 170 ppm. The methyne C2 appears at 55.9-64.1 ppm for both heterocycles. The thiazolidin-4-ones (**5**) and thiazinan-4-ones (**6**) have the sign of C5 in the region of 31.6–34.8 ppm. The thiazinan-4-ones (**6**) also have the sign of C6 in the region of 23.3-21.2 ppm. Other signals belong to the amine and corresponding arenealdehyde.

In the COSY spectrum of thiazolidin-4-one (**5i**) it was possible to confirm the H2 at 6.00 ppm because coupling with the diastereopic hydrogen H5a at 3.80 ppm. In the HMQC spectrum the carbon of diastereopic hydrogens H5a and H5b was verified at 32 ppm. Furthermore, it is also important to show the elucidation the C6 and C7 for thiazolidin-4-one (**5i**) (about 41 and 54 ppm, respectively). There is a chemical displacement inversion between C6 and C7 compared to the respective hydrogens H6 and H7. The H6 has a deshielded signal compared to the H7, however C6 has a shielded signal compared to C7. A possible explanation is due the conformational freedom of ethylene link. The HMBC spectrum confirms these assignments.

## Anticholinesterase and cytotoxic studies

**Figure 2.**
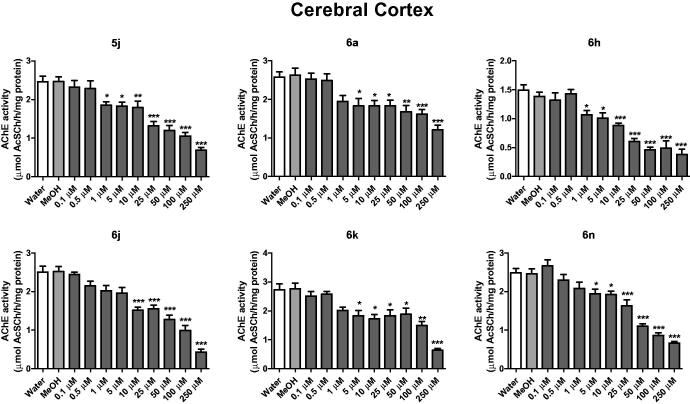
*In vitro* effect of compounds **5j**, **6a**, **6 h**, **6j**, **6k** and **6n** on the activity of AChE in cerebral cortex of rats. *p < .05, **p < .01, ***p < .001 when compared to control group (water or MeOH).

**Figure 3.**
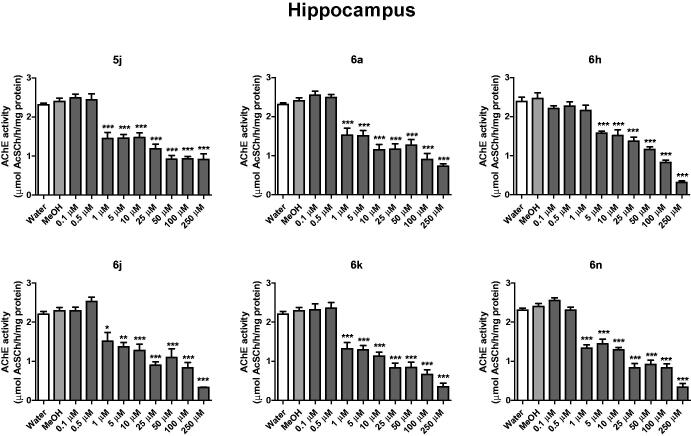
*In vitro* effect of compounds **5j**, **6a**, **6 h**, **6j**, **6k** and **6n** on the activity of AChE in cerebral hippocampus of rats. *p < .05, **p < .01, ***p < .001 when compared to control group (water or MeOH).

In cerebral cortex, compounds **5j** and **6h** inhibited AChE activity from the concentration of 1 μM, while compounds **6a**, **6n** and **6k** from 5 μM and compound **6j** from 10 μM (Figure 2). In the hippocampus, compounds **5j**, **6a**, **6j**, **6k** and **6n** inhibited AChE activity from 1 μM, when compared to the control group (water and methanol) (*p* < .05) (Figure 3). The compound 6 h inhibited the AChE activity of hippocampus at concentration of 5 μM (Figure 3).

In cerebral cortex, the compounds **5i** inhibited AChE activity from 10 μM, **6m** and **6o** from 25 μM, **6i** from 50 μM and **6l** from 100 μM when compared to the control group (water and methanol) (*p* < .05). In the hippocampus, compound **5i** inhibited the AChE activity from 10 μM, **6n** from 25 μM, **6i** from 50 μM and **6 l** from 100 μM concentrations. A decrease in activity was found for the disubstituted thiazinanone **6p**. These findings showed that groups in *para* position still had better results.

**Figure 4.**
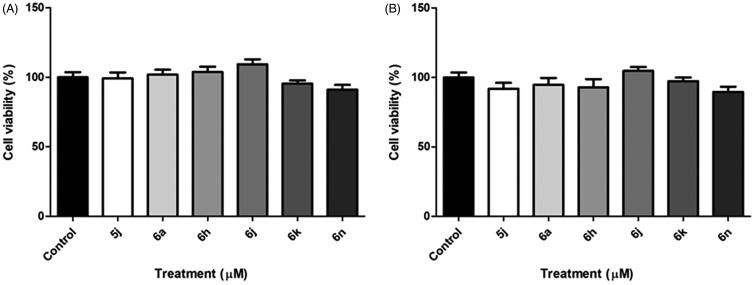
*In vitro* cytotoxicity activity of compounds **5j, 6a, 6 h, 6j, 6k** and **6n** to cell viability of the primary astrocyte culture at 100 μM, after for 48 h (A) and 72 h (B) the treatment.

Based on the results obtained, it can be observed that the molecules with the most promising inhibitory potential against AChE activity were: **5j**, **6a**, **6h**, **6j**, **6k** and **6n.** So, the cytotoxicity of these compounds was evaluated using cortical astrocyte cells cultured in 96-well plates for 15 days. Cells were exposed to the compounds at the concentration of 100 μM for 48 h and 72 h. After treatment, cell viability was determined by the 3-(4,5-dimethylthiazol-2yl)-2,5-diphenyl-tetrazoline (MTT) bromide test. Our results showed that none of the tested compounds were able to alter the cell viability of astrocyte primary culture at the concentration of 100 μM in both times (*p* > .05) (Figure 4).

The IC_50_ values for the most promising compounds were calculated using GraphPad Prism 5 programme (Table 1). Compound **6k** was the most potent with IC_50_ = 6.83 µM for cerebral cortex and IC_50_ = 4.46 µM for hippocampus. In addition, compound **6a** also demonstrated good inhibition activity of AChE with IC_50_ = 7.40 µM for cerebral cortex and IC_50_ = 5.20 µM for hippocampus. Other significant results were found for compounds bearing nitro group. The compound **5j** and **6j** with nitro at *para* position demonstrated good biological activity in hippocampus with IC_50_ = 8.68 µM and IC_50_ = 9.32 µM, respectively. The compound **5i** (*meta* nitro) and **6h** (*ortho* nitro) displayed inhibitory activity in cerebral cortex with IC_50_ = 10.25 µM and IC_50_ = 11.03 µM, respectively.

**Table 1.** IC_50_ of AChE inhibition for thiazolidin-4-ones **5** and thiazinan-4-ones **6**.

	IC_50_ (µM)		R		IC_50_ (µM)	
	Hippocampus	Cortex			Hippocampus	Cortex
**5a**	30.92	>250	H	**6a**	5.20	7.40
**5g**	>250	>250	4-Cl	**6g**	50.48	57.16
**5h**	105.40	>250	2-NO_2_	**6h**	92.80	11.03
**5i**	81.59	10.25	3-NO_2_	**6i**	29.42	50.55
**5j**	8.68	28.15	4-NO_2_	**6j**	9.32	40.06
**5k**	>250	>250	4-CH_3_	**6k**	4.46	6.83
**5l**	nd	nd	2-OCH_3_	**6l**	84.90	>250
**5m**	nd	nd	3-OCH_3_	**6m**	90.30	24.01
**5n**	>250	>250	4-OCH_3_	**6n**	17.02	22.20
**5o**	30.58	27.63	3-OH	**6o**	69.60	25.65

(nd) not determinated.

## Discussion

In the present study, we evaluated *in vitro* the anticholinesterase potential of thiazolidin-4-ones and thiazinan-4-ones from 1-(2-aminoethyl)pyrrolidine compounds. AChE inhibitors are used to increase the acetylcholine level and thus improve the memory decline in AD patients^31^. Investigation of new anticholinesterase compounds is of most importance due to the fact that: (i) epidemiological data have shown the increasing prevalence of AD worldwide, especially due to increased life expectancy^32^^,^^33^; (ii) The current drugs used to treat AD still cause many adverse effects, besides acting only to alleviate the symptoms of the disease, and therefore a palliative alternative therapy besides that presents limitation of loss of therapeutic efficacy with time^33^^,^^34^. In this sense, is very important the search for new possible therapeutic compounds.

The use of AChE from rodents, especially rats, to screen for new compounds with possible anticholinesterase activity is widely used. Possibly this is due to the very high functional and structural similarity of AChE from different species including human- and rat AChE. This similarity is partly due to the equality found in the primary and tertiary structure of AChE^35^^,^^36^. Wiesner et al.^36^ also reported that *Rattus norvegicus* AChEs structures must be very similar to human structure and have properties identical to the active site. Also, although some studies use the isolated form of AChE, the use of crude homogenised from rat’s brain structures, as used in the present work, is widely used and proves to be an important source of AChE for *in vitro* studies^27^.

Here we determinate the *in vitro* thiazolidin-4-ones 5 and thiazinan-4-ones 6 against AChE enzyme inhibition activity in hippocampus and cerebral cortex of rats. First, compounds with different substituents at *para* position of aryl ring with electron-withdrawing (F, Cl, NO_2_) and electron-donating groups (CH_3_, OH, OCH_3_) were used (Figures 2, 3, S132 and S133). The best results were found for thiazolidin-4-one **5j** (–NO_2_) and for thiazinan-4-ones **6a** (–H), **6j** (–NO_2_), **6k** (–CH_3_) and **6n** (–OCH_3_).

Once the best results were found for compounds bearing nitro and methoxy substituents, a selection was performed for thiazolidin-4-ones with substituents –NO_2_ and for thiazinan-4-ones with substituents –NO_2_ and –OCH_3_ at *ortho* and *meta* position of aryl ring (Supplementary information). Analysing the results, compounds with nitro group showed AChE inhibition activity for thiazolidin-4-one **5i** and thiazinan-4-ones **6h** and **6i** and compounds with methoxy group showed AChE inhibition activity for thiazinan-4-one **6l** and **6m**.

Between the two heterocycles tested, the six-membered thiazinan-4-one showed more compounds **6a**, **6h**, **6j**, **6k** and **6n** with good AChE inhibitory. The position of substituent on the aryl ring did not show significant difference for the biological activity with a small preference for the *para* substitution. Disubstituted aryl reduced the activity. Compounds **6a** (H) and **6k** (4-CH_3_) showed the best results in biological activity inhibition of AChE in cerebral cortex and hippocampus with the lowest IC_50_ values, suggesting that a hydrogen bond acceptor (HBA) is not required on the aryl ring. Plus, electronic effects also do not are important for activity once a neutral methyl group or unsubstituted aryl showed the best results.

Previous studies also have evaluated the effects of the others thiazolidin-4-ones compounds on AChE activity. Özadalı et al.^37^ report the synthesis of thiazolidin-4-ones derived from different heterocyclic and showed that these compounds have higher antihistamine activity but not anticholinergic action. Other studies have reported that the presence of pyrrolidine heterocycle in the synthesised products showed a potential inhibitory effect of AChE^38^^,^^39^. In addition, previous research from our research group has demonstrated the synthesis of benzothiazinan-4-one and the potential of some compounds in inhibited the AChE activity from hippocampus and cerebral cortex from rats, demonstrating the importance of this heterocycle in the cholinergic action^27^. Comparing these reports with the study proposed in this paper, I point out that the union of thiazolidin-4-one and thiazinan-4-one with pyrrolidine heterocycle aimed at enhancing AChE inhibition activity, as indeed observed with the results found.

It is important to empathise that the compounds **5j, 6a, 6h, 6j, 6k** and **6n** did not change de cell viability of astrocytes, which is an important cell of the central nervous system. Thus, these compounds become important study targets, since besides presenting a potent AChE inhibitory activity, they did not show toxicity from brain healthy cells.

## Conclusion

In conclusion, two series of thiazolidin-4-ones and thiazinan-4-ones derived from 1-(2-aminoethyl)pyrrolidine were efficient synthesised in moderate to good yields. Tests for AChE inhibition activity showed potent biological activity for thiazolidin-4-one **5j** and thiazinan-4-one **6a**, **6h**, **6j**, **6k** and **6n,** highlighted by thiazinan-4-ones **6a** and **6k** that show the lowest IC_50_ values for hippocampus. It is also highlighted that none of tested compounds showed cytotoxic when evaluated in the cellular viability tests of cortical astrocytes. This study demonstrated safety for the use of thiazinan-4-ones for future *in vivo* assays and may be potential candidates for the limited therapeutic arsenal by treatment of Alzheimer’s disease.

## Supplementary Material

Supplemental Material
