# Supplementary material for: Synthesis of thiazolidin-4-ones and thiazinan-4-ones from 1-(2-aminoethyl)pyrrolidine as acetylcholinesterase inhibitors
Source: J Enzyme Inhib Med Chem. 2019 Oct 23;35(1):31–41. doi: 10.1080/14756366.2019.1680659 (PMC6818106; doi:10.1080/14756366.2019.1680659)
Supplement: Supplemental Material [file IENZ_A_1680659_SM4006.pdf]

Supplementary Material

Synthesis of thiazolidin-4-ones and thiazinan-4-ones from 1-(2-aminoethyl)pyrrolidine as acetylcholinesterase inhibitors

**Table of Contents**	Figure / Table	Page
CG-MS spectra	S01-S42	2-23
^{1}H- and ^{13}C-NMR spectra	S43-S123	24-65
HRMS	S124-S128	66-68
2D NMR (COSY, HSQC and HMBC)	S129-S131	68-69
Tables in vitro AChE activities	TS1 and TS2	70-71
In vitro AChE activities in cerebral cortex and hippocampus	S132-S135	72-75

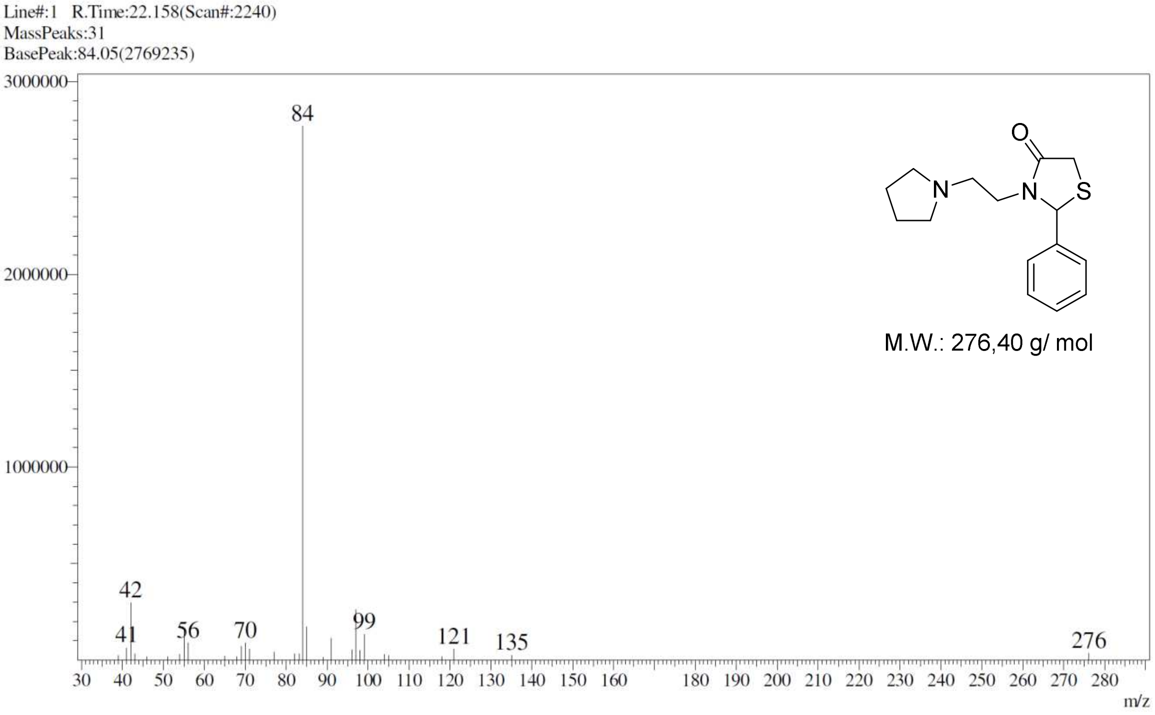

Figure S01: GC/MS of thiazolidin-4-one **5a**.

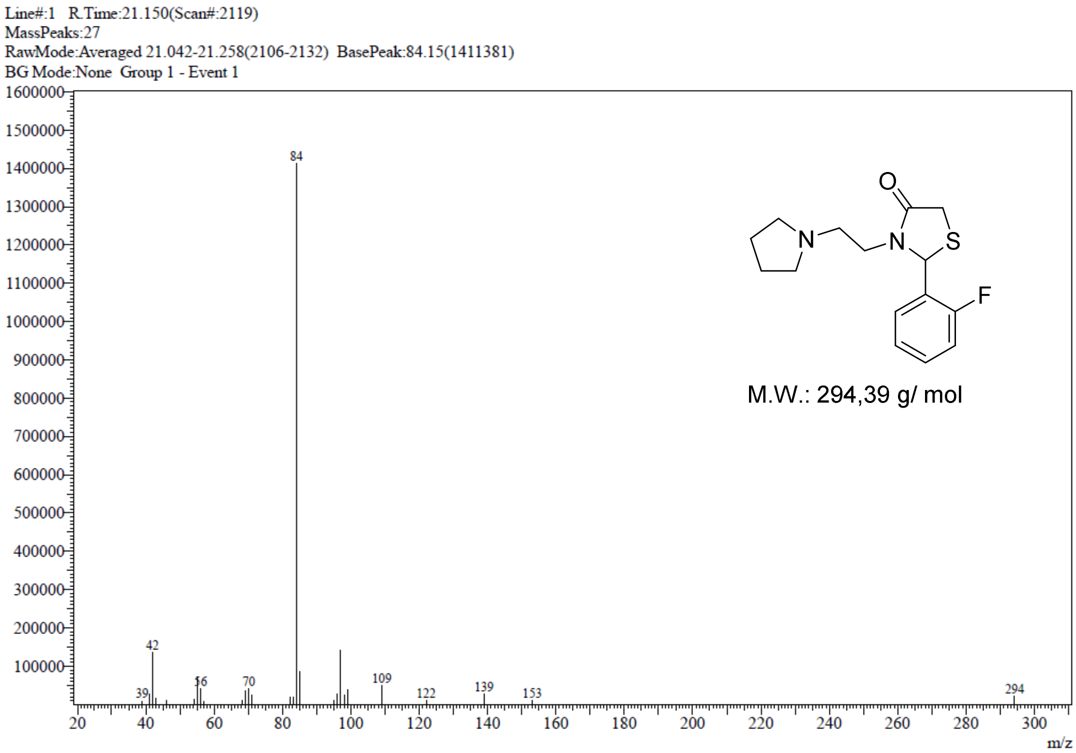

Figure S02: GC/MS of thiazolidin-4-one **5b**.

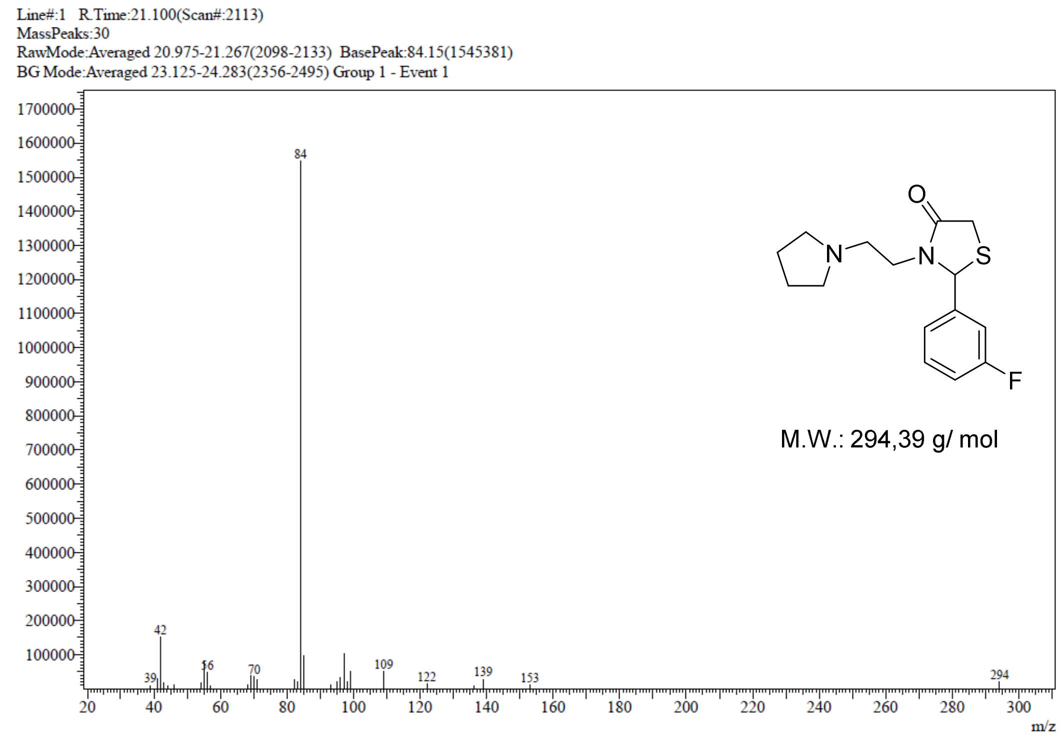

Figure S03: GC/MS of thiazolidin-4-one **5c**.

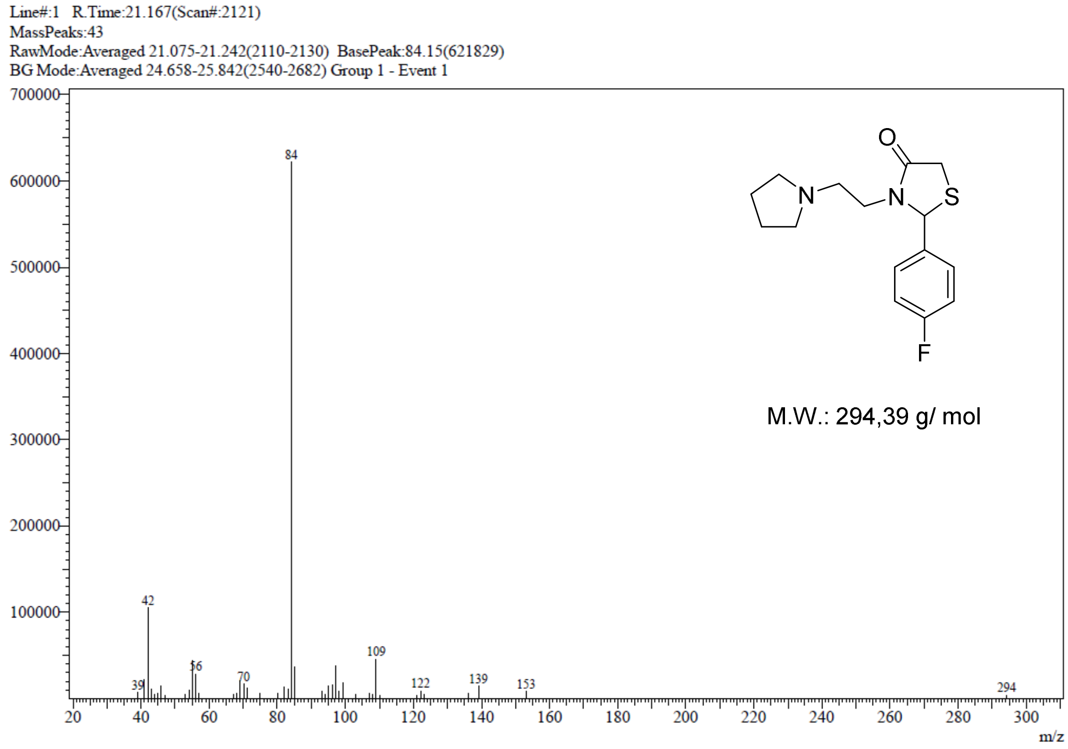

Figure S04: GC/MS of thiazolidin-4-one **5d**.

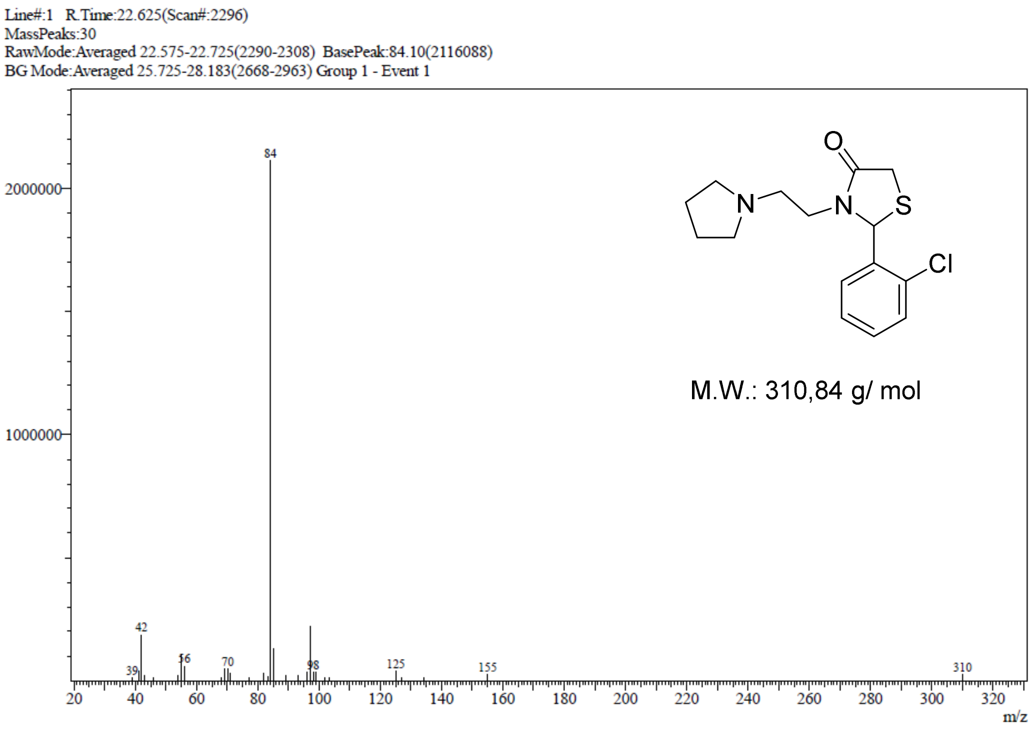

Figure S05: GC/MS of thiazolidin-4-one **5e**.

.

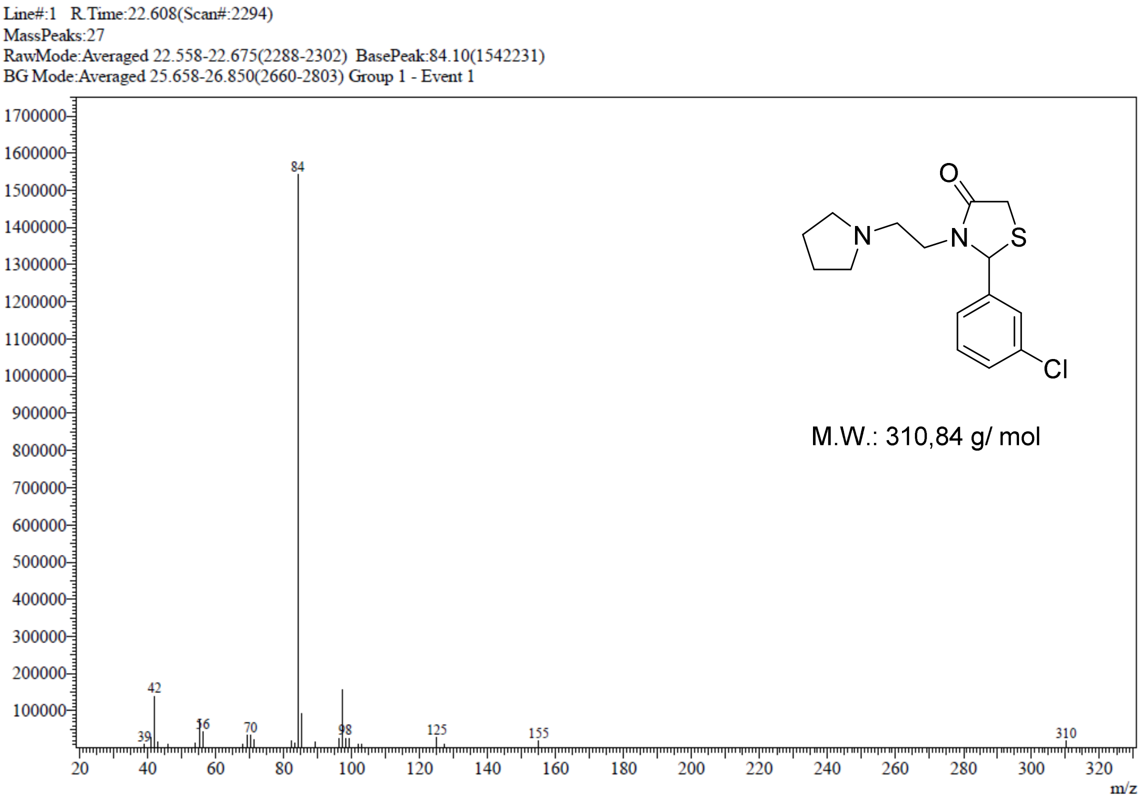

Figure S06: GC/MS of thiazolidin-4-one **5f**.

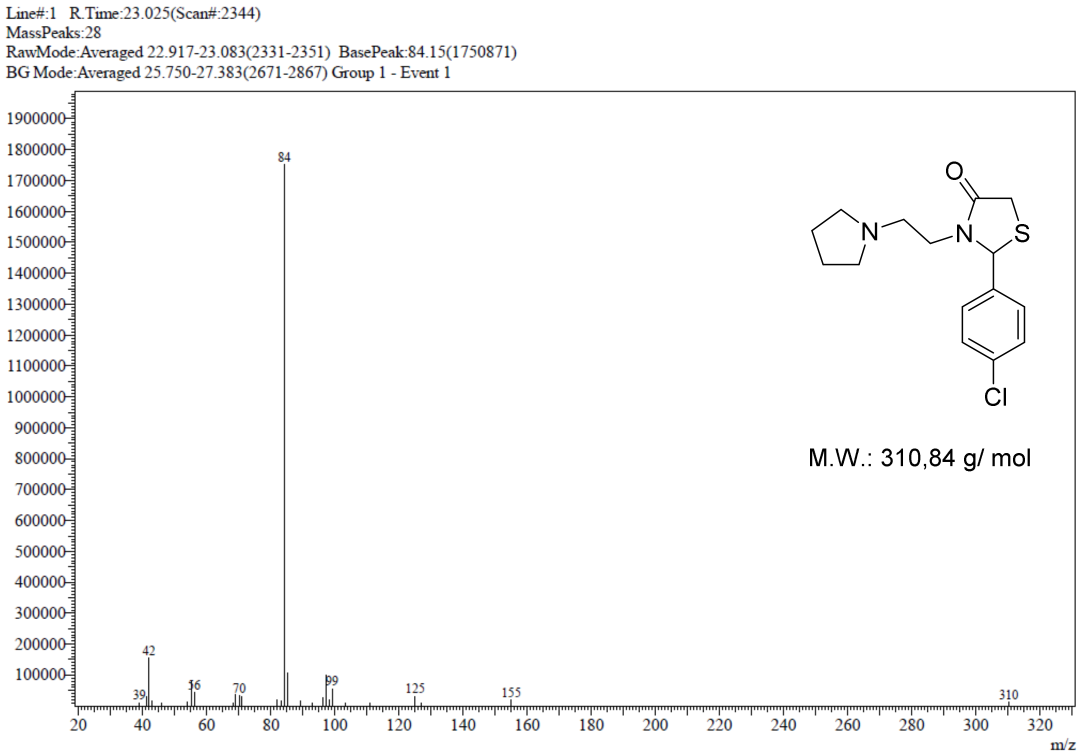

Figure S07: GC/MS of thiazolidin-4-one **5g**.

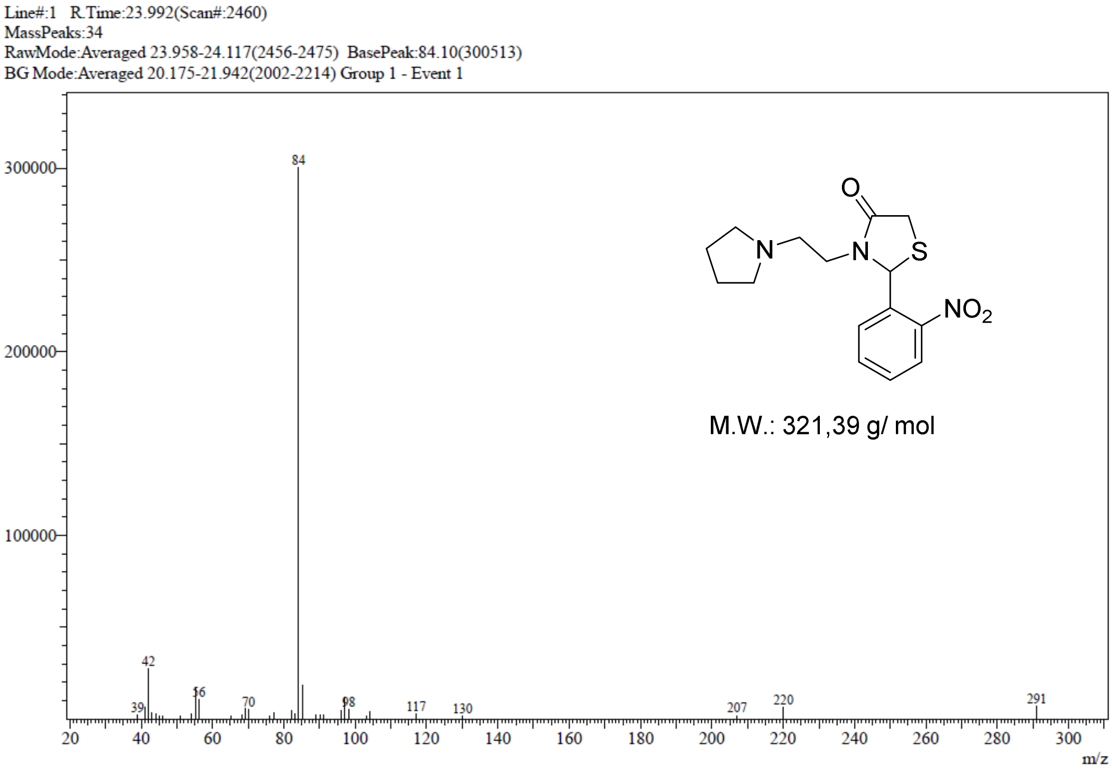

Figure S08: GC/MS of thiazolidin-4-one **5h**.

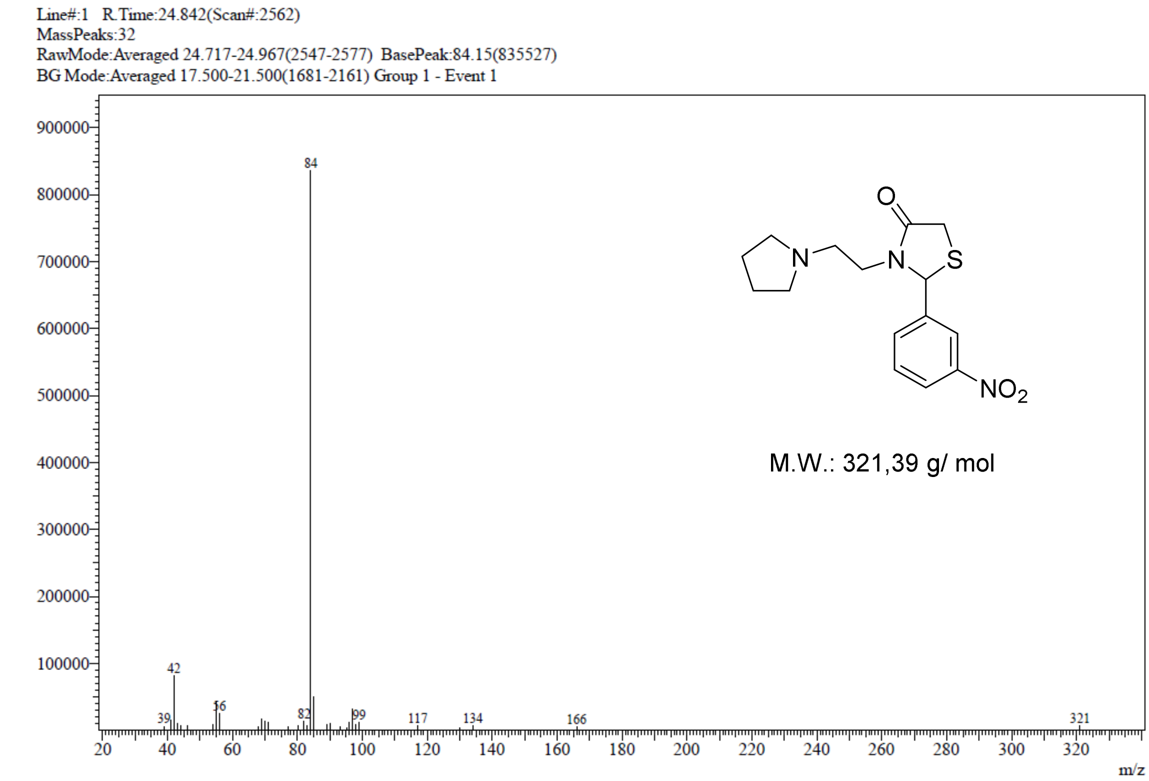

Figure S09: GC/MS of thiazolidin-4-one **5i**.

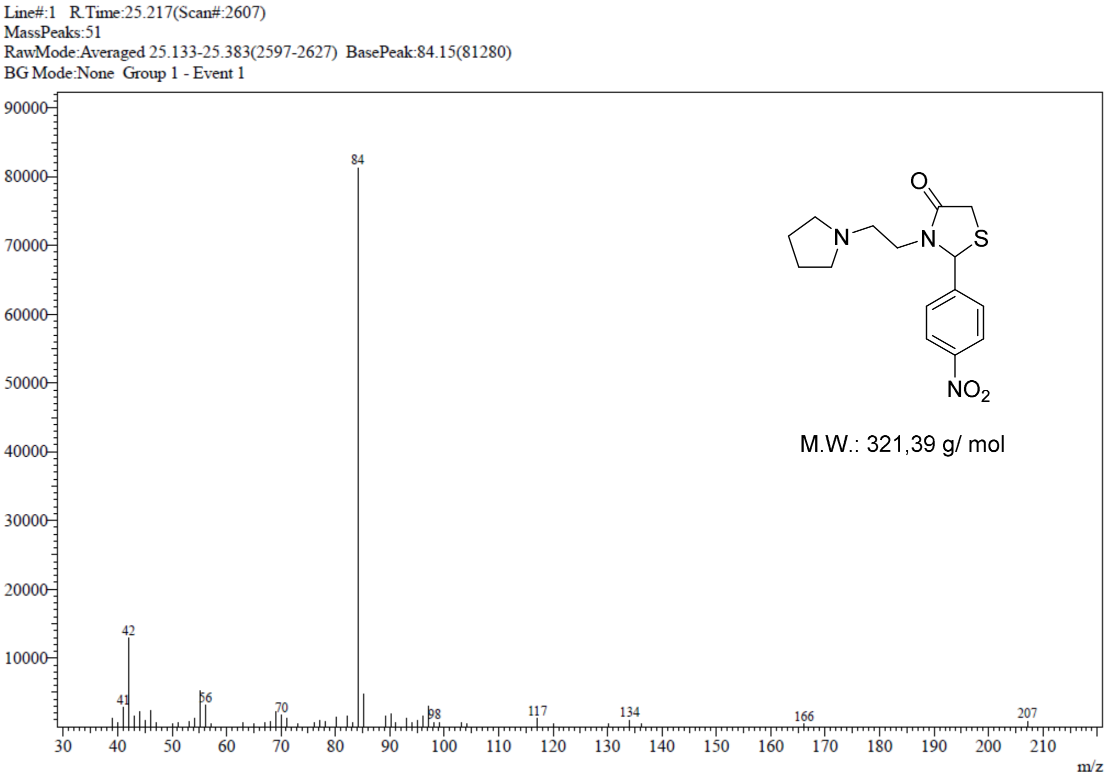

Figure S10: GC/MS of thiazolidin-4-one **5j**.

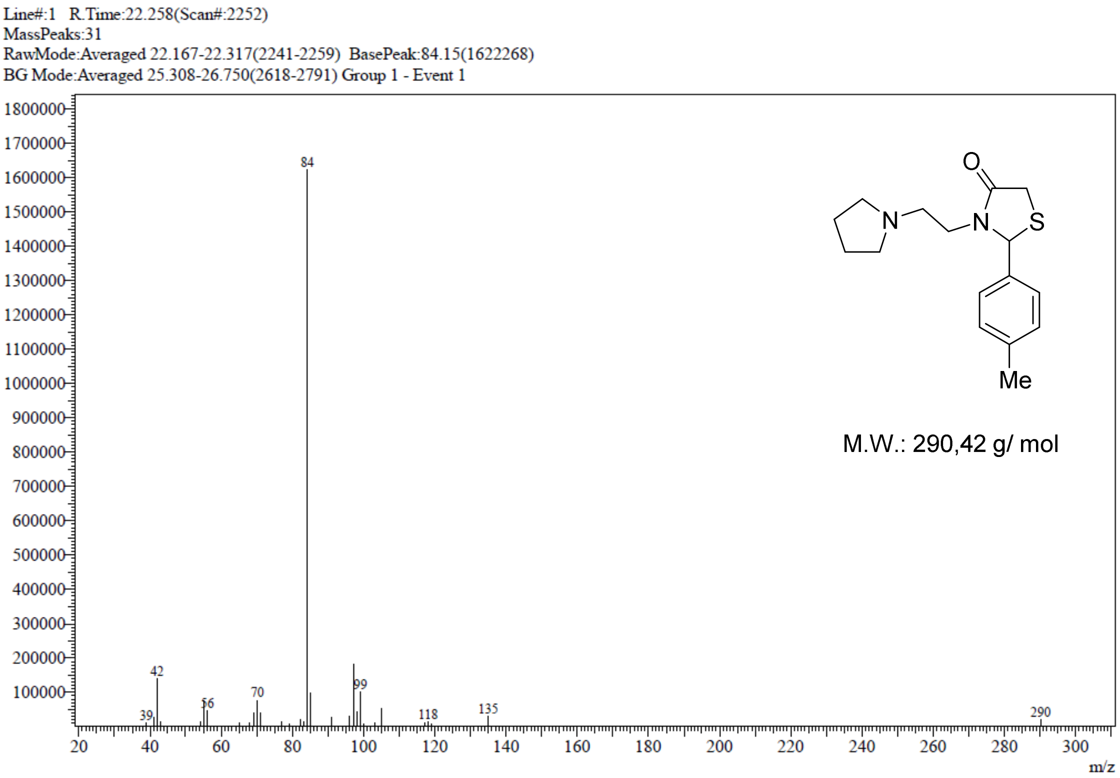

Figure S11: GC/MS of thiazolidin-4-one **5k**.

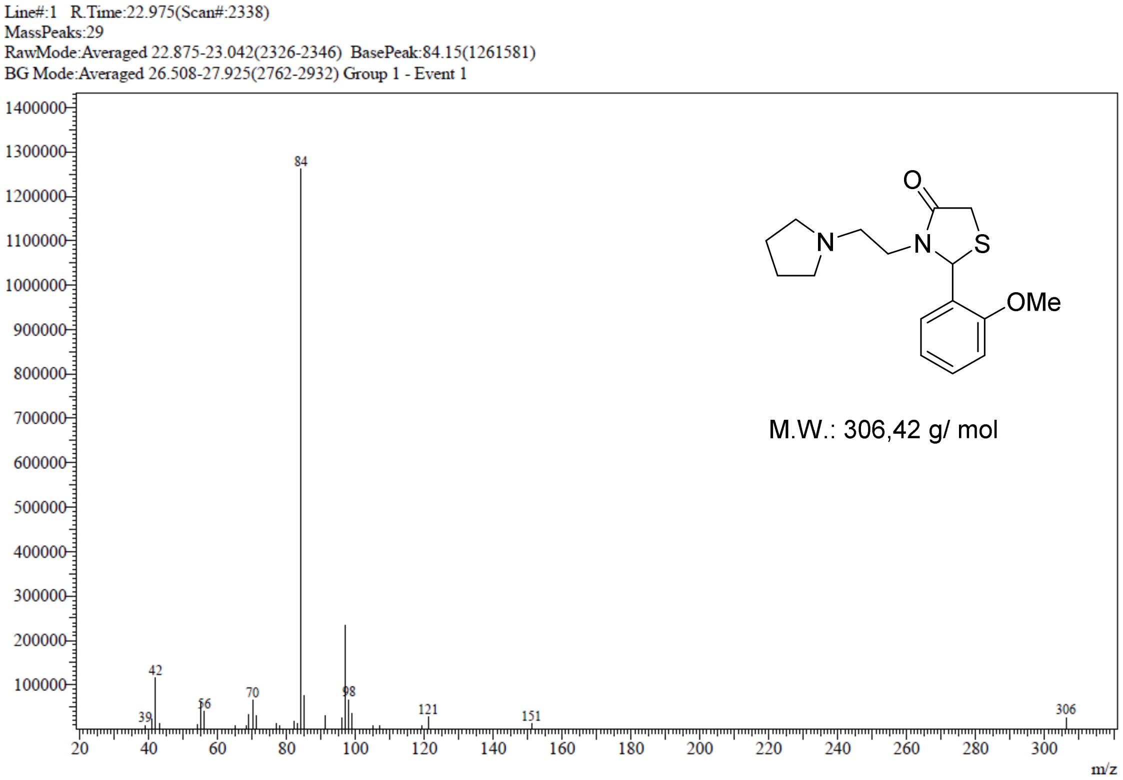

Figure S12: GC/MS of thiazolidin-4-one **5l**.

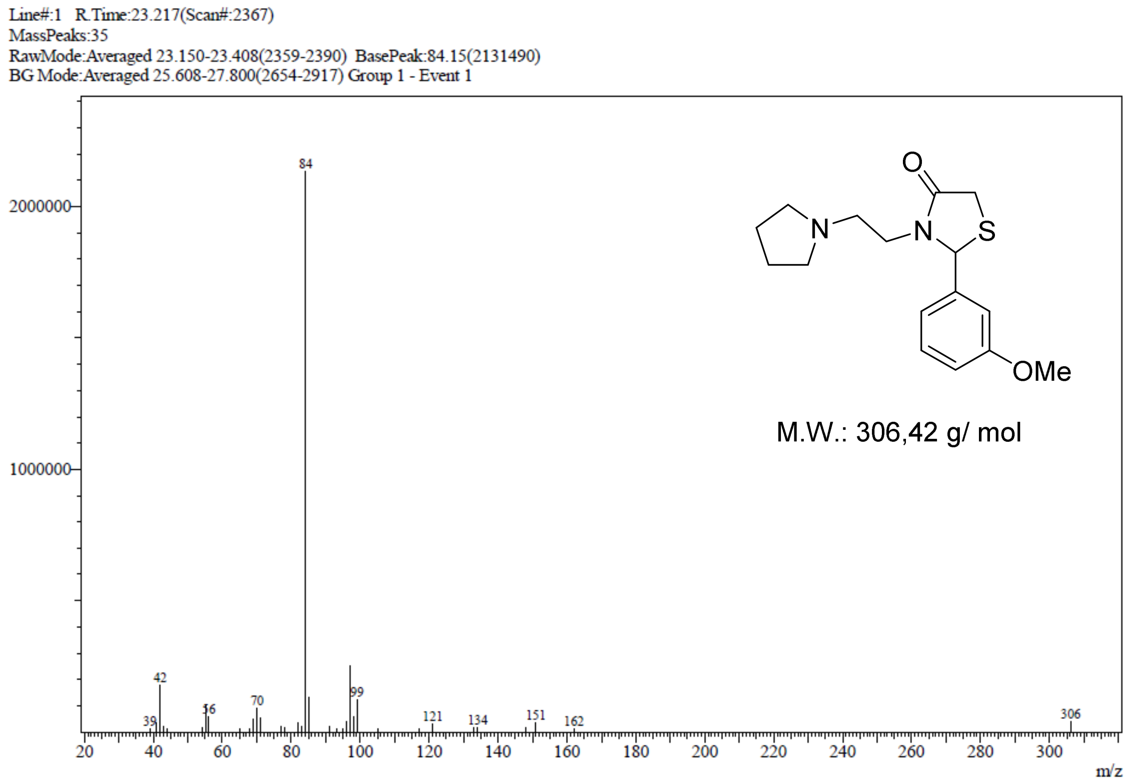

Figure S13: GC/MS of thiazolidin-4-one **5m**.

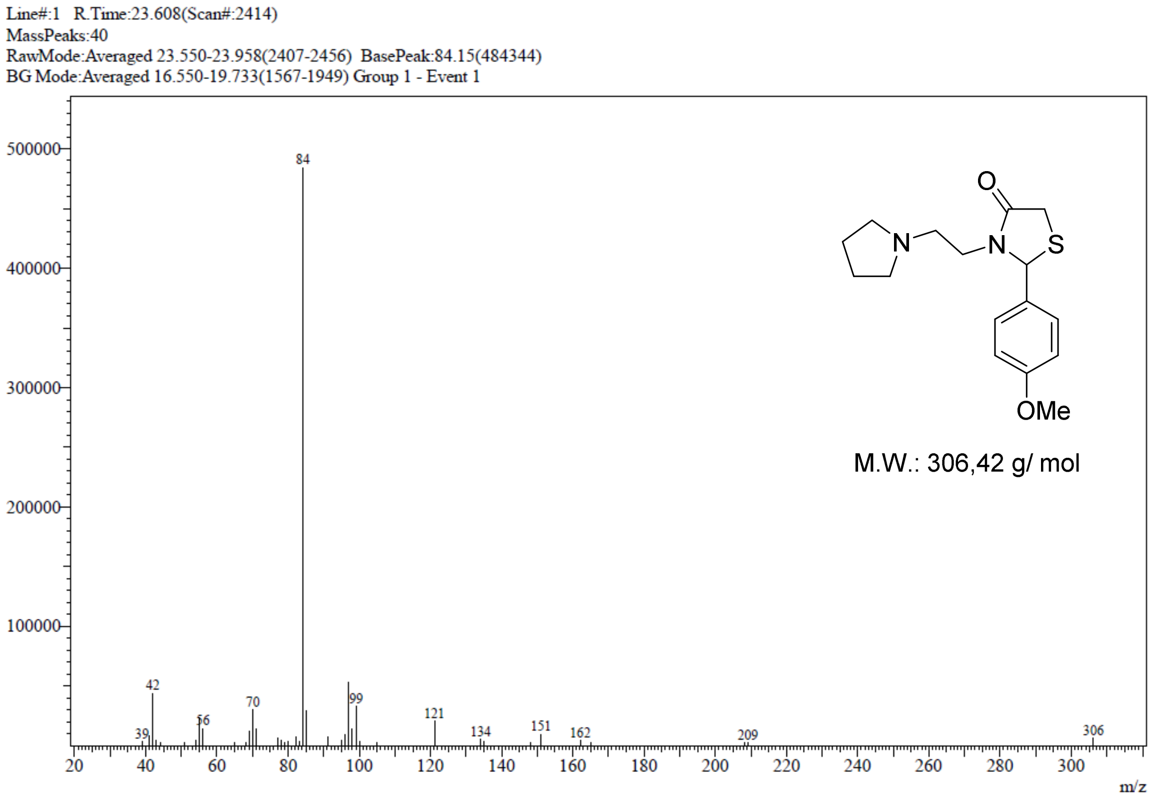

Figure S14: GC/MS of thiazolidin-4-one **5n**.

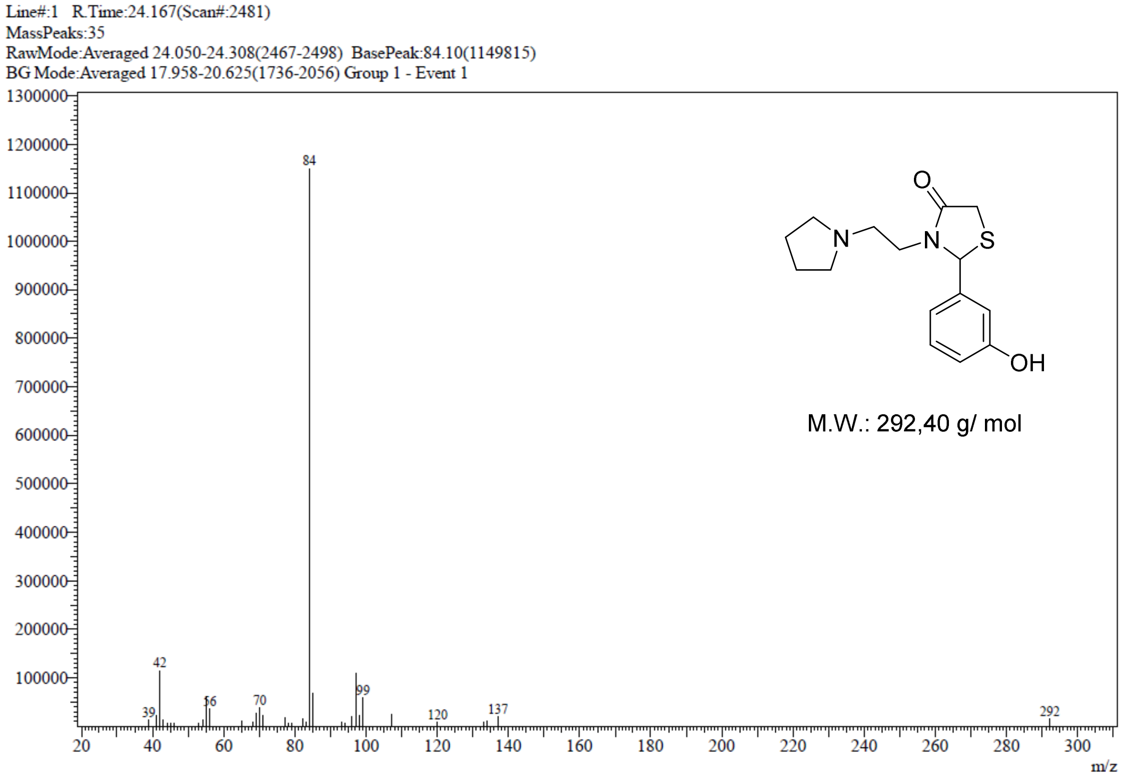

Figure S15: GC/MS of thiazolidin-4-one **5o**.

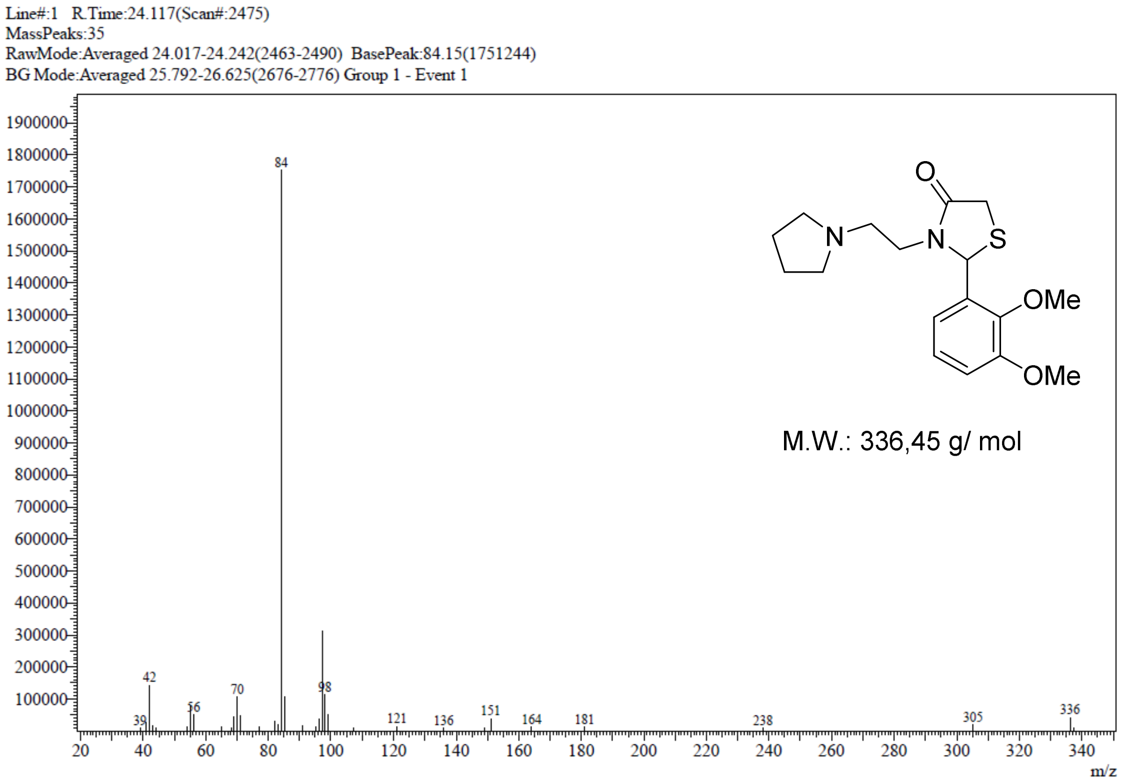

Figure S16: GC/MS of thiazolidin-4-one **5p**.

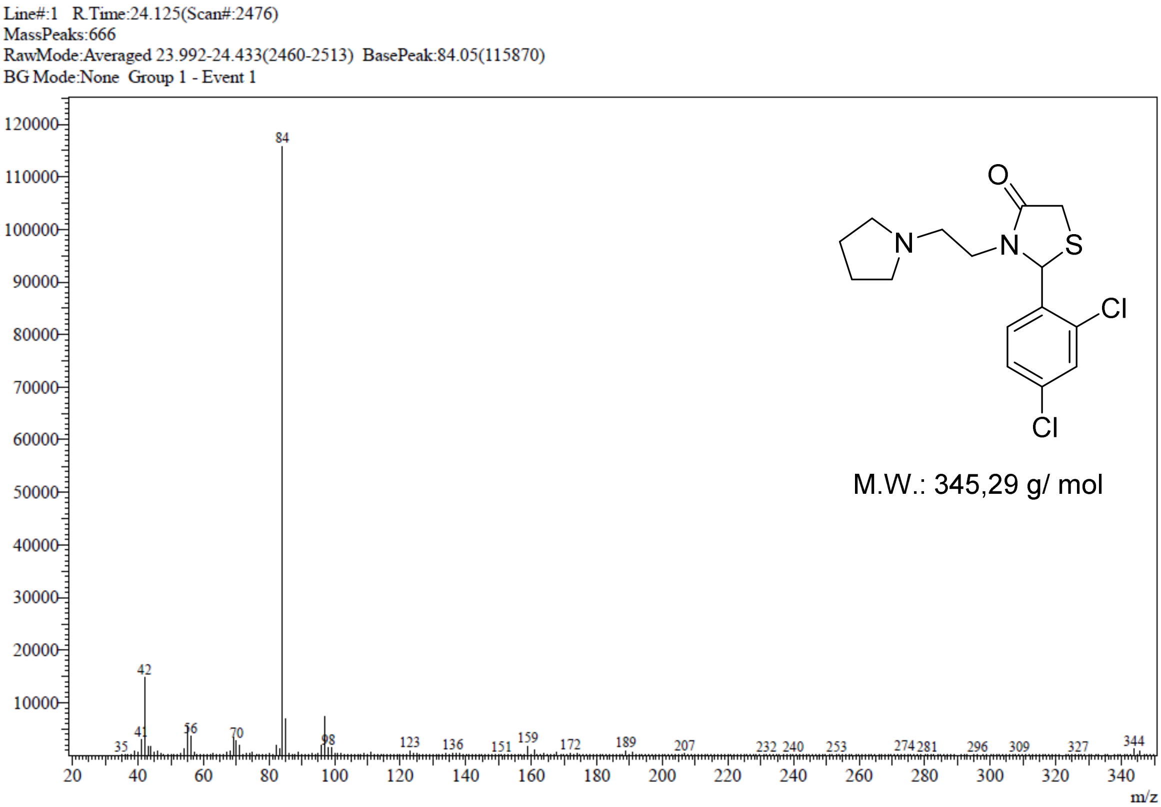

Figure S17: GC/MS of thiazolidin-4-one **5q**.

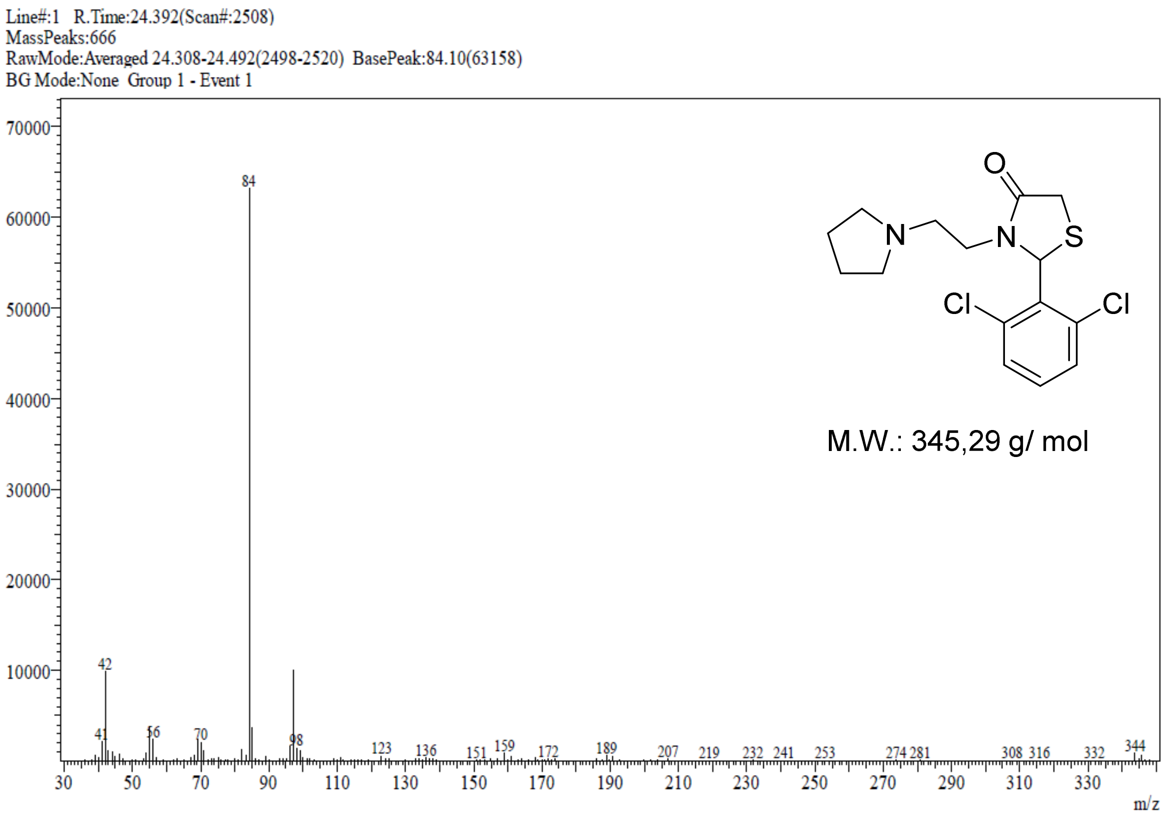

Figure S18: GC/MS of thiazolidin-4-one **5r**.

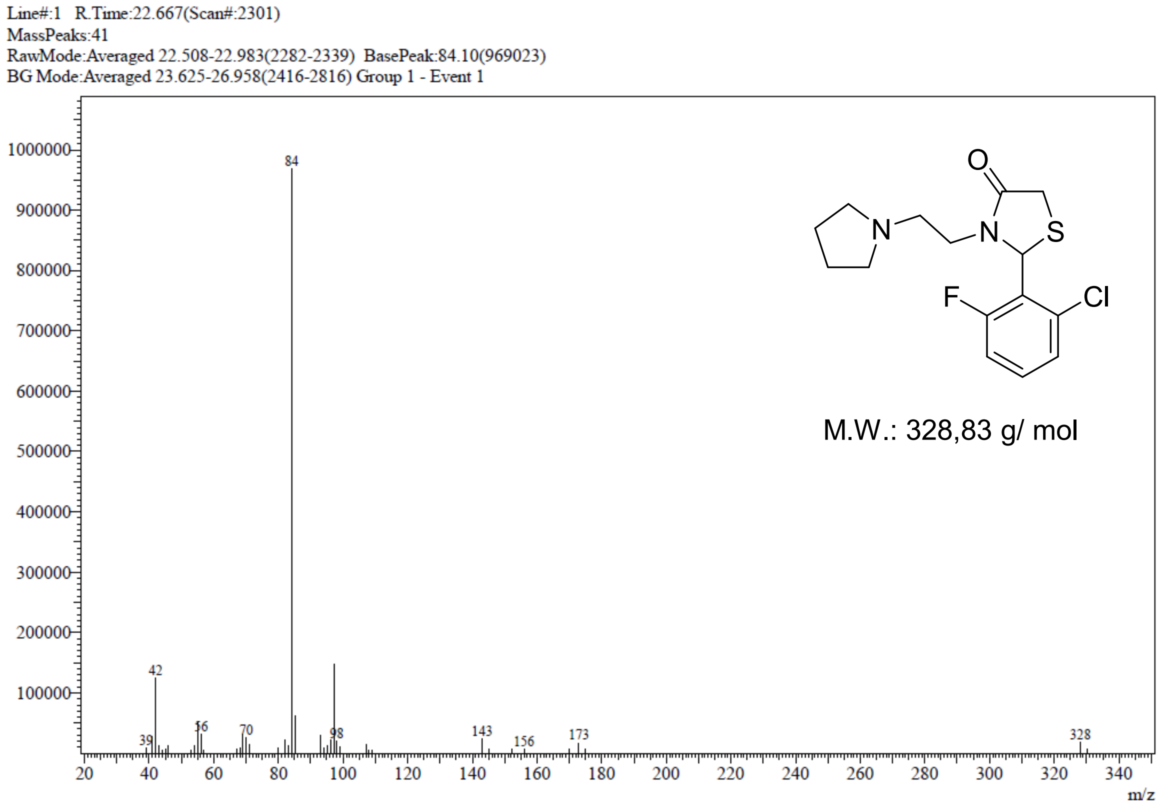

Figure S19: GC/MS of thiazolidin-4-one **5s**.

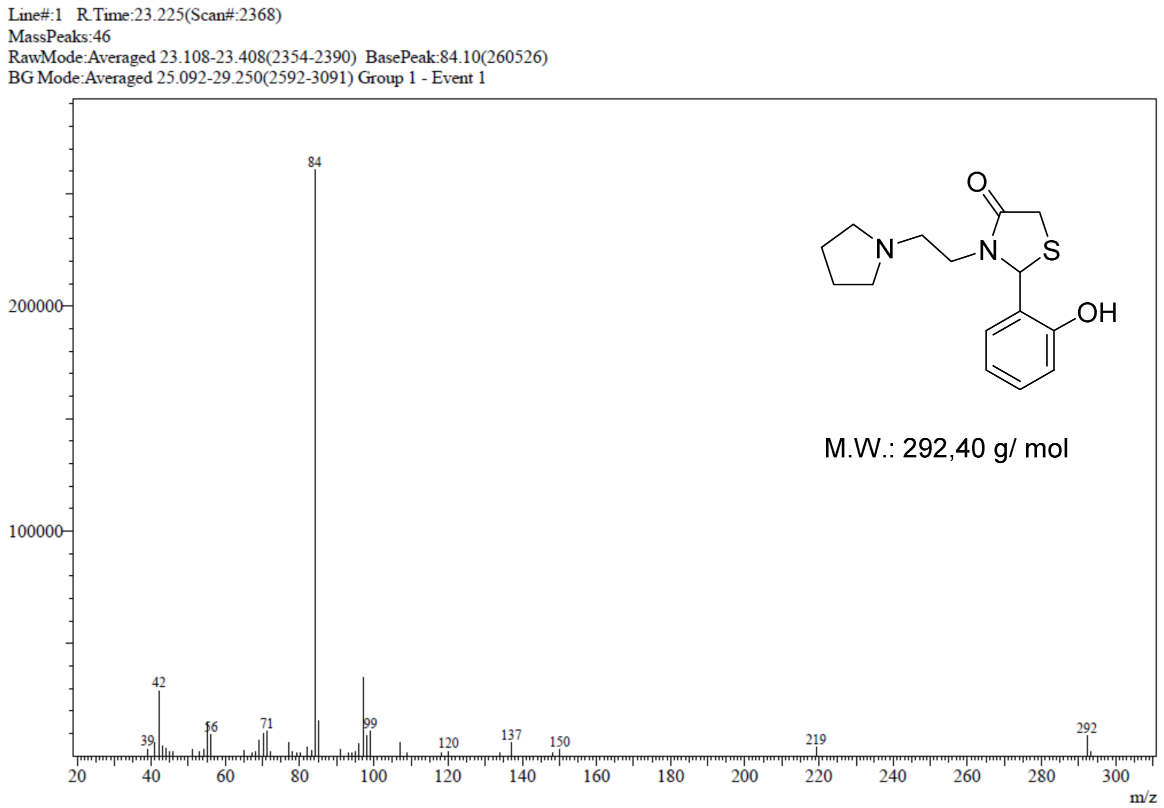

Figure S20: GC/MS of thiazolidin-4-one **5t**.

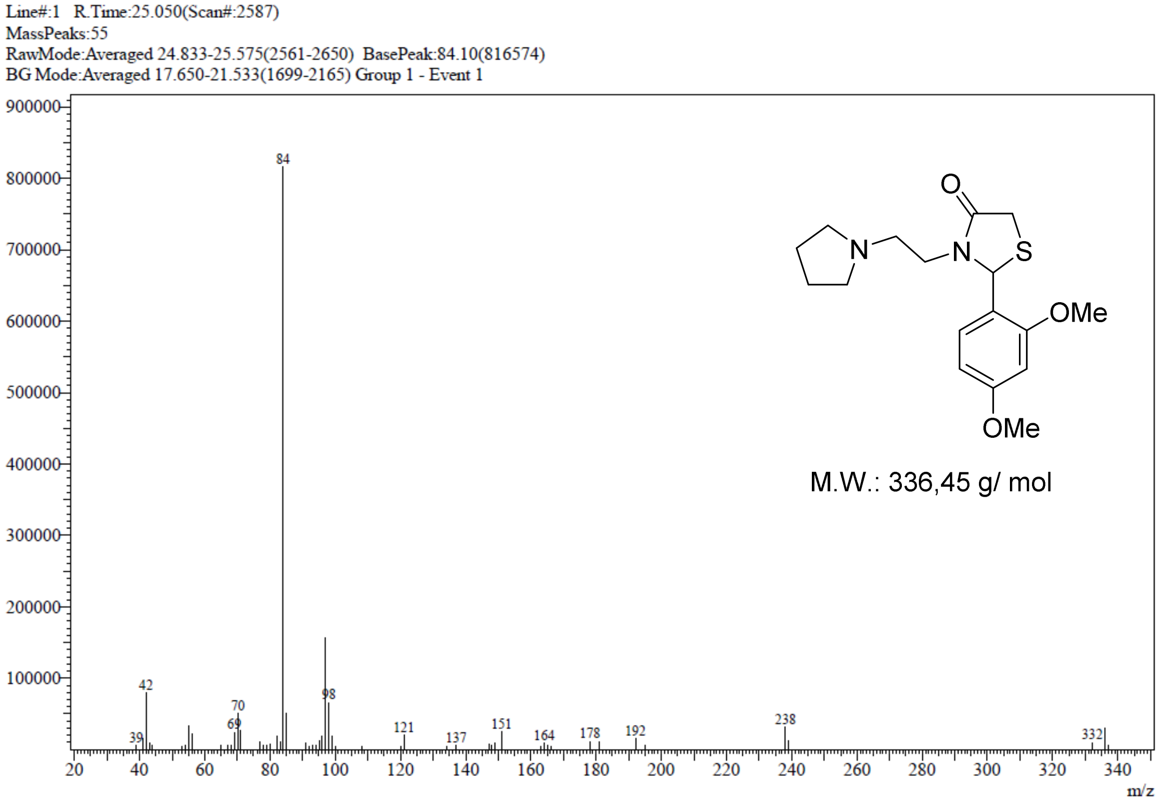

Figure S21: GC/MS of thiazolidin-4-one **5u**.

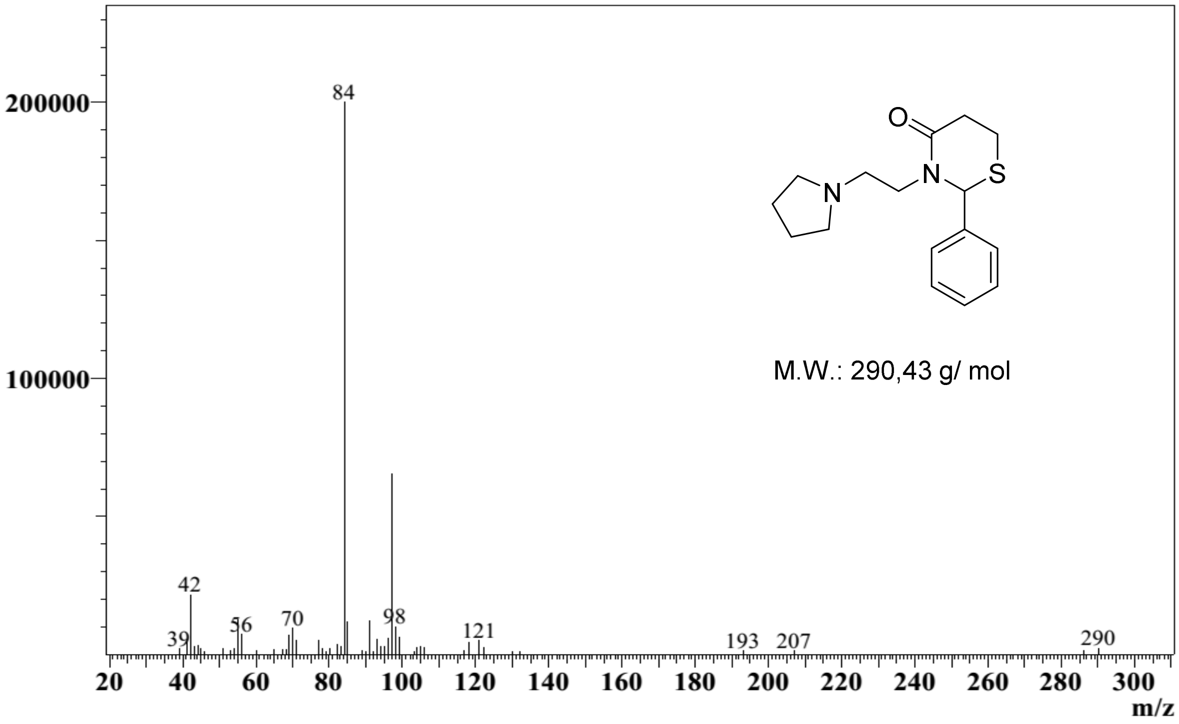

Figure S22: GC/MS of thiazinan-4-one **6a**.

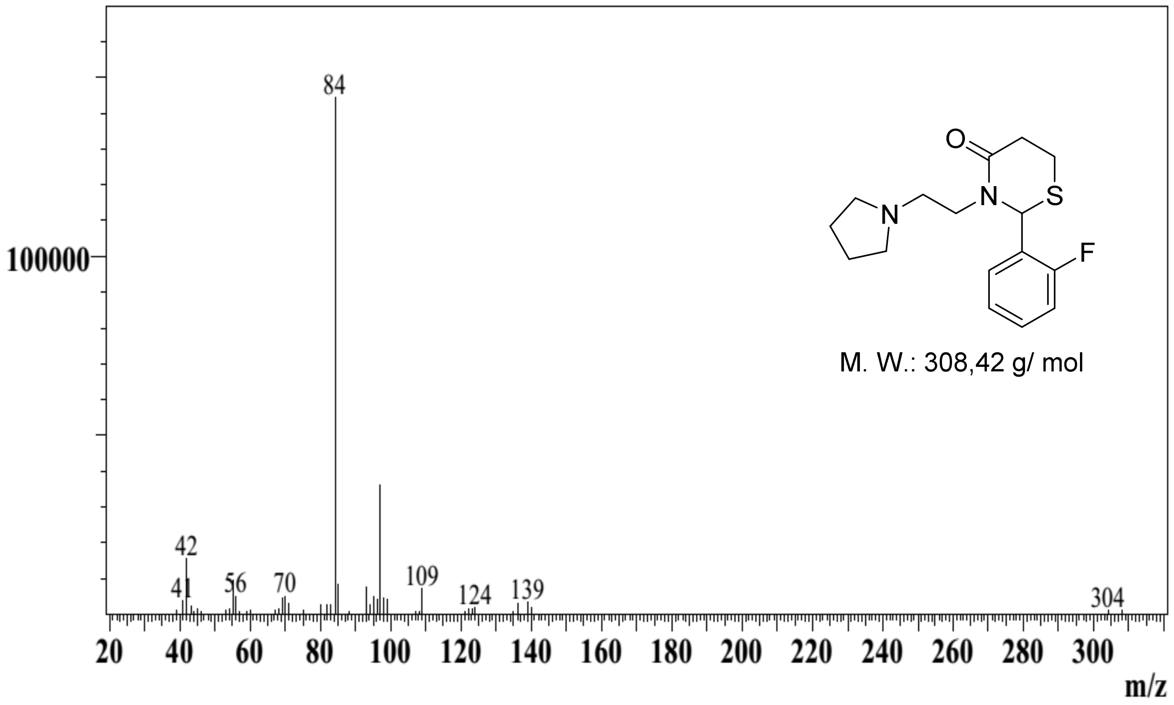

Figure S23: GC/MS of thiazinan-4-one **6b**.

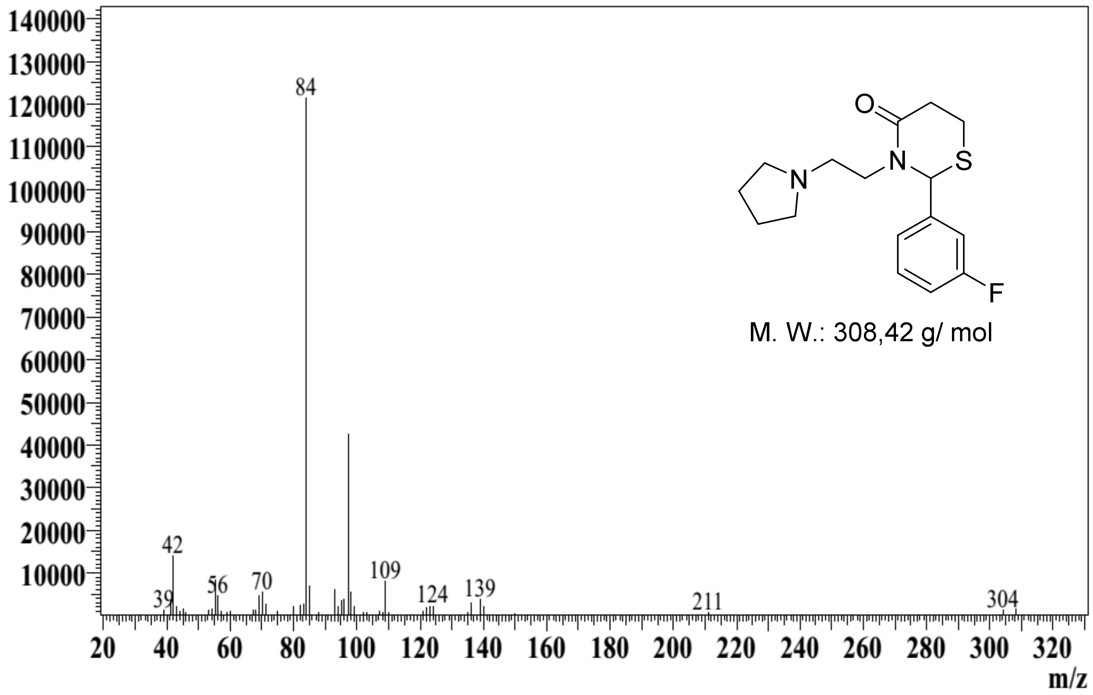

Figure S24: GC/MS of thiazinan-4-one **6c**.

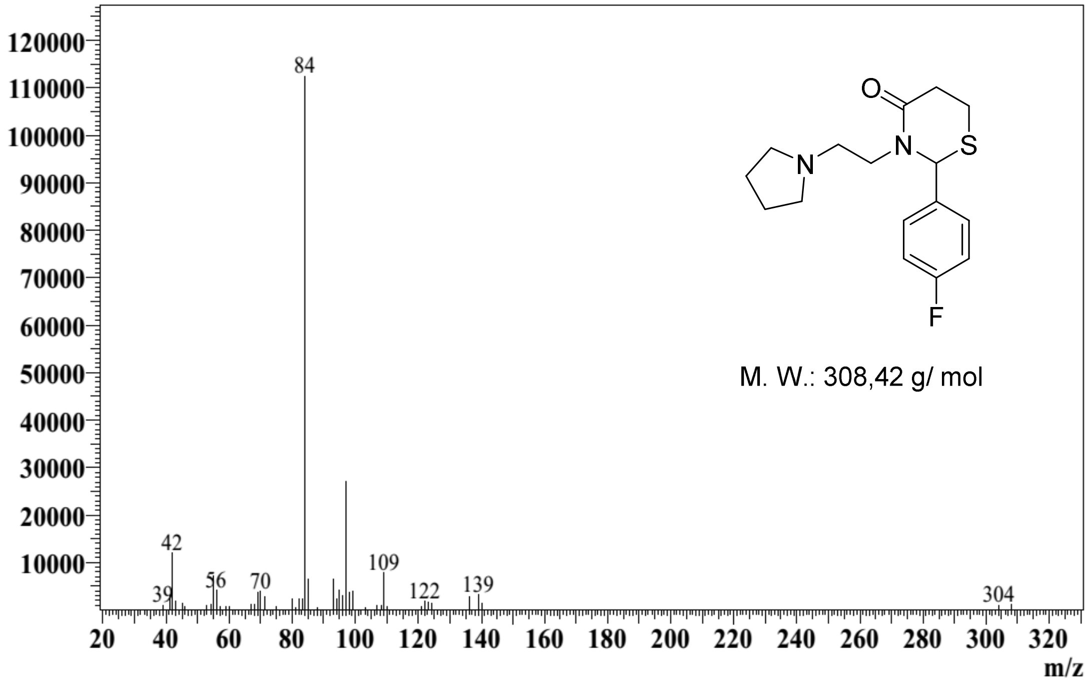

Figure S25: GC/MS of thiazinan-4-one **6d**.

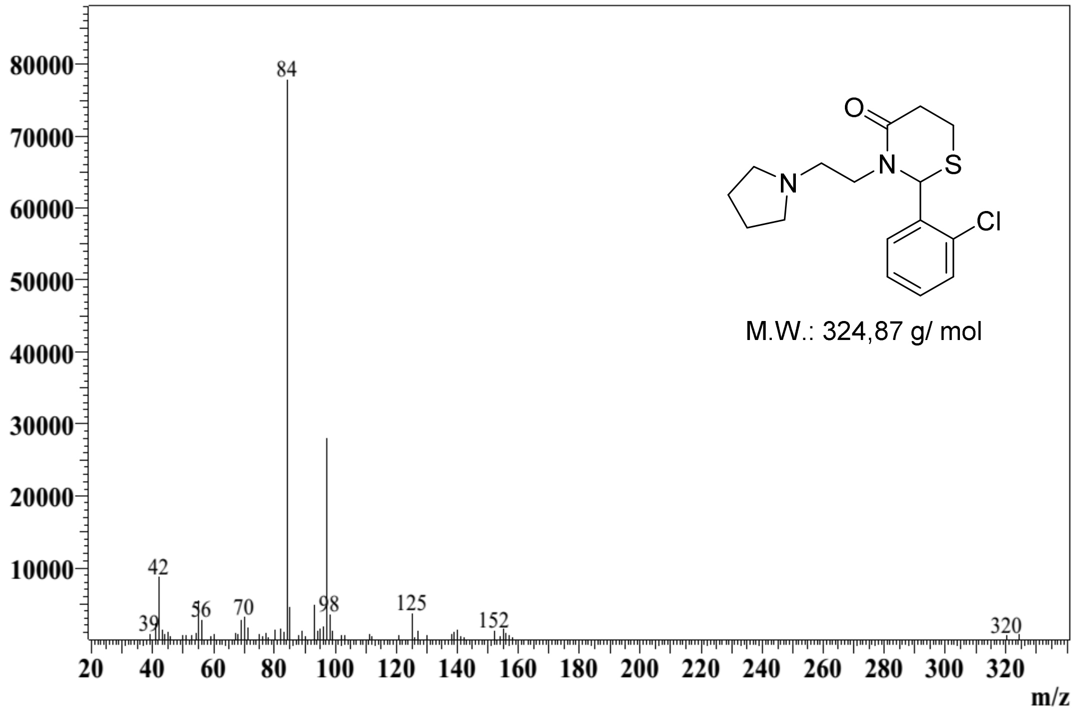

Figure S26: GC/MS of thiazinan-4-one **6e**.

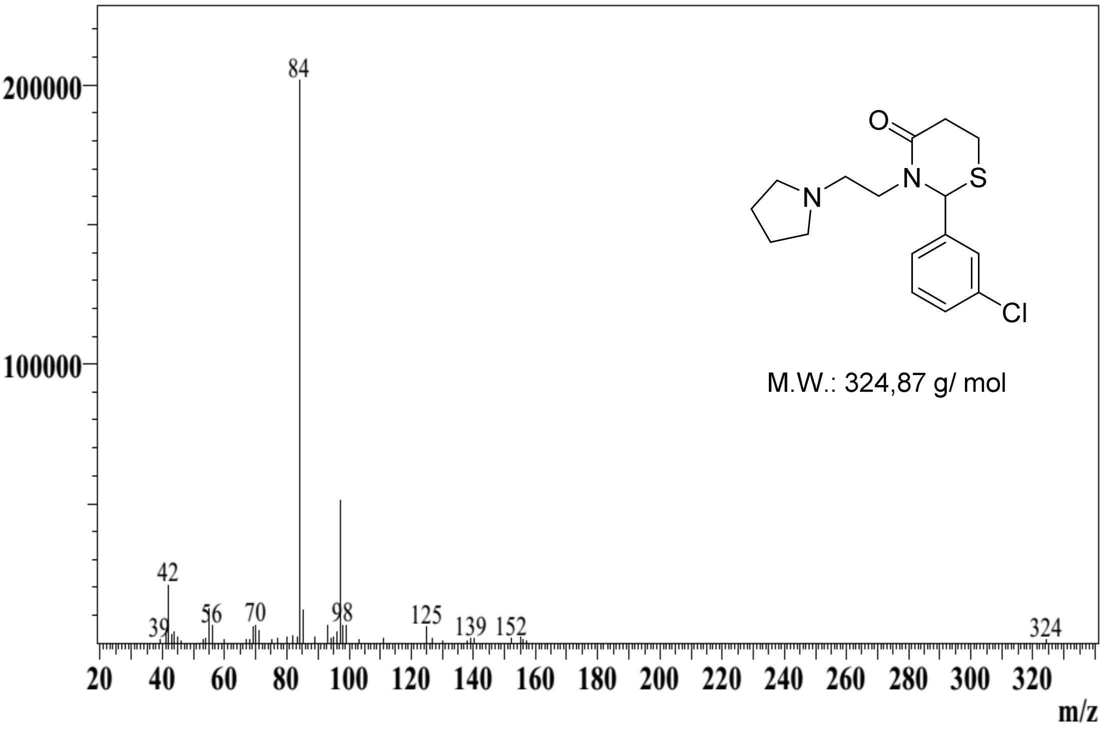

Figure S27: GC/MS of thiazinan-4-one **6f**.

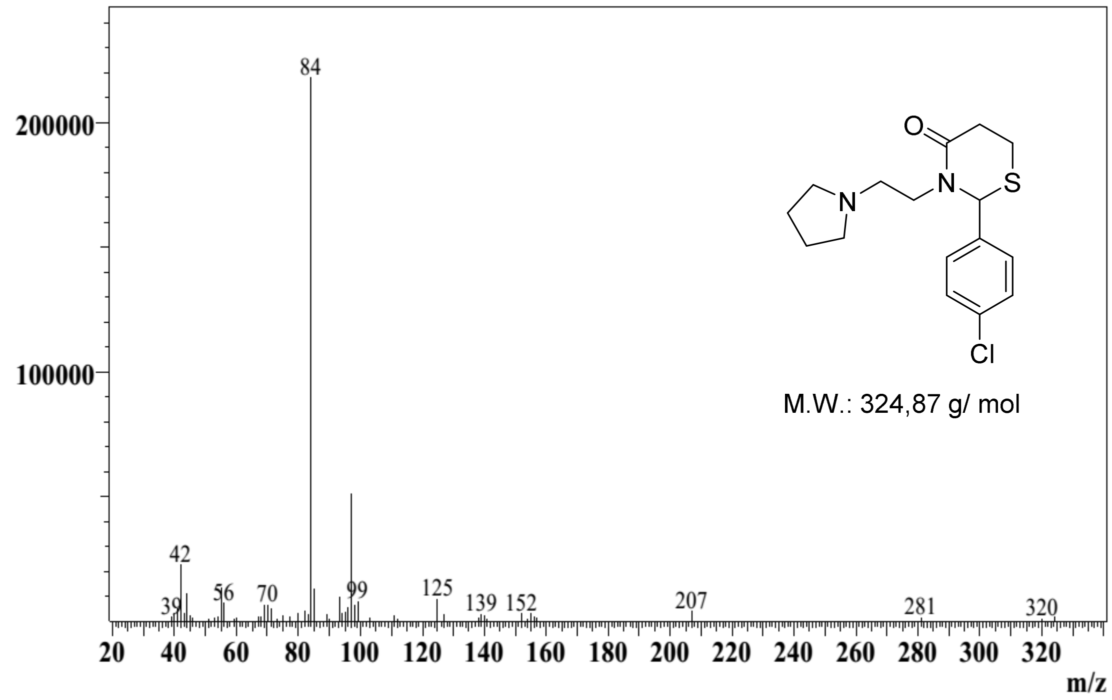

Figure S28: GC/MS of thiazinan-4-one **6g**.

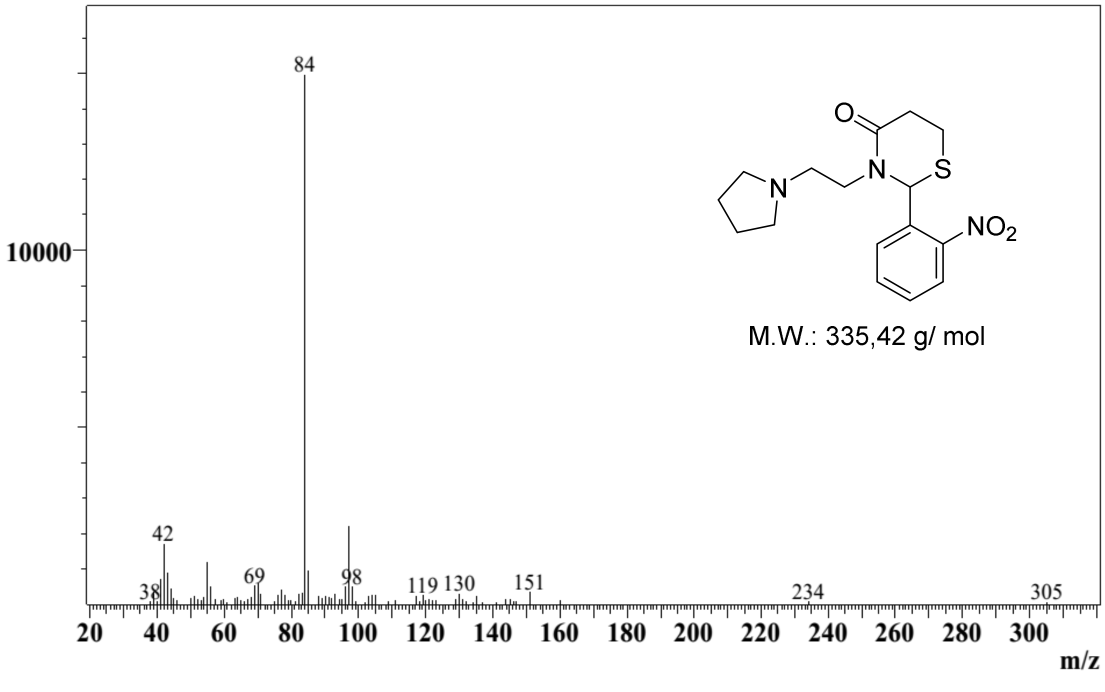

Figure S29: GC/MS of thiazinan-4-one **6h**.

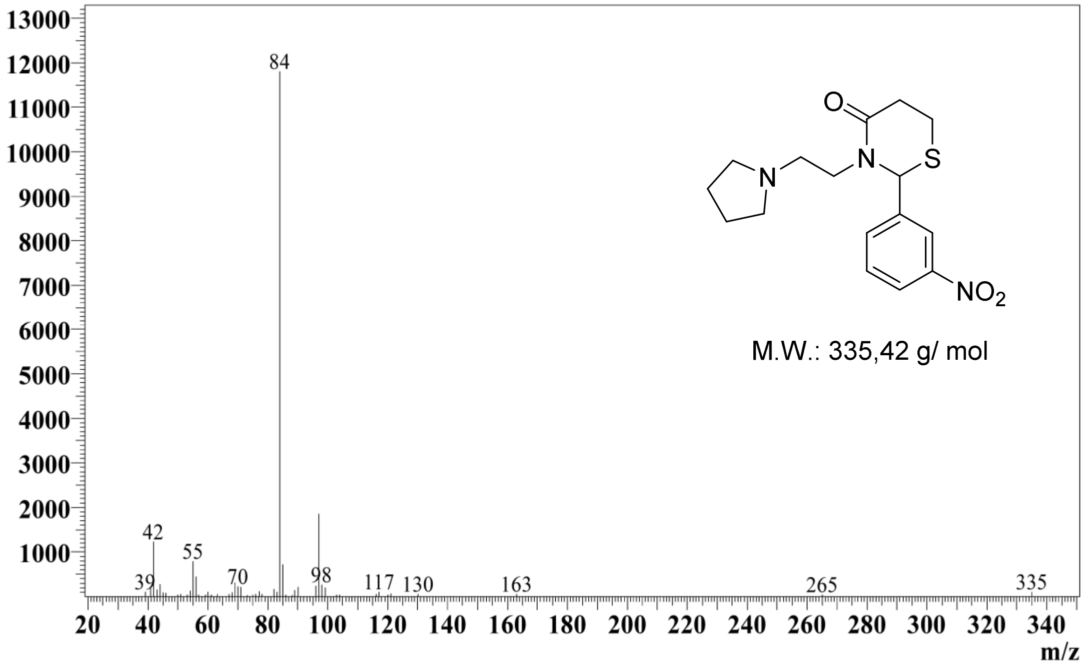

Figure S30: GC/MS of thiazinan-4-one **6i**.

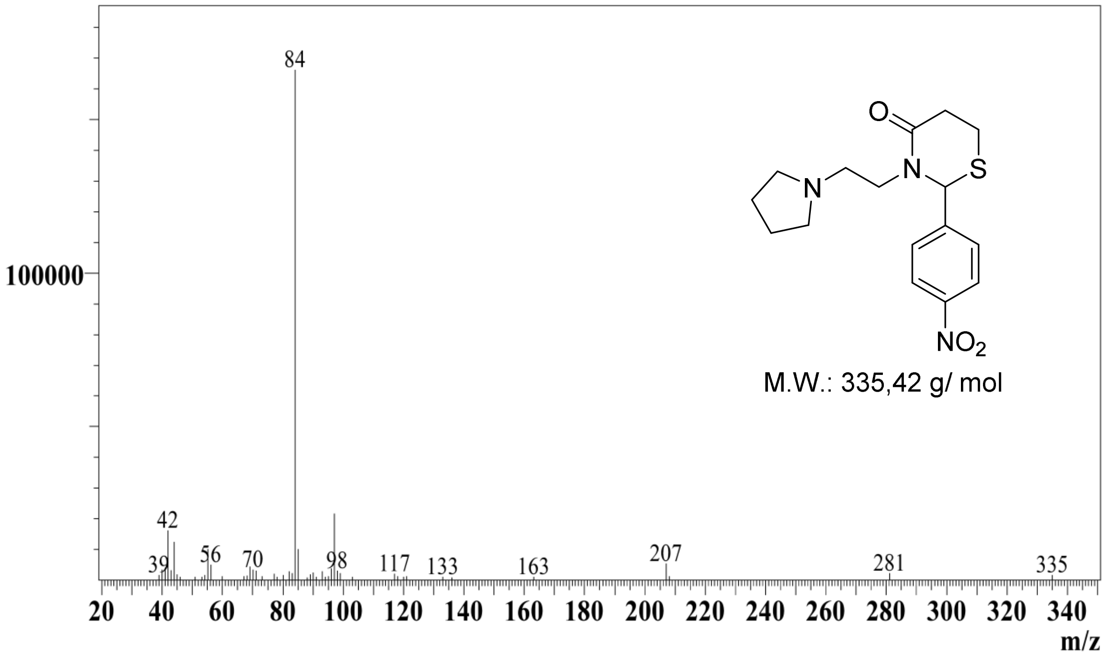

Figure S30: GC/MS of thiazinan-4-one **6j**.

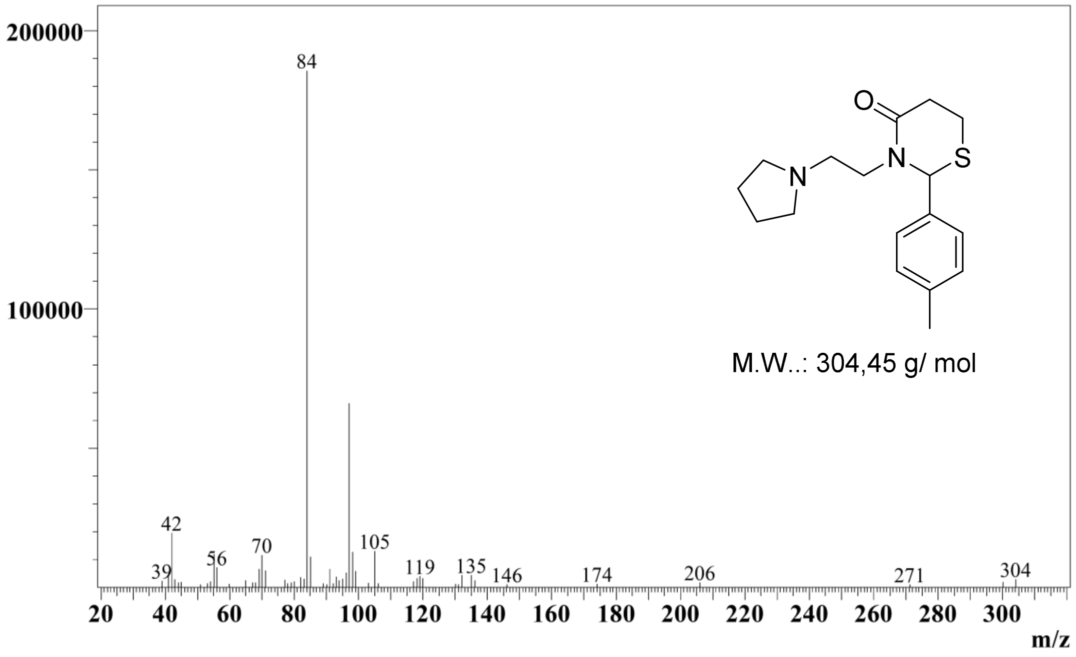

Figure S31: GC/MS of thiazinan-4-one **6k**.

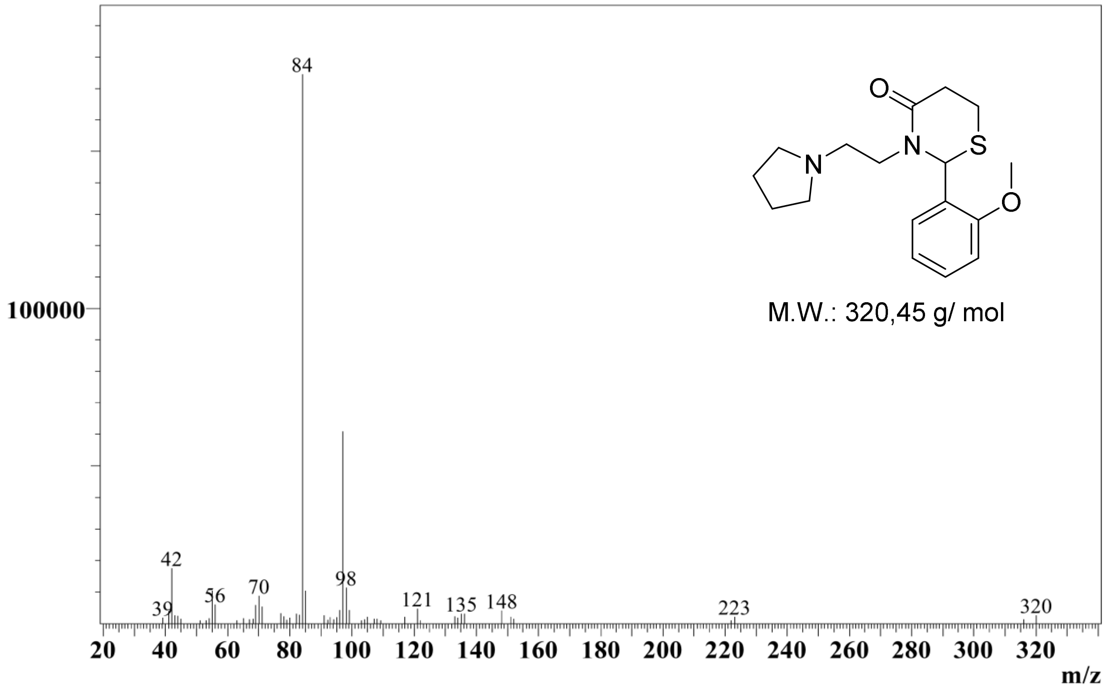

Figure S32: GC/MS of thiazinan-4-one **6l**.

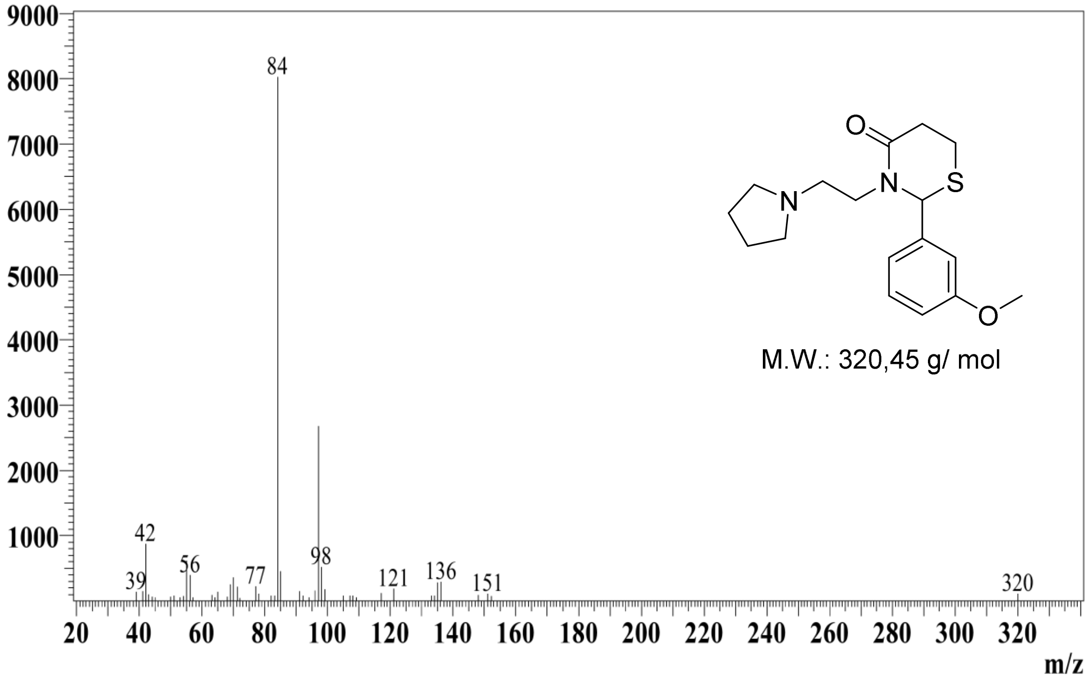

Figure S33: GC/MS of thiazinan-4-one **6m**.

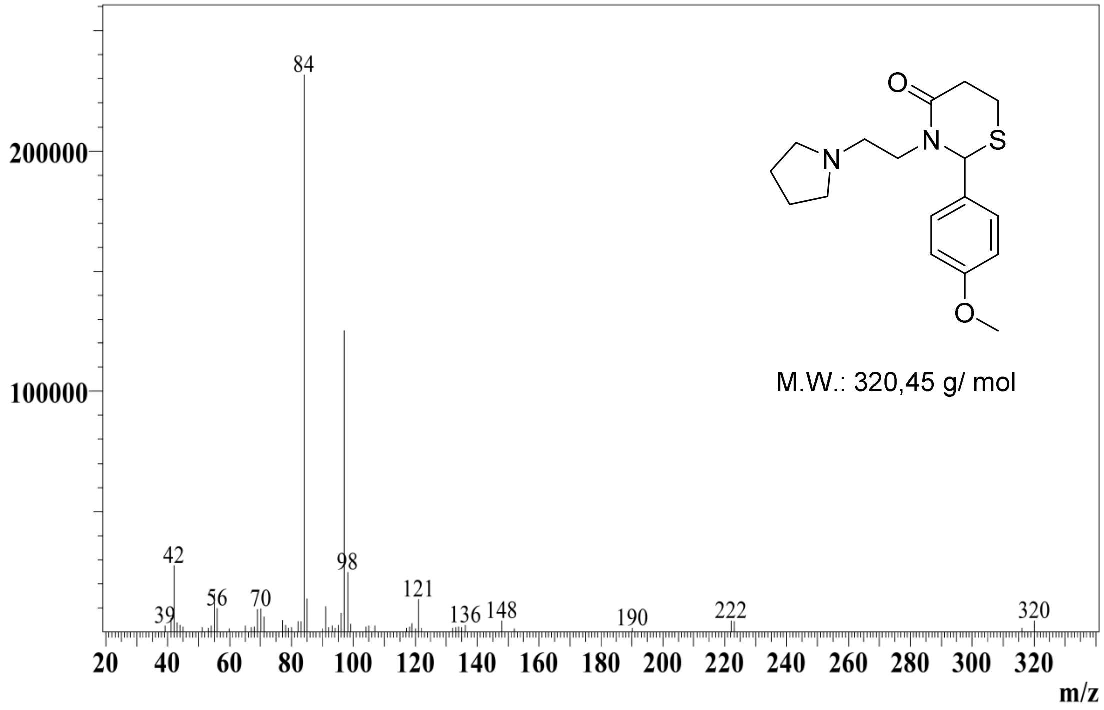

Figure S34: GC/MS of thiazinan-4-one **6n**.

.

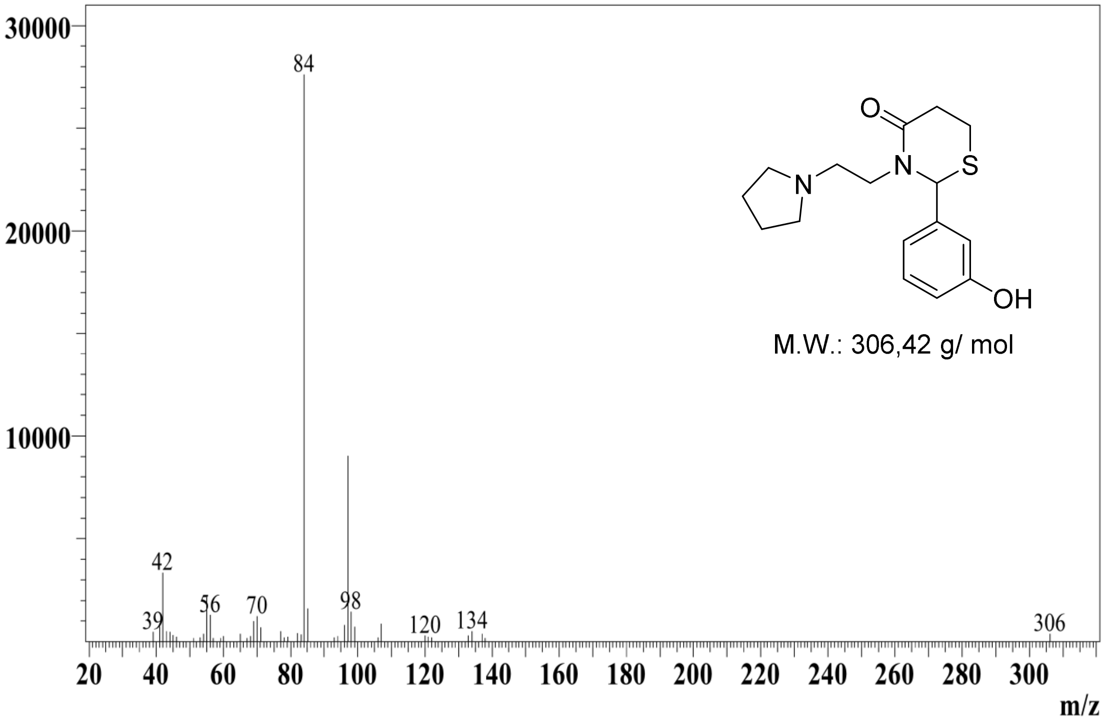

Figure S35: GC/MS of thiazinan-4-one **6o**.

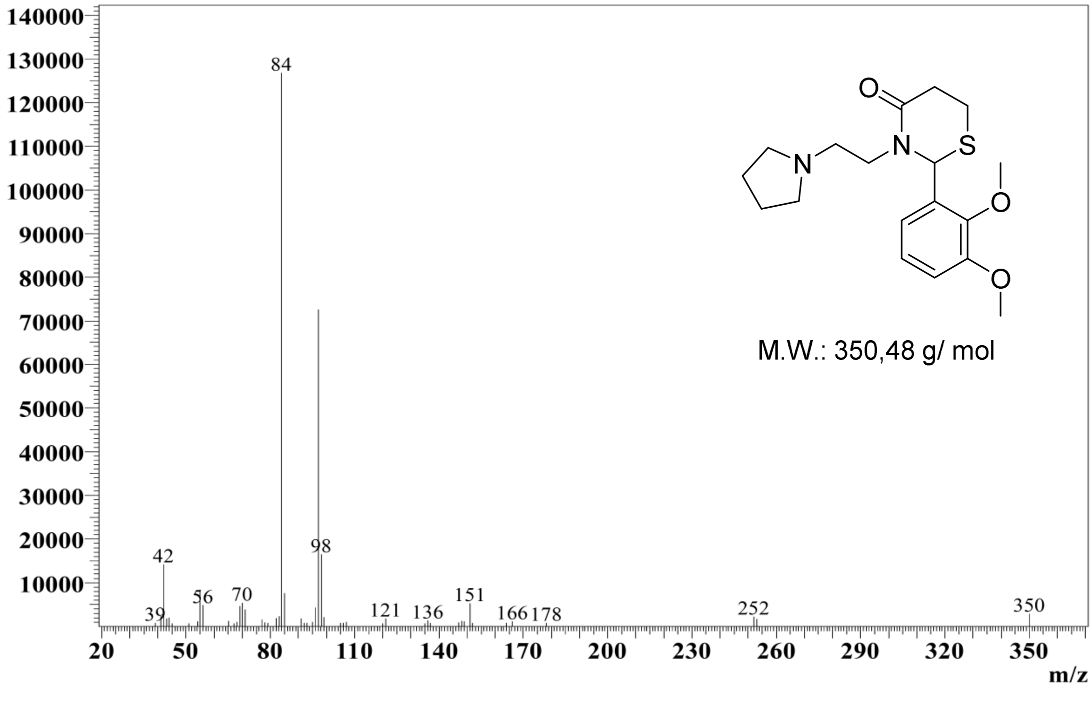

Figure S36: GC/MS of thiazinan-4-one **6p**.

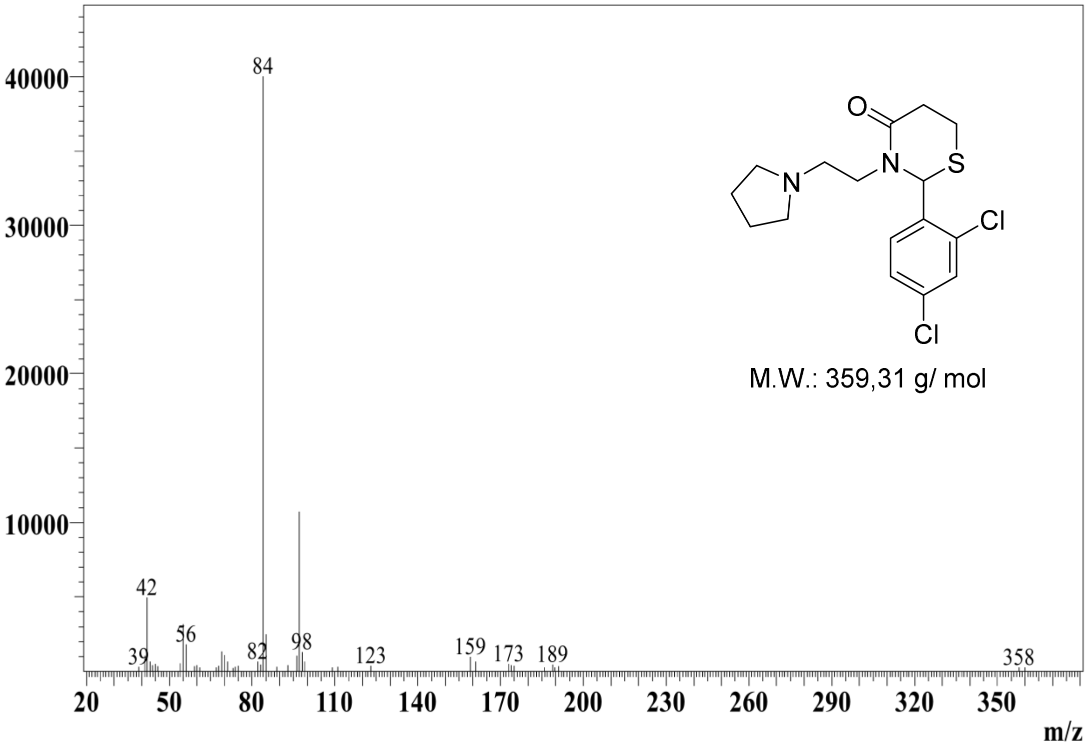

Figure S37: GC/MS of thiazinan-4-one **6q**.

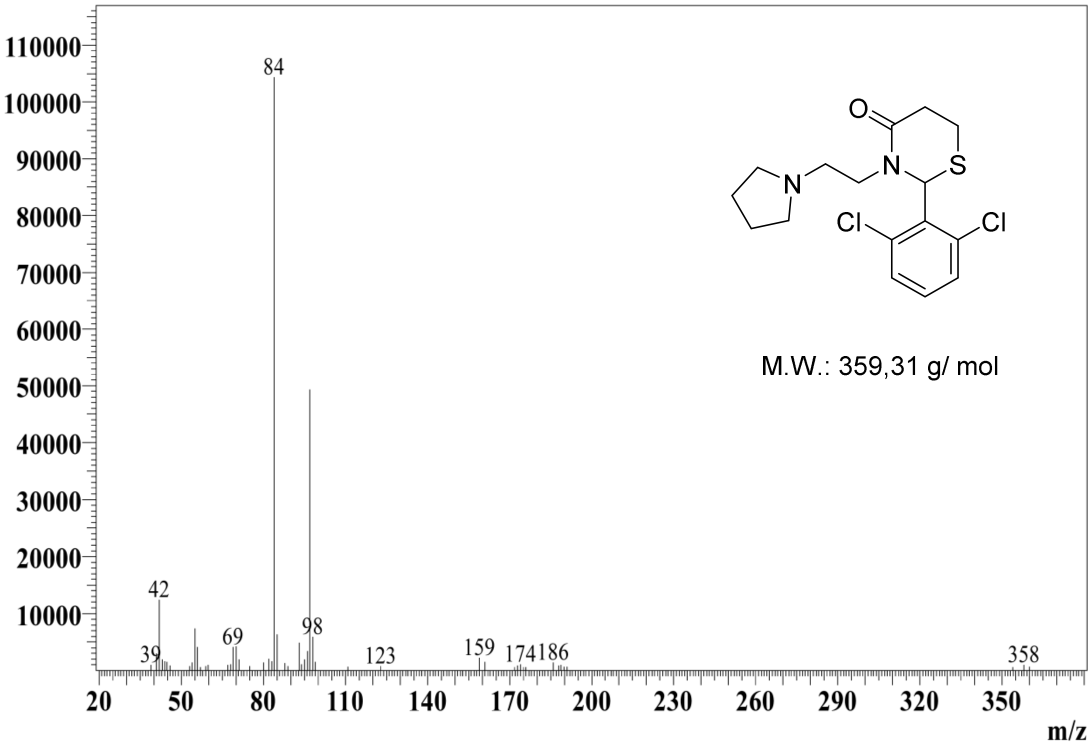

Figure S38: GC/MS of thiazinan-4-one **6r**.

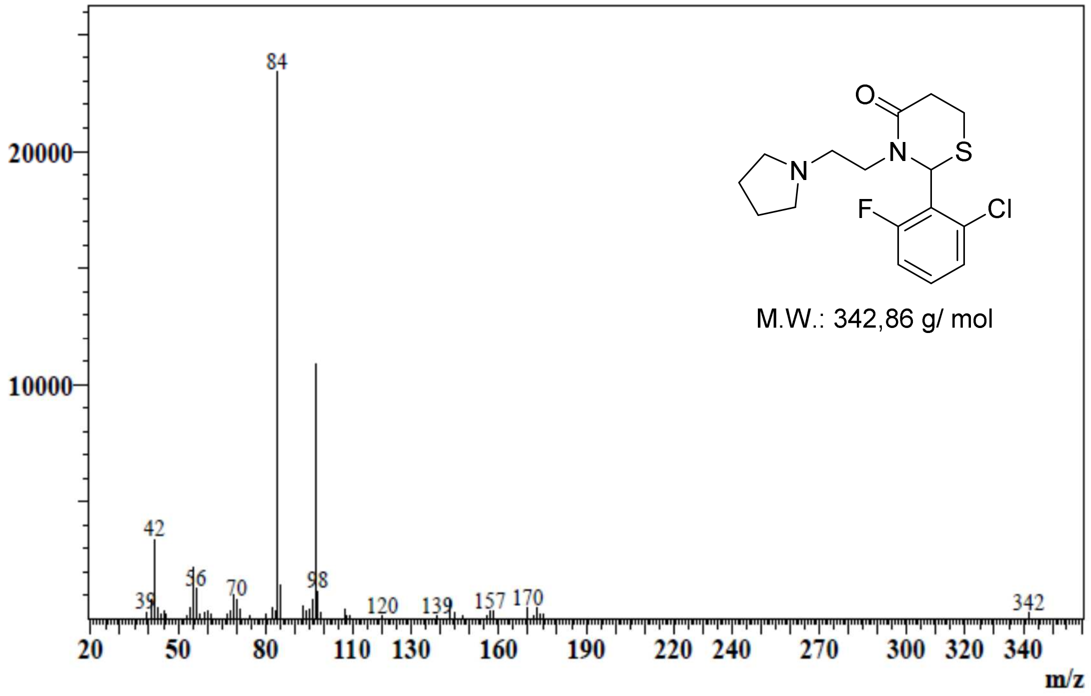

Figure S39: GC/MS of thiazinan-4-one **6s**.

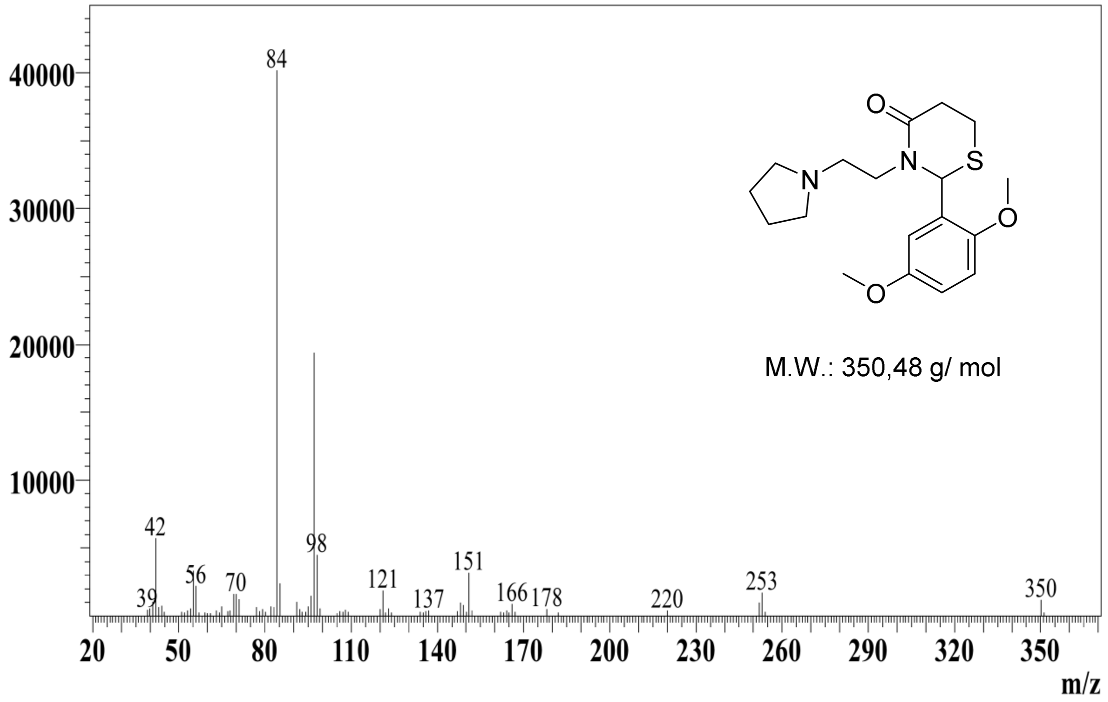

Figure S40: GC/MS of thiazinan-4-one **6v**.

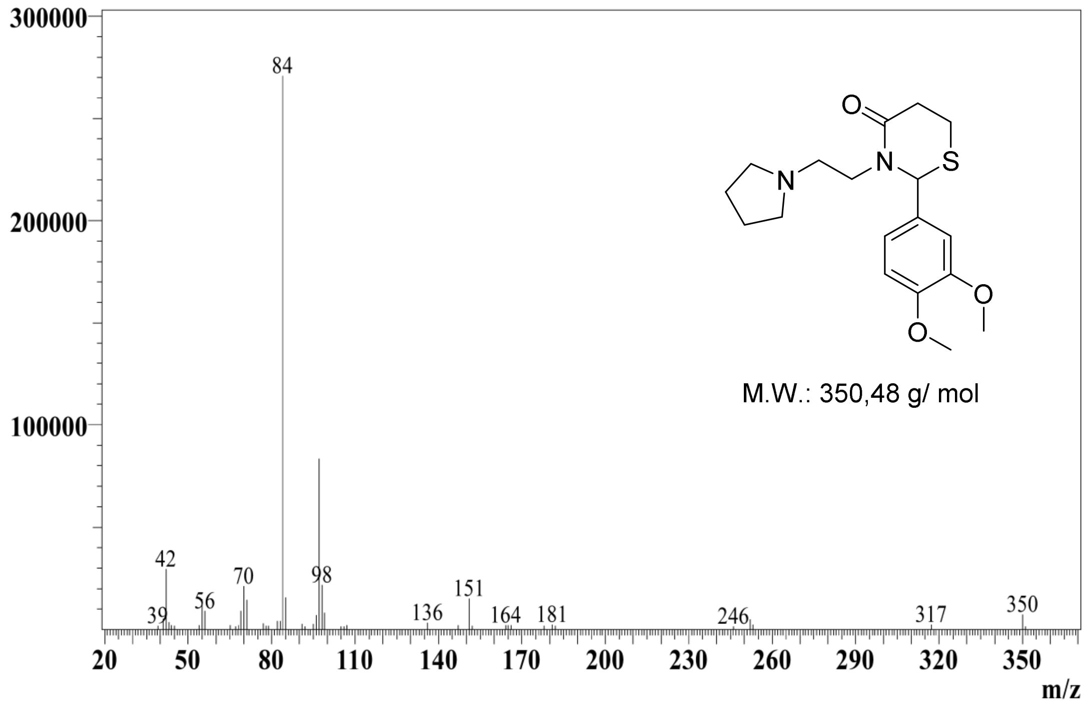

Figure S41: GC/MS of thiazinan-4-one **5x**.

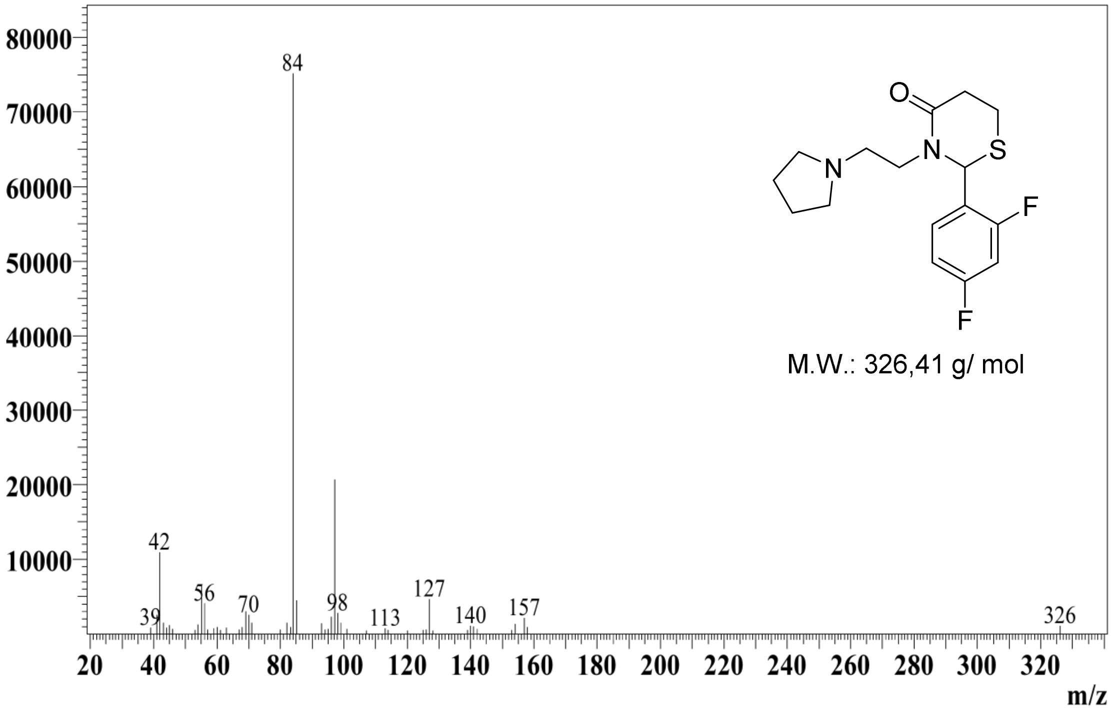

Figure S42: GC/MS of thiazinan-4-one **5w.**

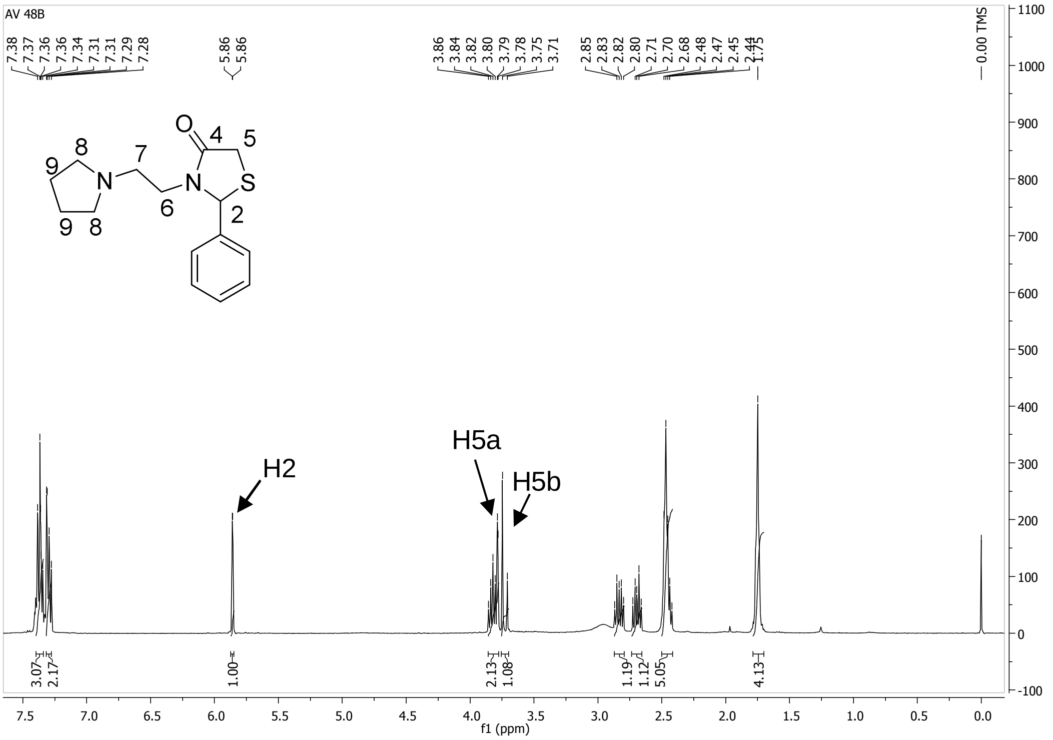

Figure S43: NMR ^{1}H spectrum of thiazolidin-4-one **5a**.

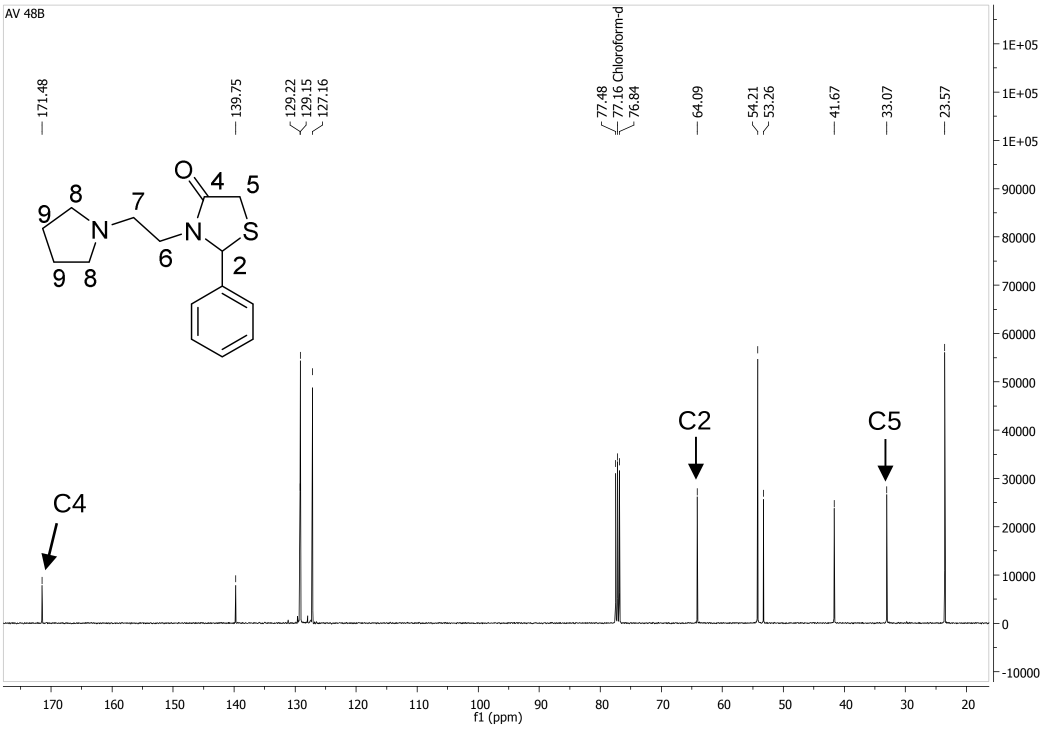

Figure S44: NMR ^{13}C spectrum of thiazolidin-4-one **5a**.

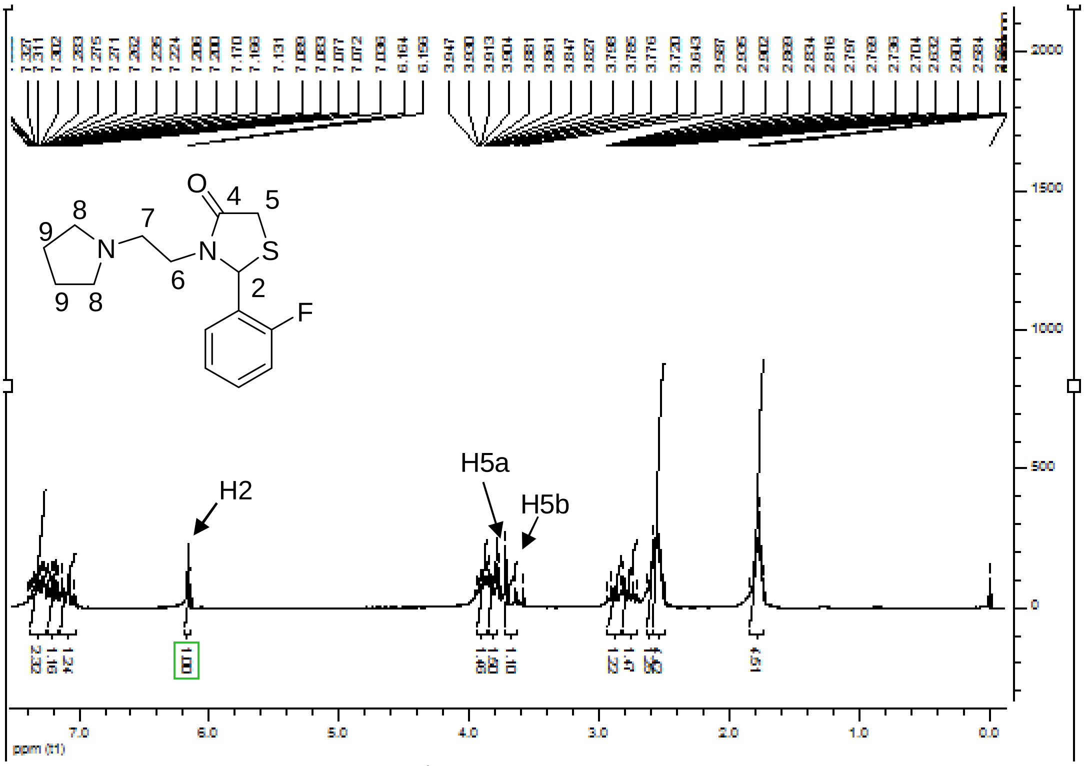

Figure S45: NMR ^{1}H spectrum of thiazolidin-4-one **5b**.

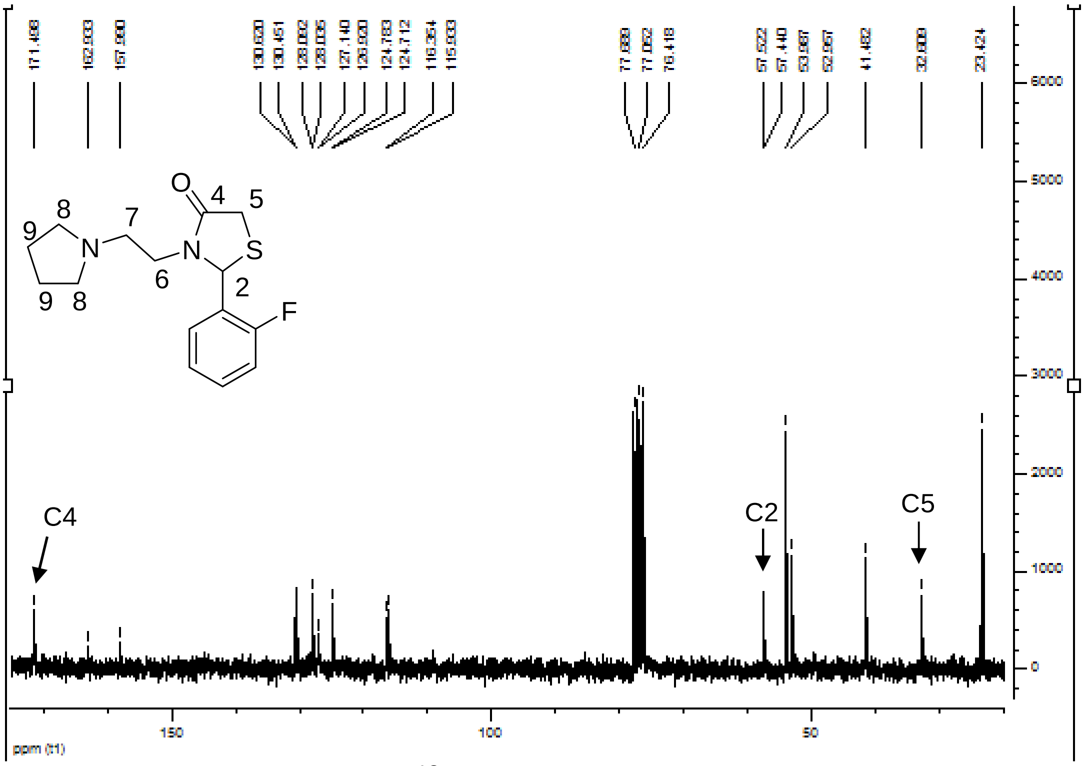

Figure S46: NMR ^{13}C spectrum of thiazolidin-4-one **5b**.

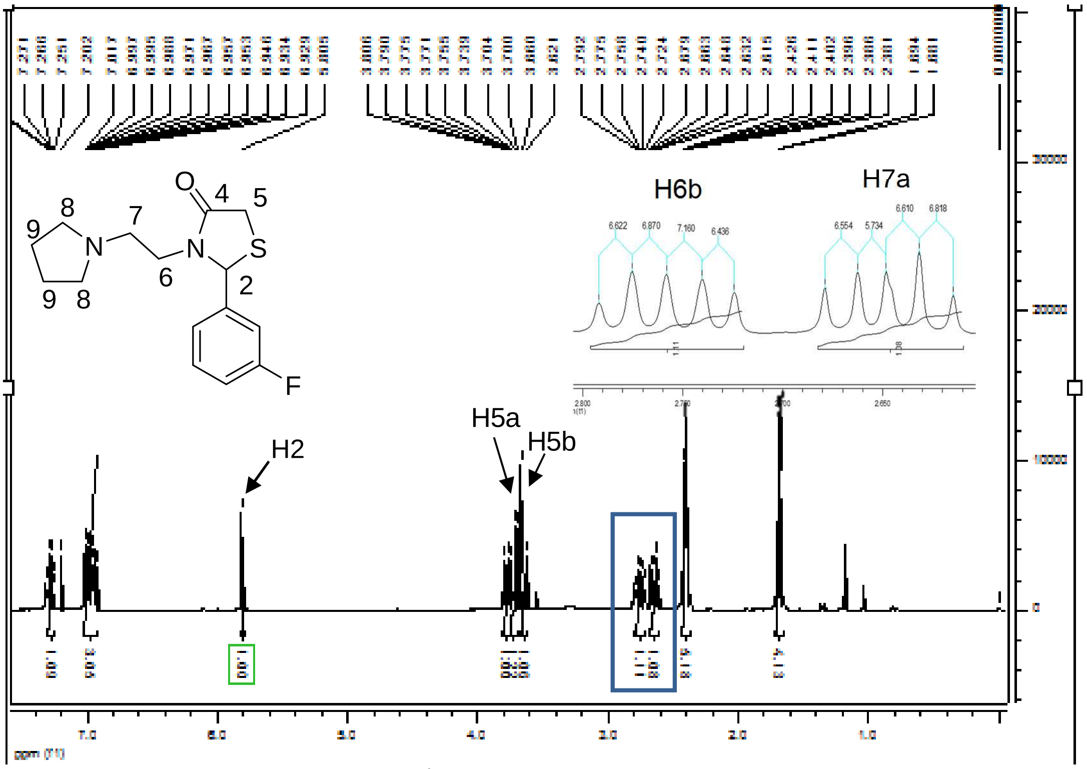

Figure S47: NMR ^{1}H spectrum of thiazolidin-4-one **5c**.

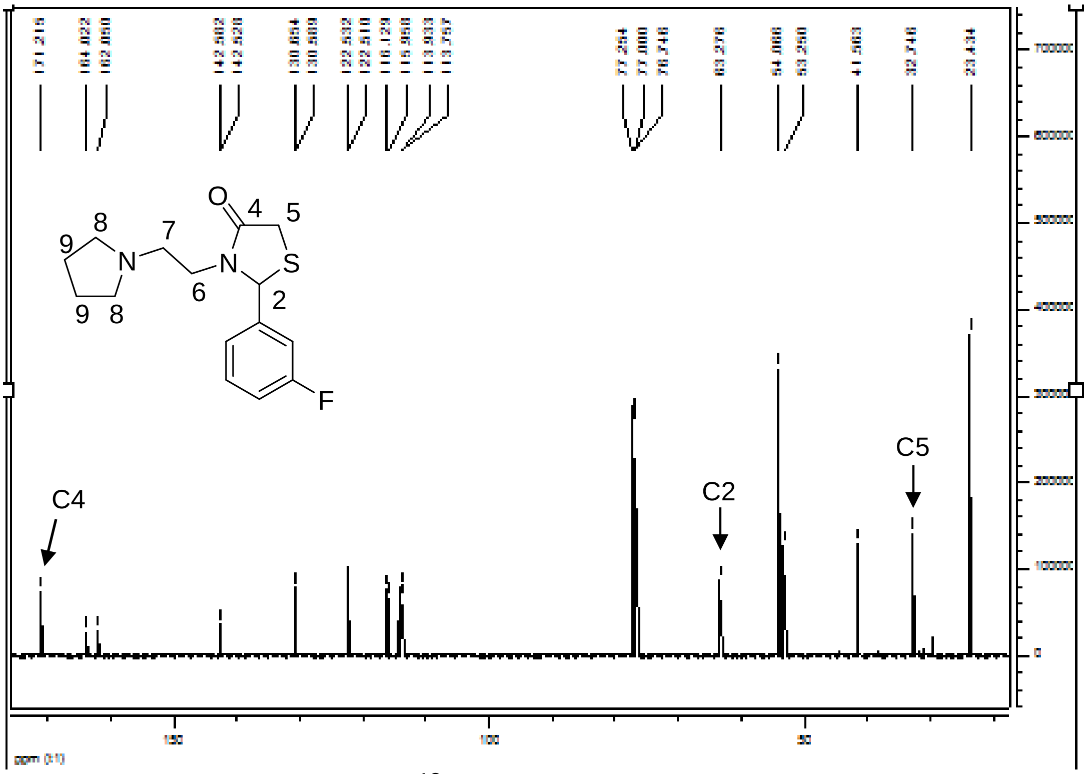

Figure S48: NMR ^{13}C spectrum of thiazolidin-4-one **5c**.

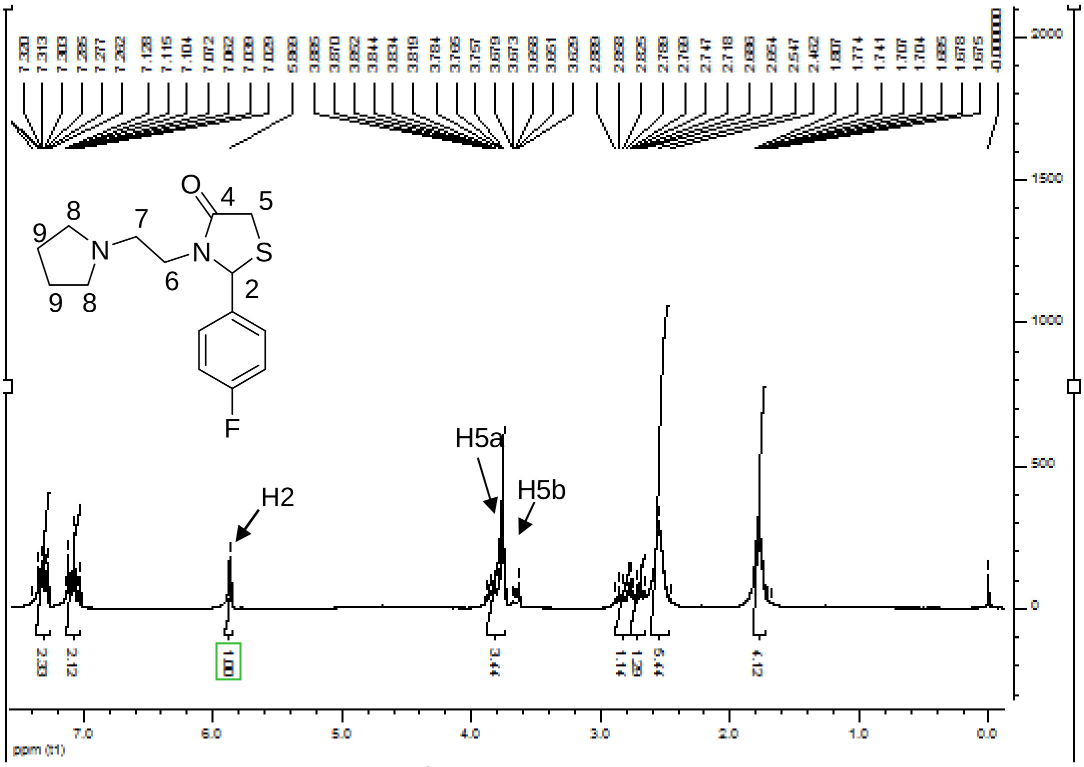

Figure S49: NMR ^{1}H spectrum of thiazolidin-4-one **5d**.

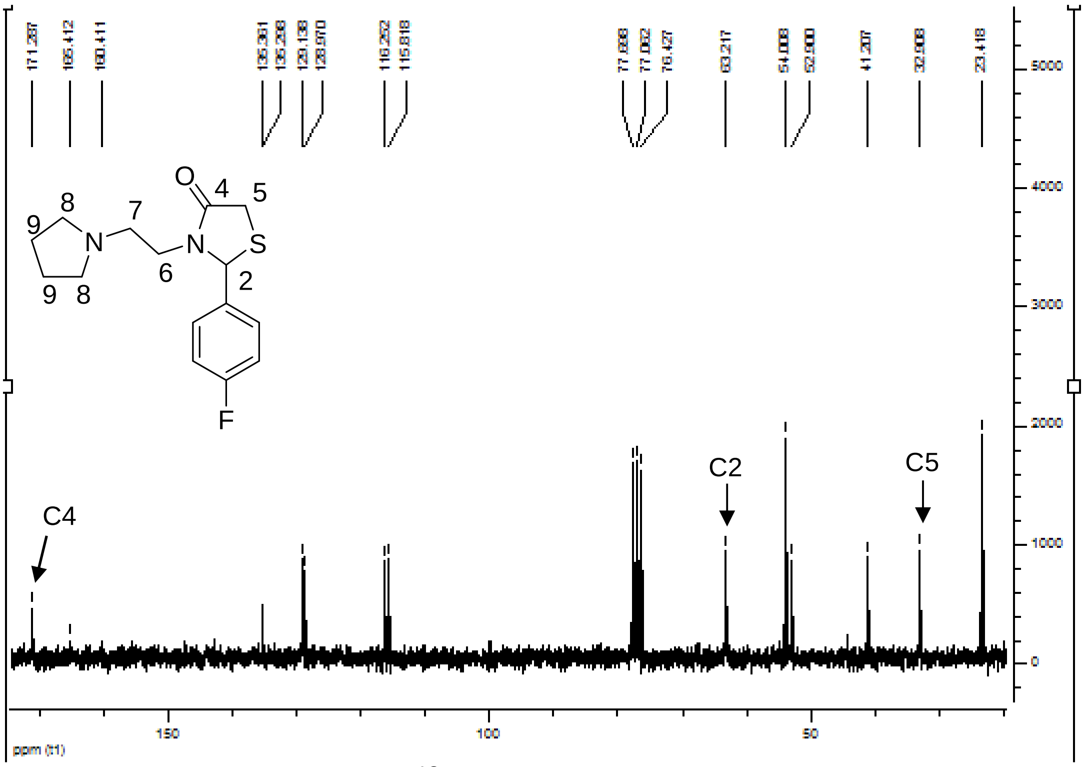

Figure S50: NMR ^{13}C spectrum of thiazolidin-4-one **5d**.

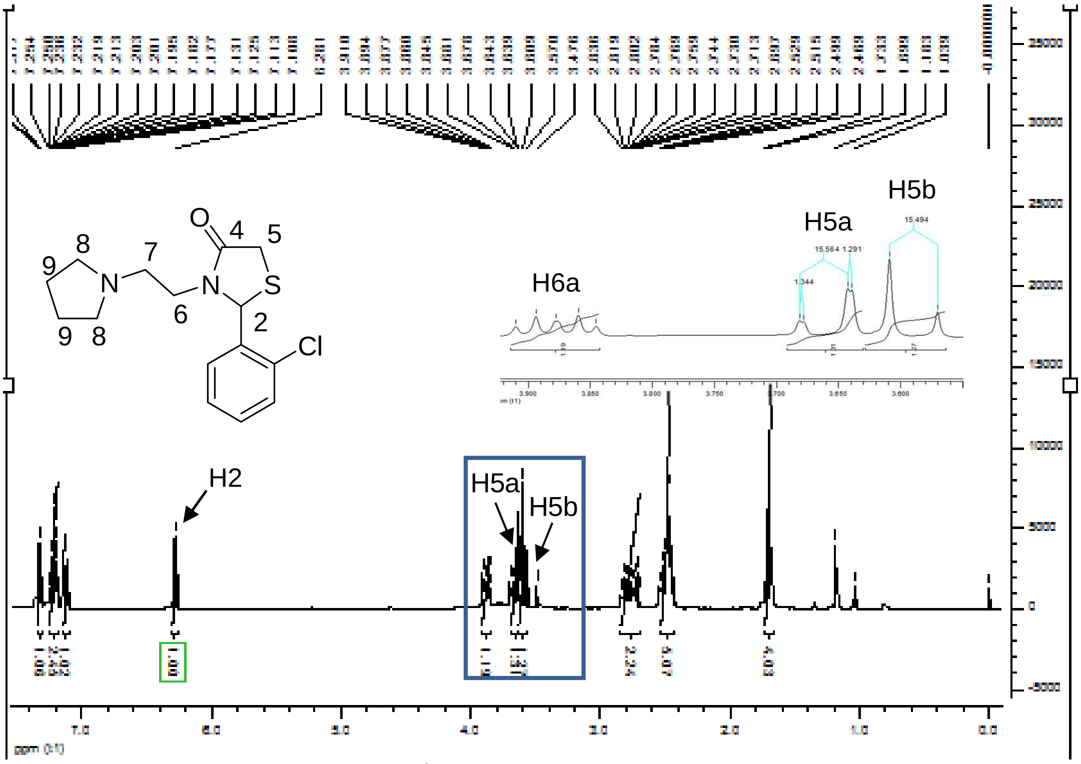

Figure S51: NMR ^{1}H spectrum of thiazolidin-4-one **5e**.

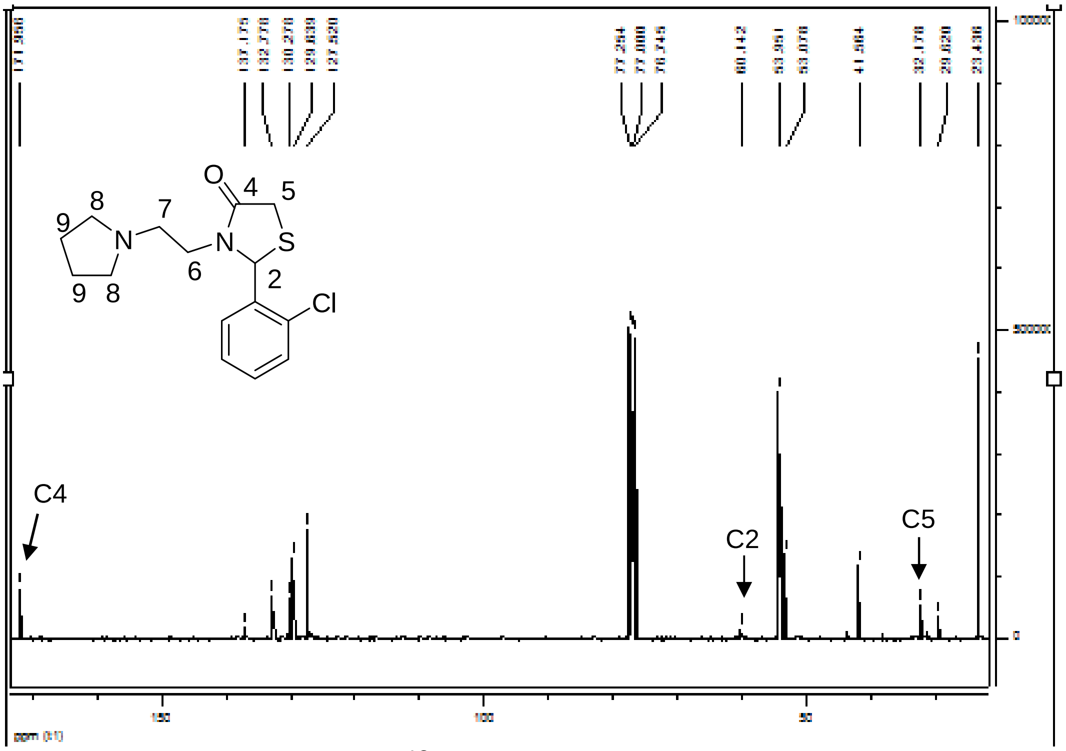

Figure S52: NMR ^{13}C spectrum of thiazolidin-4-one **5e**.

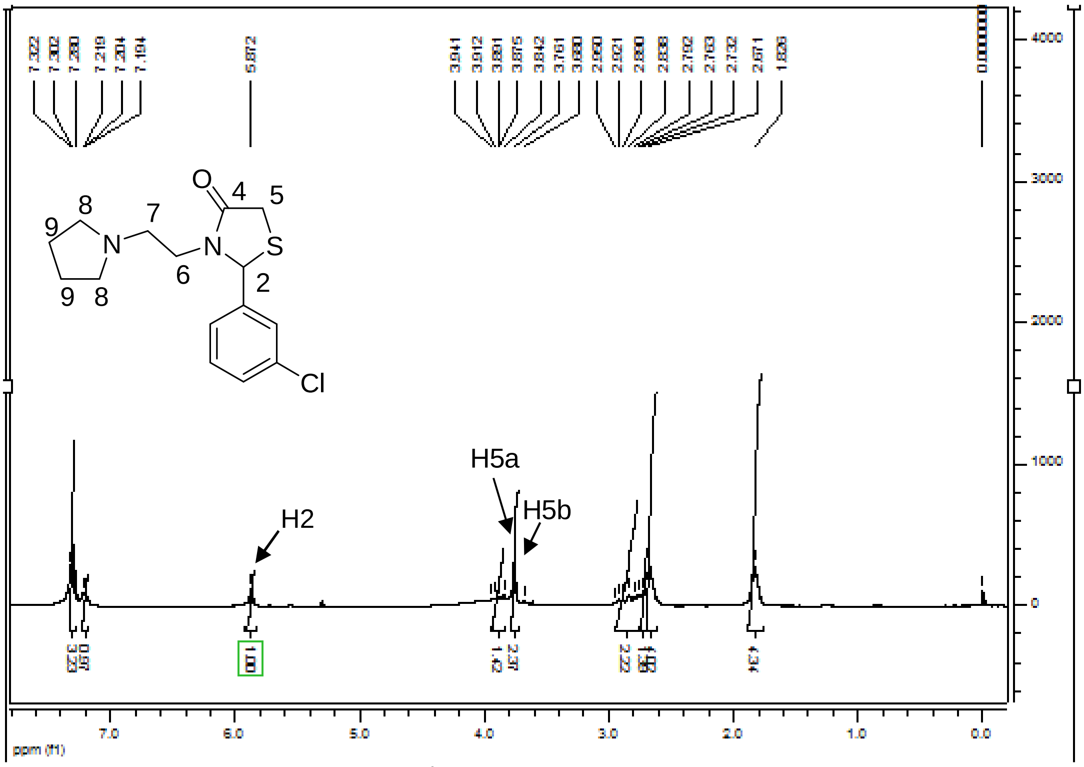

Figure S53: NMR ^{1}H spectrum of thiazolidin-4-one **5f**.

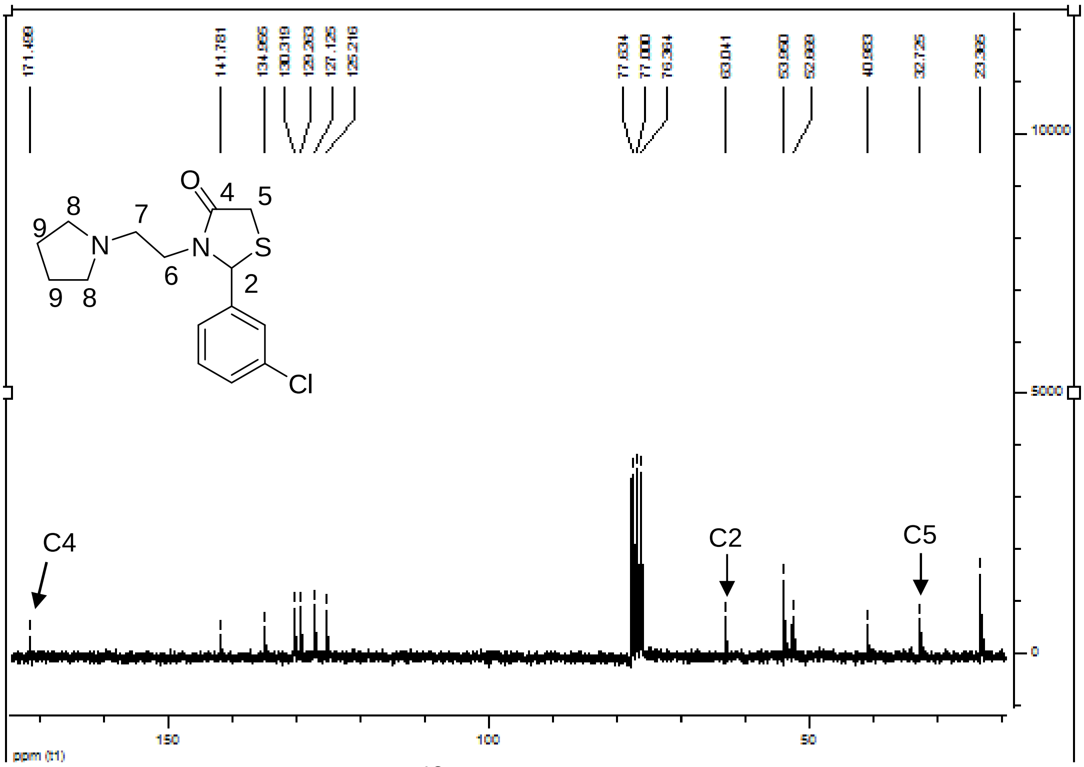

Figure S54: NMR ^{13}C spectrum of thiazolidin-4-one **5f**.

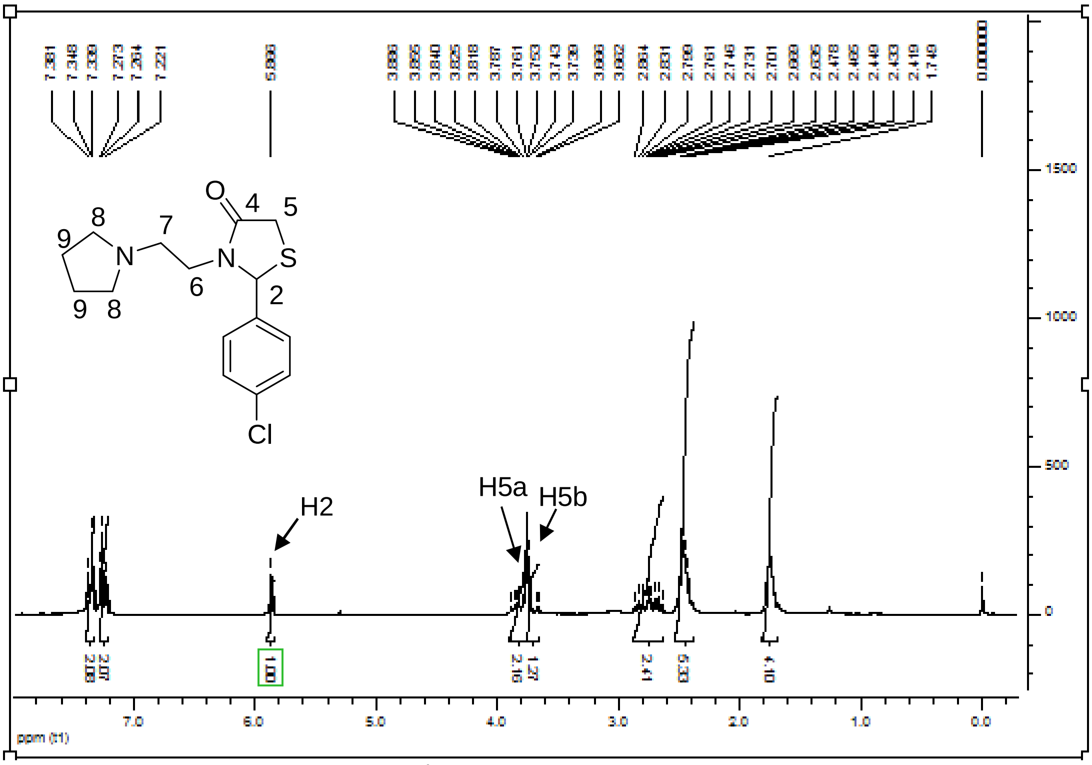

Figure S55: NMR ^{1}H spectrum of thiazolidin-4-one **5g**.

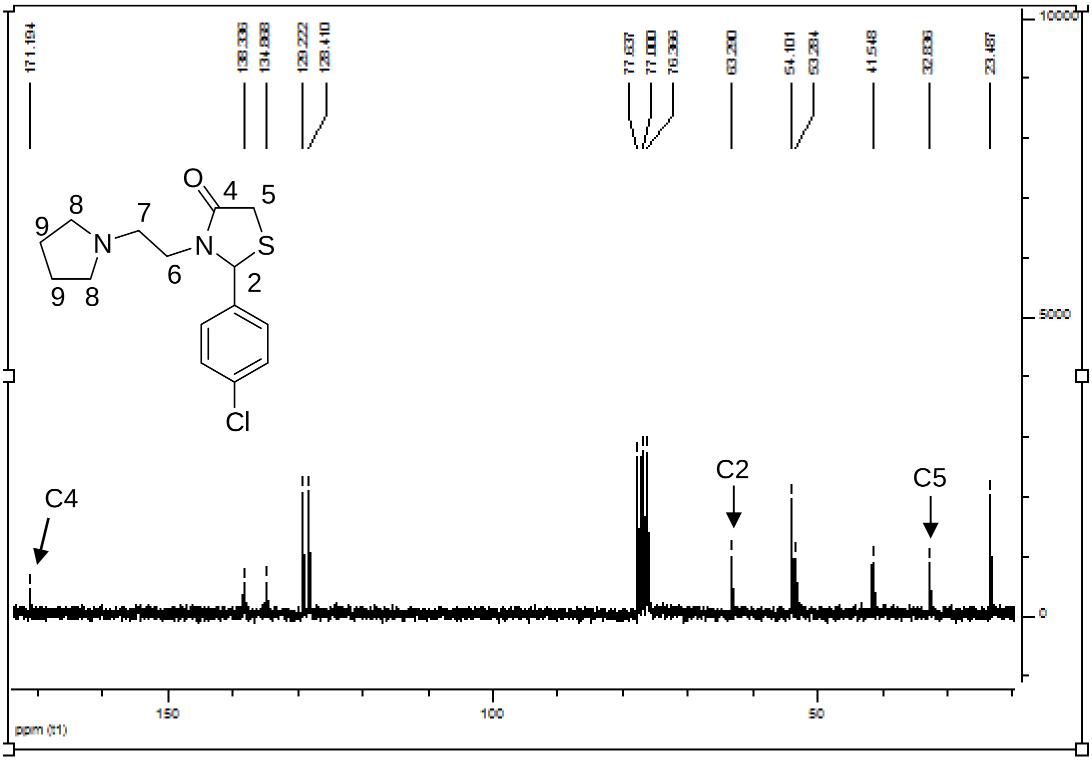

Figure S56: NMR ^{13}C spectrum of thiazolidin-4-one **5g**.

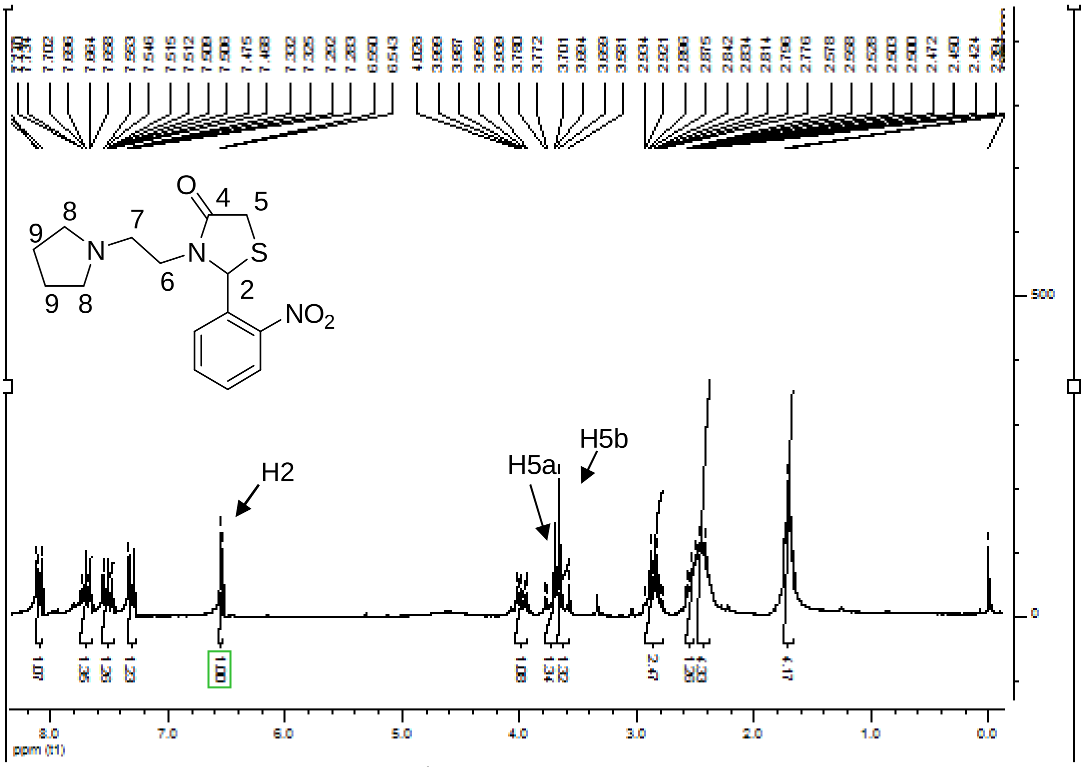

Figure S57: NMR ^{1}H spectrum of thiazolidin-4-one **5h**.

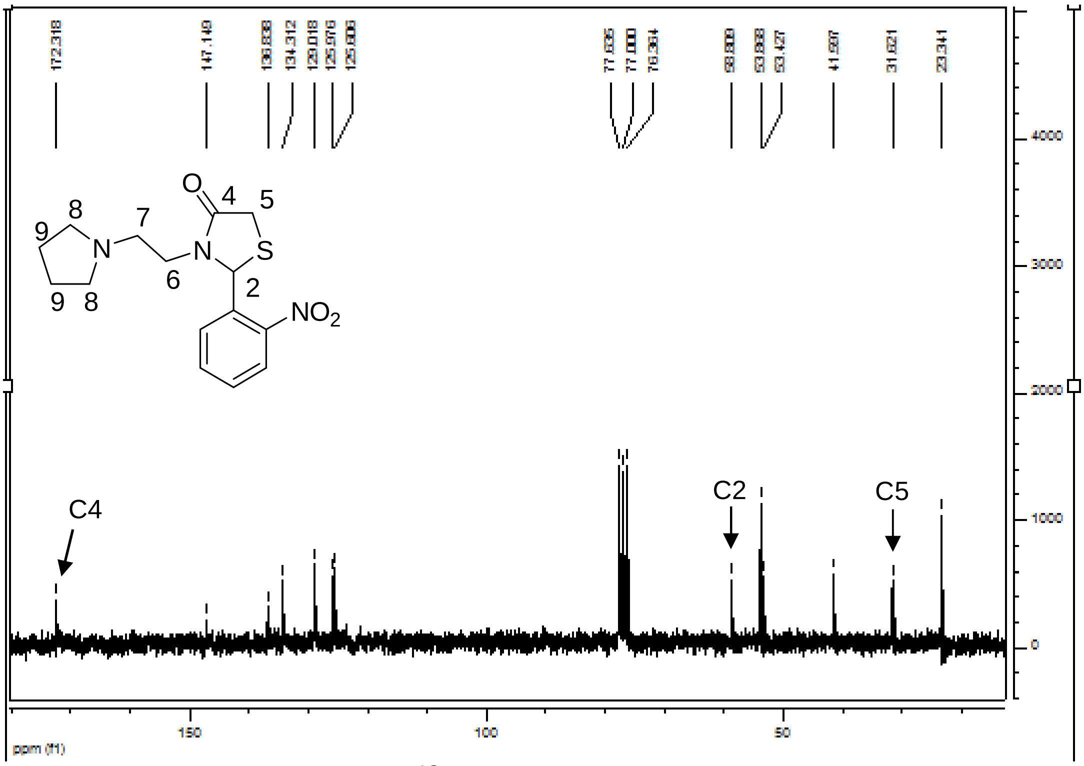

Figure S58: NMR ^{13}C spectrum of thiazolidin-4-one **5h**.

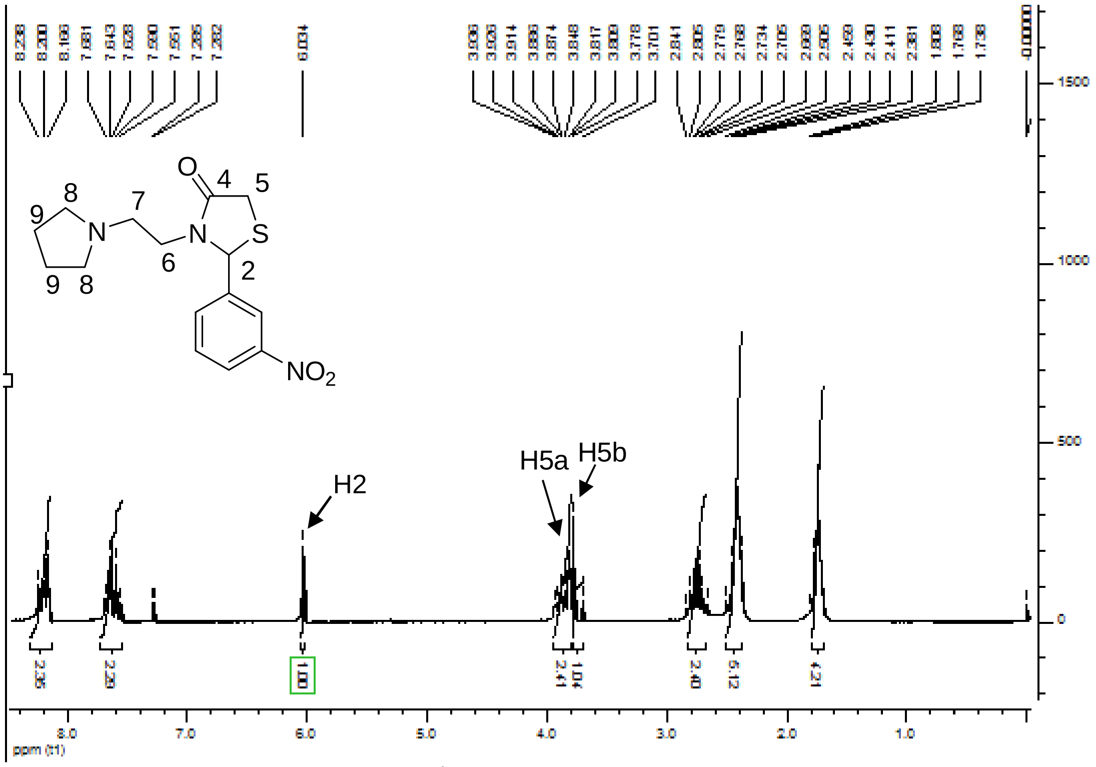

Figure S59: NMR ^{1}H spectrum of thiazolidin-4-one **5i**.

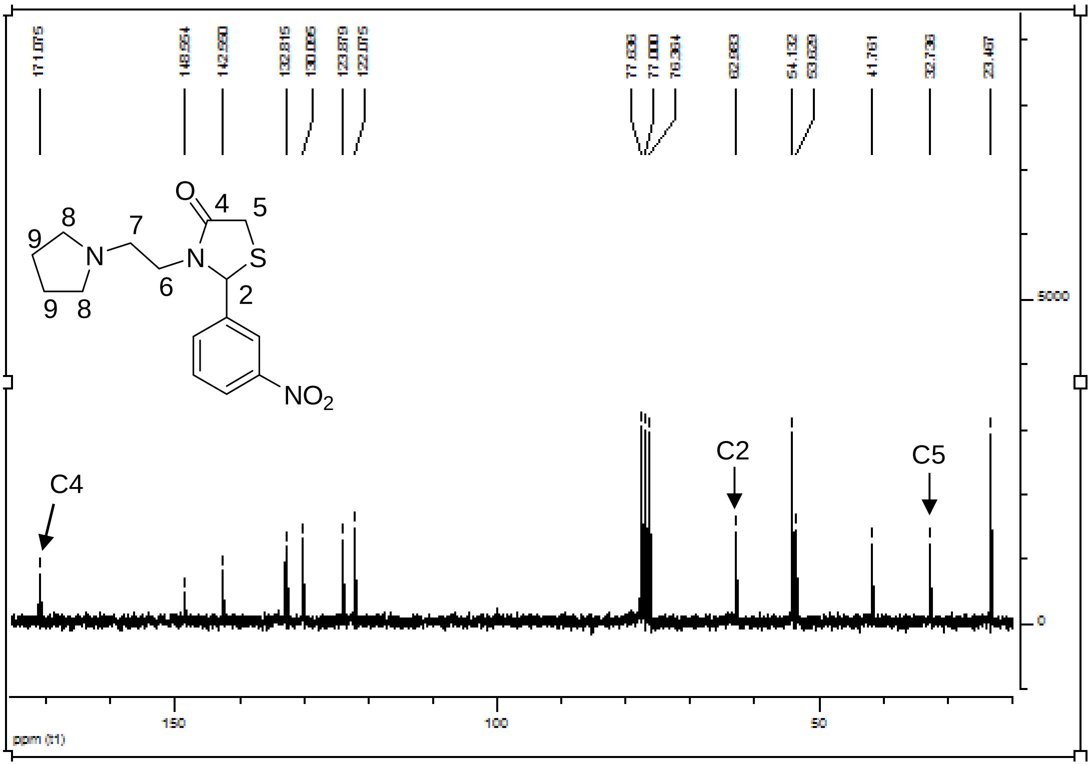

Figure S60: NMR ^{13}C spectrum of thiazolidin-4-one **5i**.

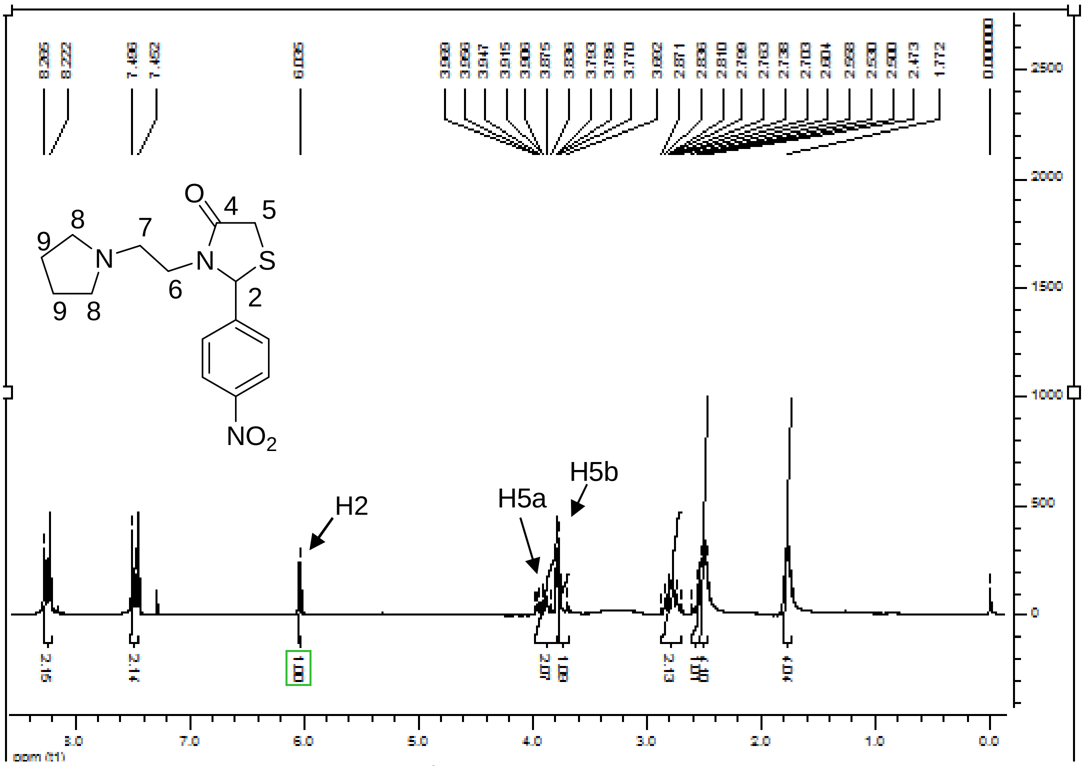

Figure S61: NMR ^{1}H spectrum of thiazolidin-4-one **5j**.

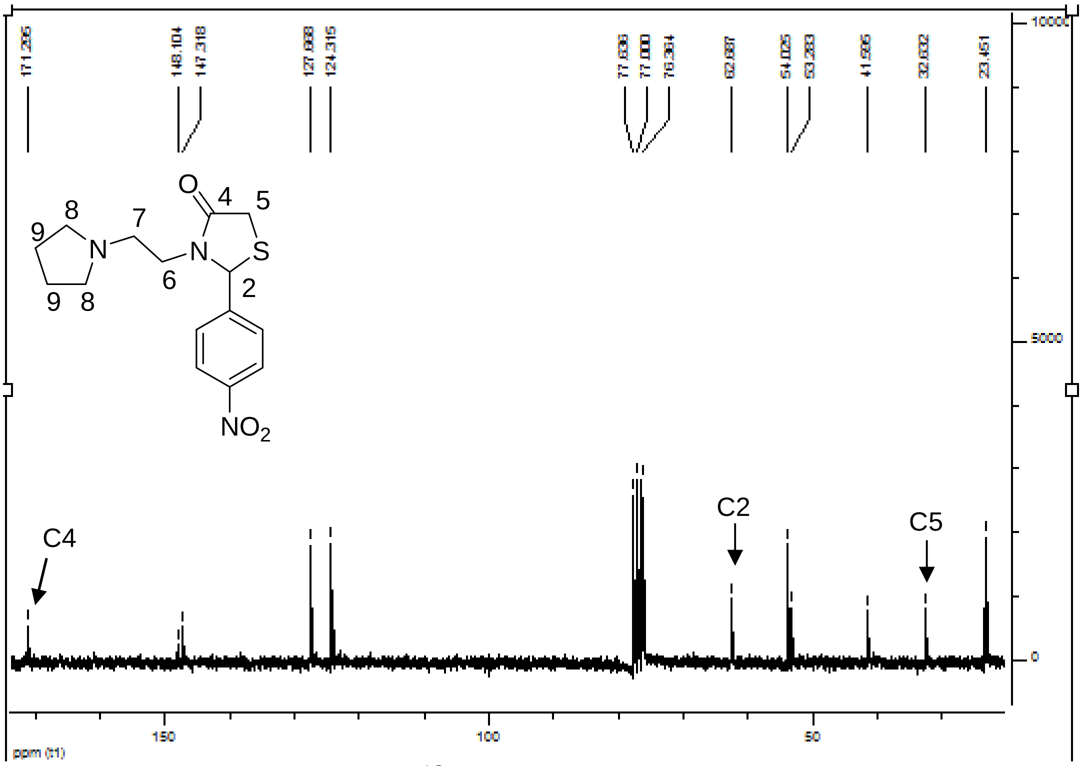

Figure S62: NMR ^{13}C spectrum of thiazolidin-4-one **5j**.

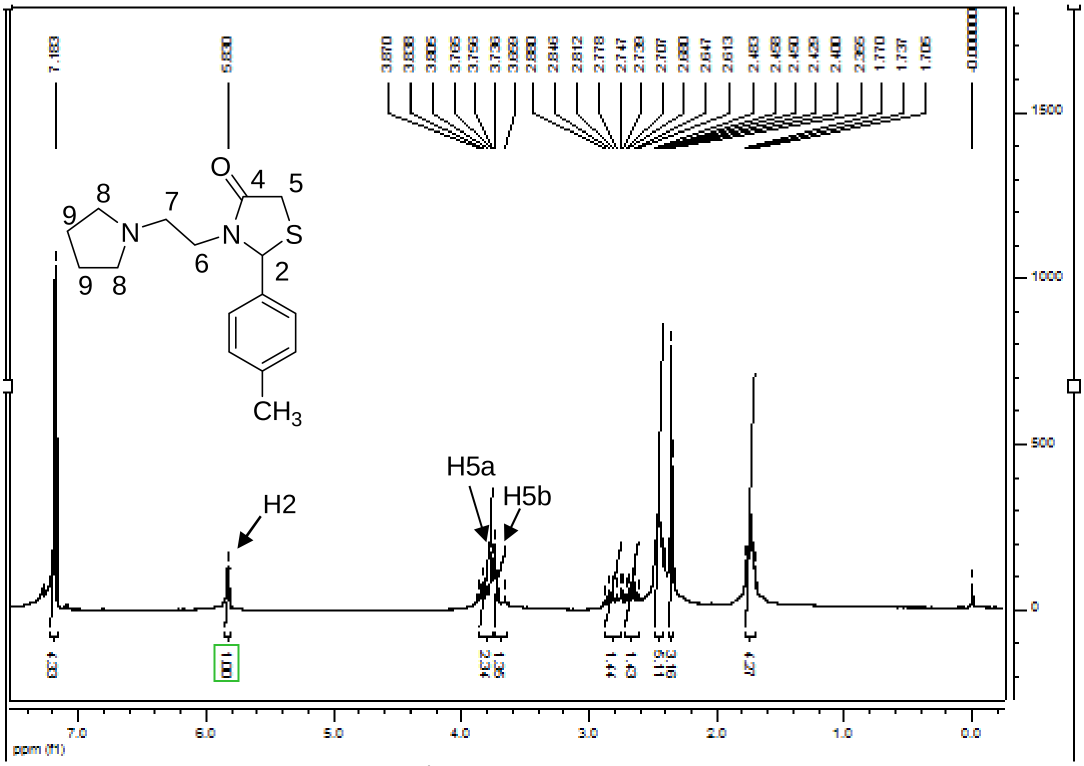

Figure S63: NMR ^{1}H spectrum of thiazolidin-4-one **5k**.

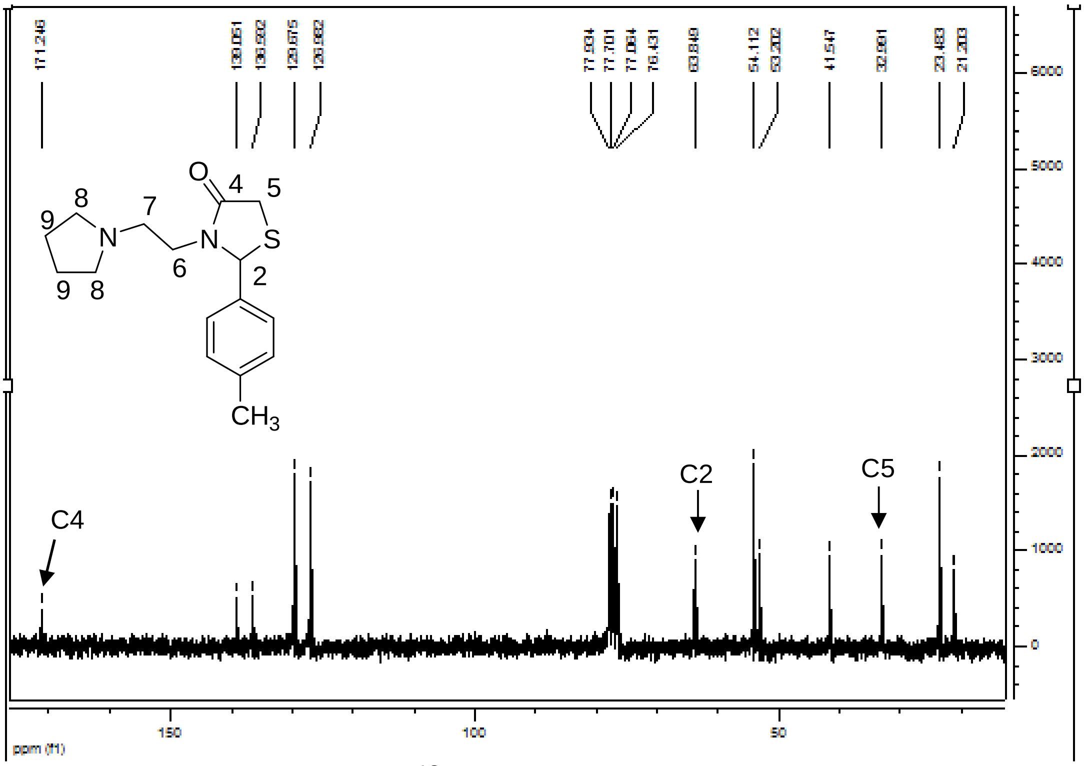

Figure S64: NMR ^{13}C spectrum of thiazolidin-4-one **5k**.

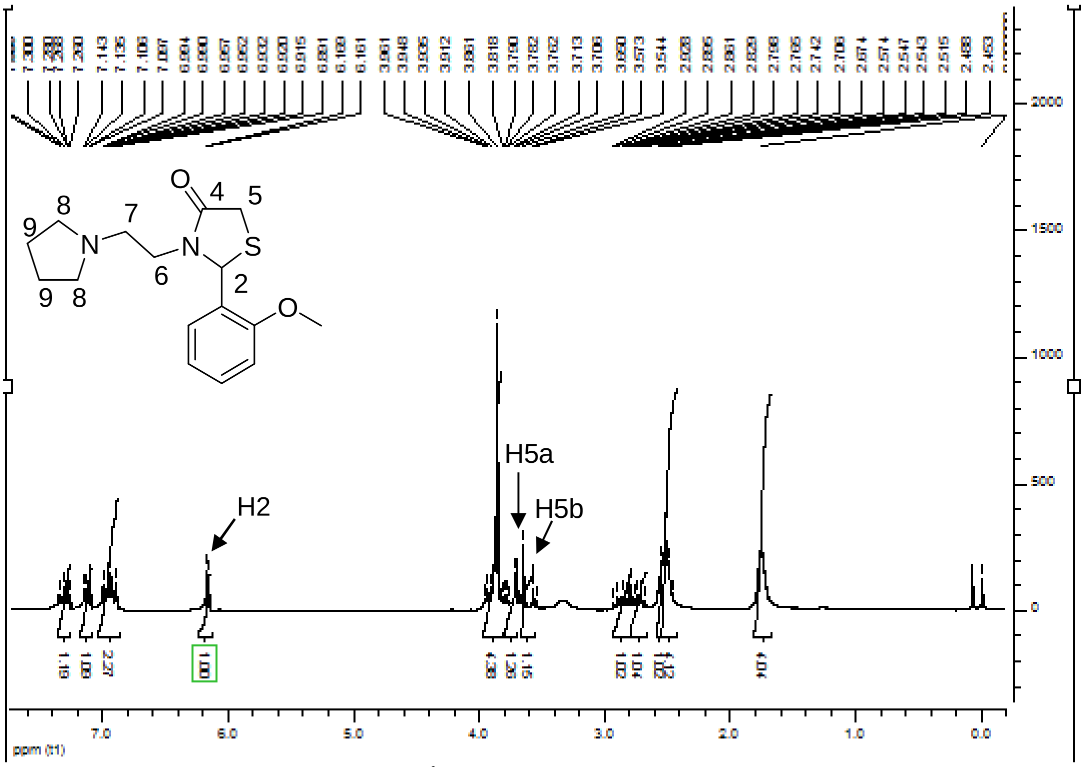

Figure S65: NMR ^{1}H spectrum of thiazolidin-4-one **5l**.

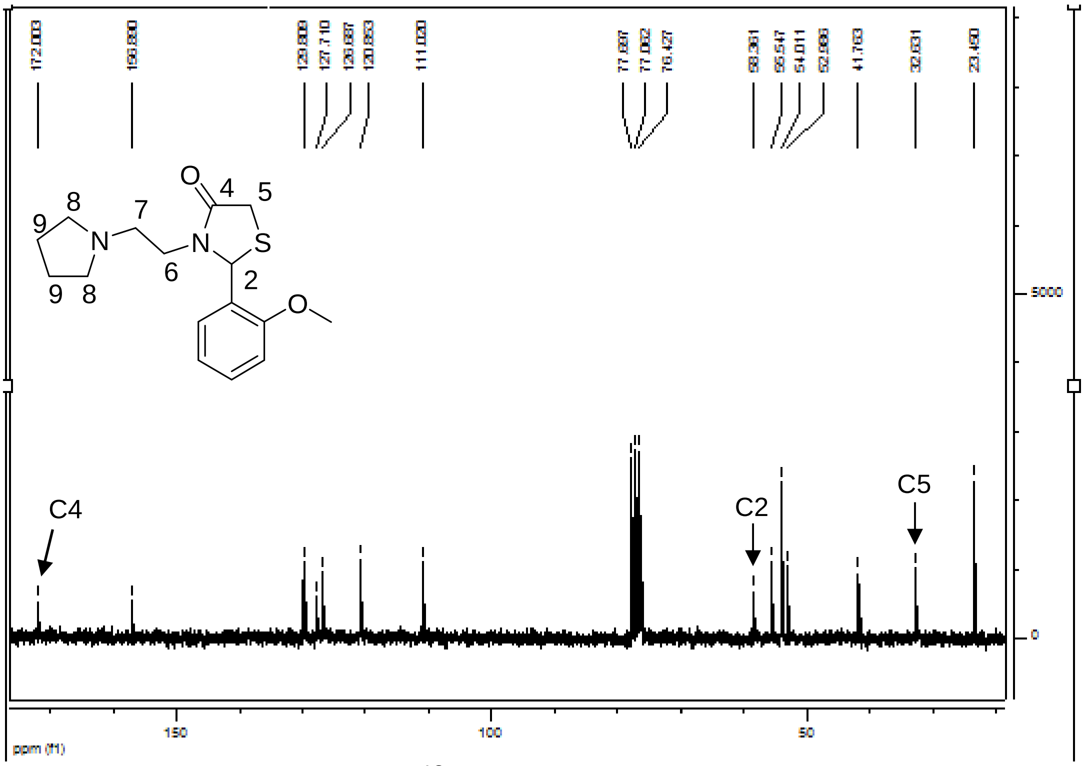

Figure S66: NMR ^{13}C spectrum of thiazolidin-4-one **5l**.

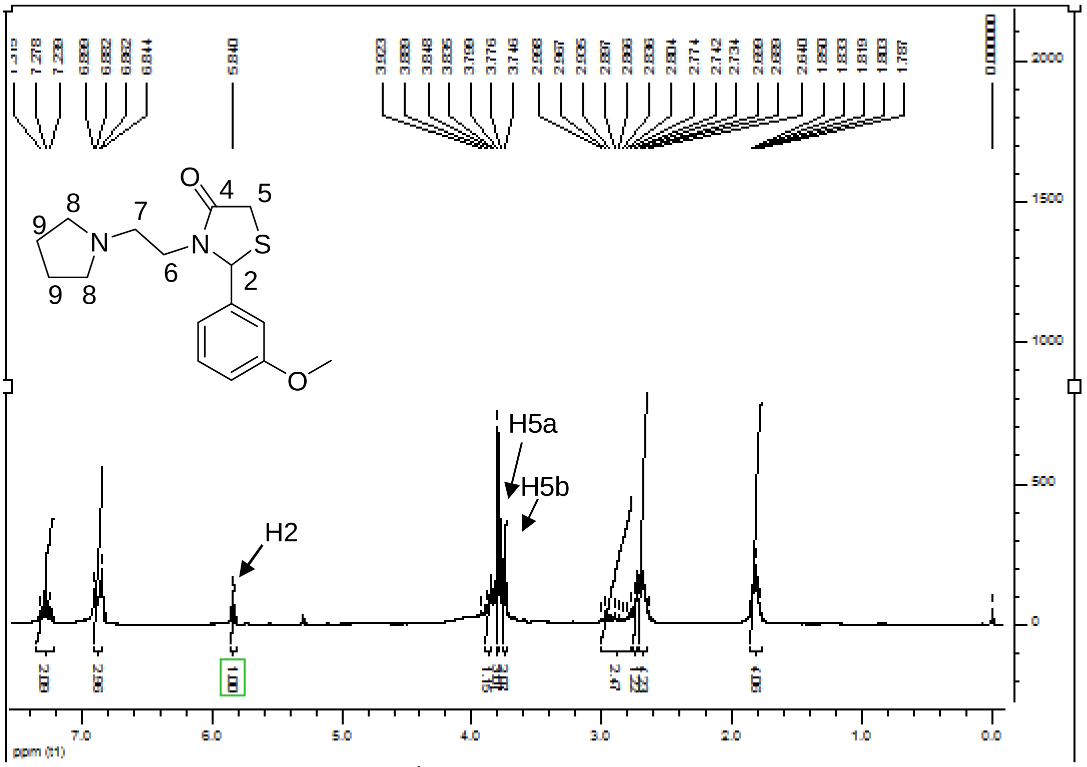

Figure S67: NMR ^{1}H spectrum of thiazolidin-4-one **5m**.

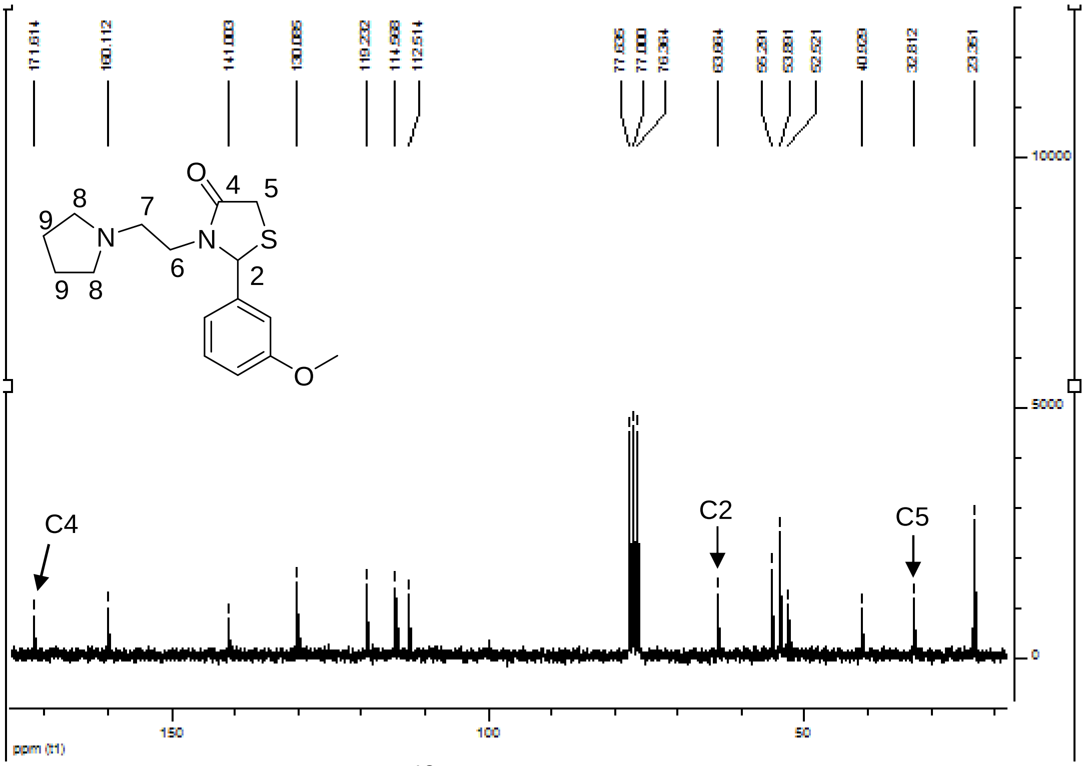

Figure S68: NMR ^{13}C spectrum of thiazolidin-4-one **5m**.

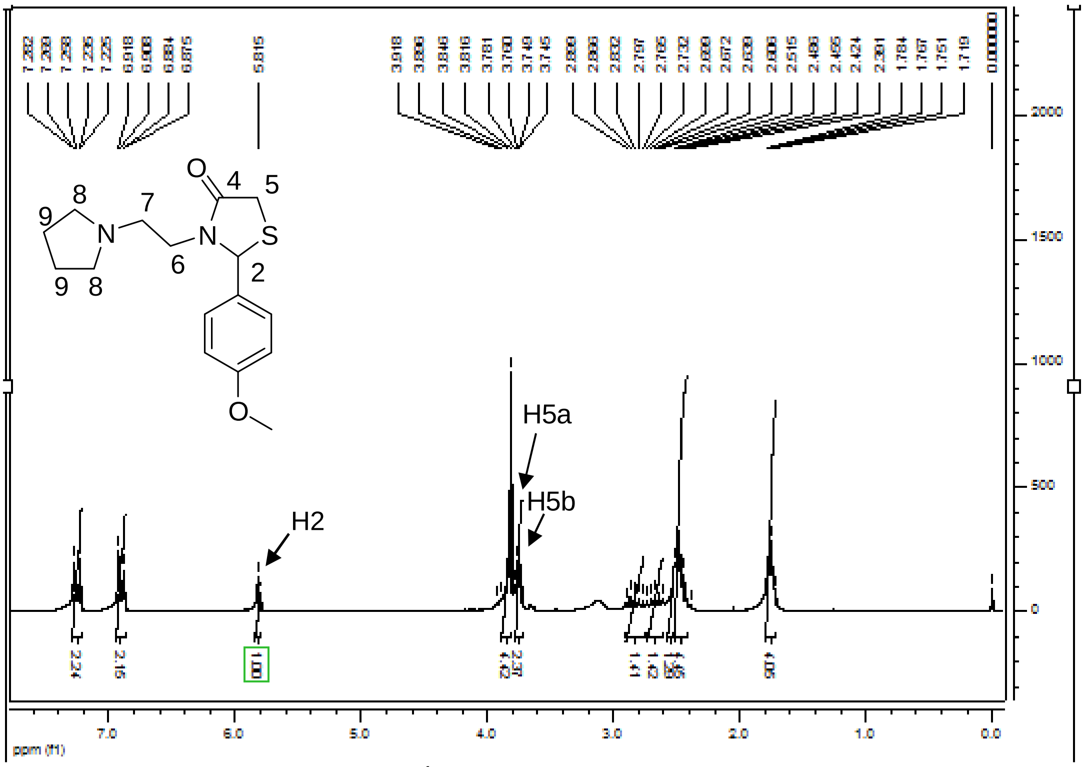

Figure S69: NMR ^{1}H spectrum of thiazolidin-4-one **5n**.

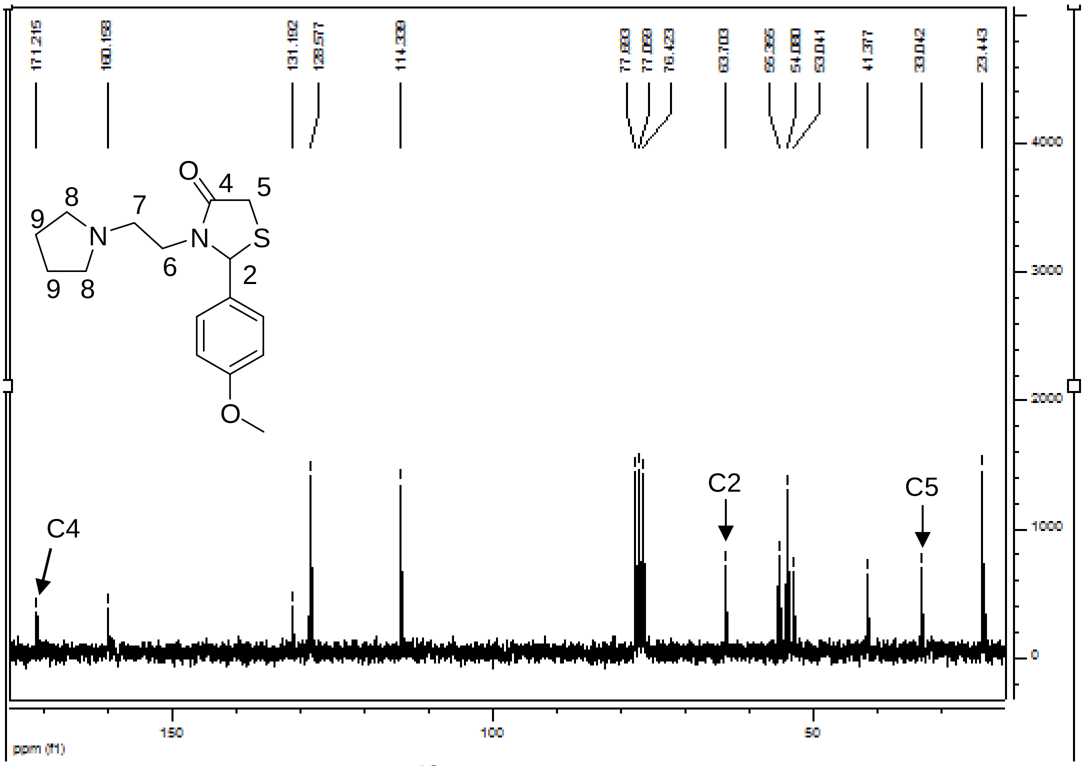

Figure S70: NMR ^{13}C spectrum of thiazolidin-4-one **5n**.

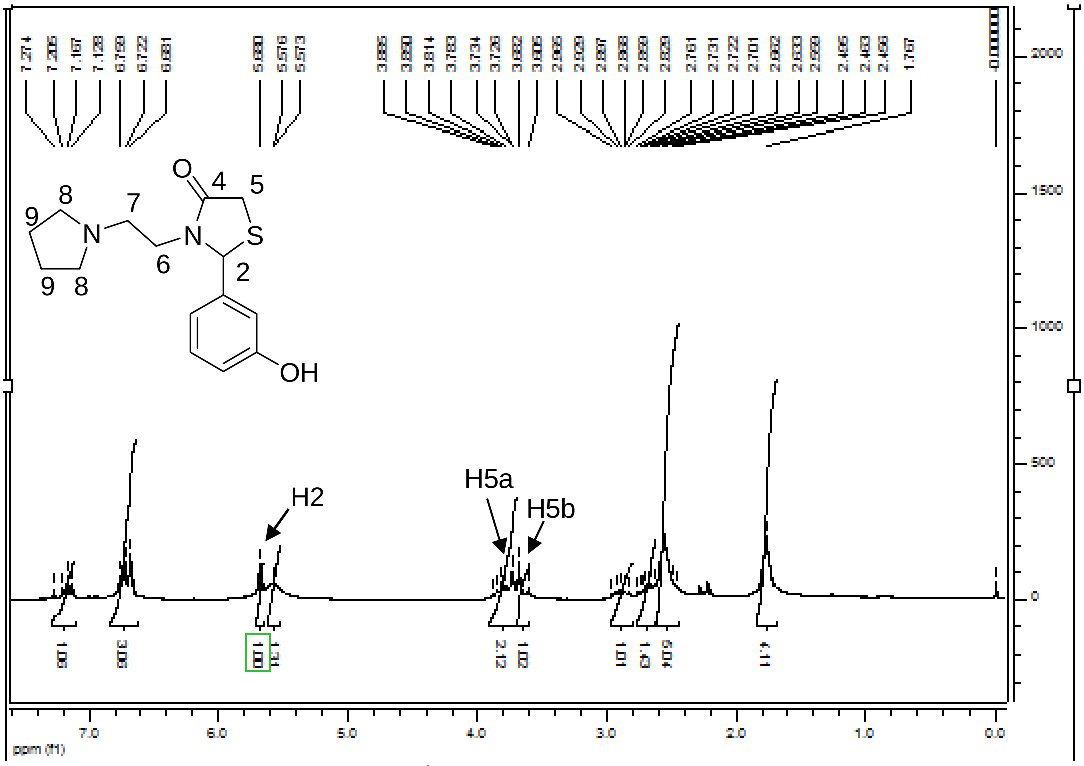

Figure S71: NMR ^{1}H spectrum of thiazolidin-4-one **5o**.

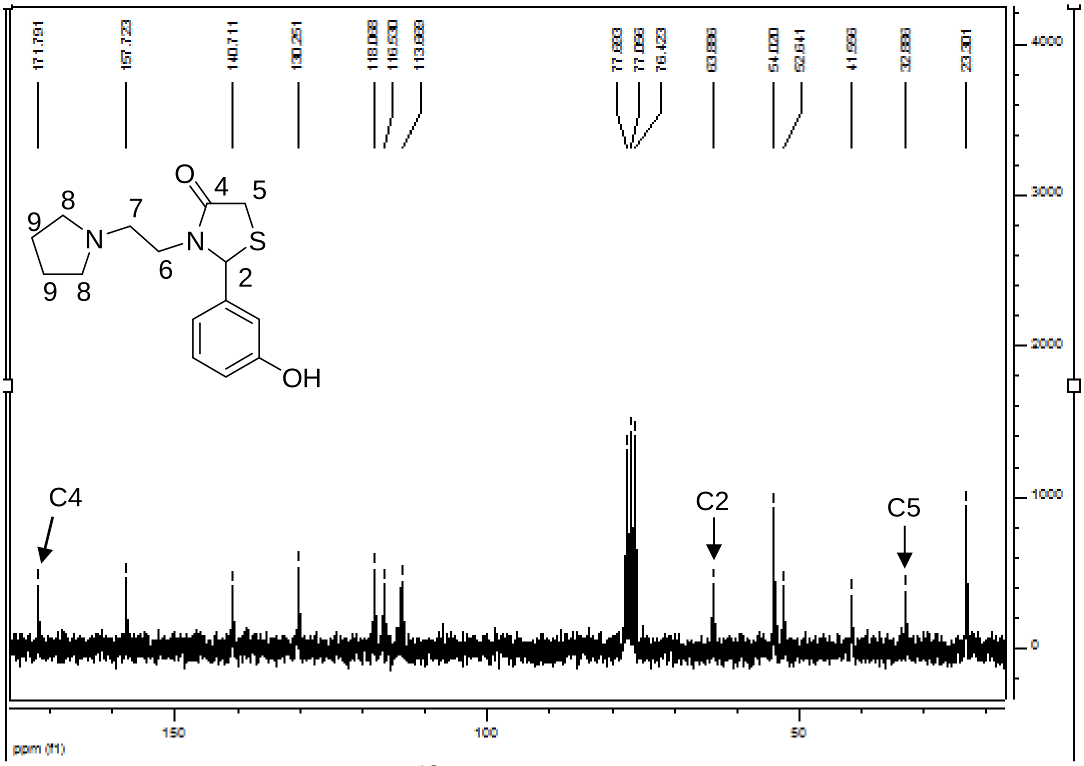

Figure S72: NMR ^{13}C spectrum of thiazolidin-4-one **5o**.

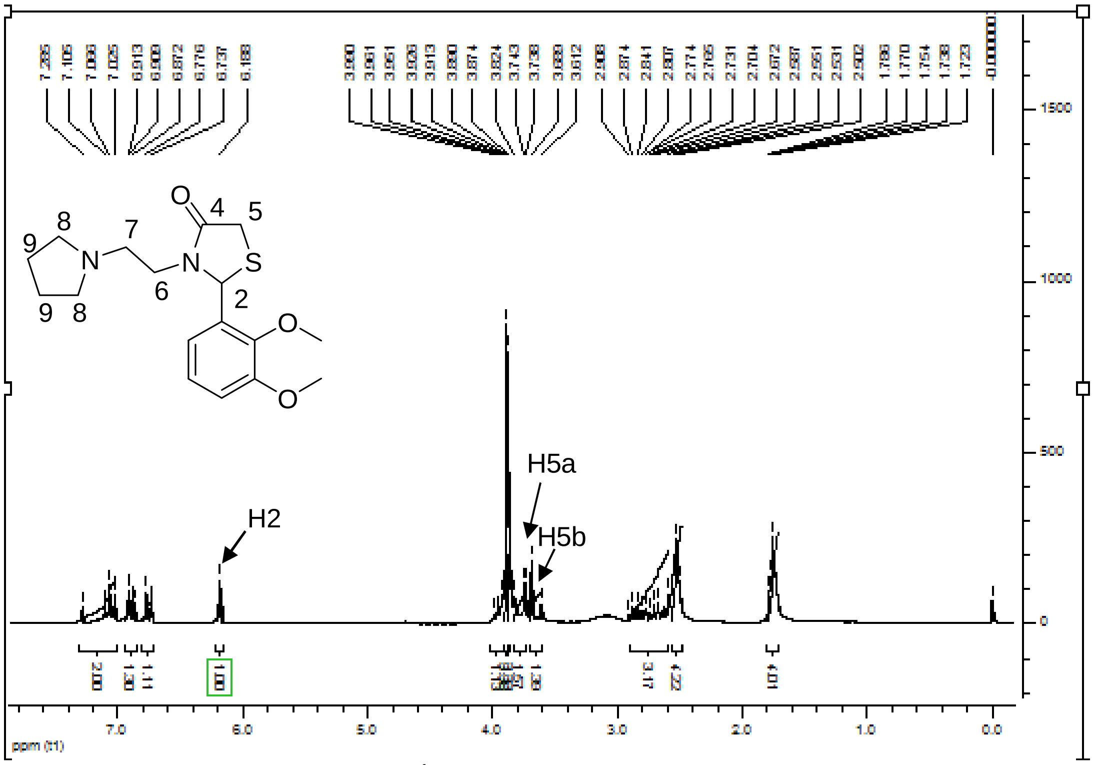

Figure S73: NMR ^{1}H spectrum of thiazolidin-4-one **5p**.

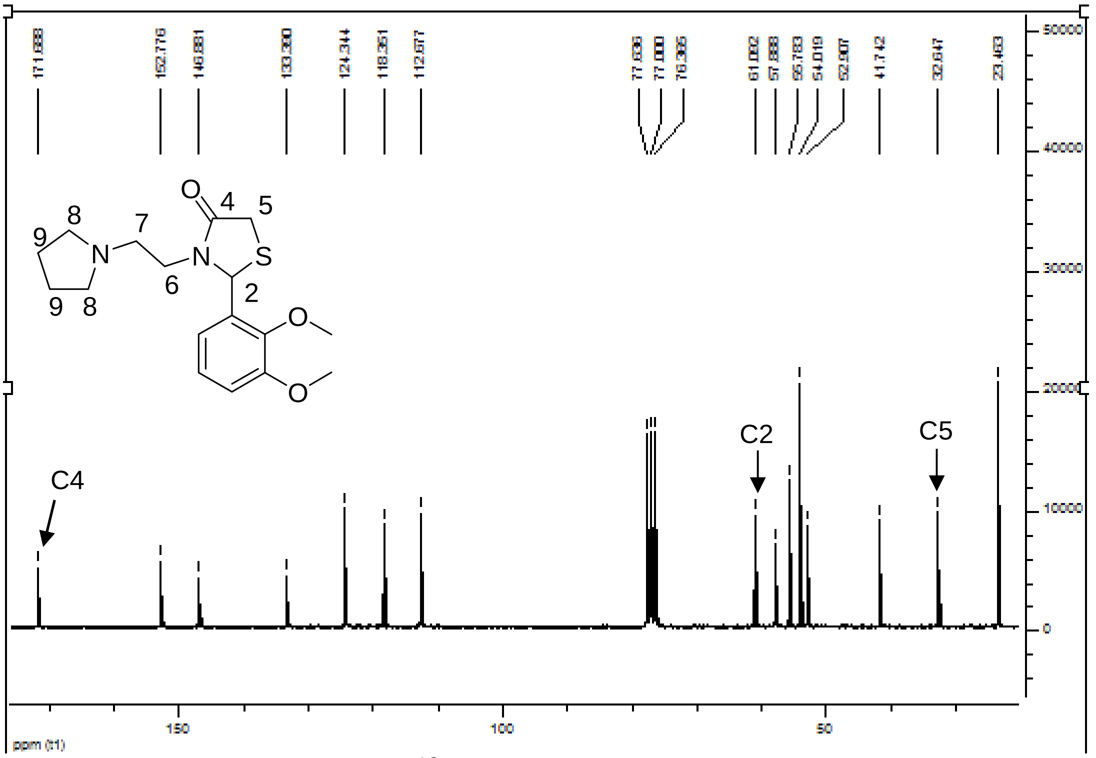

Figure S74: NMR ^{13}C spectrum of thiazolidin-4-one **5p**.

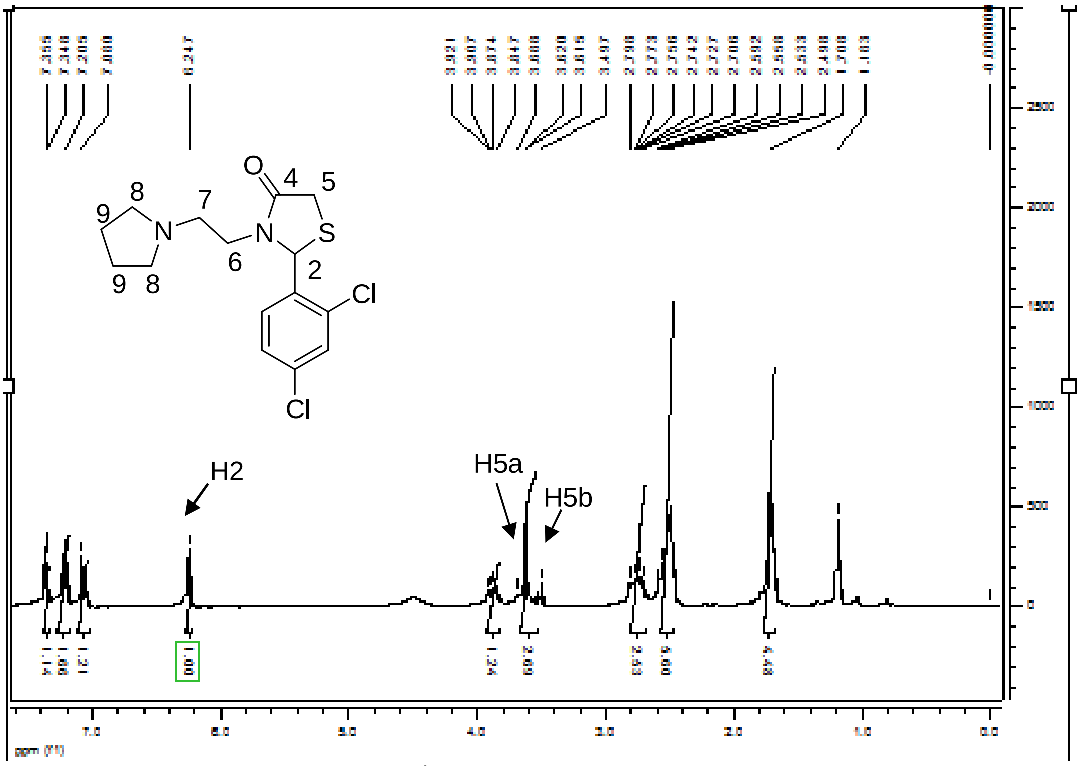

Figure S75: NMR ^{1}H spectrum of thiazolidin-4-one **5q**.

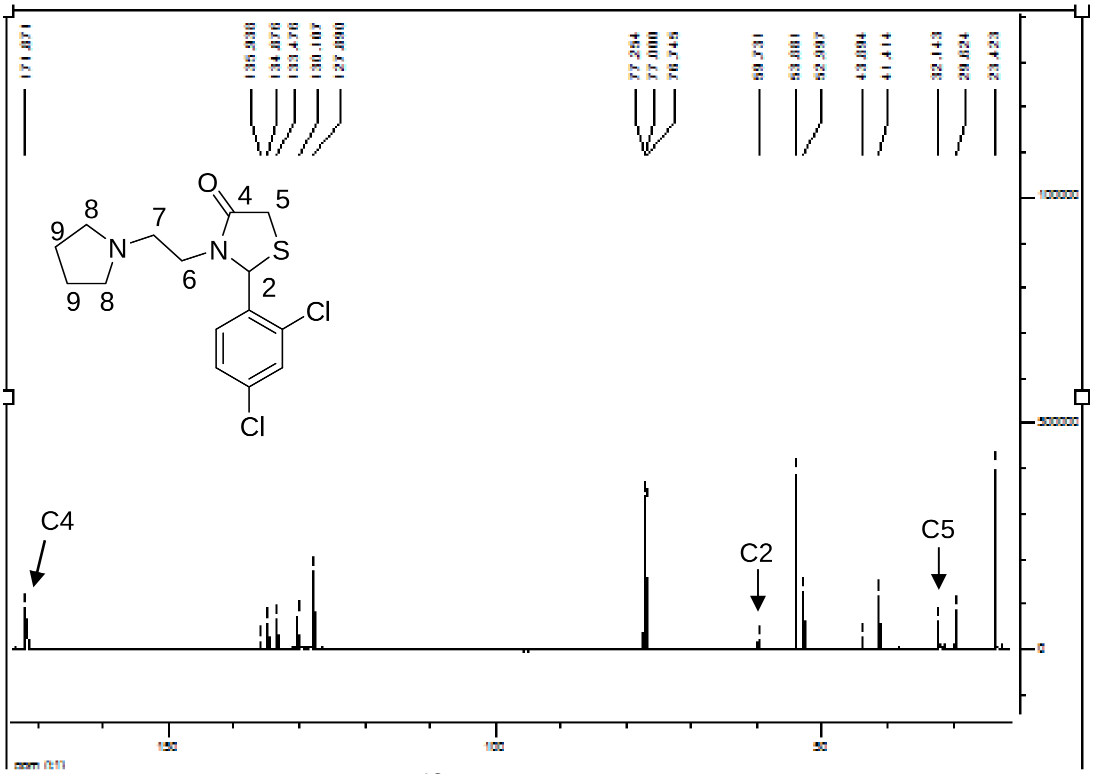

Figure S76: NMR ^{13}C spectrum of thiazolidin-4-one **5q**.

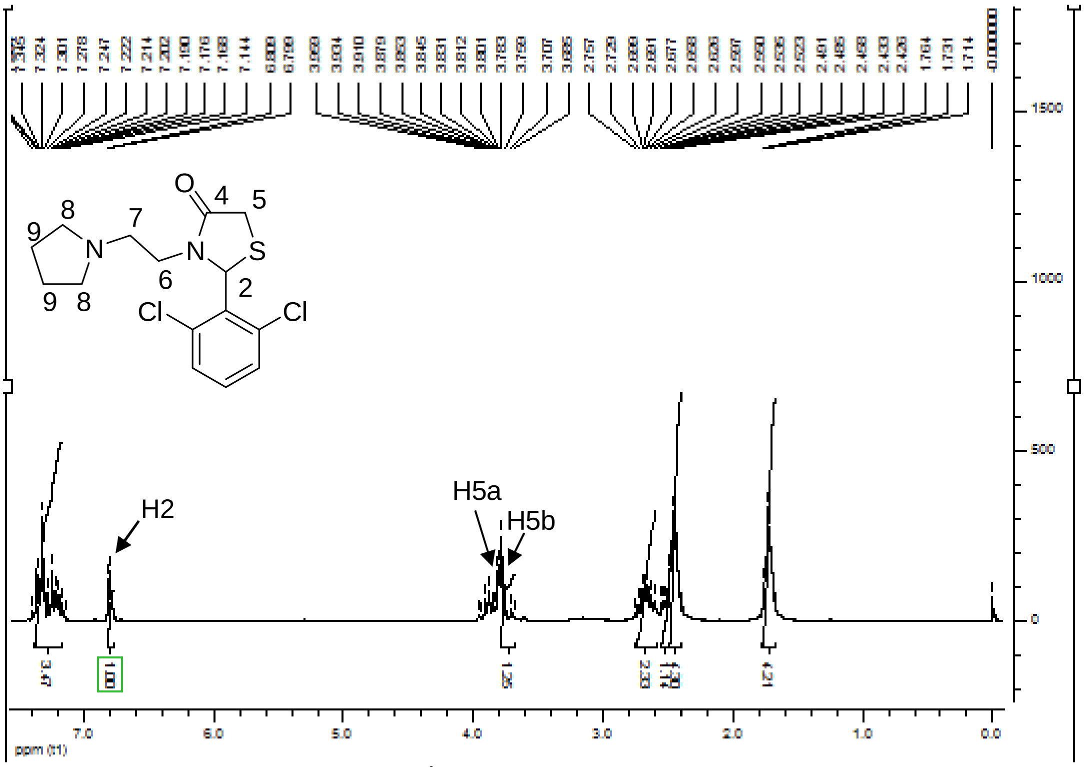

Figure S77: NMR ^{1}H spectrum of thiazolidin-4-one **5r**.

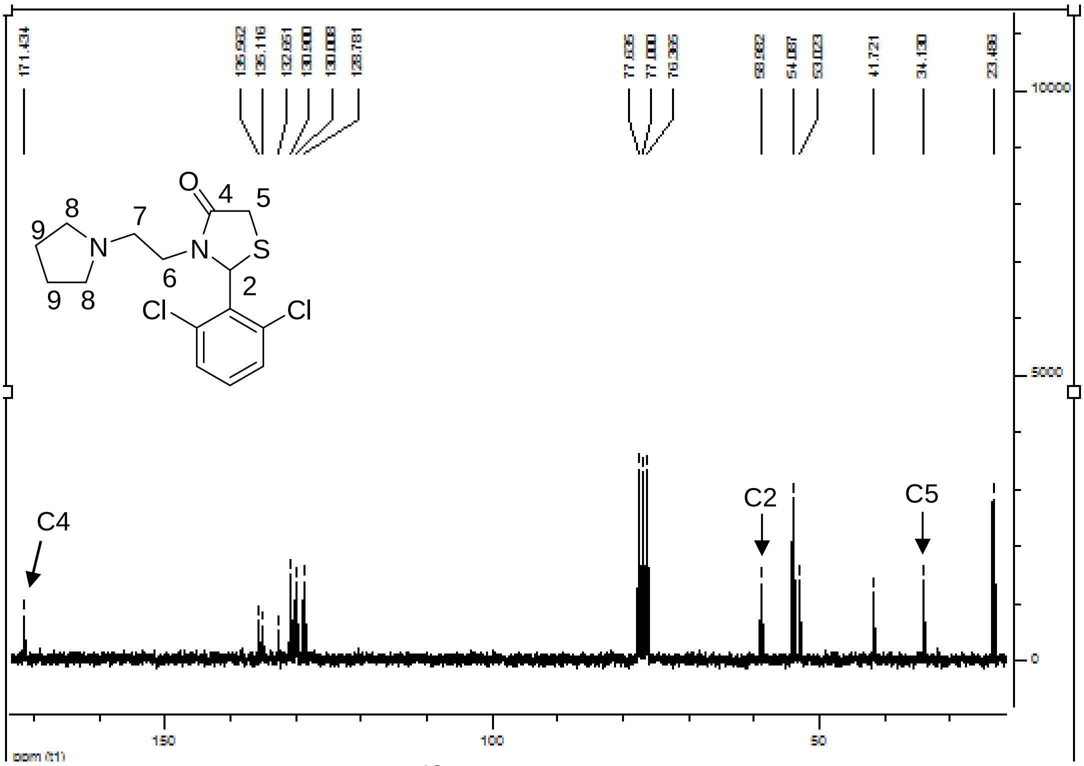

Figure S78: NMR ^{13}C spectrum of thiazolidin-4-one **5r**.

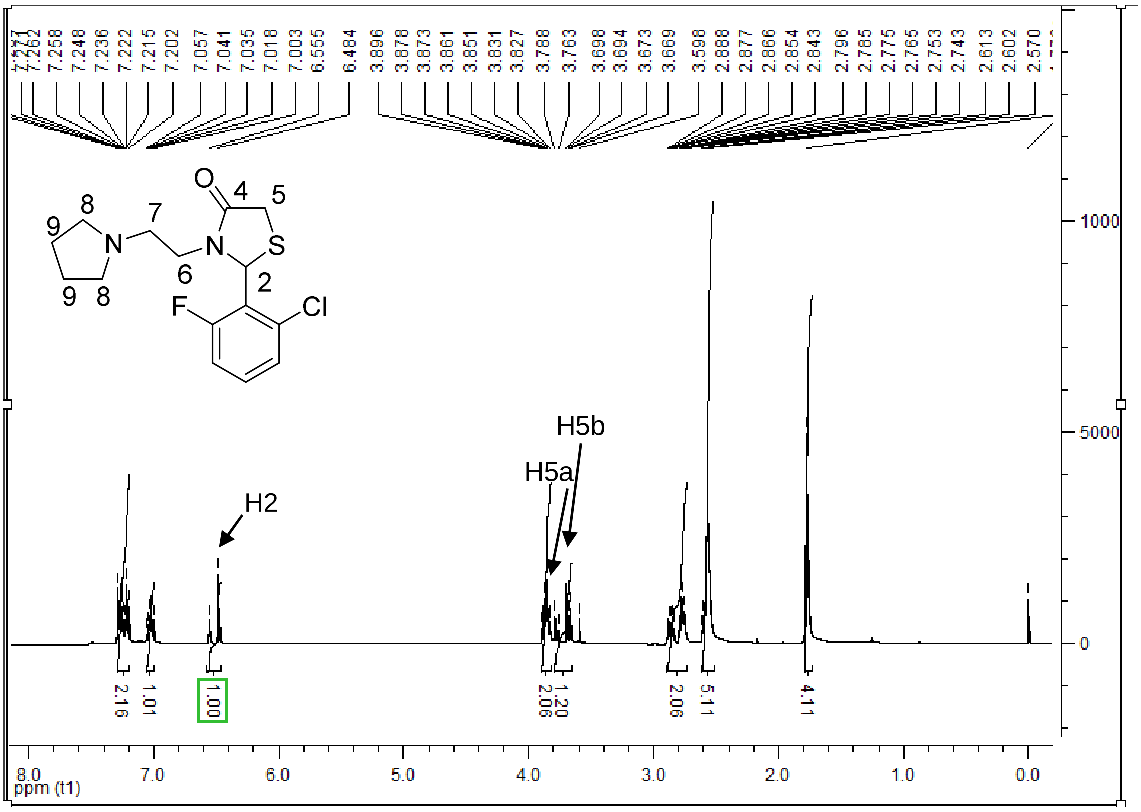

Figure S79: NMR ^{1}H spectrum of thiazolidin-4-one **5s**.

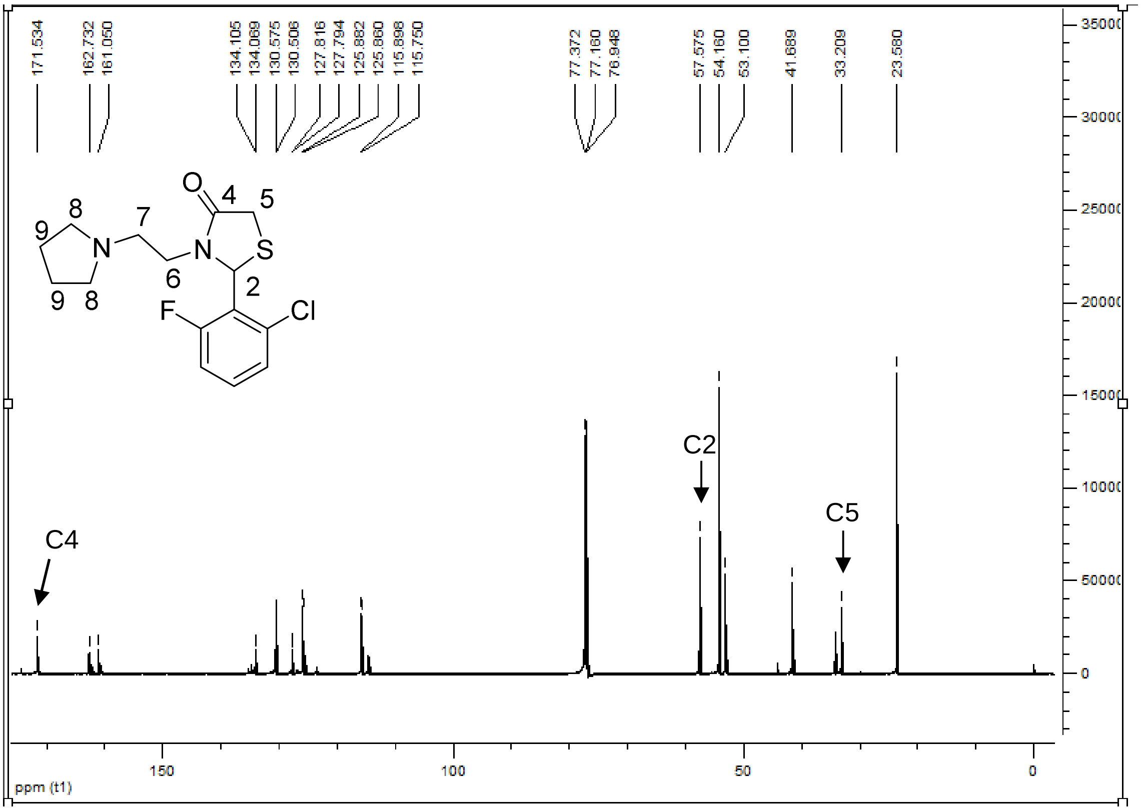

Figure S80: NMR ^{13}C spectrum of thiazolidin-4-one **5s**.

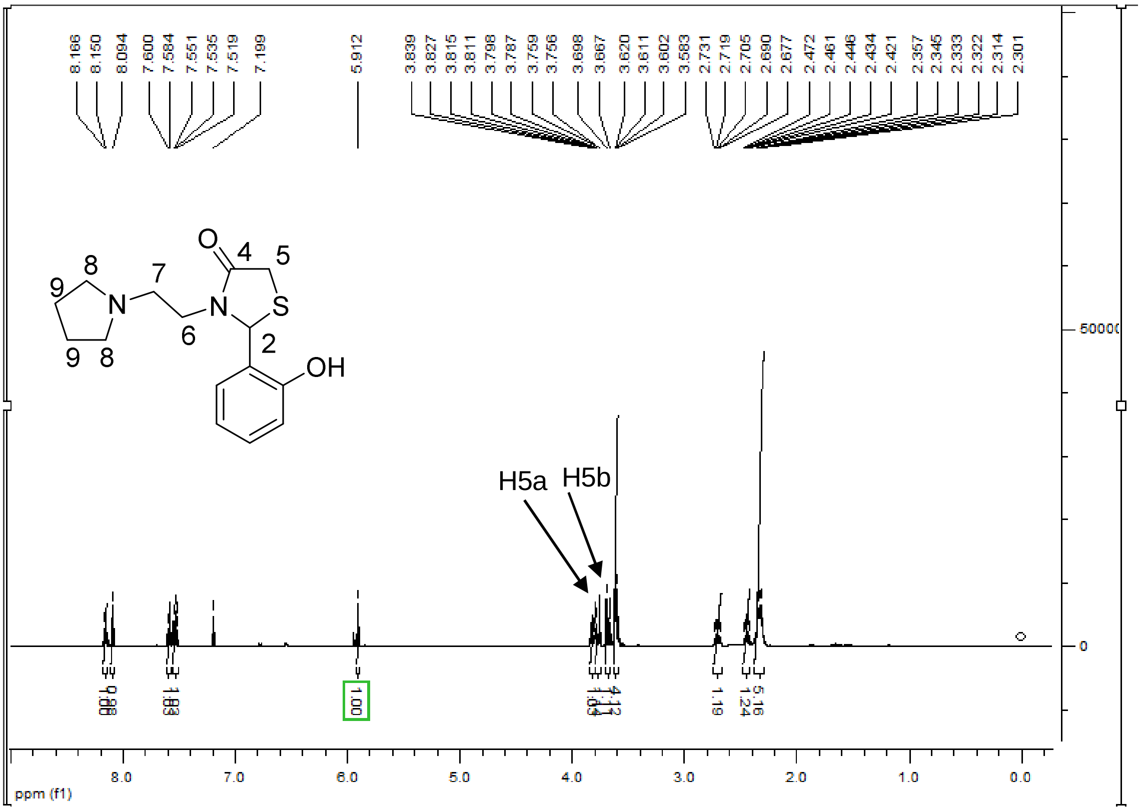

Figure S81: NMR ^{1}H spectrum of thiazolidin-4-one **5t**.

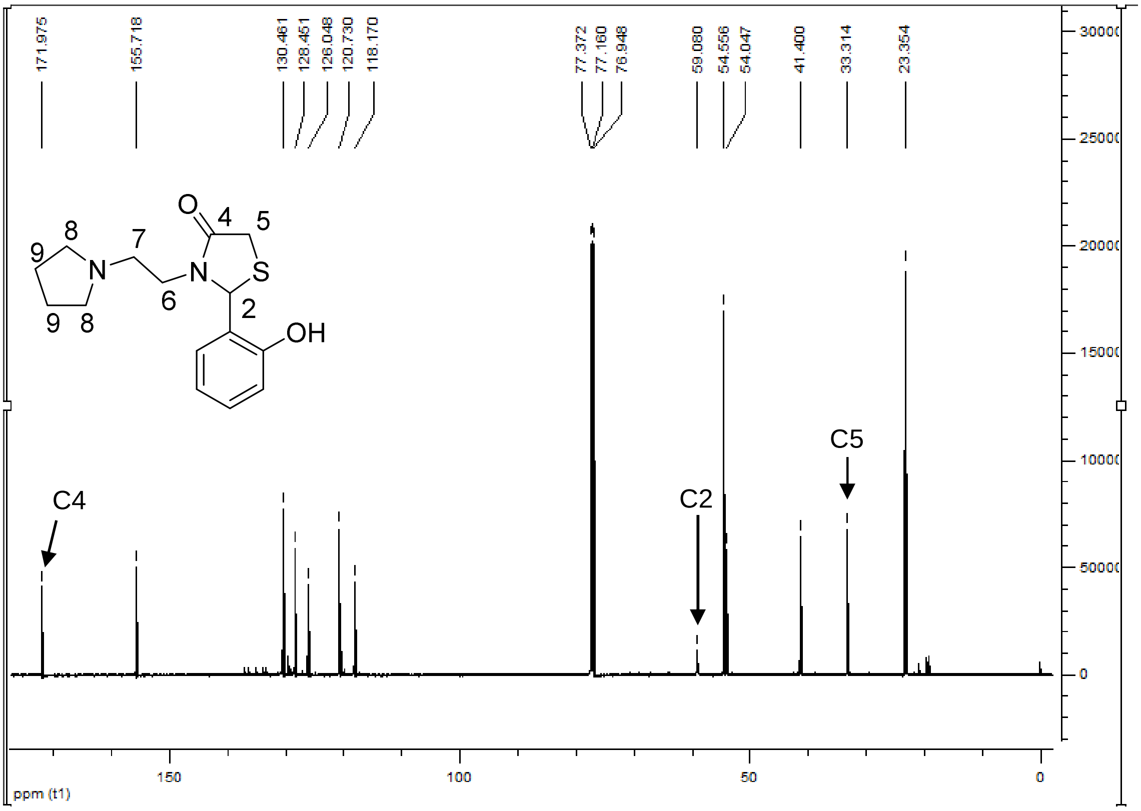

Figure S82: NMR ^{13}C spectrum of thiazolidin-4-one **5t**.

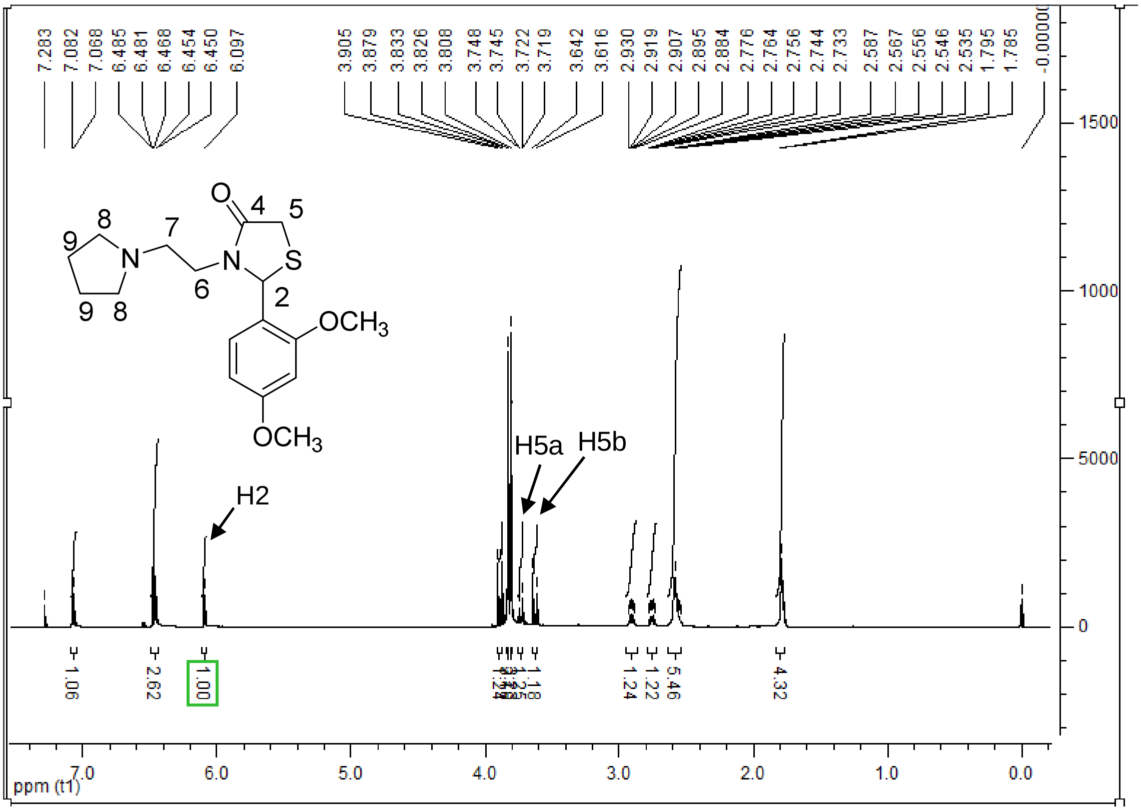

Figure S83: NMR ^{1}H spectrum of thiazolidin-4-one **5u**.

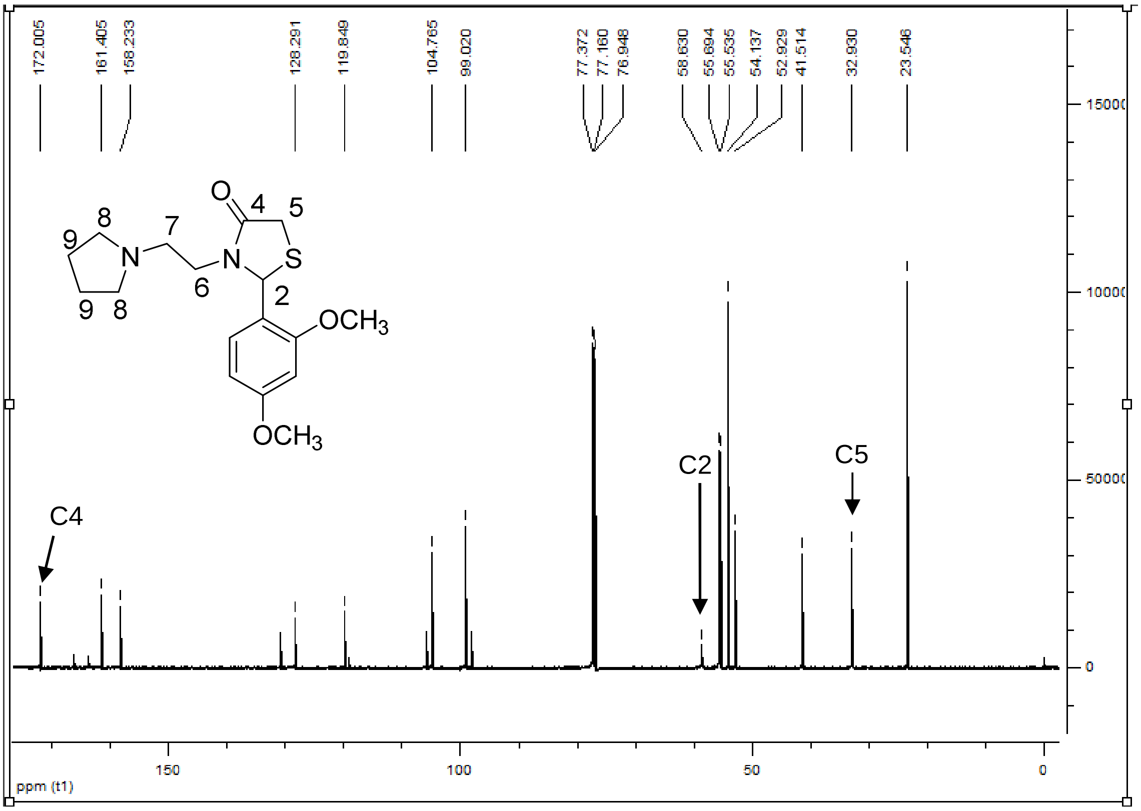

Figure S84: NMR ^{13}C spectrum of thiazolidin-4-one **5u**.

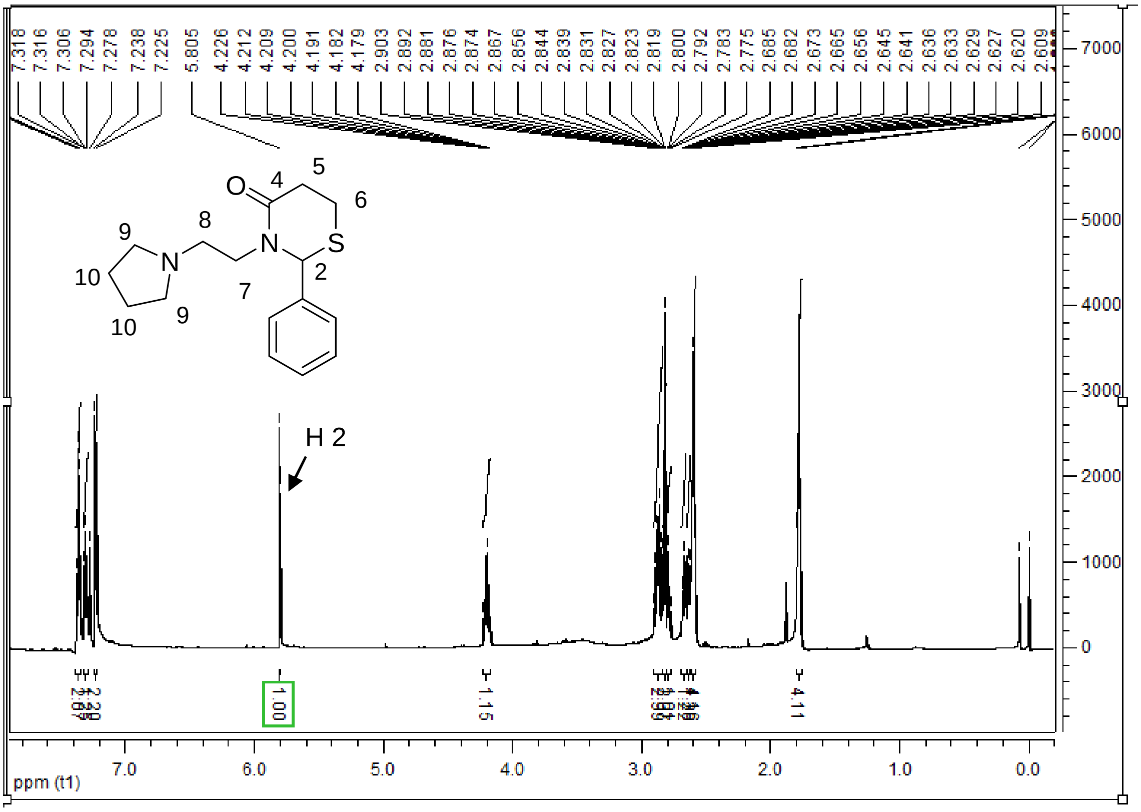

Figure S85: NMR ^{1}H spectrum of thiazinan-4-one **6a**.

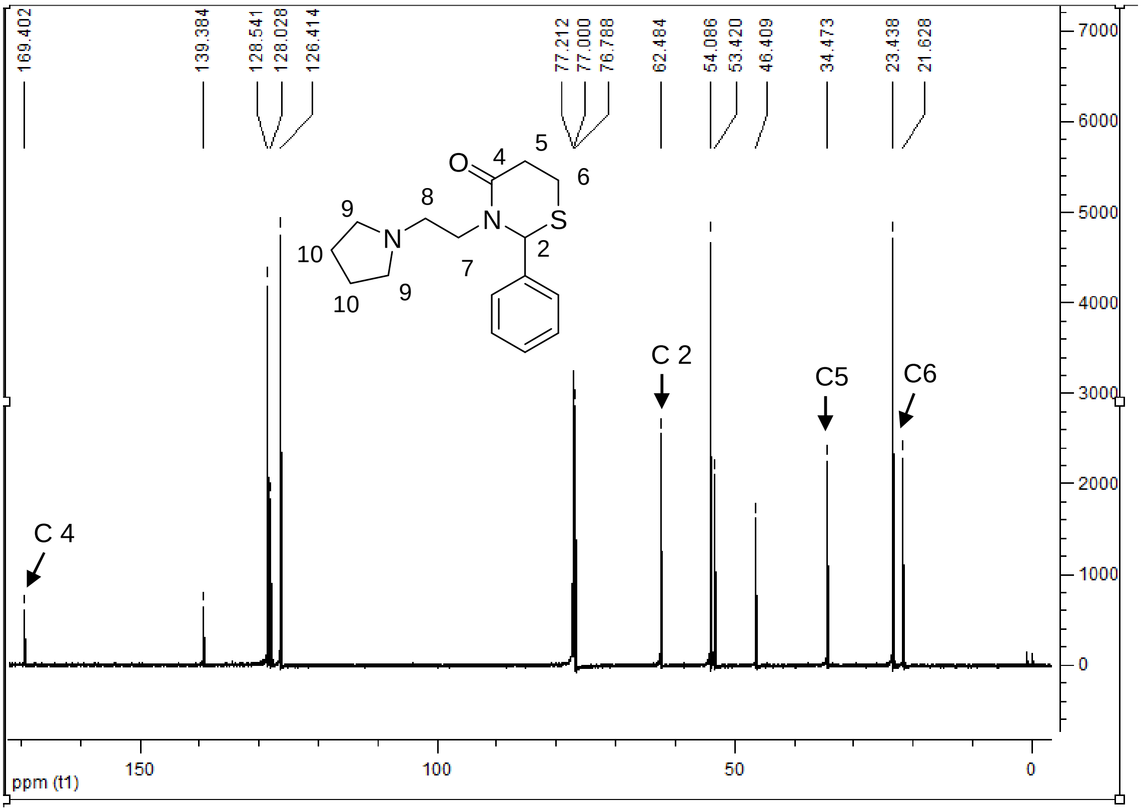

Figure S86: NMR ^{13}C spectrum of thiazinan-4-one **6a**.

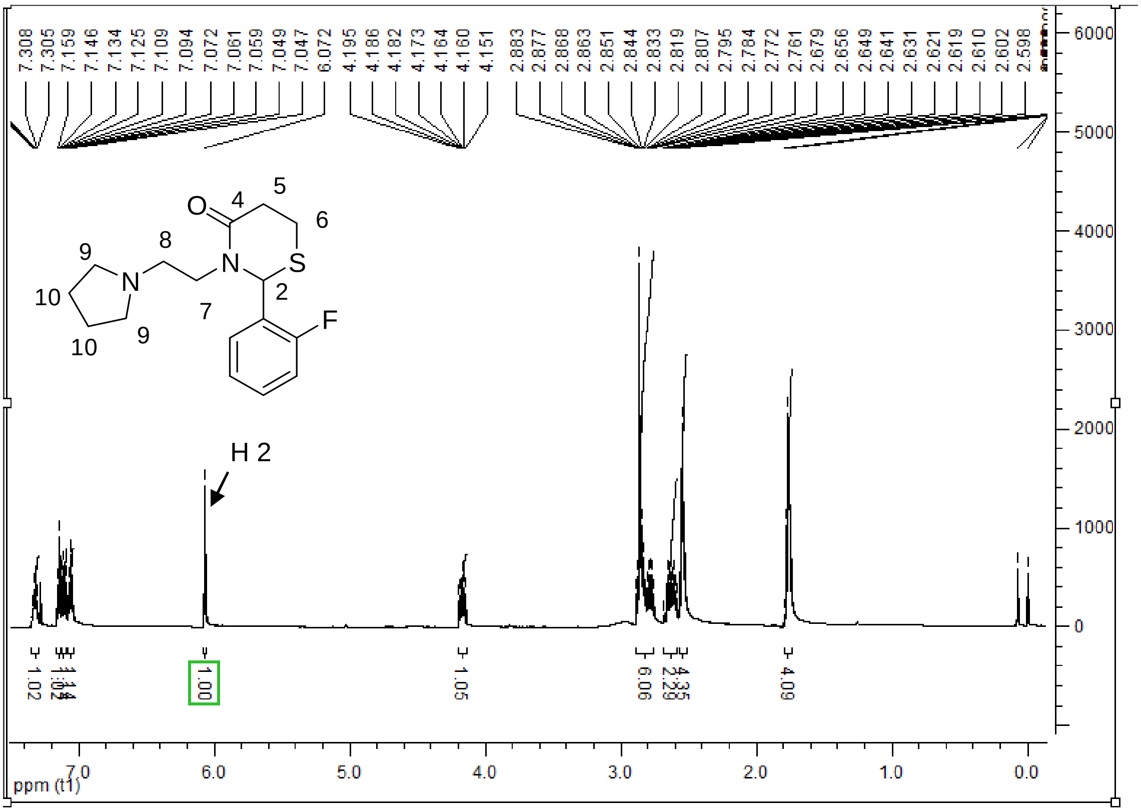

Figure S87: NMR ^{1}H spectrum of thiazinan-4-one **6b**.

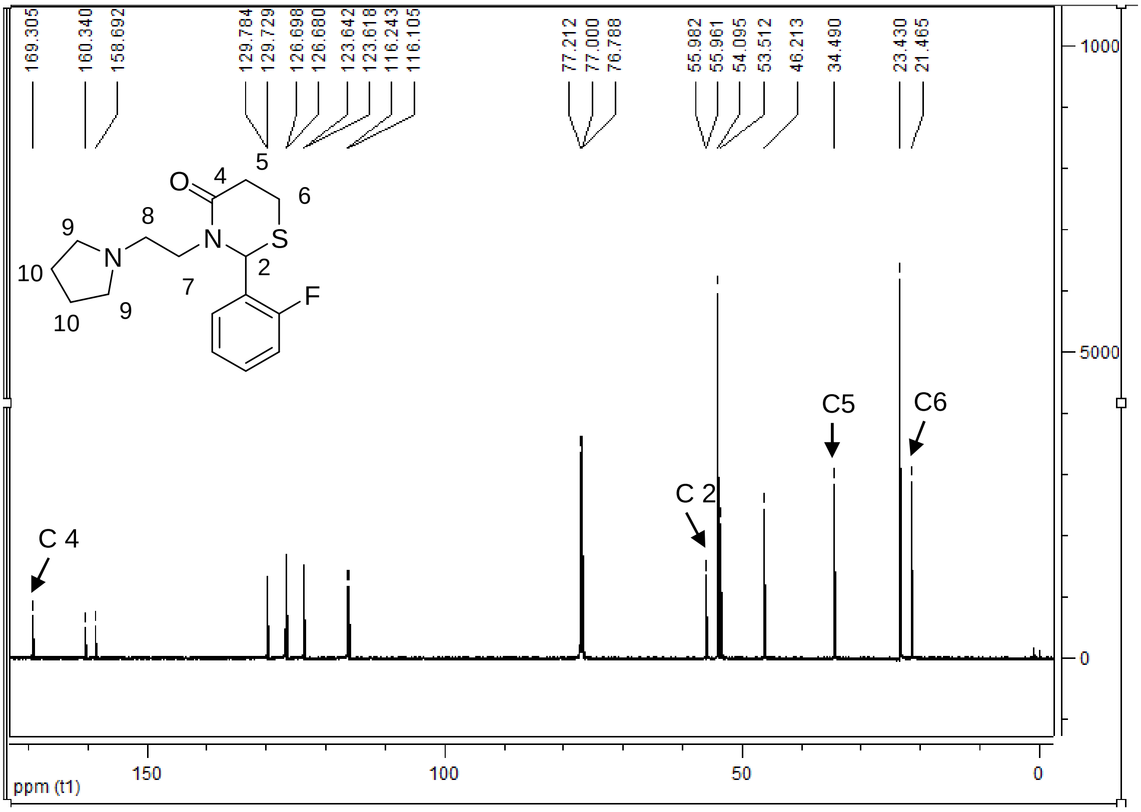

Figure S88: NMR ^{13}C spectrum of thiazinan-4-one **6b**.

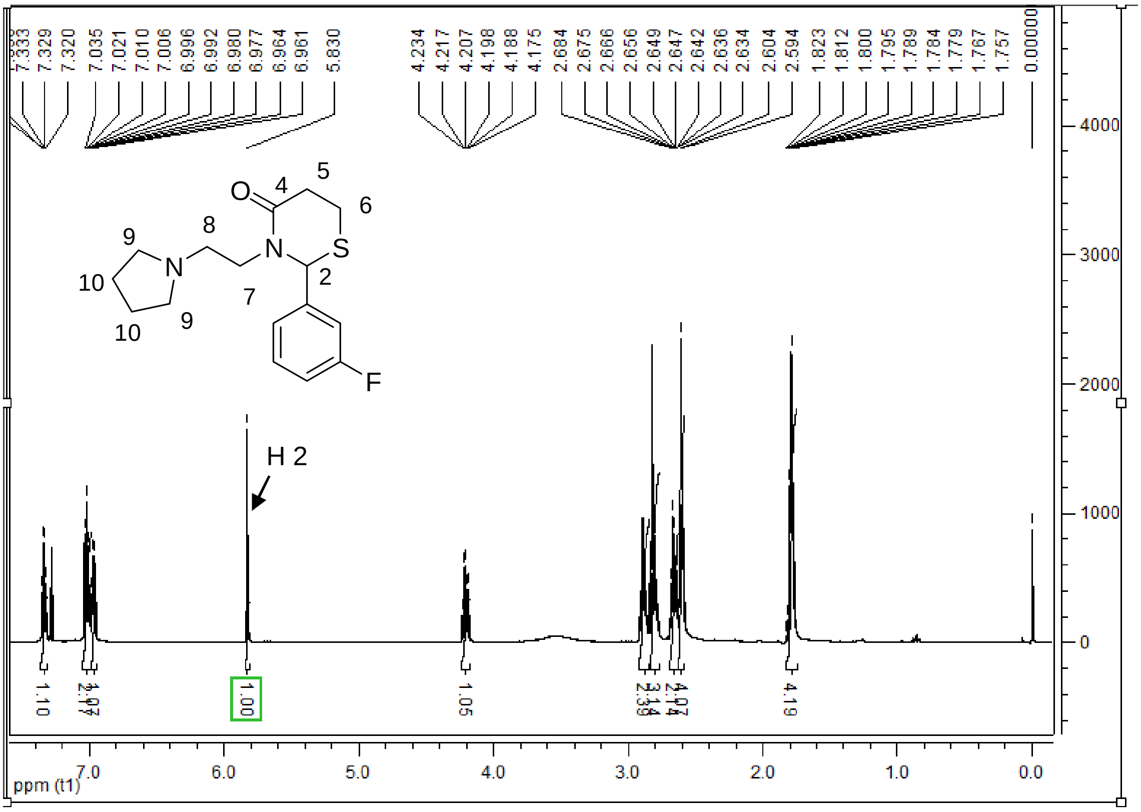

Figure S89: NMR ^{1}H spectrum of thiazinan-4-one **6c**.

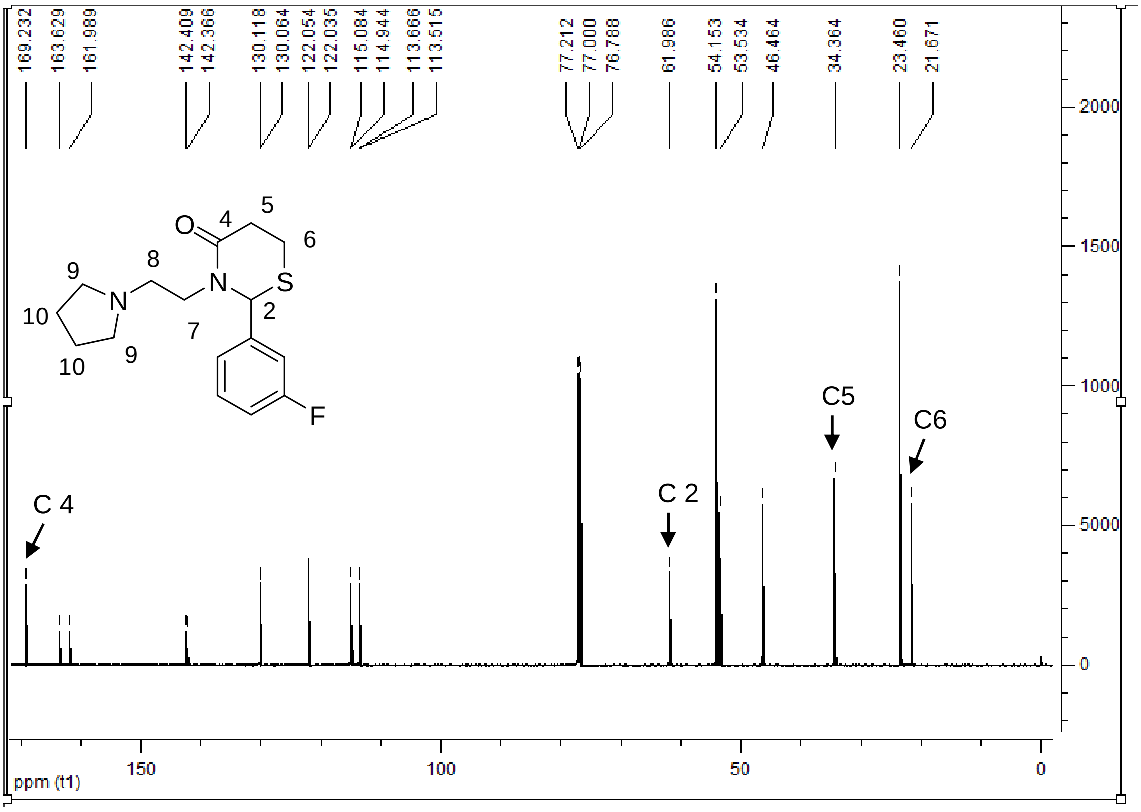

Figure S90: NMR ^{13}C spectrum of thiazinan-4-one **6c**.

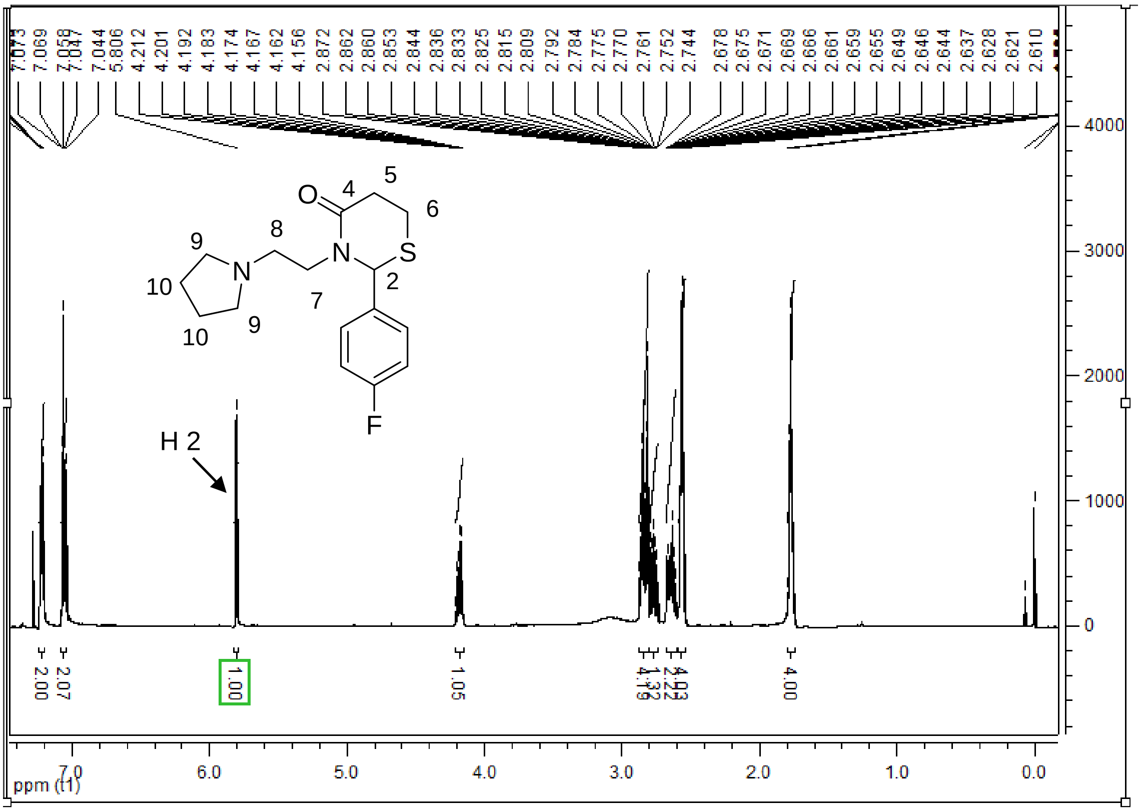

Figure S91: NMR ^{1}H spectrum of thiazinan-4-one **6d**.

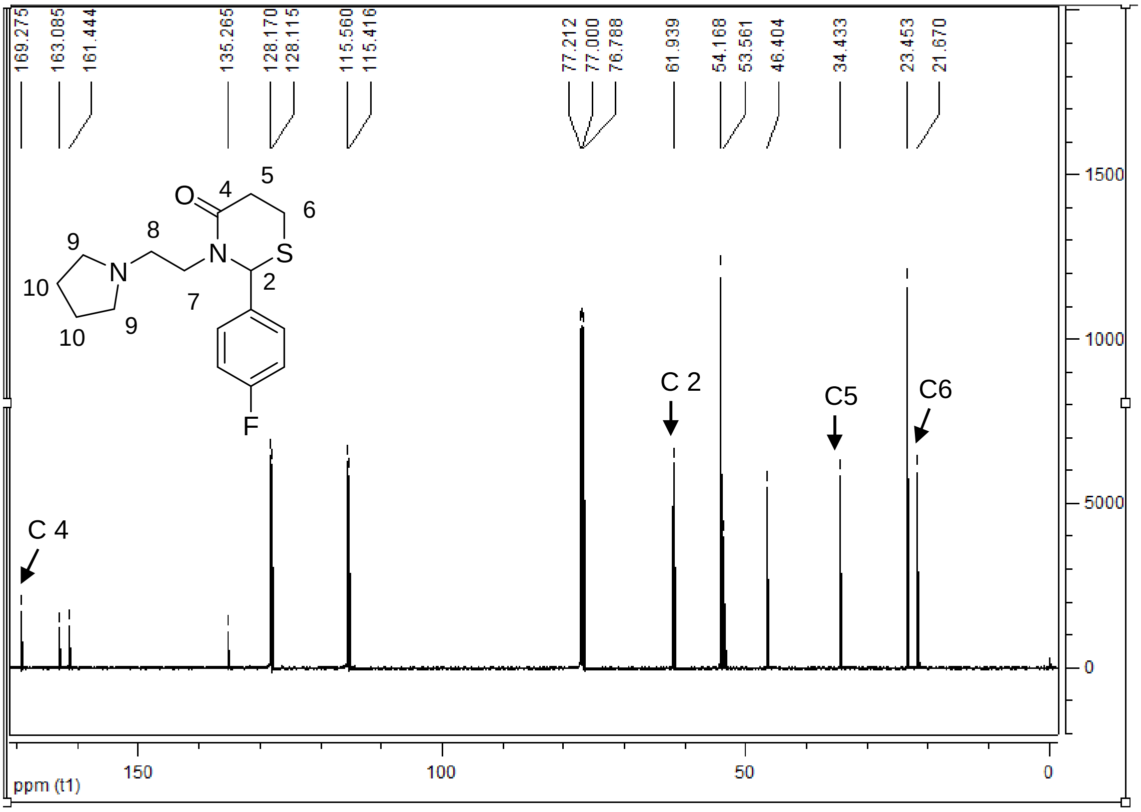

Figure S92: NMR ^{13}C spectrum of thiazinan-4-one **6d**.

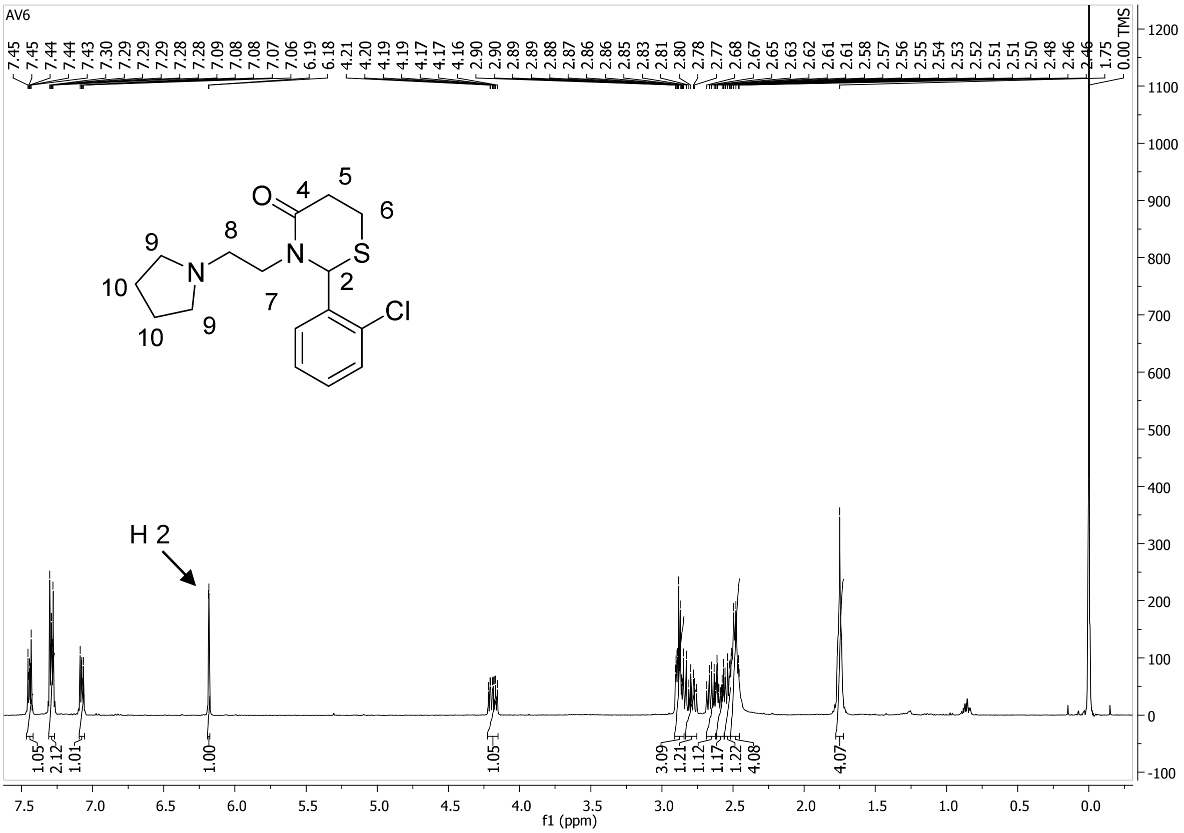

Figure S93: NMR ^{1}H spectrum of thiazinan-4-one **6e**.

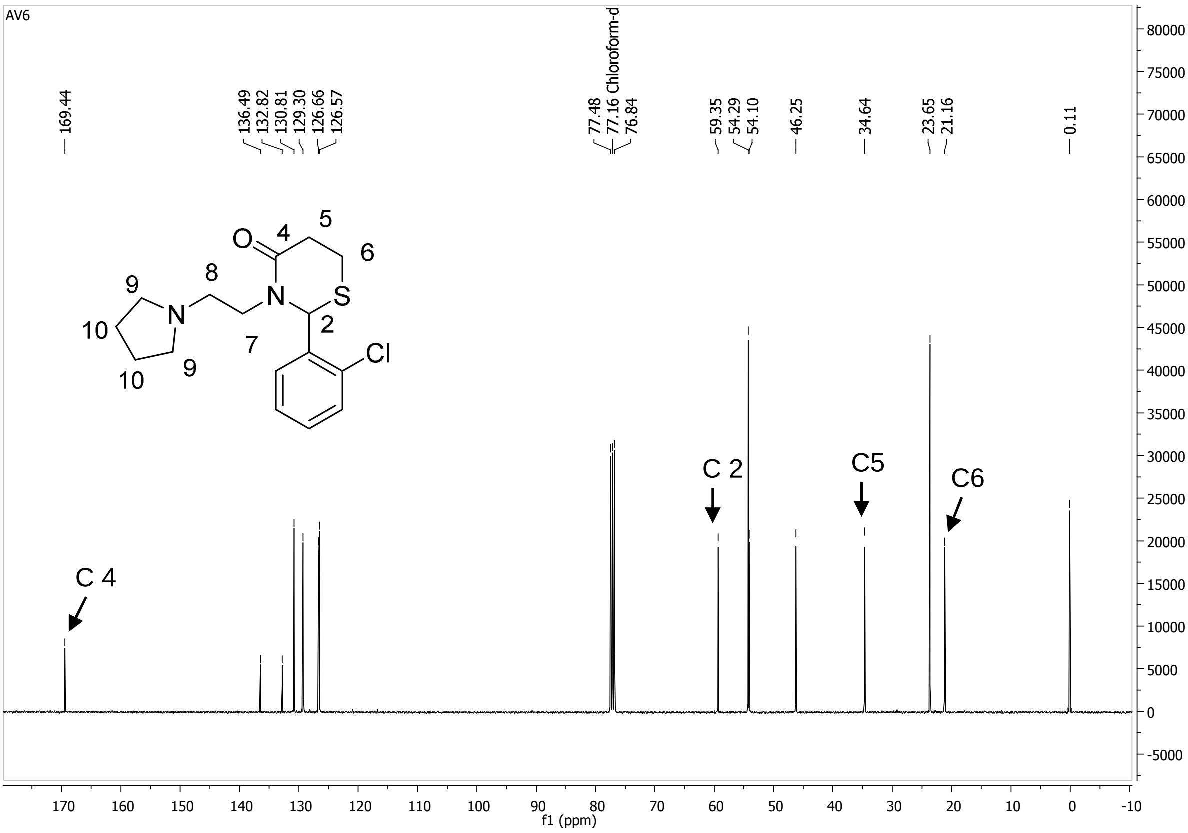

Figure S94: NMR ^{13}C spectrum of thiazinan-4-one **6e**.

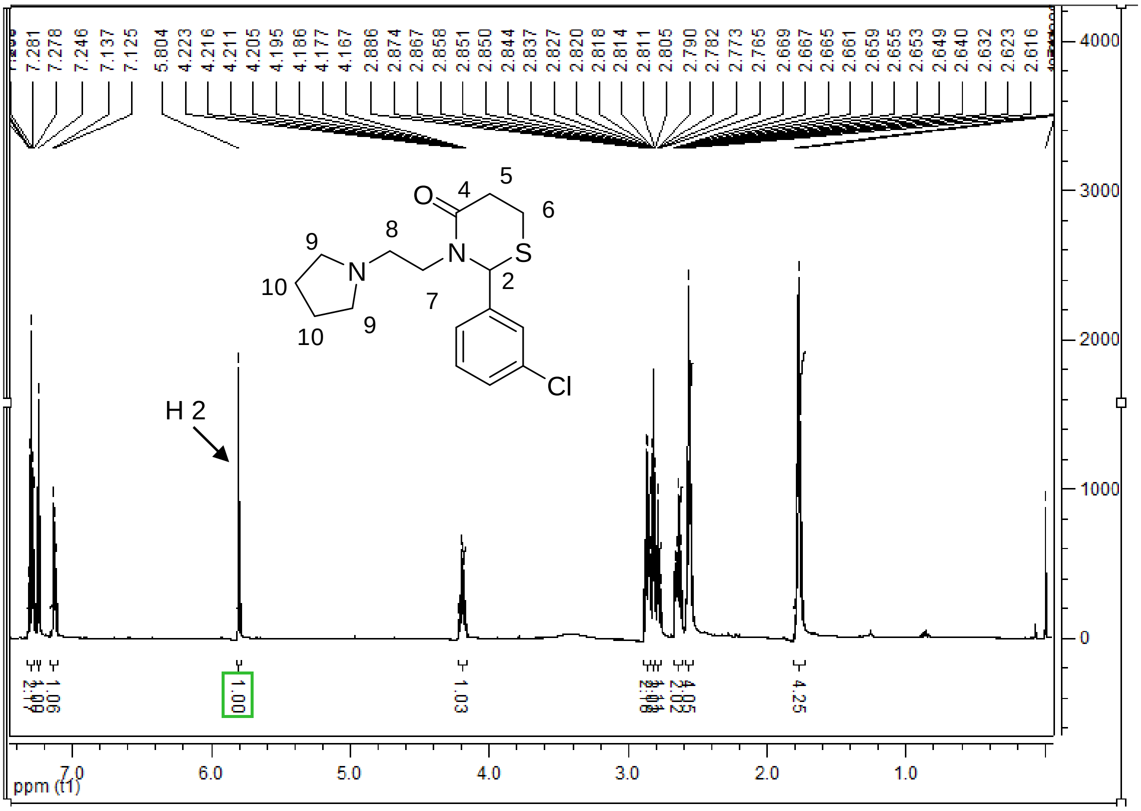

Figure S95: NMR ^{1}H spectrum of thiazinan-4-one **6f**.

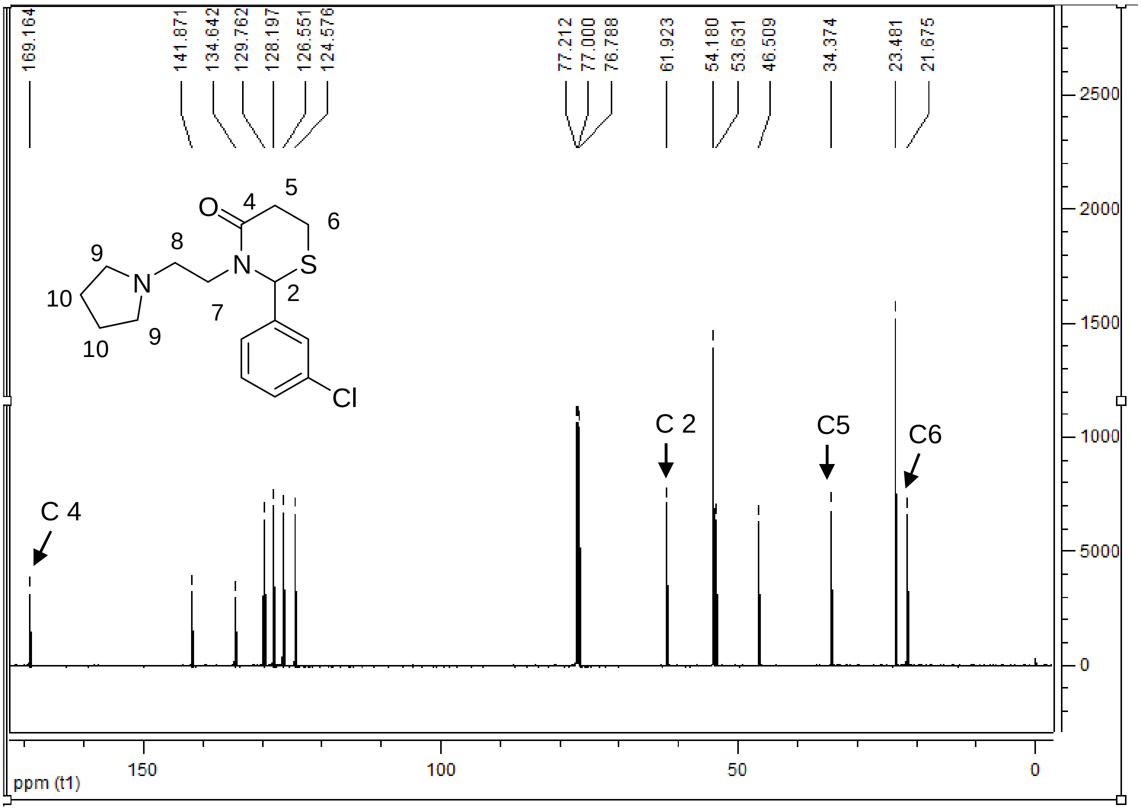

Figure S96: NMR ^{13}C spectrum of thiazinan-4-one **6f**.

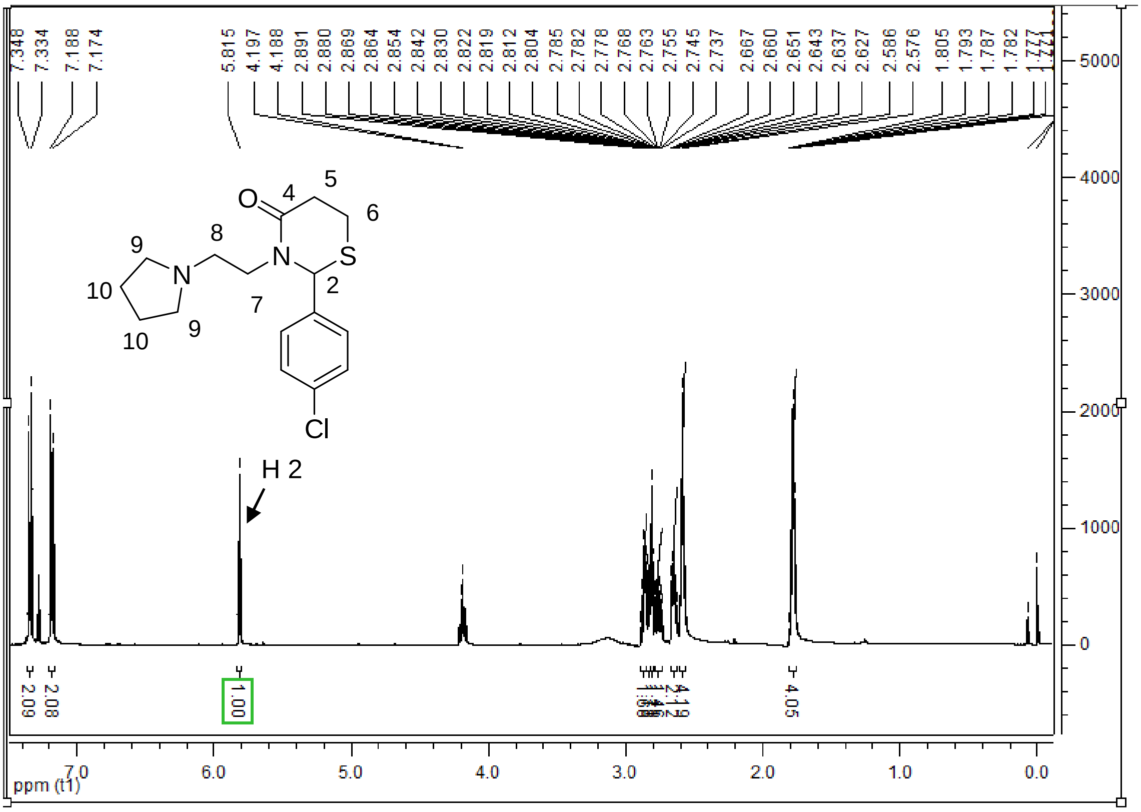

Figure S97: NMR ^{1}H spectrum of thiazinan-4-one **6g**.

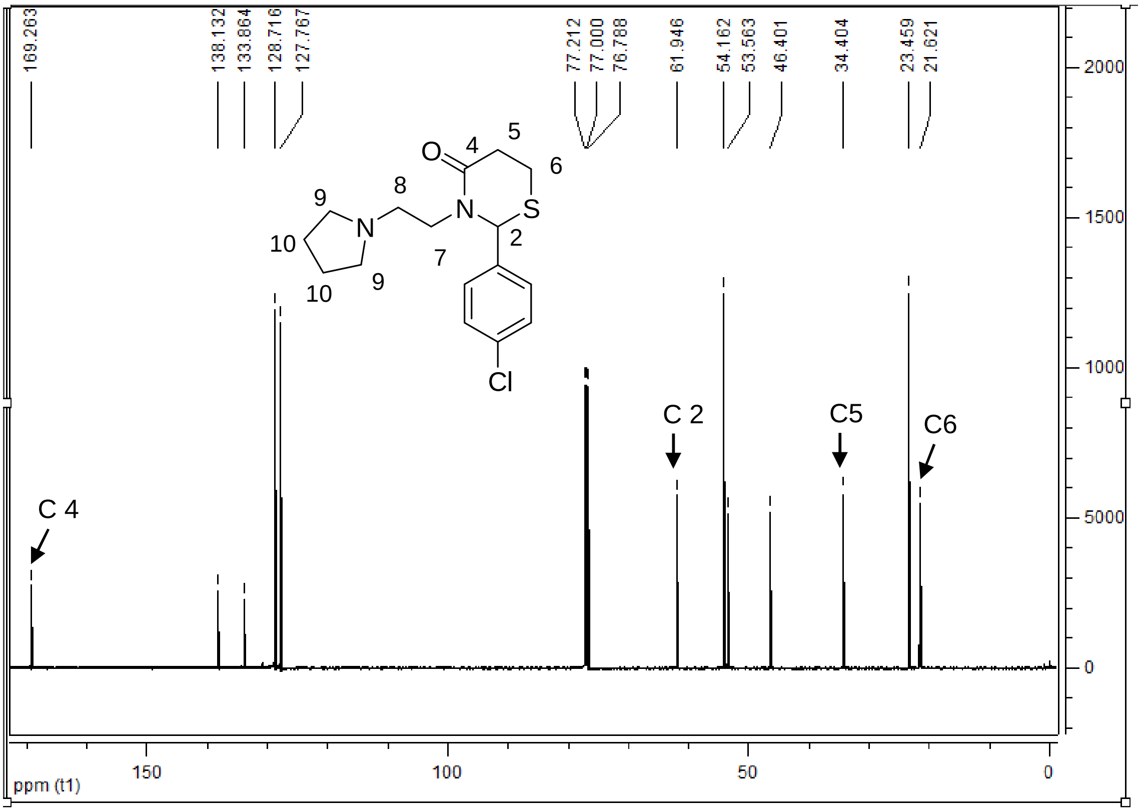

Figure S98: NMR ^{13}C spectrum of thiazinan-4-one **6g**.

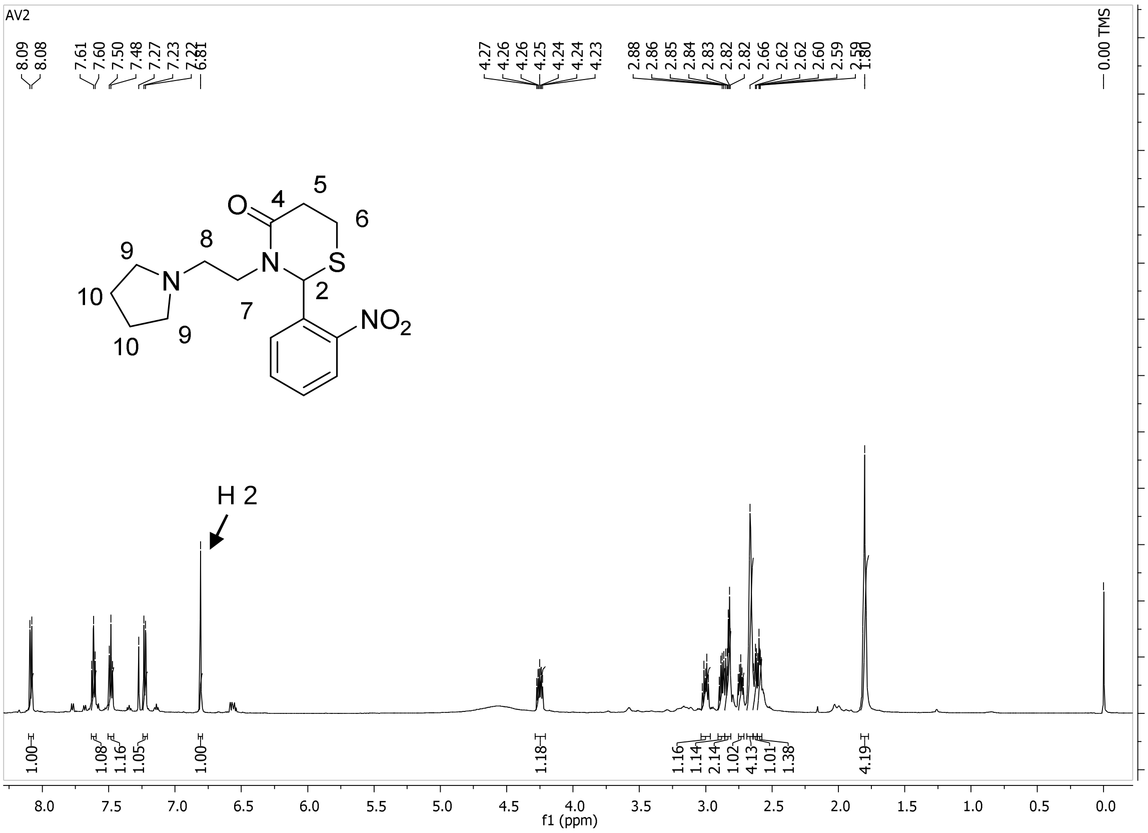

Figure S99: NMR ^{1}H spectrum of thiazinan-4-one **6h**.

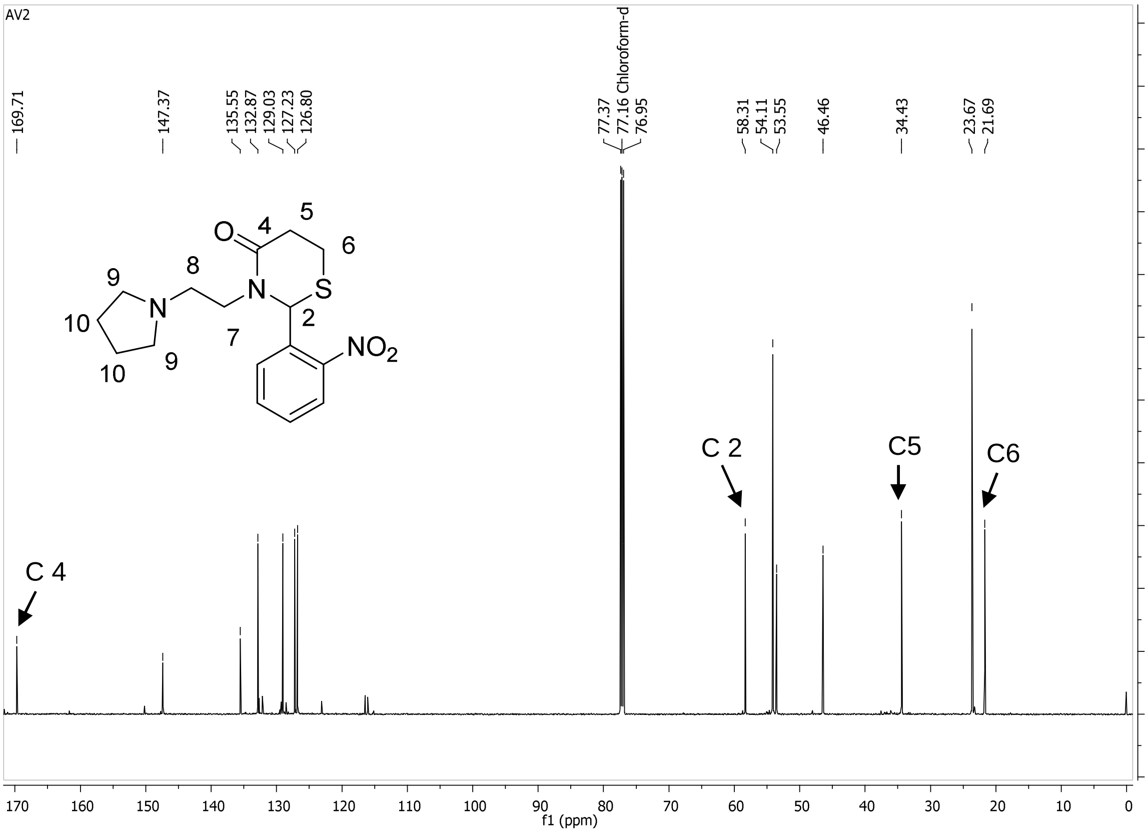

Figure S100: NMR ^{13}C spectrum of thiazinan-4-one **6h**.

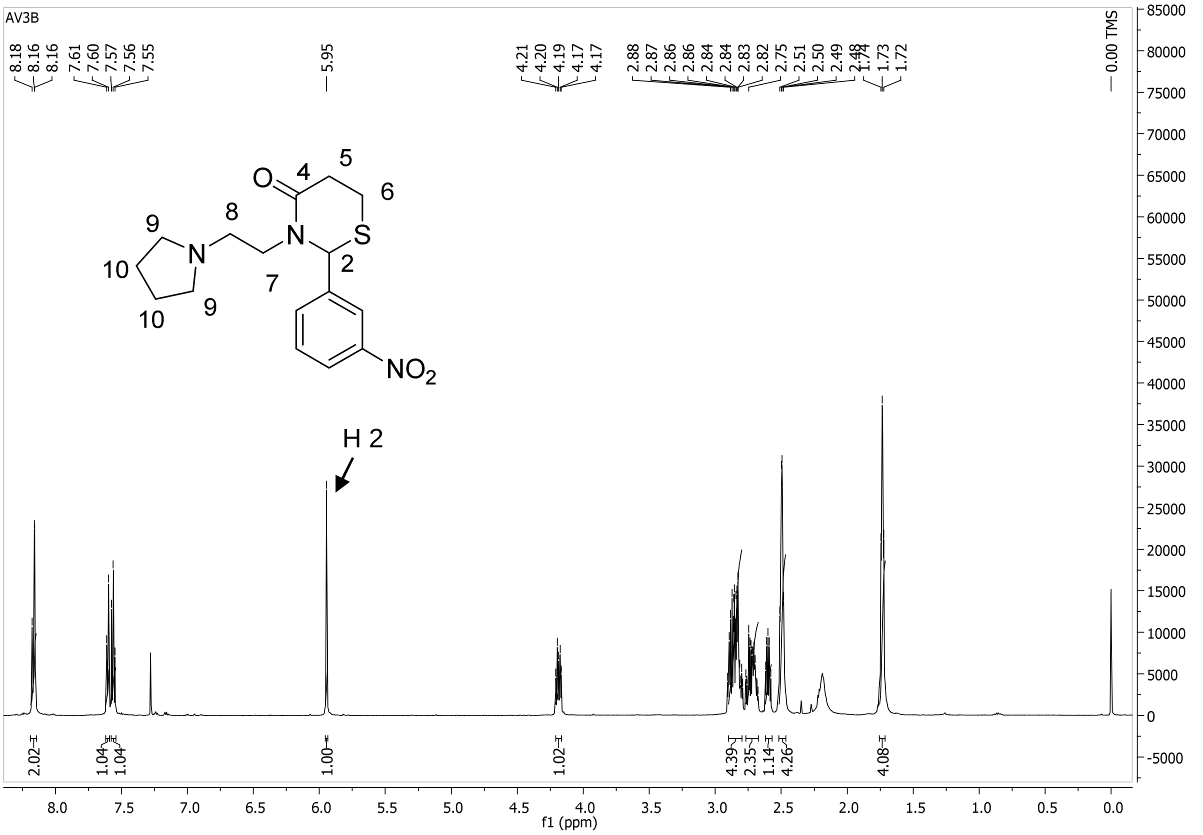

Figure S101: NMR ^{1}H spectrum of thiazinan-4-one **6i**.

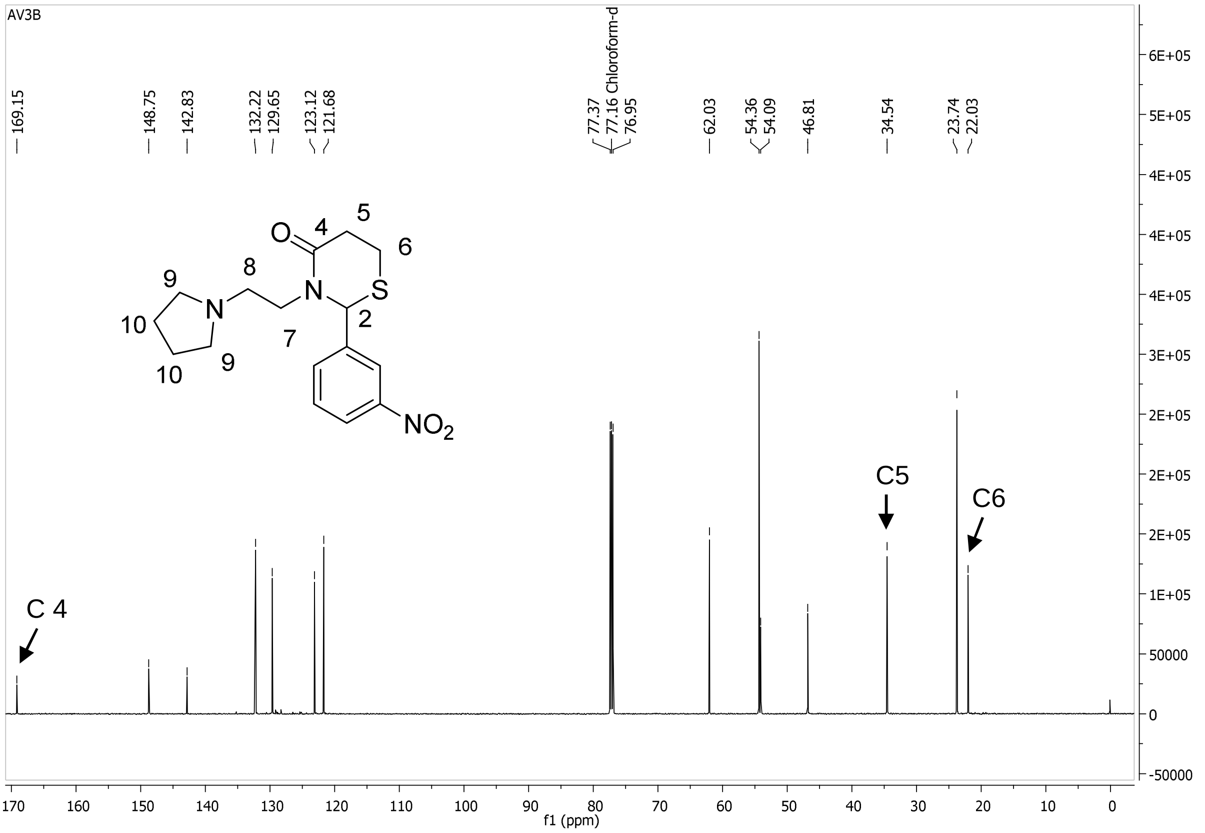

Figure S102: NMR ^{13}C spectrum of thiazinan-4-one **6i**.

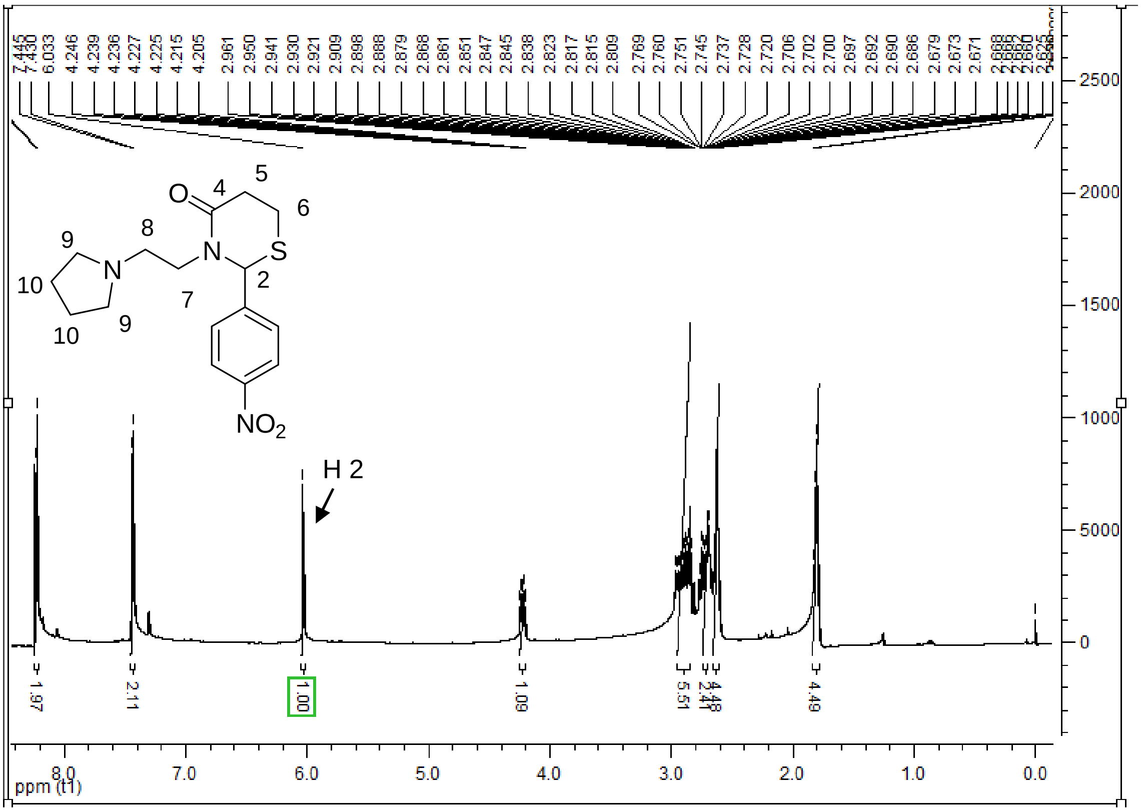

Figure S103: NMR ^{1}H spectrum of thiazinan-4-one **6j**.

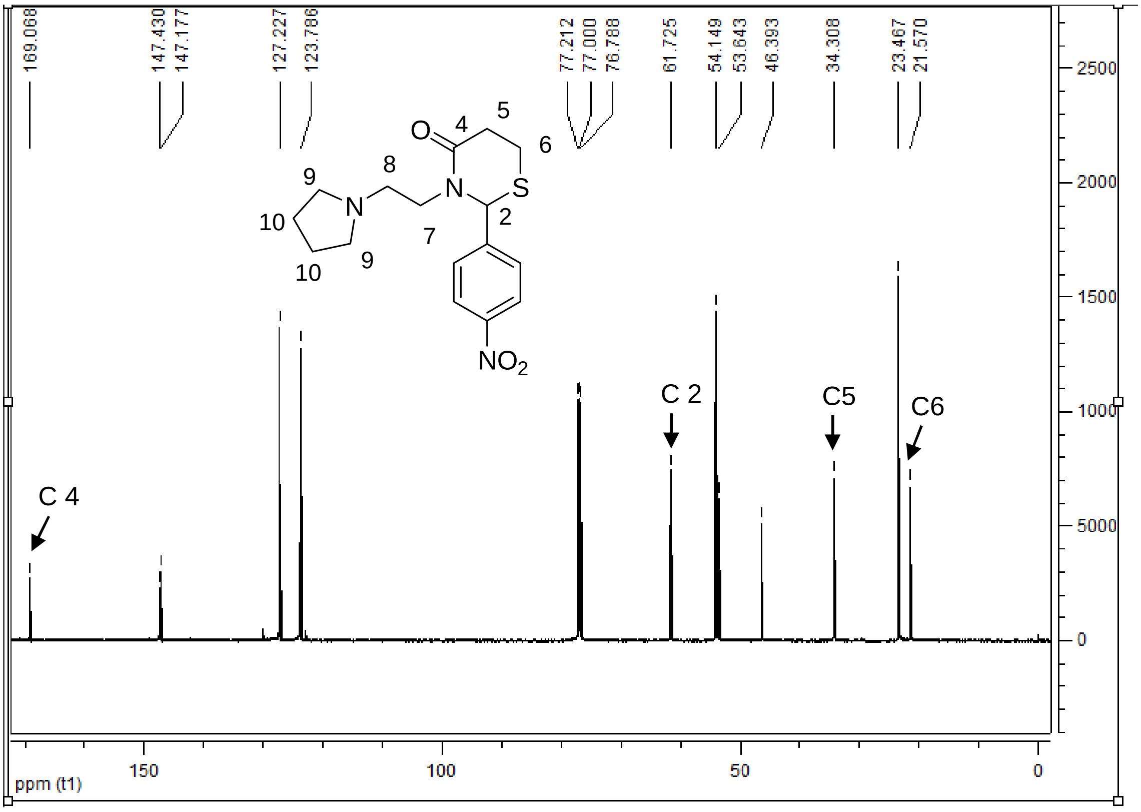

Figure S104: NMR ^{13}C spectrum of thiazinan-4-one **6j**.

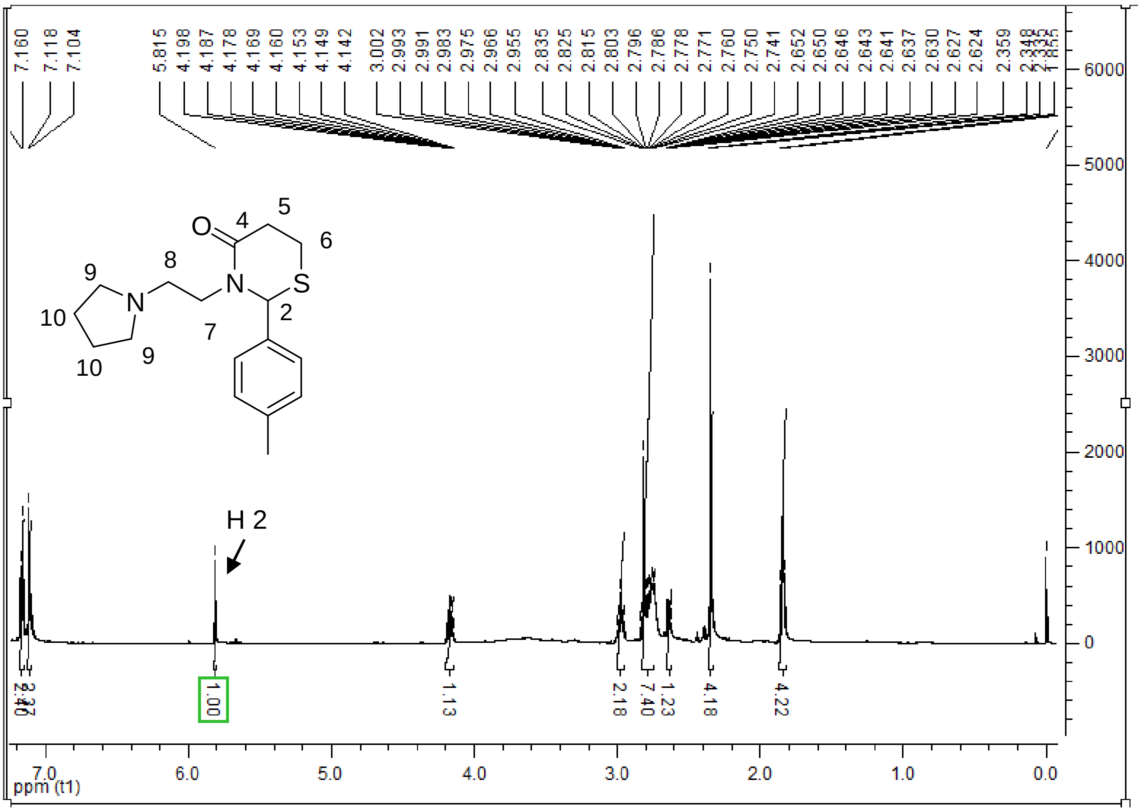

Figure S105: NMR ^{1}H spectrum of thiazinan-4-one **6k**.

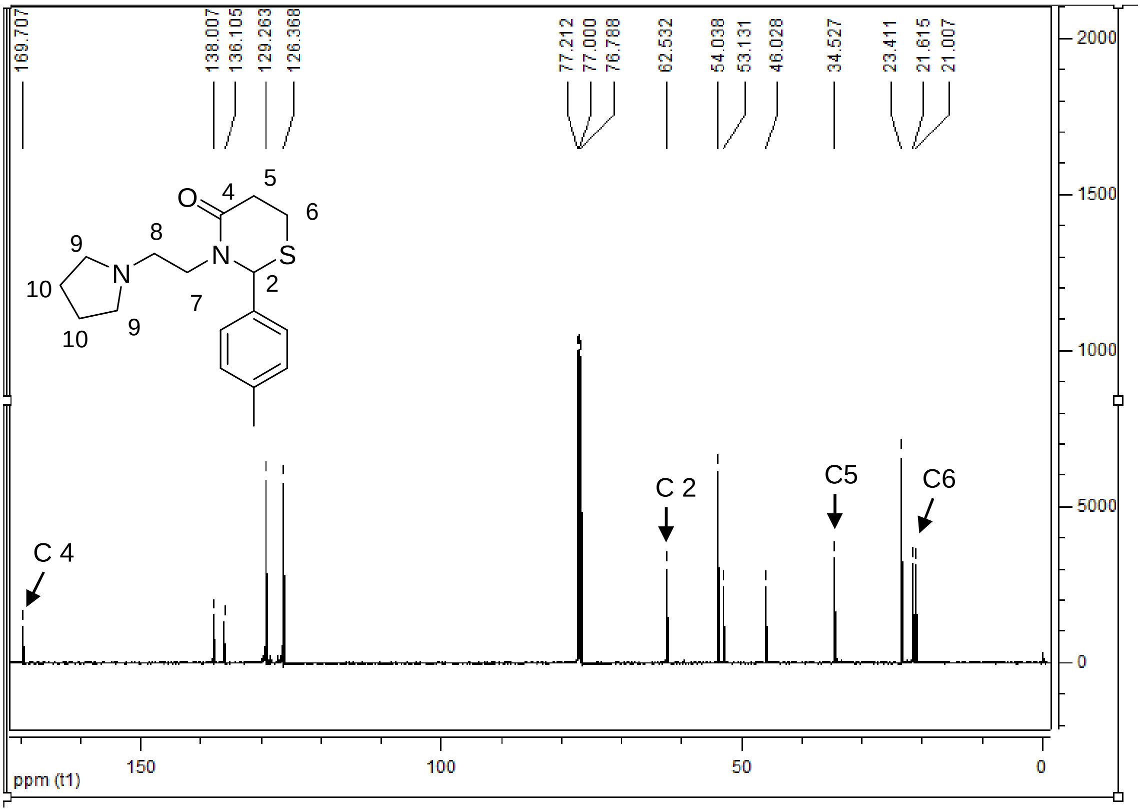

Figure S106: NMR ^{13}C spectrum of thiazinan-4-one **6k**.

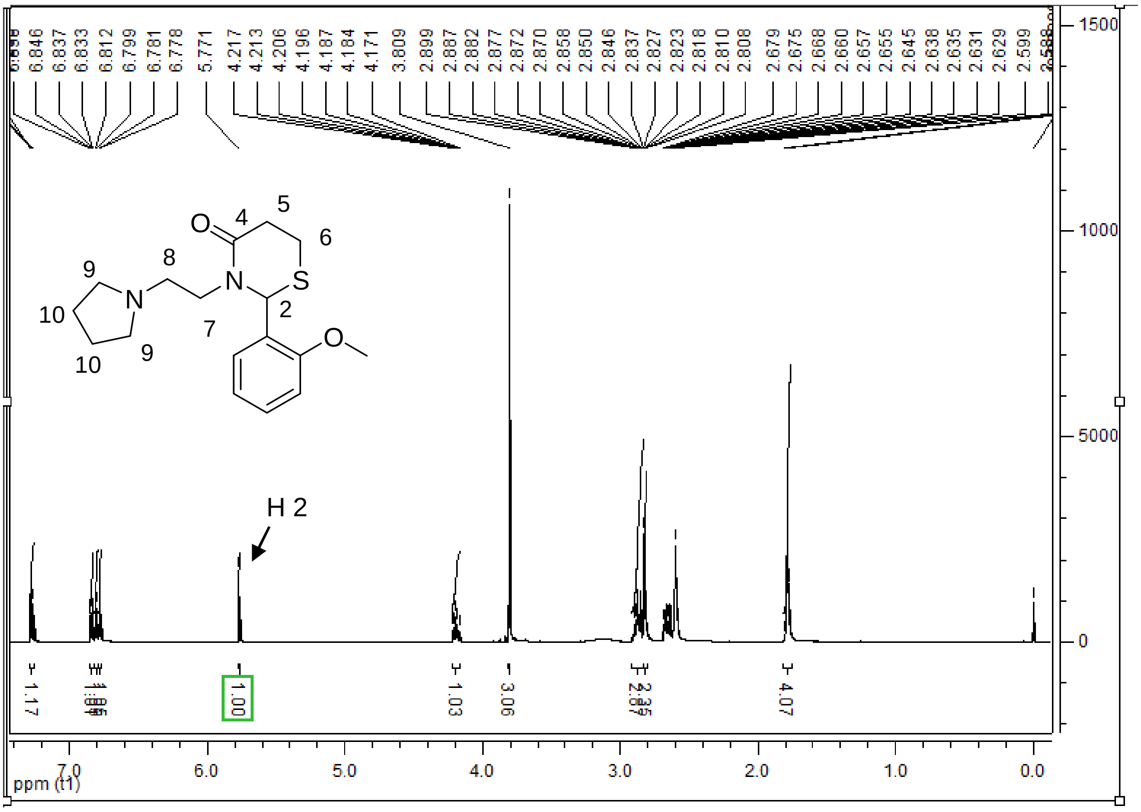

Figure S107: NMR ^{1}H spectrum of thiazinan-4-one **6l**.

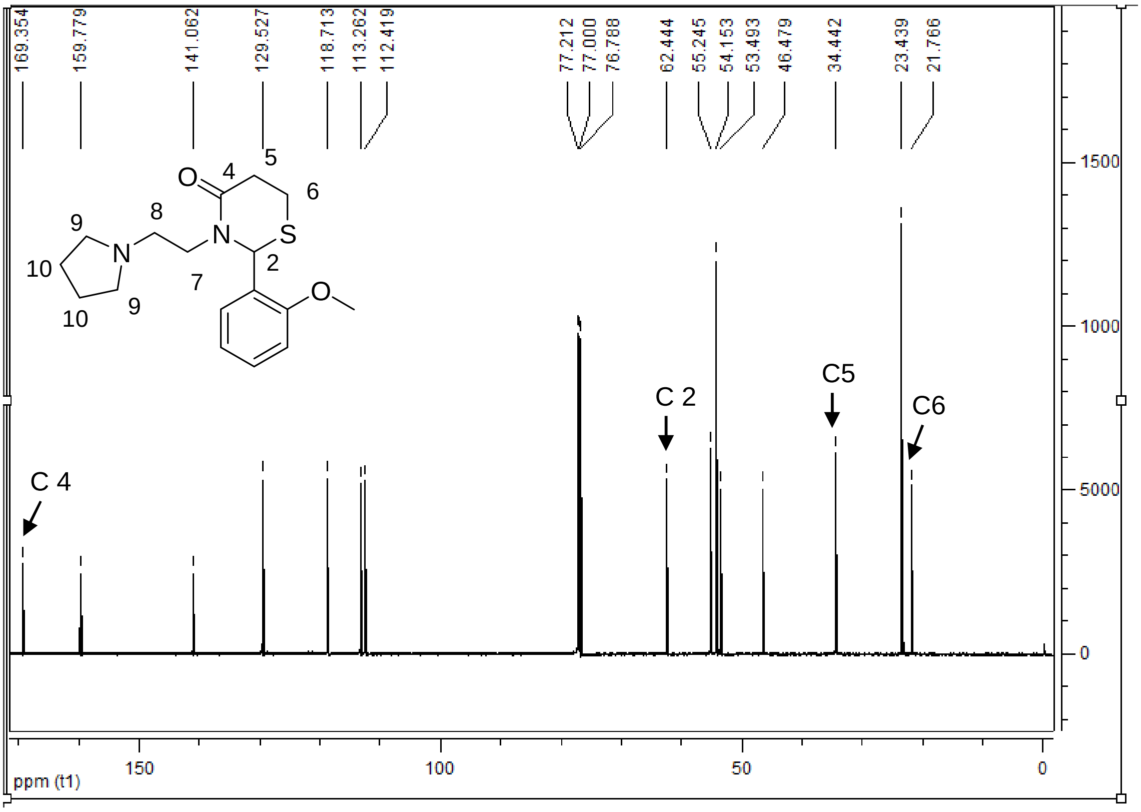

Figure S108: NMR ^{13}C spectrum of thiazinan-4-one **6l**.

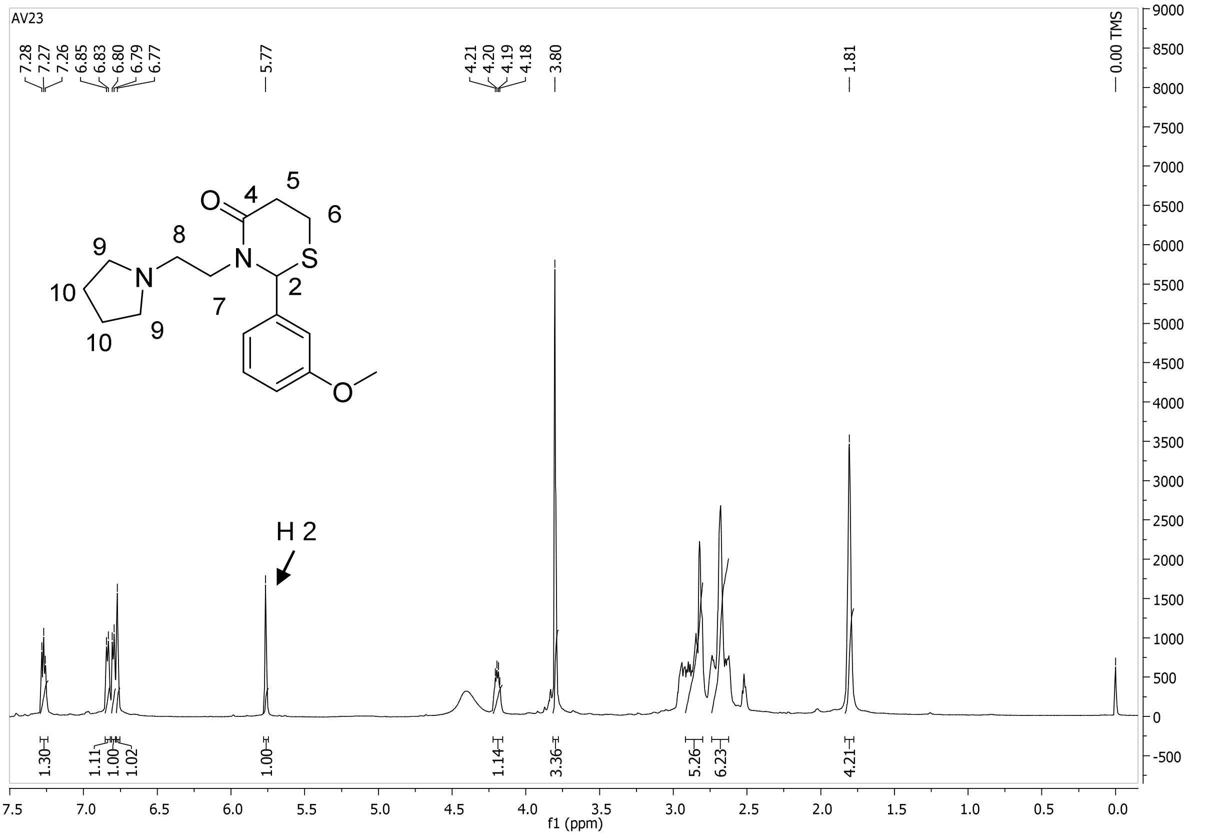

Figure S109: NMR ^{1}H spectrum of thiazinan-4-one **6m**.

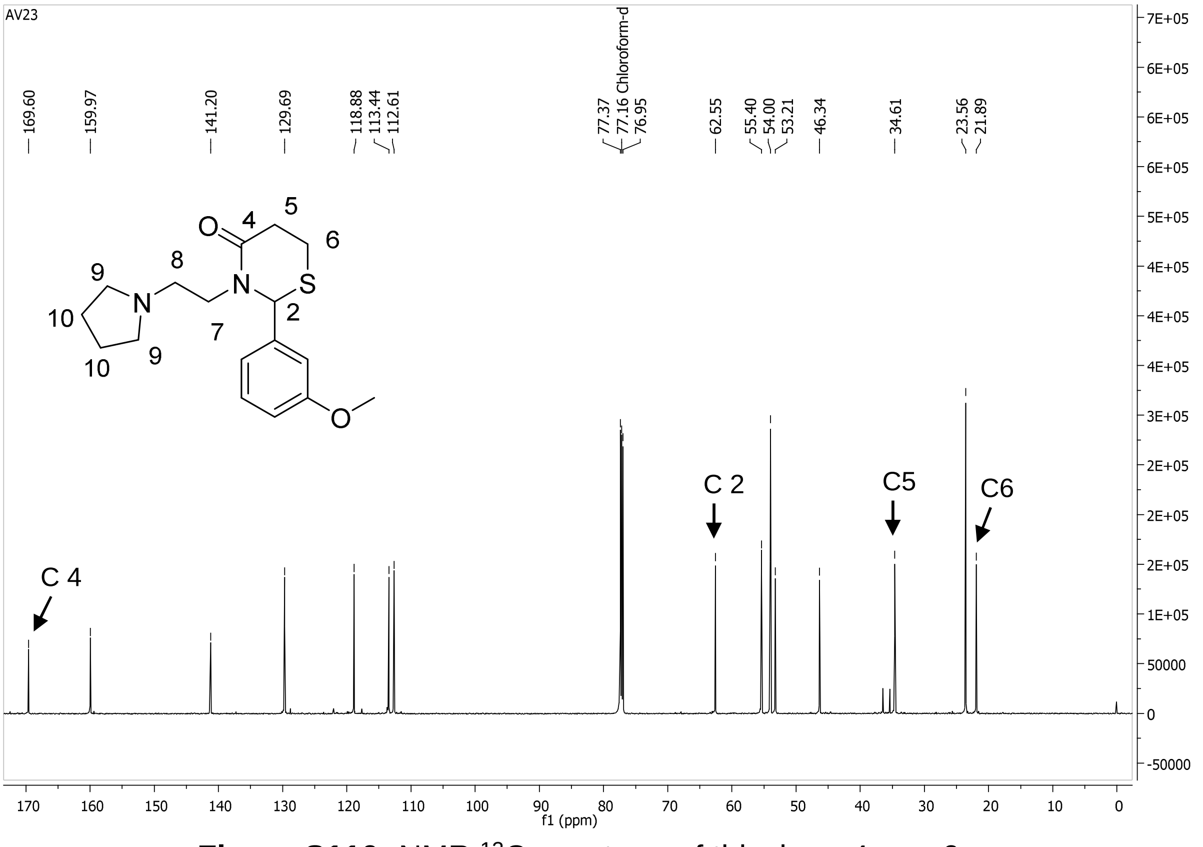

Figure S110: NMR ^{13}C spectrum of thiazinan-4-one **6m**.

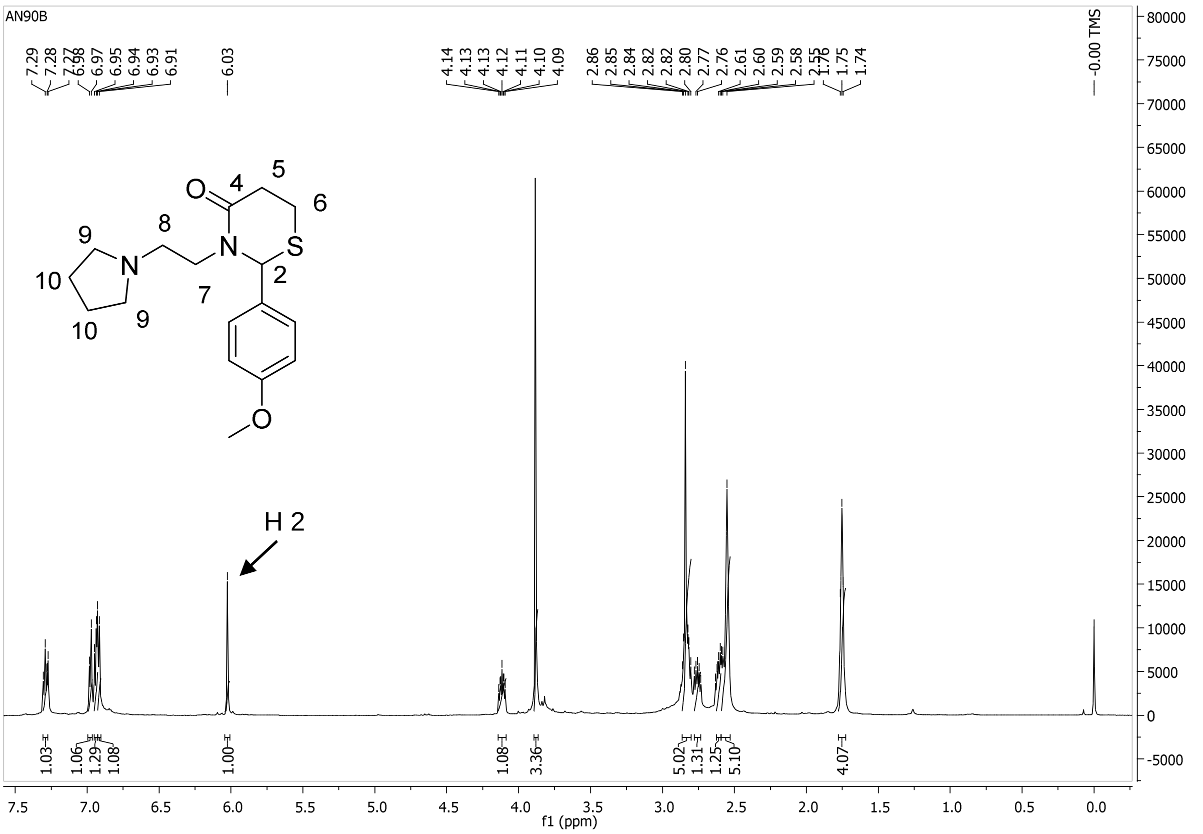

Figure S111: NMR ^{1}H spectrum of thiazinan-4-one **6n**.

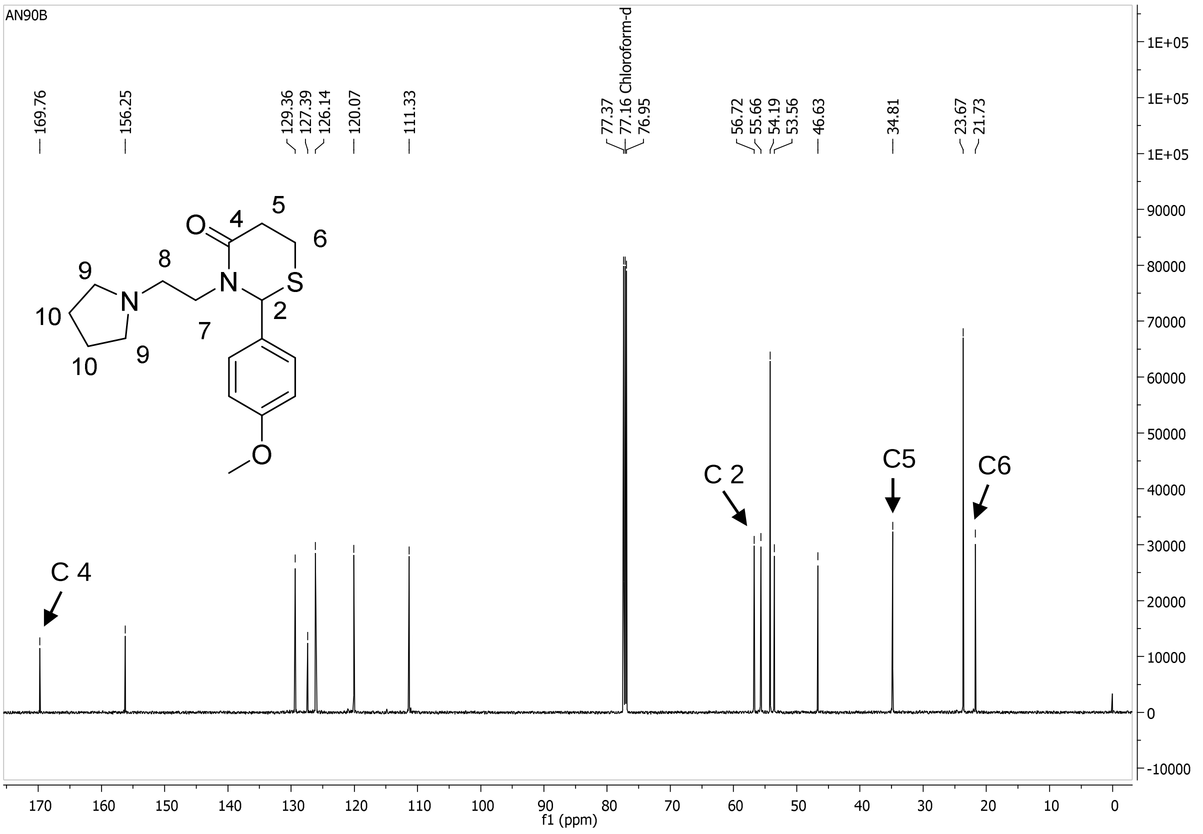

Figure S112: NMR ^{13}C spectrum of thiazinan-4-one **6n**.

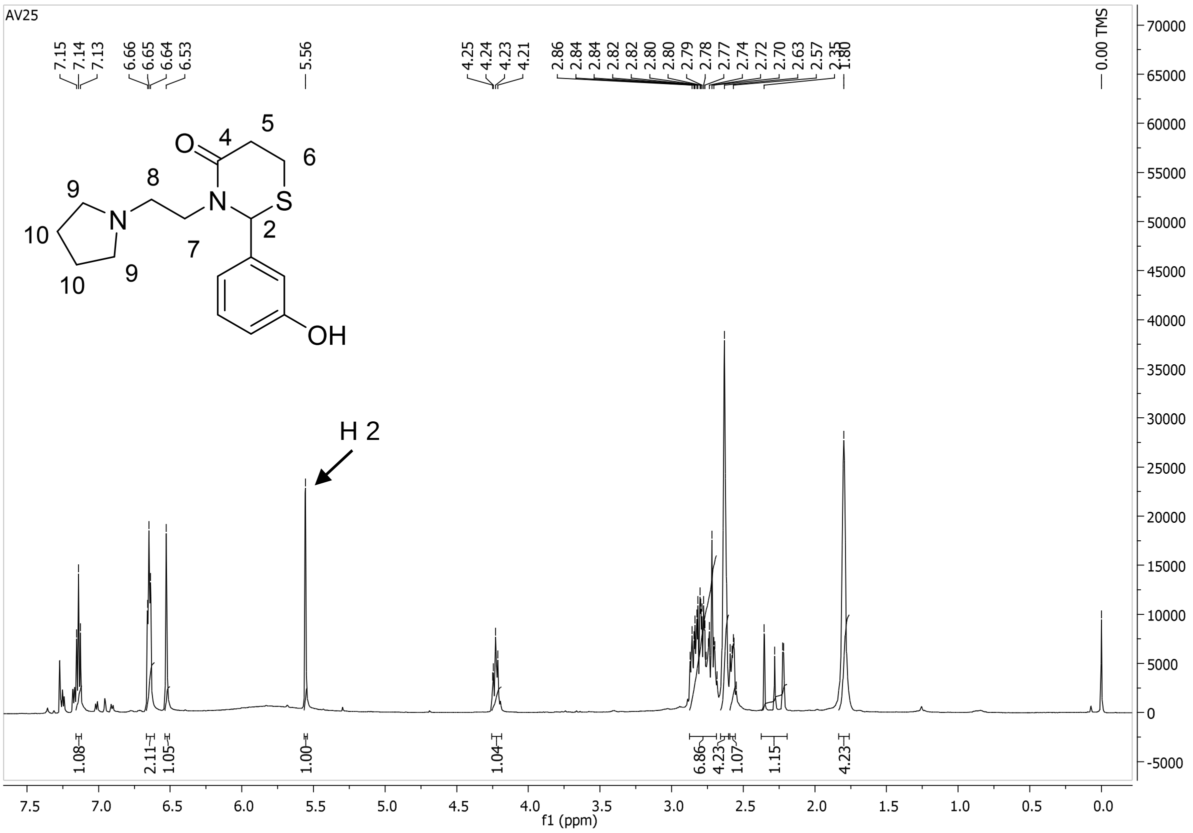

Figure S113: NMR ^{1}H spectrum of thiazinan-4-one **6o**.

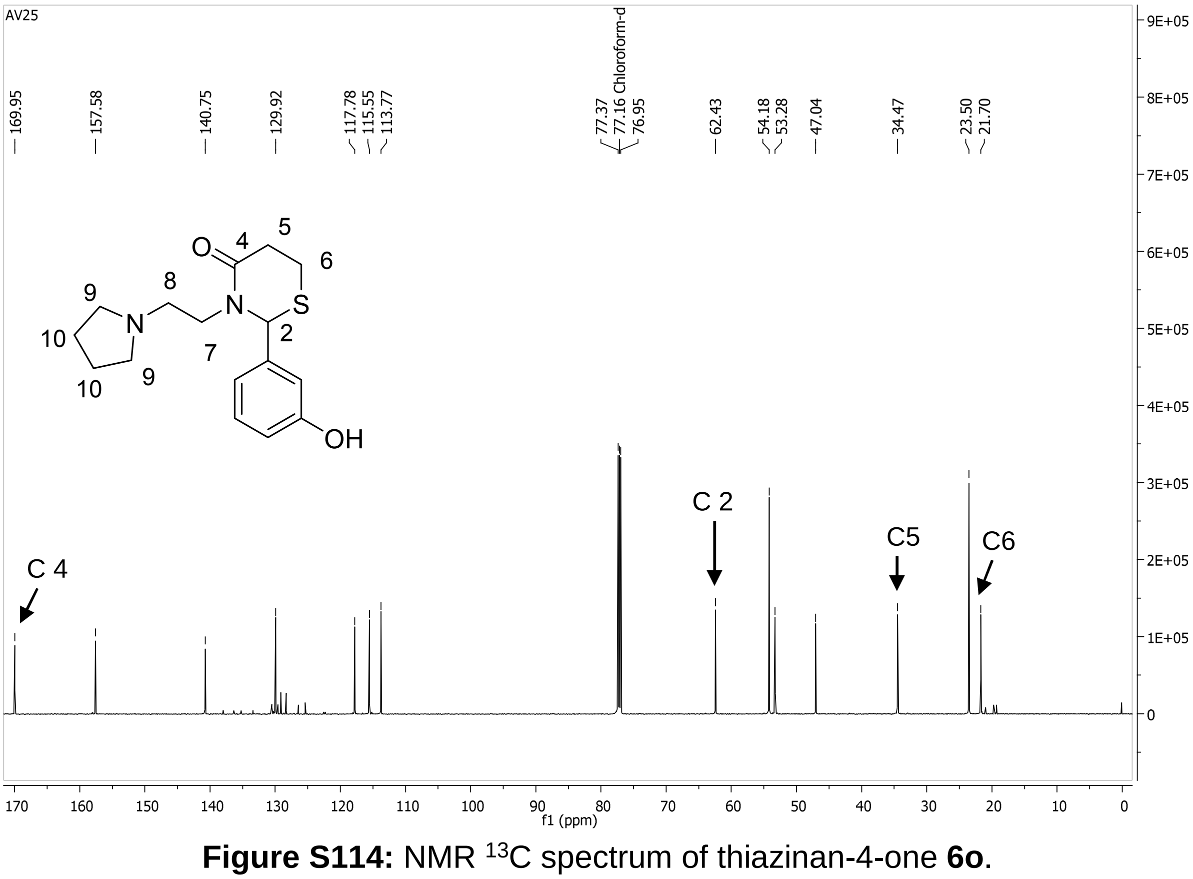

Figure S114: NMR ^{13}C spectrum of thiazinan-4-one **6o**.

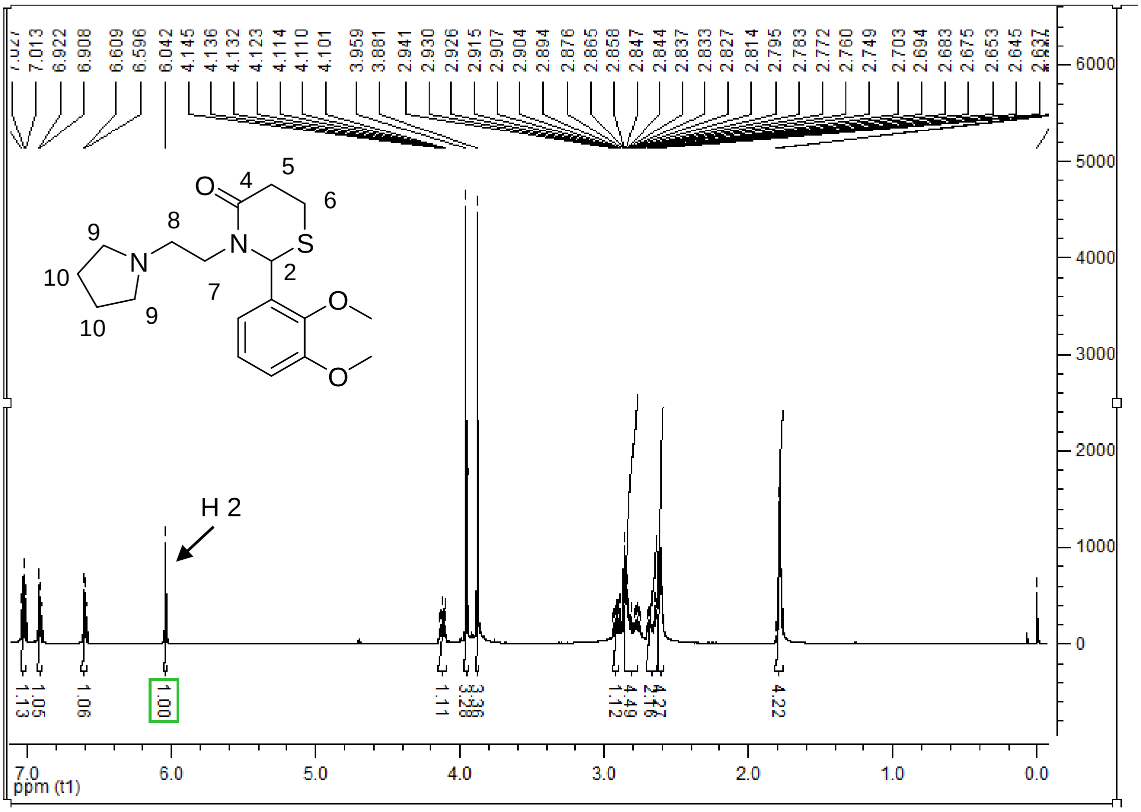

Figure S115: NMR ^{1}H spectrum of thiazinan-4-one **6p**.

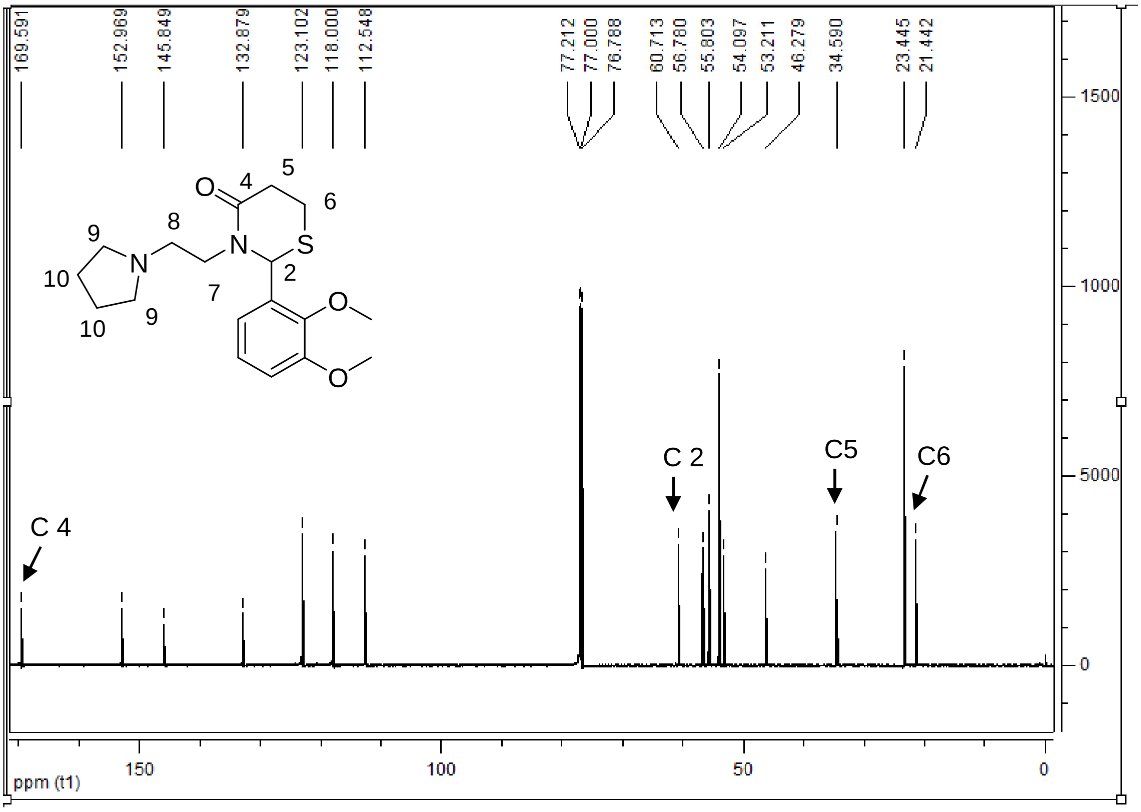

Figure S116: NMR ^{13}C spectrum of thiazinan-4-one **6p**.

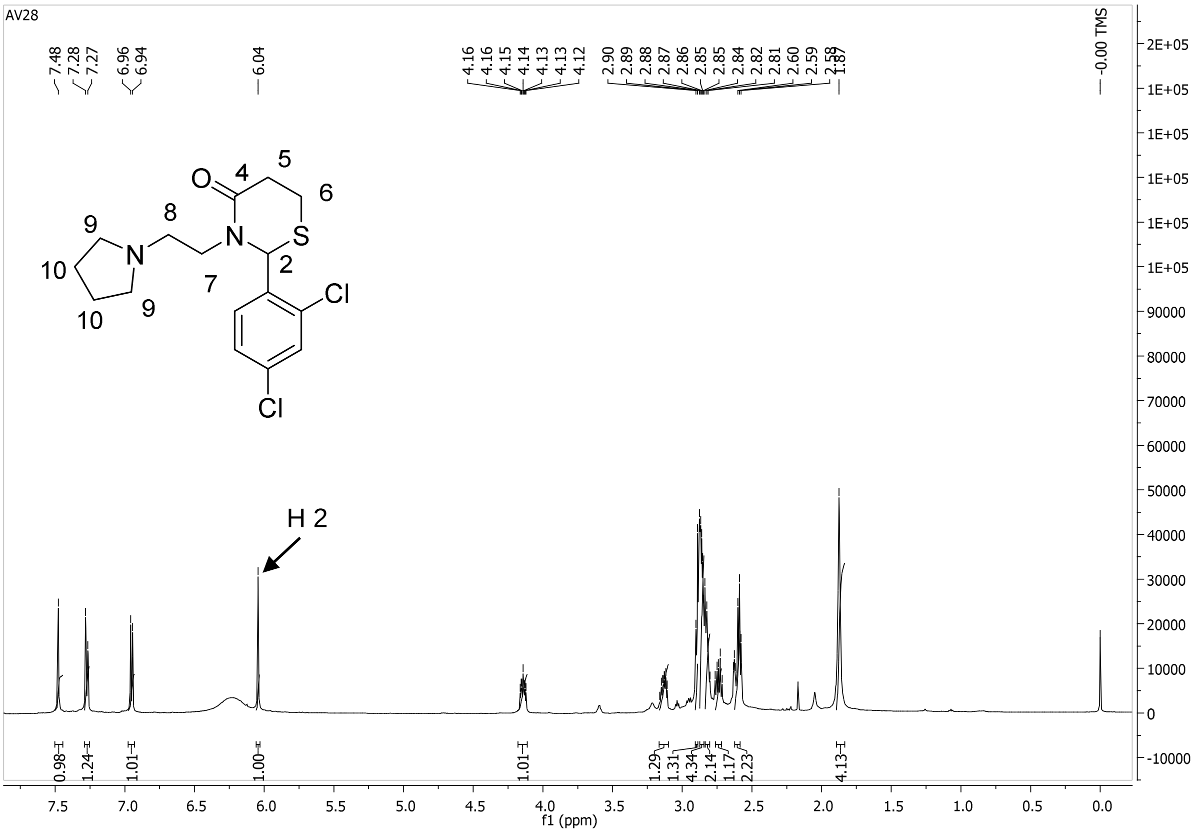

Figure S117: NMR ^{1}H spectrum of thiazinan-4-one **6q**.

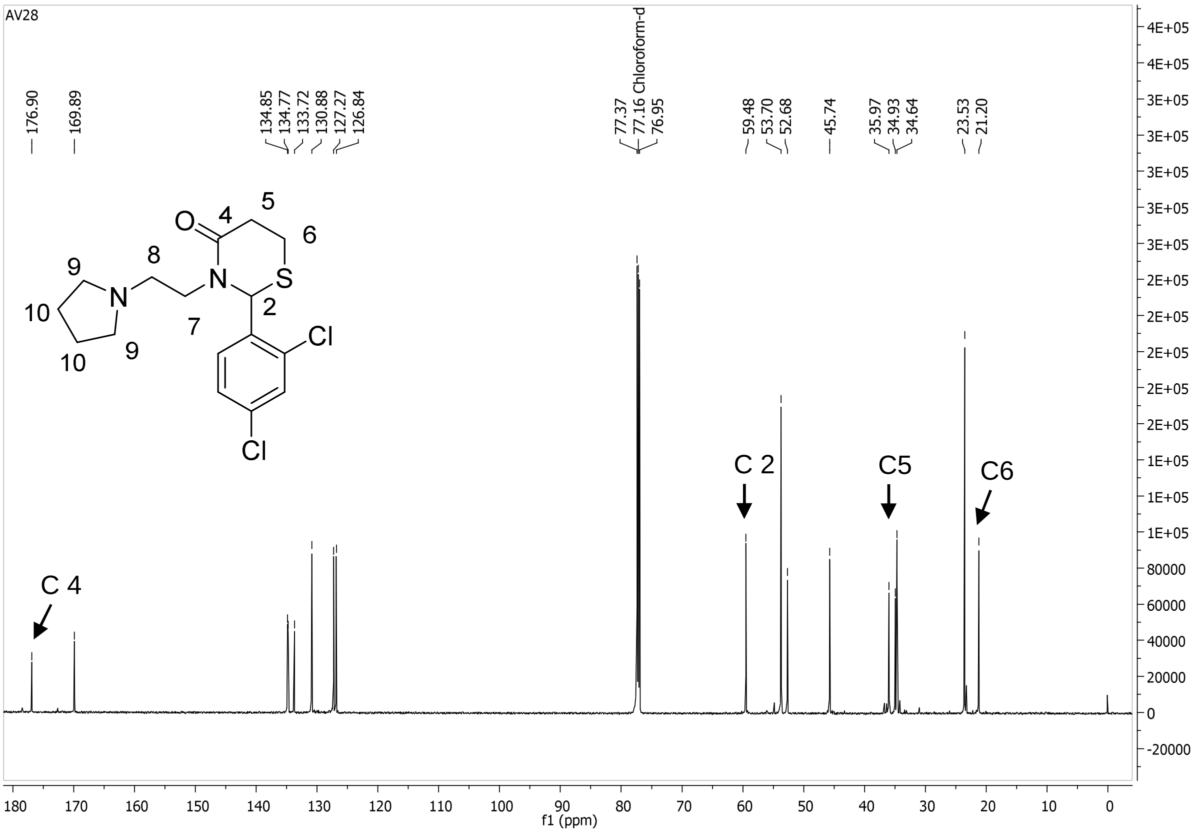

Figure S118: NMR ^{13}C spectrum of thiazinan-4-one **6q**.

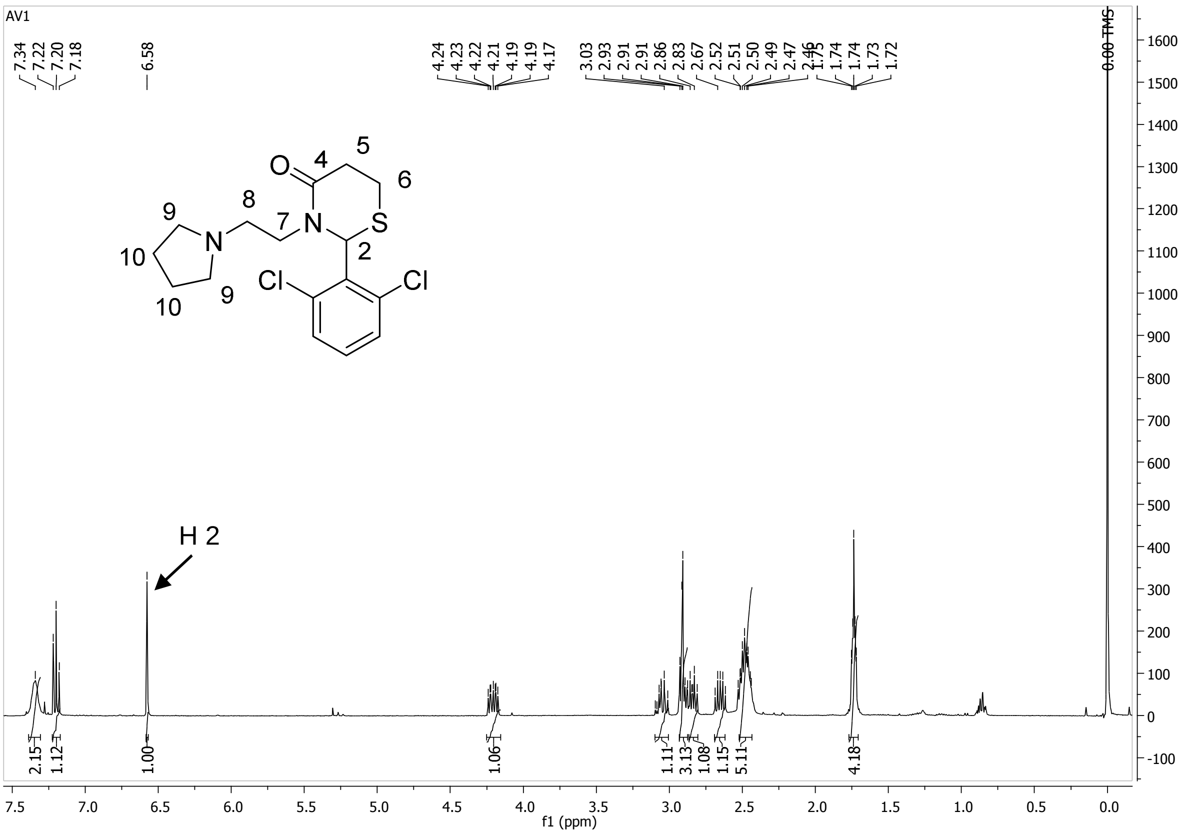

Figure S119: NMR ^{1}H spectrum of thiazinan-4-one **6r**.

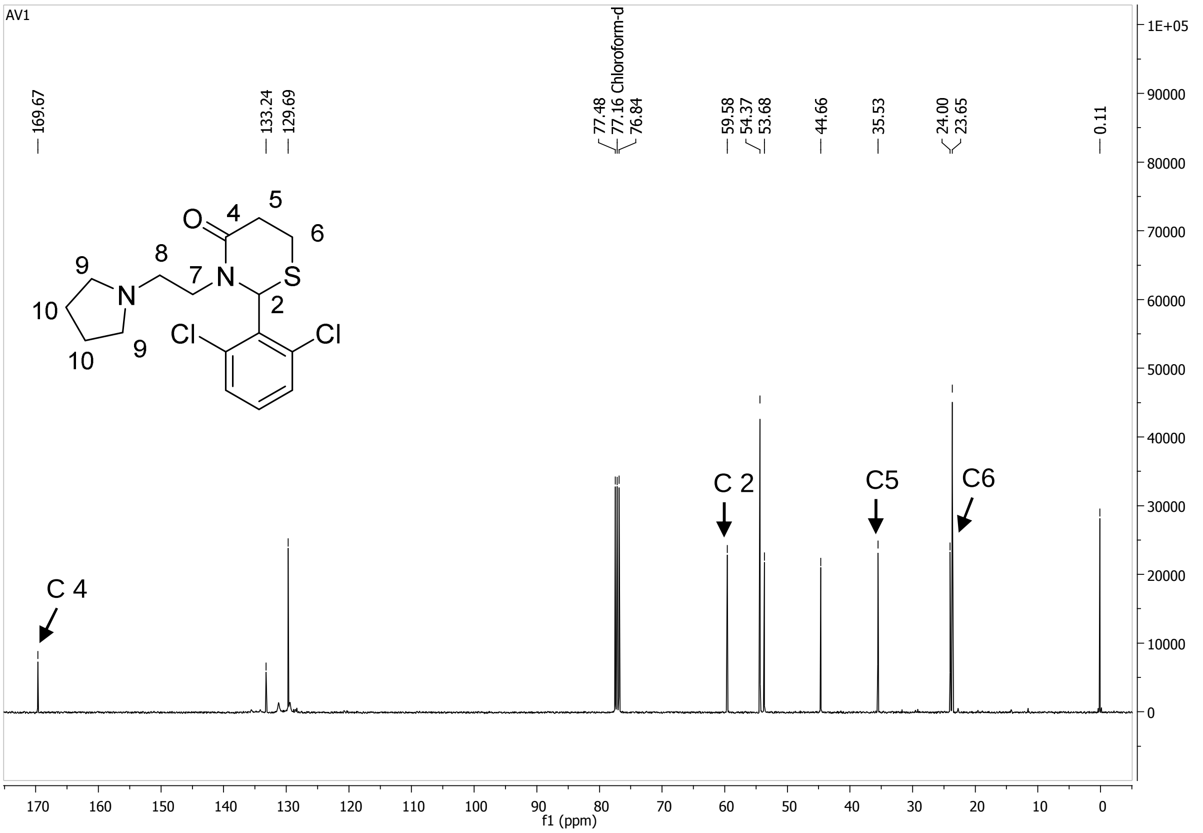

Figure S119: NMR ^{13}C spectrum of thiazinan-4-one **6r**.

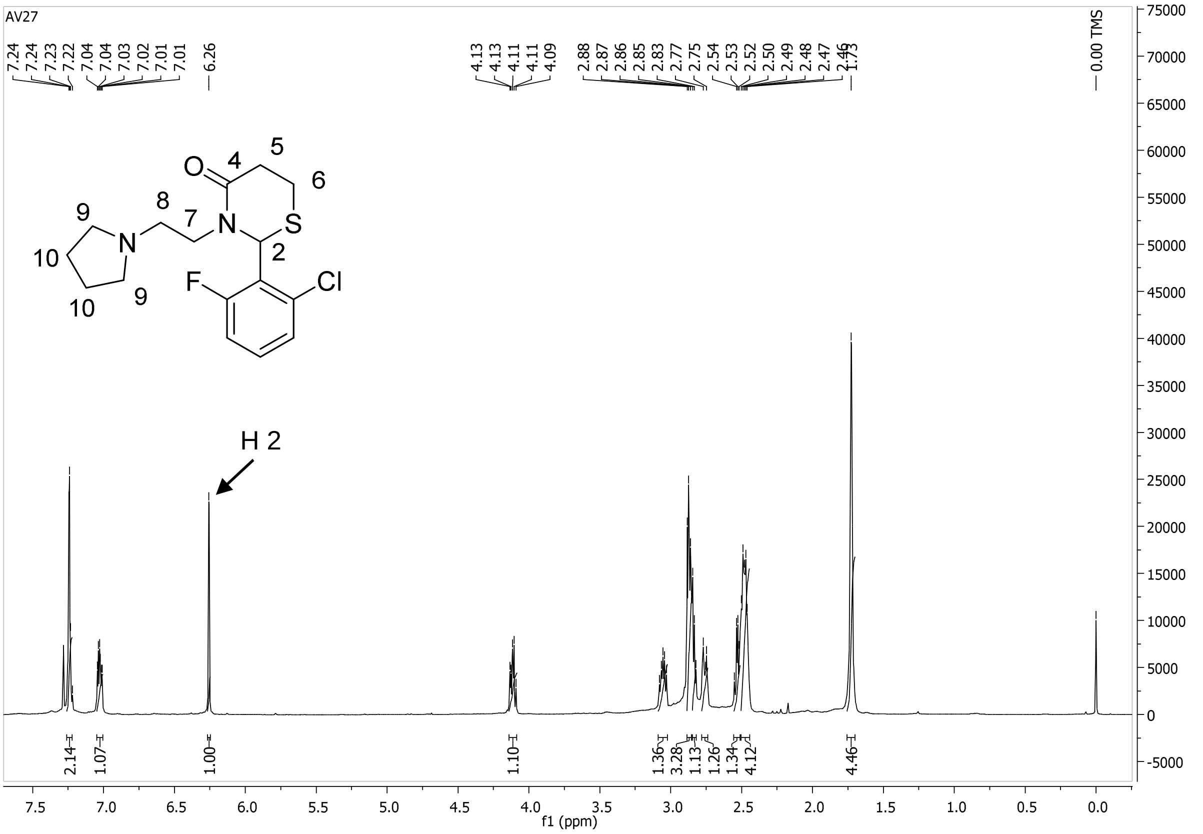

Figure S119: NMR ^{1}H spectrum of thiazinan-4-one **6s**.

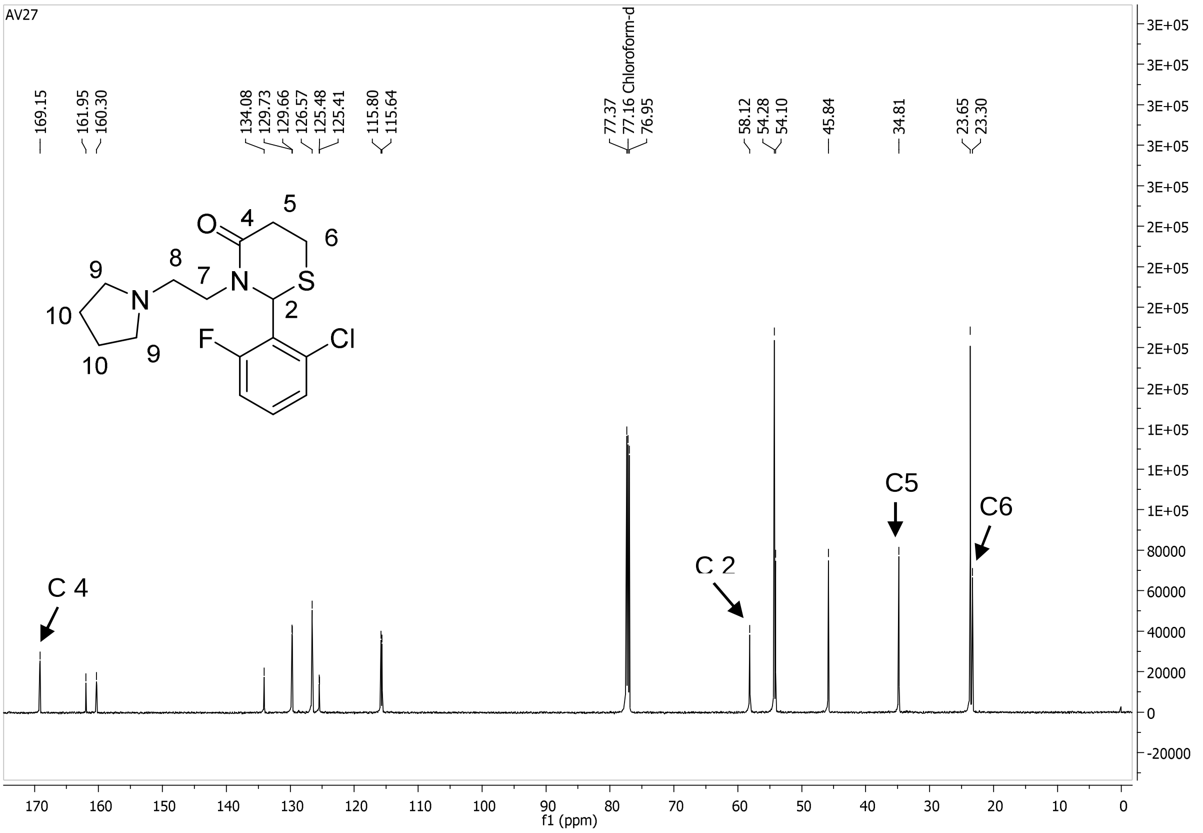

Figure S120: NMR ^{13}C spectrum of thiazinan-4-one **6s**.

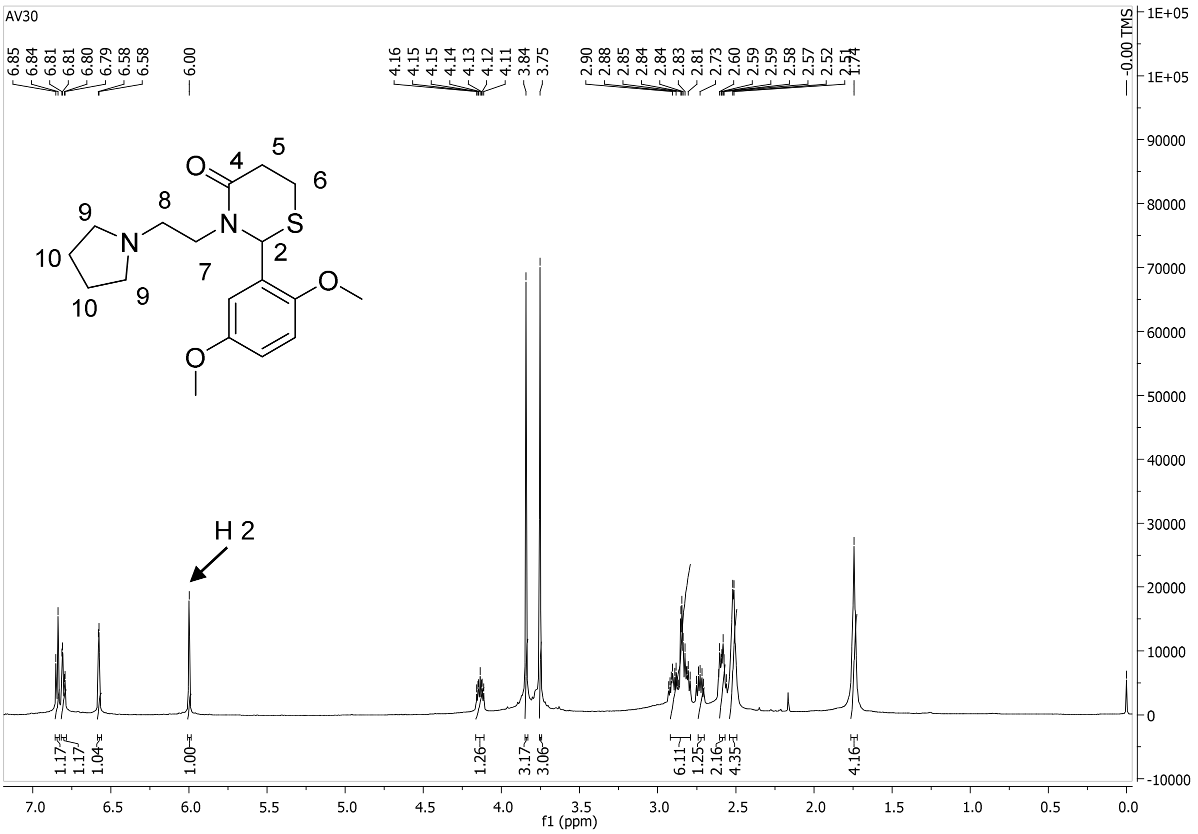

Figure S121: NMR ^{1}H spectrum of thiazinan-4-one **6v**.

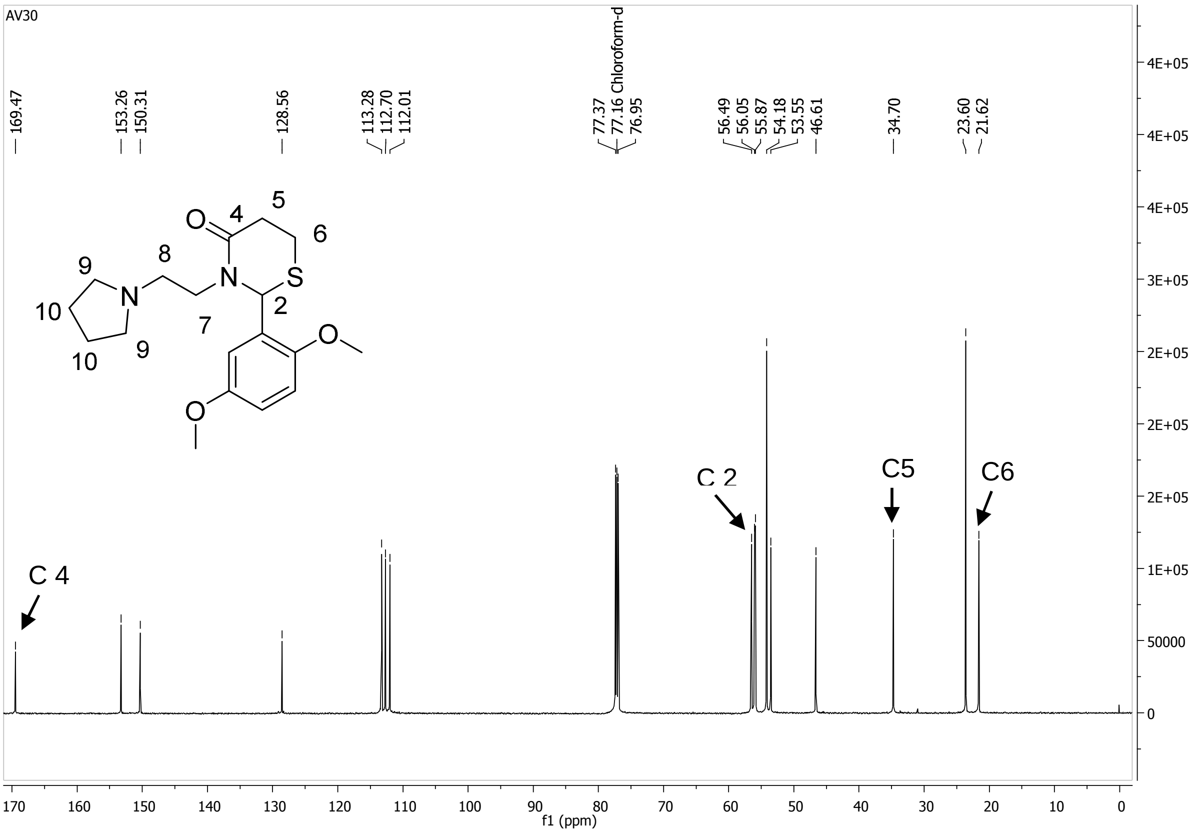

Figure S121: NMR ^{13}C spectrum of thiazinan-4-one **6v**.

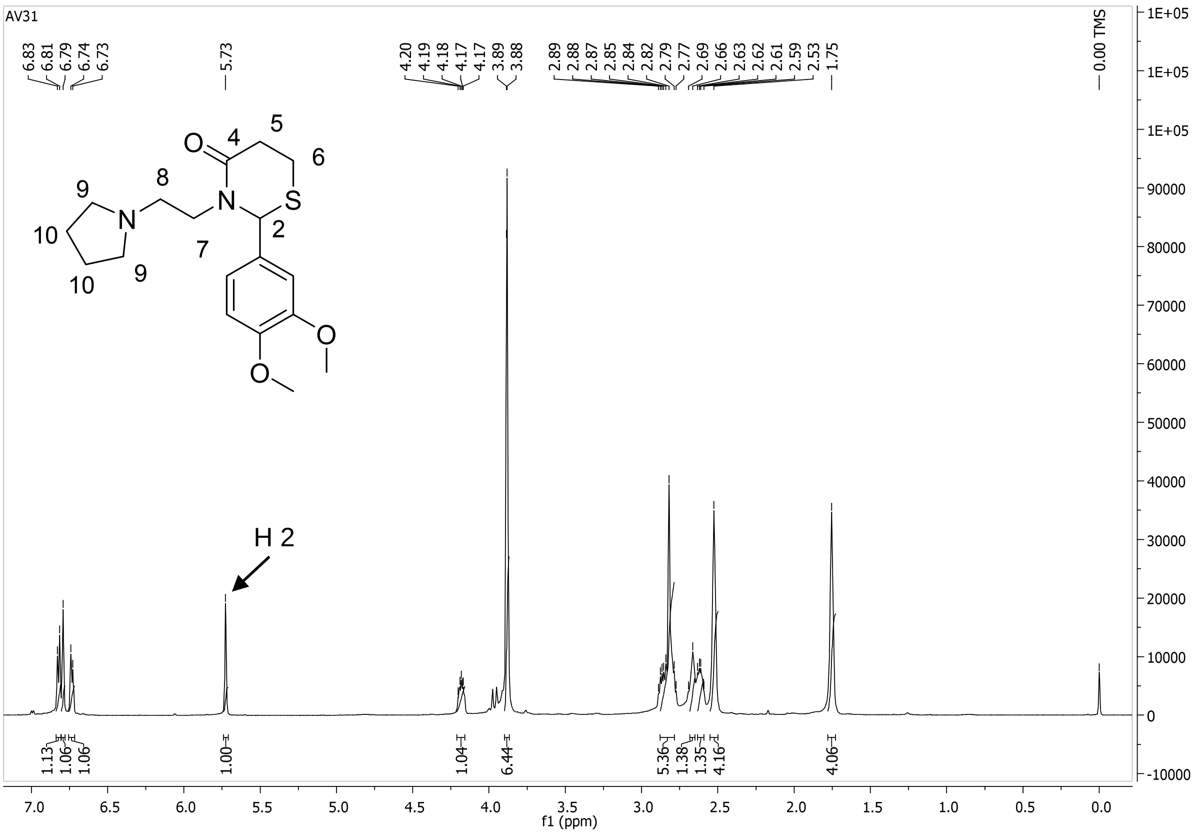

Figure S122: NMR ^{1}H spectrum of thiazinan-4-one **6x**.

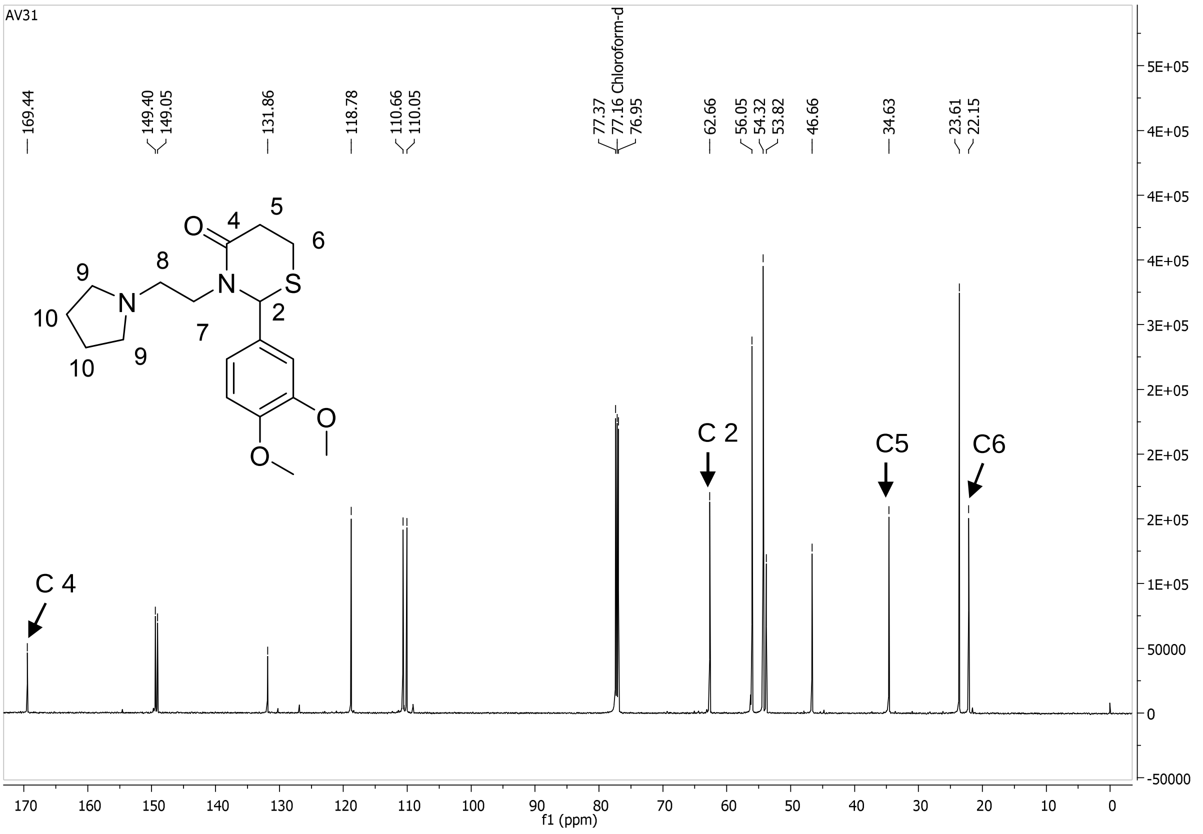

Figure S123: NMR ^{13}C spectrum of thiazinan-4-one **6x** .

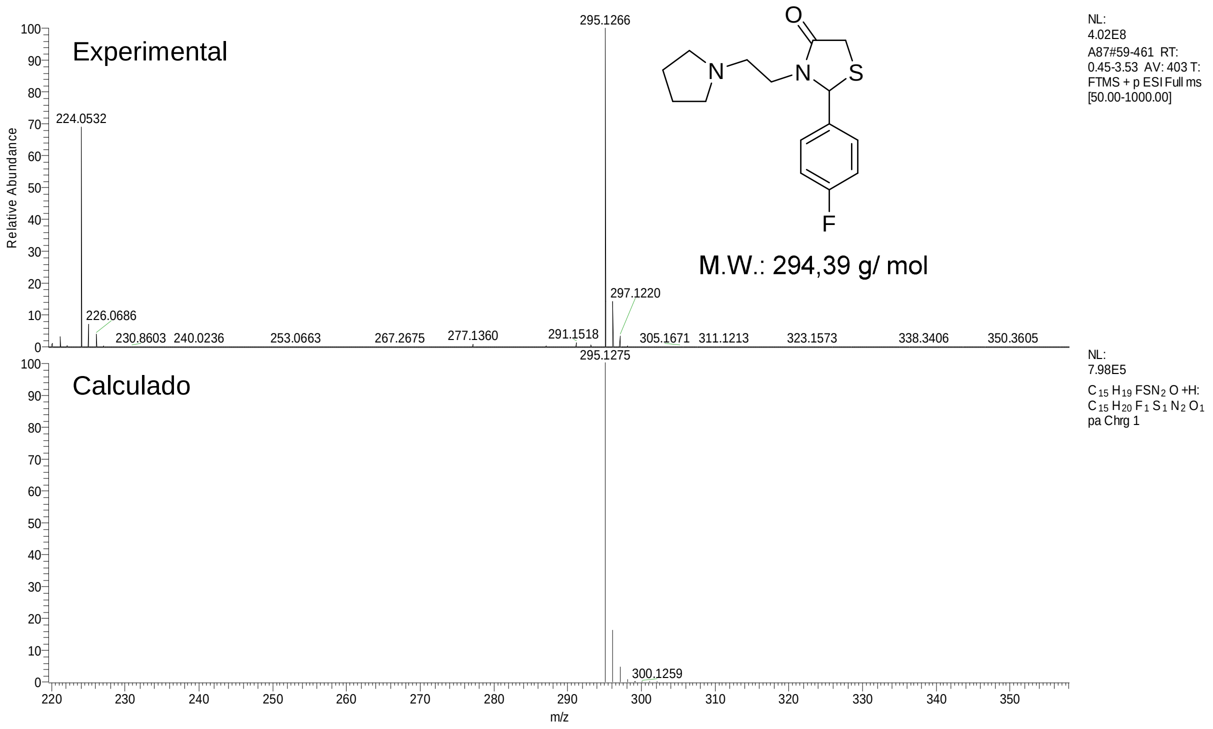

Figure S124: HRMS spectrum of thiazolidin-4-one **5d** (experimental up, calculated down).

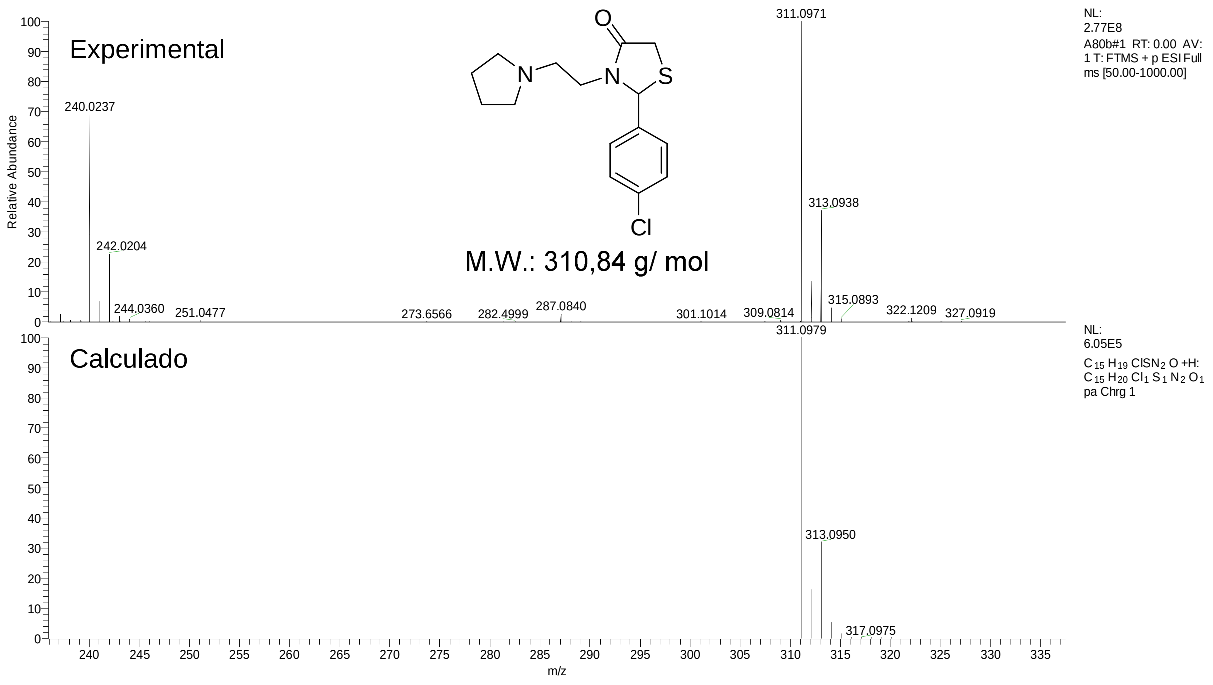

Figure S125: HRMS spectrum of thiazolidin-4-one **5g** (experimental up, calculated down).

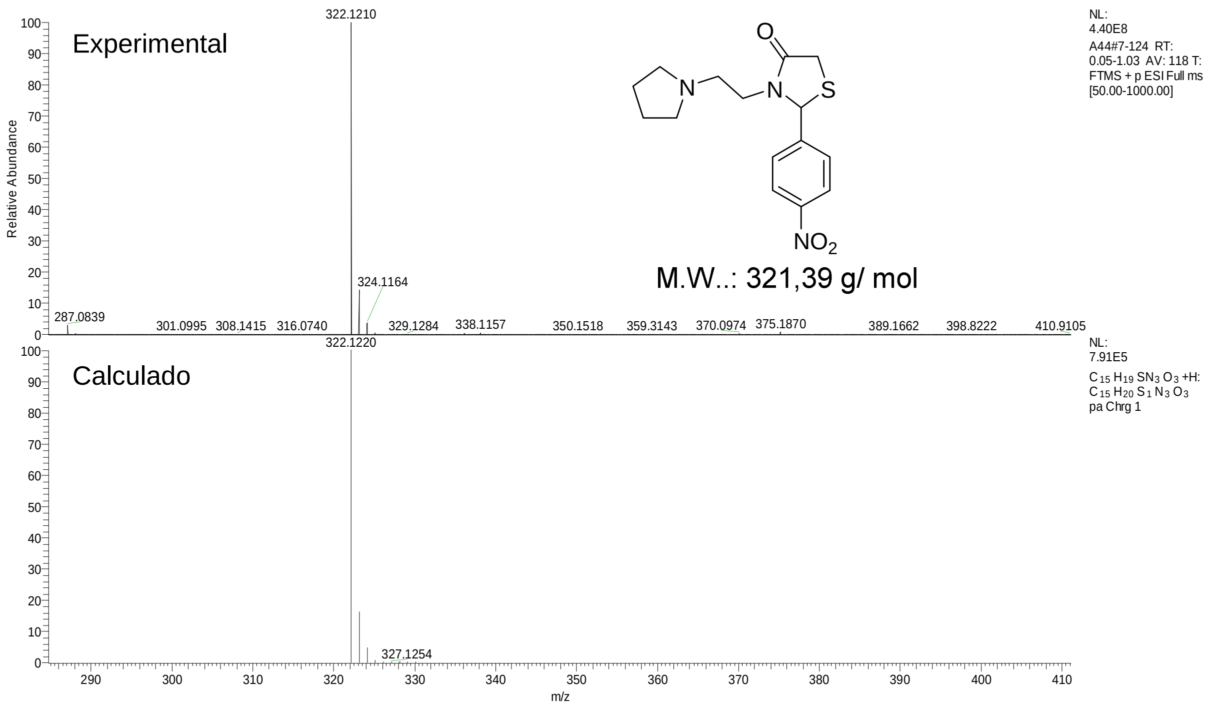

Figure S126: HRMS spectrum of thiazolidin-4-one **5j** (experimental up, calculated down).

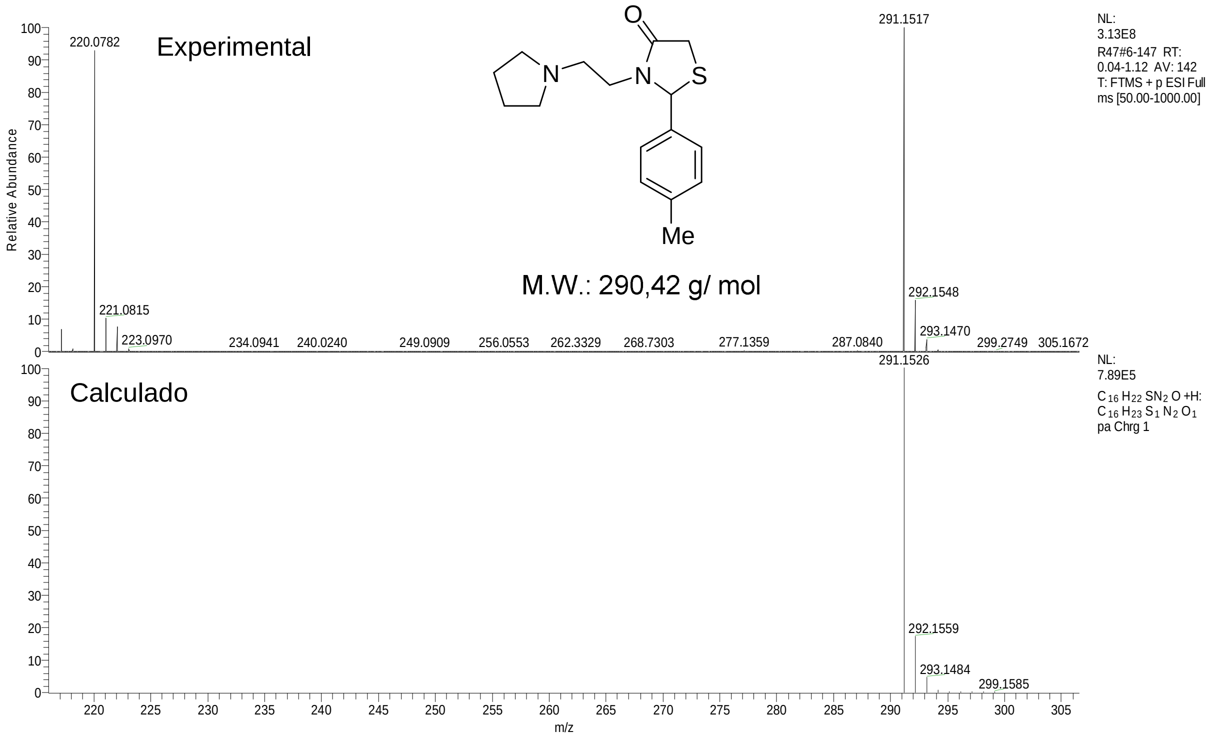

Figure S127: HRMS spectrum of thiazolidin-4-one **5k** (experimental up, calculated down).

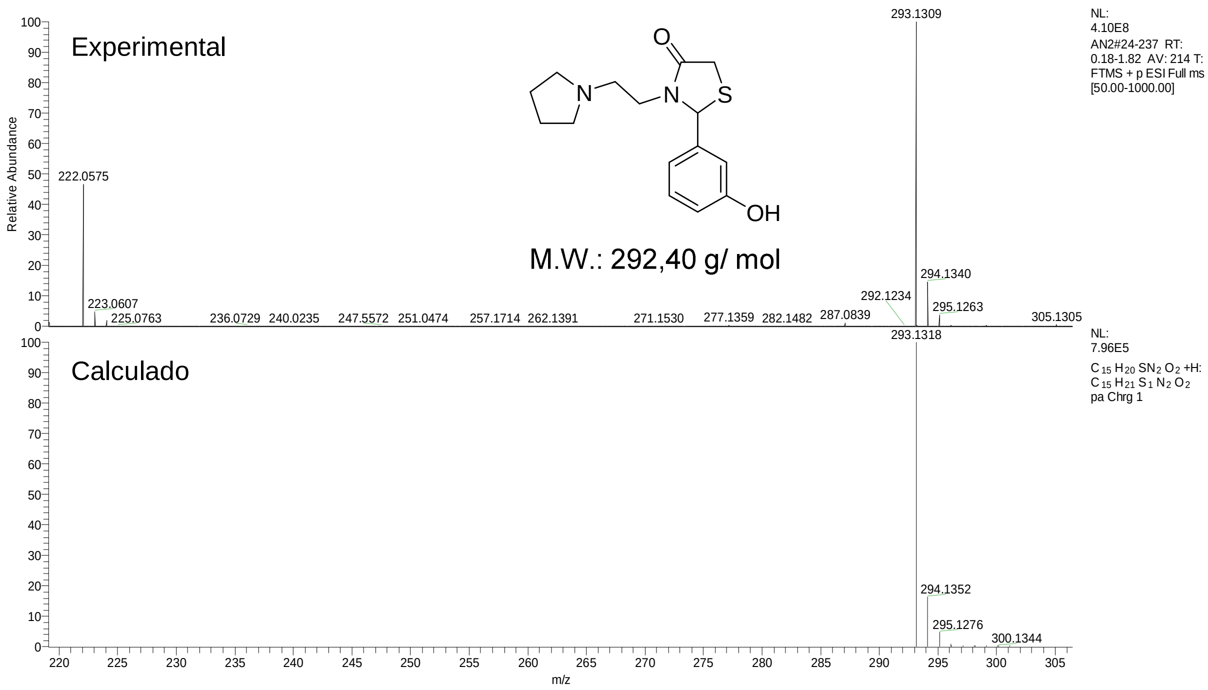

Figure S128: HRMS spectrum of thiazolidin-4-one **5o** (experimental up, calculated down).

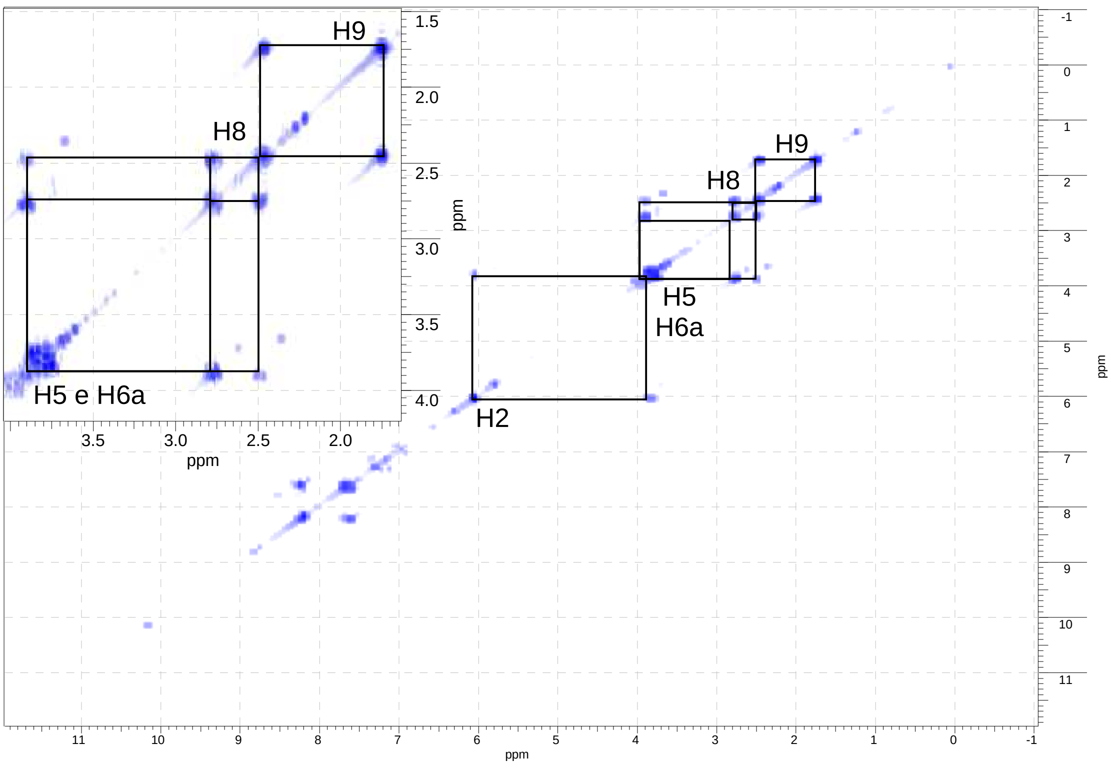

Figure S129: Spectrum 2D NMR COSY of thiazolidin-4-one **5i**.

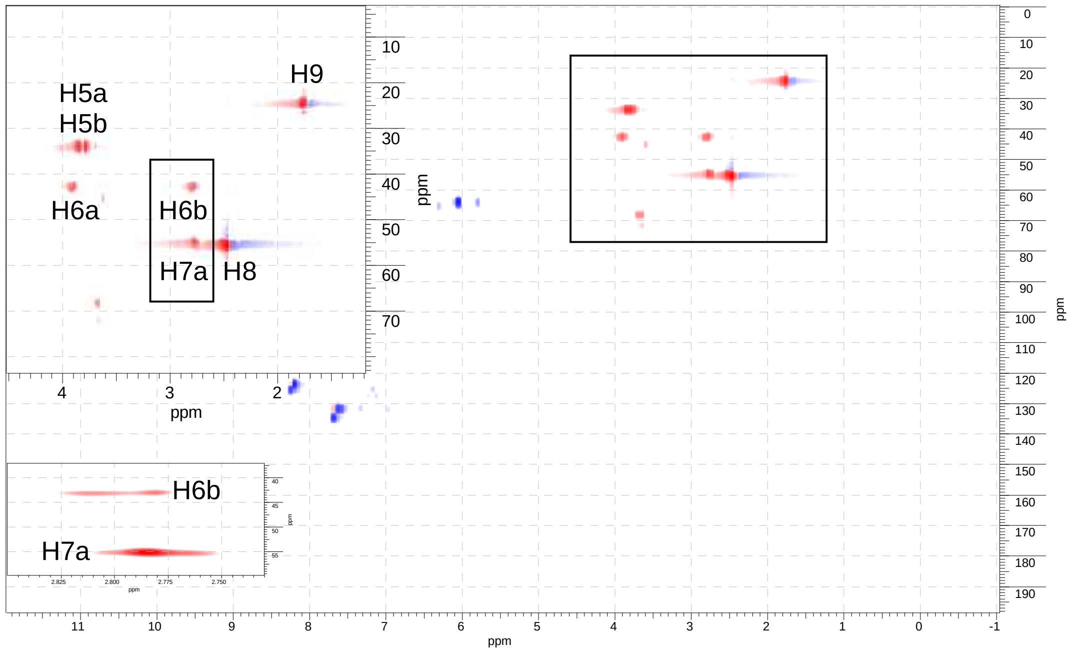

Figure S130: Spectrum 2D NMR HSQC of thiazolidin-4-one **5i**.

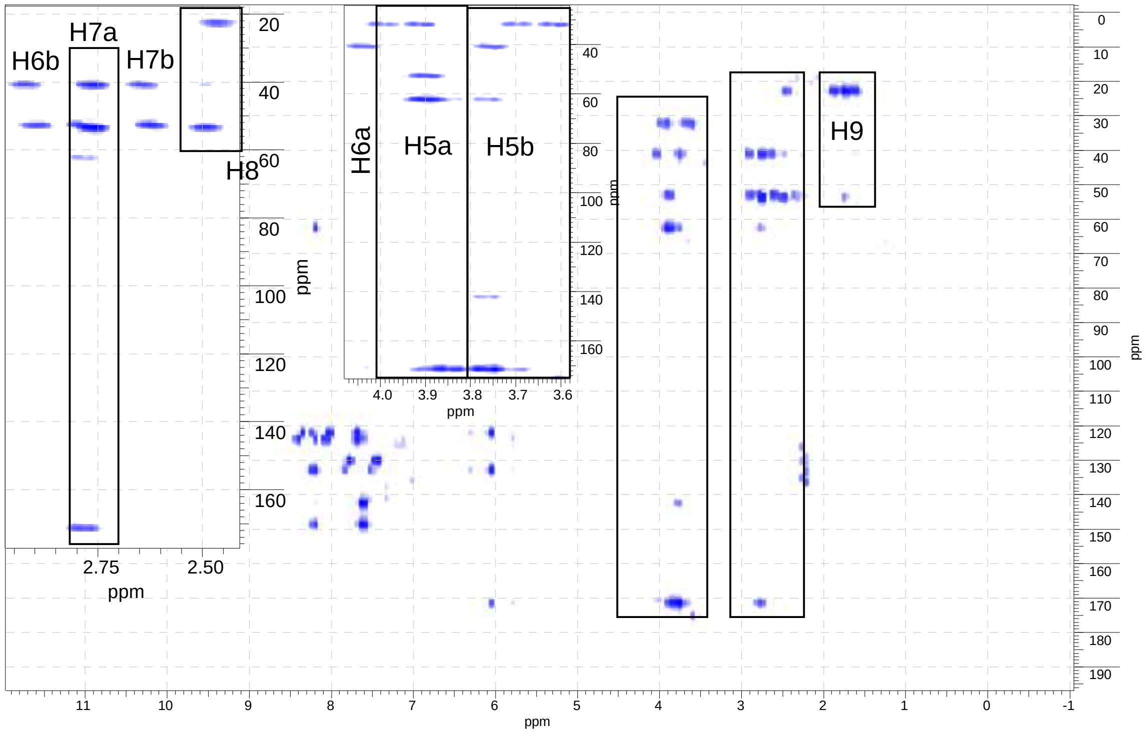

Figure S131: Spectrum 2D NMR HMBC of thiazolidin-4-one **5i**.

Table S1: Thiazolidin-4-ones **5** effects in vitro on the AChE activity in hippocampus and cerebral cortex of rats.

	Cerebral structure	Water	MeOH	0,1 μM	0,5 μM	1 μM	5 μM	10 μM	25 μM	50 μM	100 μM	250 μM
**5a**	Cerebral Cortex	2.59±0.05	2.74±0.08	-------------	-------------	2.12±0.14	2.09±0.12	2.19±0.15	2.01±0.14	2.10±0.17	2.04±0.10	1.66±0.19**
	Hippocampus	2.25±0.05	2.26±0.08	-------------	-------------	1.73±0.16	1.70±0.21	1.61±0.05*	1.44±0.22**	1.43±0.15**	1.49±0.23**	1.17±0.29***
**5d**	Cerebral Cortex	2.65±0.25	2.58±0.20	------------	-------------	2.75±0.35	2.75±0.13	2.59±0.24	2.46±0.18	2.36±0.17	1 .42±0.20*	0.77±0.06**
	Hippocampus	2.34±0.16	2.51±0.09	------------	-------------	1.99±0.16	1.86±0.14	1.67±0.21	1.78±0.08	1.75±0.04	1 .20±0.18***	1.25±0.08***
**5g**	Cerebral Cortex	2.65±0.25	2.58±0.20	-------------	-------------	2.55±0.22	2.52±0.17	2.79±0.10	2.71±0.13	2.03±0.035	2.09±0.045	2.00±0.050
	Hippocampus	2.09±0.29	2.15±0.27	-------------	-------------	2.20±0.13	2.31±0.19	2.06±0.19	2.20±0.19	2.15±0.12	1.56±0.06	1.20±0.08
**5h**	Cerebral Cortex	1.49±0.09	1.38±0.07	------------	-------------	1.16±0.09	1.12±0.10	1.19±0.13	1.22±0.17	1.27±0.06	1 .04±0.07	0.74±0.08*
	Hippocampus	1.97±0.14	1.77±0.13	------------	-------------	1.61±0.21	1.68±0.11	1.76±0.12	1.46±0.03	1.53±0.08	1 .29±0.05**	0.97±0.10***
**5i**	Cerebral Cortex	1.49±0.09	1.38±0.07	------------	-------------	1.16±0.17	1.03±0.013	0.92±0.17**	0.67±0.04***	0.57±0.06***	0.53±0.04***	0.48±0.12***
	Hippocampus	2.16±0.16	2.09±0.11	------------	-------------	2.09±0.19	1.92±0.08	1.57±0.08*	1.38±0.09**	1.23±0.09***	0.94±0.11***	0.48±0.10***
**5j**	Cerebral Cortex	2.46±0.14	2.46±0.12	2.32±0.17	2.28±0.20	1.85±0.08*	1.83±0.10*	1.79±0.16**	1.32±0.11***	1.19±0.12***	1.05±0.09***	0.69±0.06***
	Hippocampus	2.31±0.04	2.40±0.07	2.49±0.09	2.43±0.15	1.45±0.15***	1.46±0.08***	1.47±0.12***	1.19±0.11***	0.92±0.10***	0.93±0.61***	0.9125
**5k**	Cerebral Cortex	2.42±0.15	2.42±0.15	-------------	-------------	2.12±0.23	2.20±0.22	2.13±0.32	2.37±0.26	2.19±0.19	2.41±0.15	1.93±0.22
	Hippocampus	2.20±0.07	2.29±0.08	-------------	-------------	2.07±0.15	2.00±0.15	1.91±0.19	1.96±0.20	2.03±0.23	1.99±0.18	1.79±0.15
**5n**	Cerebral Cortex	2.56±0.18	2.49±0.18	------------	-------------	2.05±0.13	1.954±0.17	1.98±0.11	1.85±0.13	1.90±0.15	1.78±0.05*	1.05±0.15***
	Hippocampus	2.20±0.07	2.29±0.08	------------	-------------	2.16±0.15	1.80±0.20	1.74±0.19	1.56±0.13*	1.46±0.17*	1.54±0.14*	1.15±0.22***
**5o**	Cerebral Cortex	2.65±0.25	2.58±0.20	------------	-------------	2.92±0.28	2.46±0.17	2.13±0.26	1.64±0.18*	1.59±0.12*	1.17±0.08**	0.42±0.09***
	Hippocampus	2.29±0.19	2.51±0.09	------------	-------------	2.09±0.12	1.91±0.19	1.75±0.14	1.10±0.04***	0.86±0.08***	0.79±0.05***	0.31±0.07***

Table S2: Thiazinan-4-ones **6** effects in vitro on the AChE activity in hippocampus and cerebral cortex of rats.

	Structure	Water	MeOH	0,1 *μ*M	0,5 *μ*M	1 *μ*M	5 *μ*M	10 *μ*M	25 *μ*M	50 *μ*M	100 *μ*M	250 *μ*M
**6a**	Cerebral Cortex	2.57±0.13	2.62±0.17	2.52±0.14	2.48±0.18	1.9±0.15	1.83±0.19*	1.83±0.13*	1.82±0.14*	1.67±0.16**	1.61±0.12***	1.20±0.12***
	Hippocampus	2.31±0.04	2.40±0.07	2.54±0.10	2.48±0.07	1.53±0.17***	1.51±0.12***	1.15±0.14***	1.16±0.13****	1.27±0.14***	0.90±0.14***	0.73±0.06****
**6d**	Cerebral Cortex	3.13±0.20	2.94±0.13	-------------	-------------	3.15±0.30	2.92±0.10	3.04±0.35	3.02±0.29	2.74±0.09	2 .15±0.28	1.64±0.30**
	Hippocampus	2.30±0.14	2.25±0.13	-------------	-------------	2.10±0.14	1.89±0.14	1.91±0.20	1.96±0.19	1.52±0.16*	1.46±0.12*	0.64±0.10***
**6g**	Cerebral Cortex	2.65±0.25	2.58±0.20	------------	-------------	2.79±0.20	2.83±0.13	2.67±020	2.28±0.22	2.13±0.07	1.34±0.07**	1.03±0.18***
	Hippocampus	2.33±0.21	2.40±0.16	------------	-------------	2.33±0.16	2.51±0.18	2.17±022	1.86±0.13	1.58±0.19	0.83±0.01***	0.55±0.08***
**6h**	Cerebral Cortex	1.49±0.09	1.38±0.07	1.31±0.13	1.42±0.07	1.06±0.07*	1.01±0.08*	0.88±0.03***	0.60±0.05***	0.4 6±0.04***	0.49±0.12***	0.38±0.09***
	Hippocampus	2.39±0.10	2.46±0.15	2.20±0.07	2.27±0.11	2.15±0.13	1.57±0.05***	1.52±0.14***	1.37±0.10***	1. 16±0.06***	0.83±0.05***	0.31±0.04***
**6i**	Cerebral Cortex	1.49±0.09	1.38±0.07	-------------	-------------	1.28±0.10	1.30±0.15	1.28±0.06	1.26±0.05	0.85±0.02***	0.74±0.10***	0.45±0.01***
	Hippocampus	2.46±0.18	2.33±0.14	-------------	-------------	2.22±0.09	2.14±0.17	1.91±010	1.96±0.31	0.94±0.05***	0.81±0.01***	0.70±0.06***
**6j**	Cerebral Cortex	2.50±0.16	2.52±0.13	2.43±0.06	2.14±0.12	2.02±0.13	1.96±0.14	1.51±0.08***	1.55±0.08***	1.27± 0.10***	0.99±0.13***	0.42±0.08***
	Hippocampus	2.20±0.07	2.29±0.08	2.29±0.09	2.53±0.10	1.51±0.22*	1.37±0.11**	1.27±0.16***	0.90±0.08***	1.09±0.22***	0.83±0.12***	0.33±0.008***
**6k**	Cerebral Cortex	2.73±0.20	2.75±0.20	2.50±0.16	2.57±0.09	2.02±0.11	1.83±0.17*	1.72±0.15*	1.83±0.19*	1.88±0.21*	1.49±0.13**	0.64±0.06***
	Hippocampus	2.20±0.07	2.29±0.08	2.32±0.14	2.35±0.14	1.32±0.16***	1.29±0.10***	1.13±0.10***	0.83±0.11***	0.84±0.13***	0.66±0.12***	0.35±0.08***
**6l**	Cerebral Cortex	1.59±0.21	1.52±0.24	-------------	-------------	1.31±0.23	1.29±0.10	1.31±0.07	1.00±0.07	1.06±0.11	0 .86±0.02*	0.47±0.13***
	Hippocampus	2.05±0.01	2.04±0.19	-------------	-------------	1.99±0.10	2.09±0.04	1.77±0.11	1.58±0.16	1.51±0.06	1 .08±0.10***	0.34±0.06***
**6m**	Cerebral Cortex	1.49±0.09	1.38±0.07	-------------	-------------	1.34±0.04	1.19±0.09	1.22±0.14	1.01±0.09*	0.84±0.03**	0.62±0.10***	0.44±0.07***
	Hippocampus	2.40±0.12	2.38±0.11	-------------	-------------	2.39±0.10	2.07±0.20	1.81±0.18	1.68±0.09*	1.41±0.09***	1.18±0.03***	0.59±0.01***
**6n**	Cerebral Cortex	2.48±0.11	2.45±0.13	2.66±0.15	2.29±0.14	2.07±0.16	1.93±0.12*	1.92±0.08*	1.62±0.15***	1.10±0 .05***	0.86±0.06***	0.66±0.04***
	Hippocampus	2.31±0.04	2.40±0.07	2.55±0.07	2.31±0.07	1.34±0.07***	1.44±0.12***	1.29±0.06***	0.83±0.11***	0.92±0.10***	0.83±0.10***	0.34±0.08***
**6o**	Cerebral Cortex	2.65±0.25	2.58±0.20	-------------	-------------	2.65±0.17	2.39±0.14	2.26±0.19	1.72±0.17*	0.97±0.18***	0.68±0.24***	0.41±0.06***
	Hippocampus	2.12±0.06	2.13±0.17	-------------	-------------	2.12±0.21	2.22±0.26	2.23±0.12	1.45±0.07*	1.28±0.05**	1.17±0.11***	0.43±0.07***
**6p**	Cerebral Cortex	2.71±0.18	2.49±0.17	-------------	-------------	2.67±0.39	2.53±0.26	2.77±0.22	2.20±0.31	1.88±0.20	1 .61±0.14	1.12±0.16**
	Hippocampus	2.80±0.33	2.64±0.36	-------------	-------------	2.70±0.46	2.61±0.47	2.78±0.33	2.15±00.28	1.94±0.21	1.82±0.34	0.82±0.08*

Cerebral Cortex

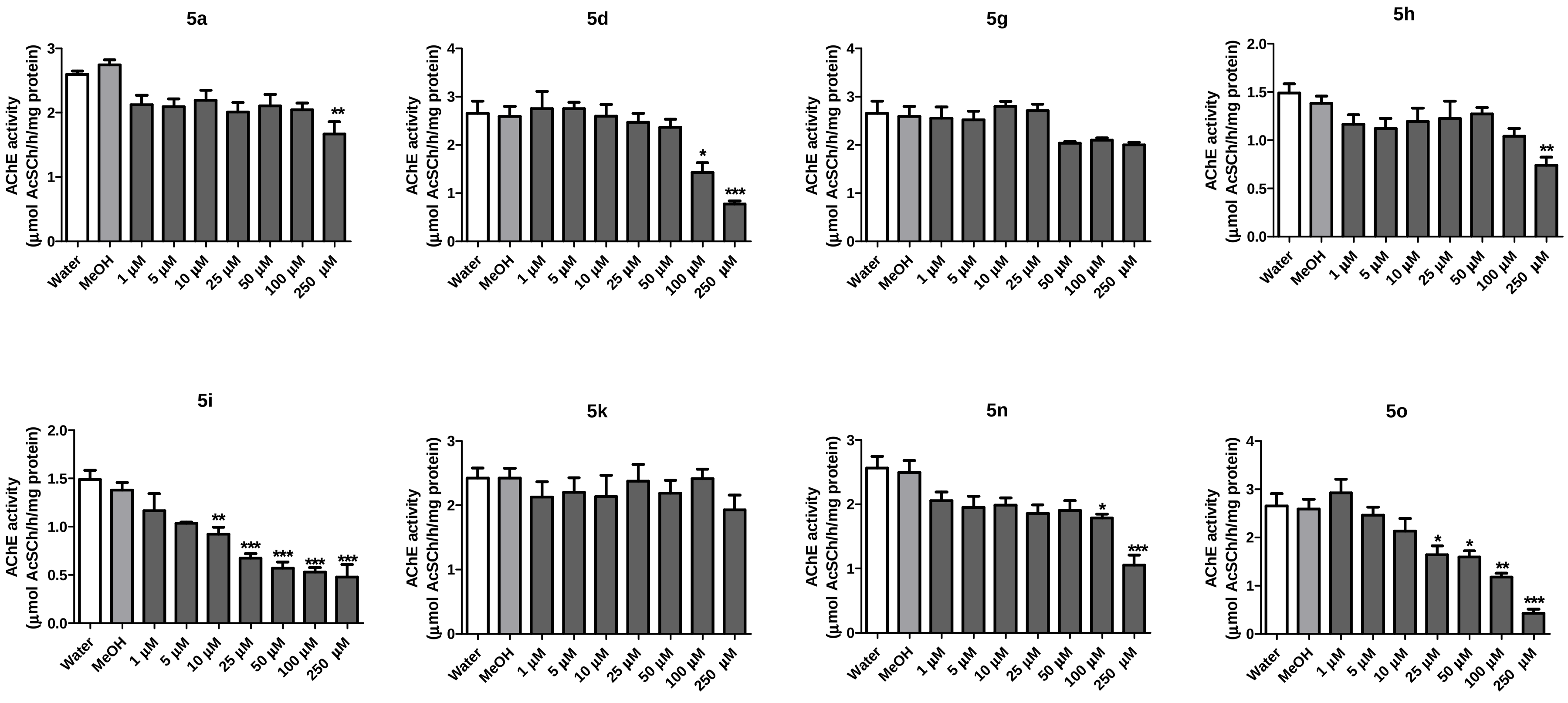

Figure S132: In vitro AChE activity of thiazolidin-4-ones **5** in cerebral cortex.

Hippocampus

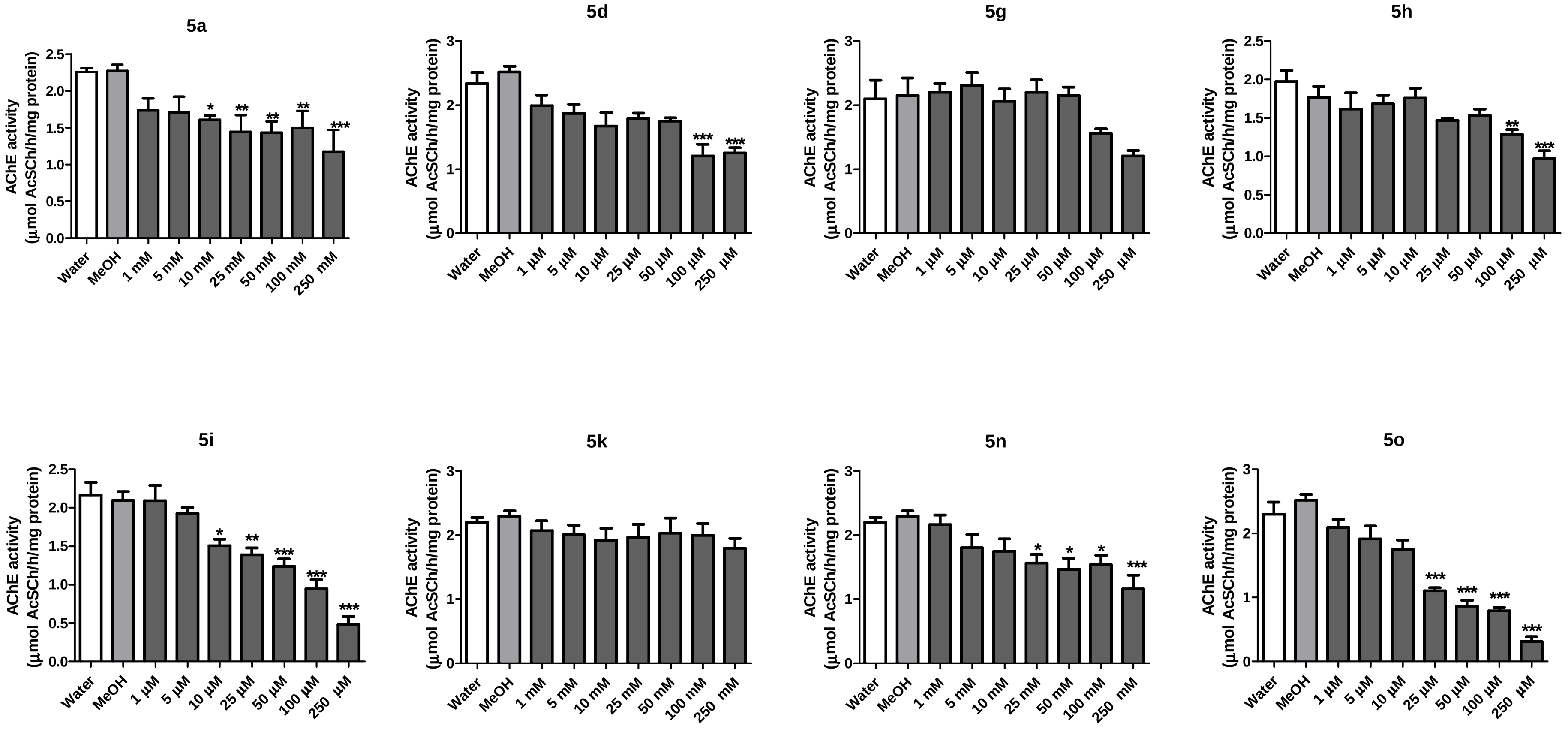

Figure S133: In vitro AChE activity of thiazolidin-4-ones **5** in hippocampus.

Cerebral Cortex

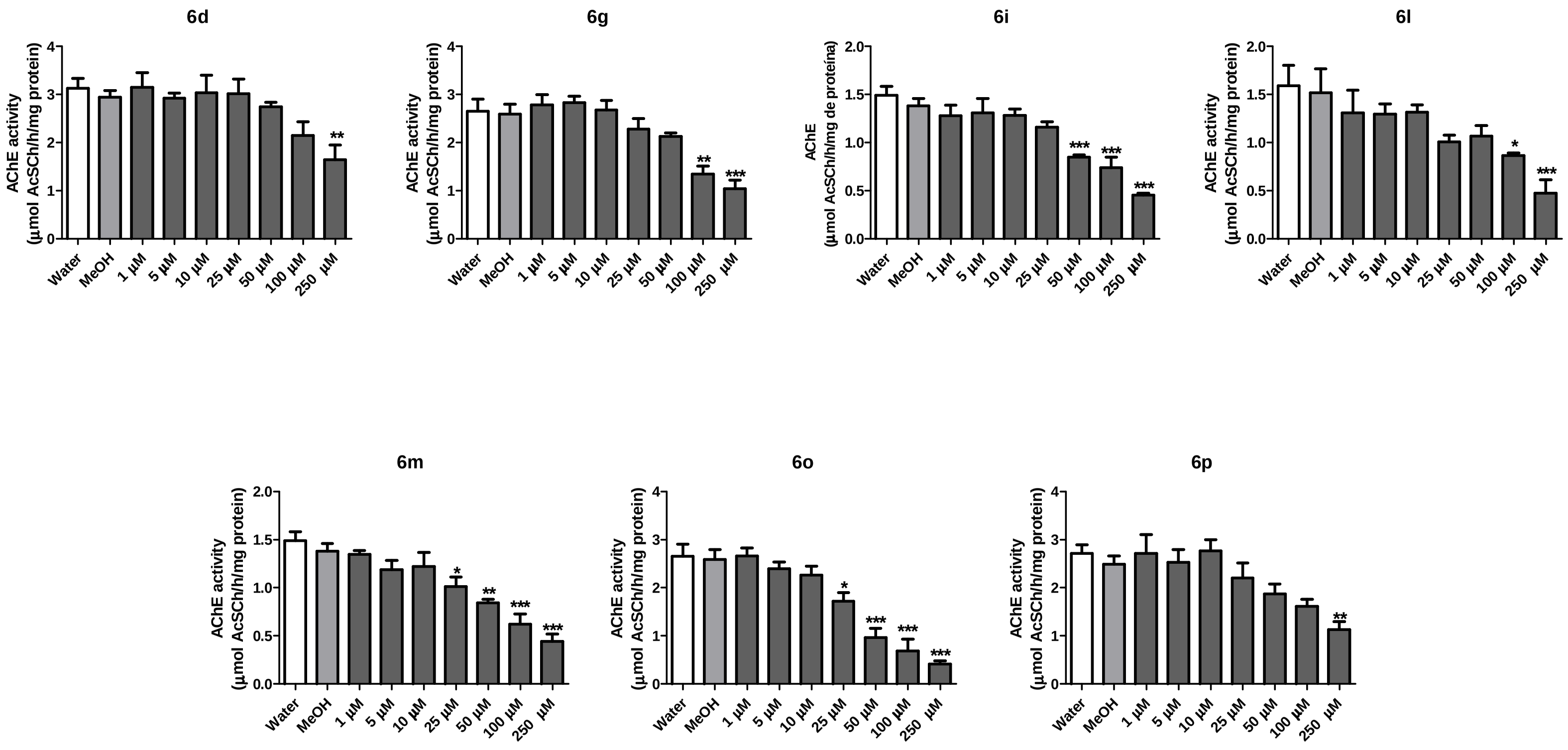

Figure S134: In vitro AChE activity of thiazinan-4-ones **6** in cerebral cortex.

Hippocampus

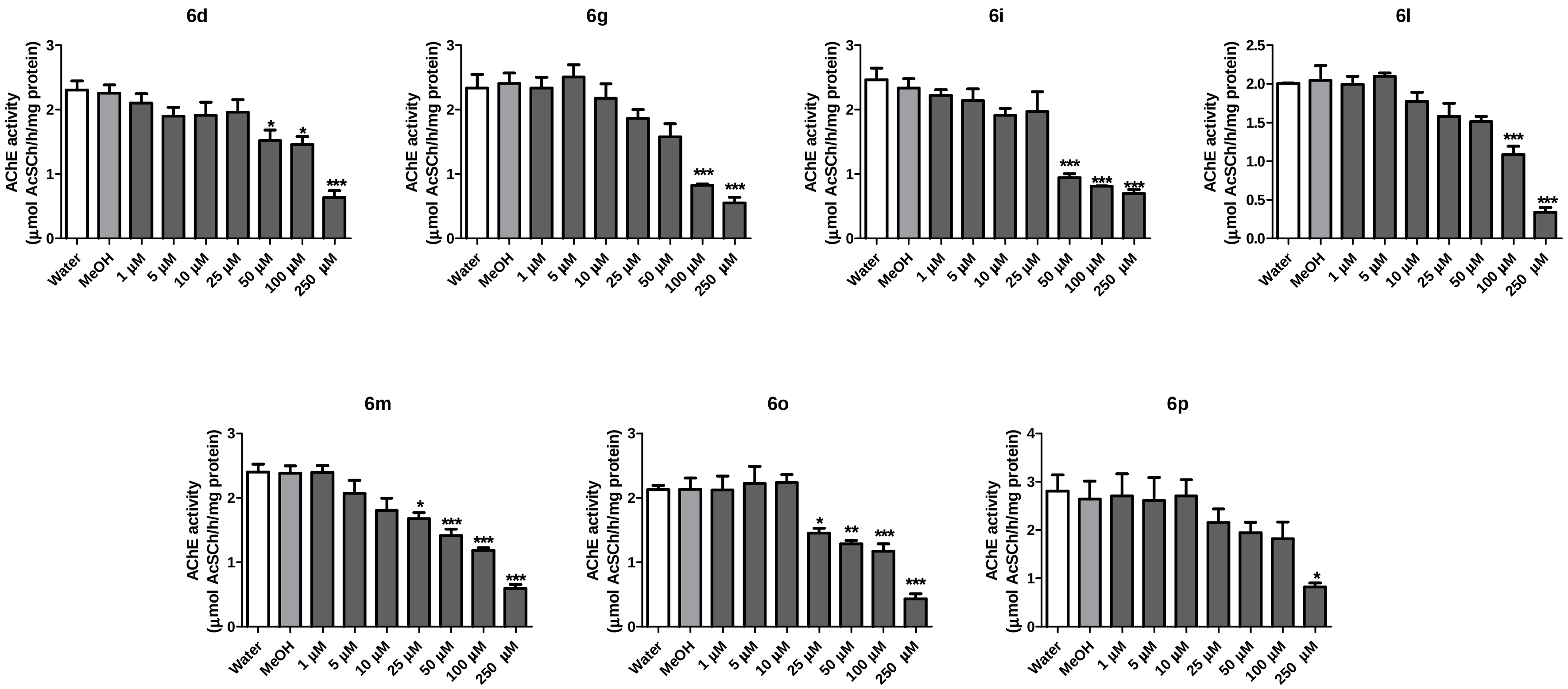

Figure S135: In vitro AChE activity of thiazinan-4-ones **6** in hippocampus.
